# Assessment of fine-tuned large language models for real-world chemistry and material science applications†

**DOI:** 10.1039/d4sc04401k

**Published:** 2024-11-22

**Authors:** Joren Van Herck, María Victoria Gil, Kevin Maik Jablonka, Alex Abrudan, Andy S. Anker, Mehrdad Asgari, Ben Blaiszik, Antonio Buffo, Leander Choudhury, Clemence Corminboeuf, Hilal Daglar, Amir Mohammad Elahi, Ian T. Foster, Susana Garcia, Matthew Garvin, Guillaume Godin, Lydia L. Good, Jianan Gu, Noémie Xiao Hu, Xin Jin, Tanja Junkers, Seda Keskin, Tuomas P. J. Knowles, Ruben Laplaza, Michele Lessona, Sauradeep Majumdar, Hossein Mashhadimoslem, Ruaraidh D. McIntosh, Seyed Mohamad Moosavi, Beatriz Mouriño, Francesca Nerli, Covadonga Pevida, Neda Poudineh, Mahyar Rajabi-Kochi, Kadi L. Saar, Fahimeh Hooriabad Saboor, Morteza Sagharichiha, K. J. Schmidt, Jiale Shi, Elena Simone, Dennis Svatunek, Marco Taddei, Igor Tetko, Domonkos Tolnai, Sahar Vahdatifar, Jonathan Whitmer, D. C. Florian Wieland, Regine Willumeit-Römer, Andreas Züttel, Berend Smit

**Affiliations:** a Laboratory of Molecular Simulation (LSMO), Institut des Sciences et Ingénierie Chimiques, École Polytechnique Fédérale de Lausanne (EPFL) Rue de l’Industrie 17 CH-1951 Sion Switzerland Berend.Smit@epfl.ch; b Instituto de Ciencia y TecnologÍa del Carbono (INCAR), CSIC Francisco Pintado Fe 26 33011 Oviedo Spain; c Laboratory of Organic and Tecnolog'ıa Chemistry (IOMC), Friedrich Schiller University Jena Humboldtstrasse 10 07743 Jena Germany; d Helmholtz Institute for Polymers in Energy Applications Jena (HIPOLE Jena) Lessingstrasse 12-14 07743 Jena Germany; e Yusuf Hamied Department of Chemistry, University of Cambridge Cambridge CB2 1EW UK; f Department of Energy Conversion and Storage, Technical University of Denmark DK-2800 Kgs. Lyngby Denmark; g Department of Chemistry, University of Oxford Oxford OX1 3TA UK; h Department of Chemical Engineering & Biotechnology, University of Cambridge Philippa Fawcett Drive Cambridge CB3 0AS UK; i Department of Computer Science, University of Chicago Chicago IL 60637 USA; j Data Science and Learning Division, Argonne National Laboratory Lemont IL 60439 USA; k Department of Applied Science and Technology (DISAT), Politecnico di Torino 10129 Turino Italy; l Laboratory of Catalysis and Organic Synthesis (LCSO), Institute of Chemical Sciences and Engineering (ISIC), École Polytechnique Fédérale de Lausanne (EPFL) CH-1015 Lausanne Switzerland; m Laboratory for Computational Molecular Design (LCMD), Institute of Chemical Sciences and Engineering (ISIC), École Polytechnique Fédérale de Lausanne (EPFL) CH-1015 Lausanne Switzerland; n Department of Chemical and Biological Engineering, Koç University Rumelifeneri Yolu, Sariyer 34450 Istanbul Turkey; o The Research Centre for Carbon Solutions (RCCS), School of Engineering and Physical Sciences, Heriot-Watt University Edinburgh EH14 4AS UK; p BIGCHEM GmbH Valerystraße 49 85716 Unterschleißheim Germany; q Laboratory of Chemical Physics, National Institute of Diabetes and Digestive and Kidney Diseases, National Institutes of Health Bethesda Maryland 20892 USA; r Institute of Metallic Biomaterials, Helmholtz Zentrum Hereon Geesthacht Germany; s Polymer Reaction Design Group, School of Chemistry, Monash University Clayton VIC 3800 Australia; t Cavendish Laboratory, Department of Physics, University of Cambridge Cambridge CB3 0HE UK; u Department of Chemical Engineering, University of Waterloo Waterloo N2L3G1 Canada; v Institute of Chemical Sciences, School of Engineering and Physical Sciences, Heriot-Watt University Edinburgh EH14 4AS UK; w Chemical Engineering & Applied Chemistry, University of Toronto Toronto Ontario M5S 3E5 Canada; x Dipartimento di Chimica e Chimica Industriale, Unità di Ricerca INSTM, Università di Pisa Via Giuseppe Moruzzi 13 56124 Pisa Italy; y Chemical Engineering Department, University of Mohaghegh Ardabili P. O. Box 179 Ardabil Iran; z Department of Chemical Engineering, College of Engineering, University of Tehran Tehran Iran; a Department of Chemical Engineering, Massachusetts Institute of Technology Cambridge MA 02139 USA; b Department of Chemical and Biomolecular Engineering, University of Notre Dame Notre Dame Indiana 46556 USA; c Institute of Applied Synthetic Chemistry, TU Wien Getreidemarkt 9 1060 Vienna Austria; d Institute of Structural Biology, Molecular Targets and Therapeutics Center, Helmholtz Munich - Deutsches Forschungszentrum für Gesundheit und Umwelt (GmbH) Ingolstädter Landstraße 1 85764 Neuherberg Germany; e Department of Chemistry and Biochemistry, University of Notre Dame Notre Dame Indiana 46556 USA; f Laboratory of Materials for Renewable Energy (LMER), Institut des Sciences et Ingénierie Chimiques, École Polytechnique Fédérale de Lausanne (EPFL) Rue de l'Industrie 17 CH-1951 Sion Switzerland

## Abstract

The current generation of large language models (LLMs) has limited chemical knowledge. Recently, it has been shown that these LLMs can learn and predict chemical properties through fine-tuning. Using natural language to train machine learning models opens doors to a wider chemical audience, as field-specific featurization techniques can be omitted. In this work, we explore the potential and limitations of this approach. We studied the performance of fine-tuning three open-source LLMs (GPT-J-6B, Llama-3.1-8B, and Mistral-7B) for a range of different chemical questions. We benchmark their performances against “traditional” machine learning models and find that, in most cases, the fine-tuning approach is superior for a simple classification problem. Depending on the size of the dataset and the type of questions, we also successfully address more sophisticated problems. The most important conclusions of this work are that, for all datasets considered, their conversion into an LLM fine-tuning training set is straightforward and that fine-tuning with even relatively small datasets leads to predictive models. These results suggest that the systematic use of LLMs to guide experiments and simulations will be a powerful technique in any research study, significantly reducing unnecessary experiments or computations.

## Abstract 2

We studied the performance of fine-tuning open-source LLMs for a range of different chemical questions. We benchmark their performances against “traditional” machine learning models and find that, in most cases, the fine-tuning approach is superior.

## Graphical abstract

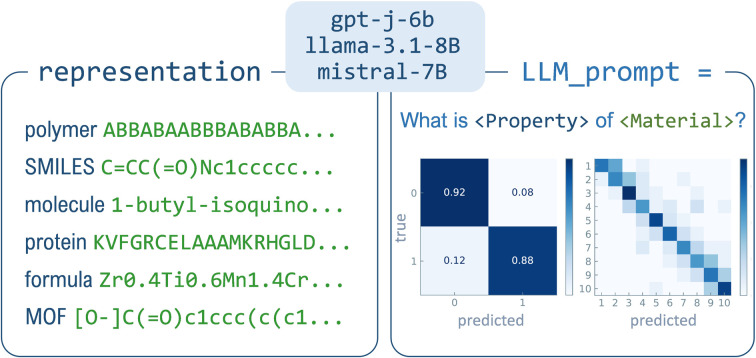

## 1 Introduction

The traditional machine-learning workflow starts with the painstaking process of harvesting the literature for the relevant data. Some help can be obtained from text harvesting programs.^1^ These data can be used to find correlations in the properties or synthesis of molecules or materials or correlations in any other relevant chemical question. For this, it is crucial to describe the system with features fed into a model. Ultimately, the trained model allows us to make predictions from the features of unknown materials. These models typically improve when more data becomes available.

In chemistry and material science, however, the amount of experimental data is often, if not always, a bottleneck. Therefore, it is essential to have some leverage. One way of doing this is by expanding a dataset with computer simulations.^2^ Alternatively, we can leverage knowledge of the system. For example, suppose we want to predict the pressure of a gas at a given density and temperature; we can focus our machine learning (ML) on predicting the deviations from the ideal gas law.^3^ Another option is to introduce descriptors with proper inductive biases that capture our understanding of the underlying systems.^4^

Another way of leveraging knowledge is through transfer learning. Imagine that one has a lot of data on some particular properties of a class of materials but, as is typical in many practical applications, not enough data for the property of interest. The idea of transfer learning is that we can train a model on the properties for which we have a lot of data and subsequently fine-tune this model for the property of interest.^5^ The mechanism of this fine-tuning or transfer learning is that one only retrains a small part of, for example, a transformer model (or of an added layer) and hence leverages all the pre-trained information locked in the model's part that remains unchanged. This fine-tuned model can then be used to make predictions for these properties.

In this context, a remarkable recent discovery is that one can also use fine-tuned large language models (LLMs) to answer chemistry and material science questions for which the base LLM would not know the answer.^6,7^ LLMs are pre-trained (without supervision) on web-scale data. Their training is to predict the next likely character (or word) to complete a sentence. For example, if we use GPT-3 (*e.g.*, *via* ChatGPT) to ask a specific chemical question, say, if the high-entropy alloy Tb_0.5_Y_0.5_ is a single phase, it will reproduce the knowledge is has. GPT-3 would not know the answer (GPT-4 knows more chemistry^8^). Hence, it will likely not get an answer to such chemical questions. However, we can fine-tune an LLM with experimental data of high-entropy alloys, of which we know whether it is a single phase or not. This gives us a new model that only aims to predict whether a particular high entropy alloy is a single phase.

In addition, the fact that these LLMs use natural language as input instead of a descriptor is one of its most attractive features; it creates a convenient way for researchers to interact with data and tools. Numerous successful chemical applications exploiting this power of LLMs exist today, ranging from tools that summarize literature to the deployment of “chatbots” for experimental instrumentation.^9^

These general-purpose LLMs are important because they do not require pre-training, can be used for any chemical question, and do not require knowledge of machine learning. In our previous work, we measured the potential of LLMs in solving chemical problems against conventional machine learning specifically developed and optimized for that problem.^7^ We showed that LLM models fine-tuned on classification, regression, and inverse design problems can be competitive with current state-of-the-art machine learning models. For this, we searched for chemical problems with a known ML solution and validated our approach against it.

In this work, we want to go a step further and attempt to address relevant chemical questions from a more practical point of view. This implies that most of the data have not been curated or selected previously for machine learning studies but are the data that researchers have at hand. The case studies we present are guided by the questions these researchers have.

With this data, we performed simple “experiments”. First, we asked whether fine-tuned LLMs show any signs of learning. To address this question, we split the dataset in half and used a simple classification to test if the model would classify the data correctly on a holdout set that the model has not seen in training. Accurate binary classifications can be particularly useful in experimental scenarios where precise numerical values are not necessary, simplifying and facilitating the decision-making process in routine research. Such classifiers can already be of great practical interest, as they mimic daily “yes” or “no” questions of researchers, *e.g.*, “Can we synthesize this molecule?” or “Will property X of this molecule be high or low?”. Having access to accurate predictions of the answer of these questions has the potential to facilitate chemical workflows, reducing computational or experimental resources. Technically, this first step involved the fine-tuning of a model using a standard setting without any optimization. For this step, we used open-source models, which we tuned using parameter-efficient fine-tuning techniques.^10–12^ The models showed some learning for almost all problems. The extent of learning depends on the dataset and the complexity of the question. Inevitably, the performance of such models will not be optimal for every study; therefore, we optimized the models by performing basic hyperparameter optimization in those cases.

In the following sections, we outline the methodology and then summarize the main conclusions of each case study. The corresponding section of the ESI† provides a detailed account of each study. The main aim of these summaries is to illustrate the range of chemical questions that can be addressed. The discussion section summarizes the lessons we have learned from these case studies.

## 2 Methods

Researchers from different disciplines presented 22 datasets. We used these datasets in case studies to better understand the potential and limitations of fine-tuning LLMs. For these case studies, we used three open-source LLMs: GPT-J-6B,^13^ Llama-3.1-8M,^14^ and Mistral-7B.^15^ The LLMs used are smaller models than, for example, GPT-3 (and GPT-4). Our previous results show that GPT-3 typically performs better, *i.e.*, it requires less training data to get similar performance.^7^ However, this increase in accuracy does not compensate for the fact that with open-source models, anybody can reproduce our results.

To present the results in a structured manner, we have organized the case studies into three categories: Materials and properties, Reactions and synthesis, and Systems and applications.

Each case study was approached similarly. The first step requires converting the dataset into a set of questions and answers that we can use for fine-tuning. We obtained some general knowledge of the systems' scientific background for this. This background is given in more detail in the ESI† and summarized at the beginning of each paragraph describing the case studies. In the ESI,† one can find more details on how the dataset was obtained.

The first test we carried out was a standard test to determine if our fine-tuned LLM learned anything. This test was a simple classification problem in which we split the dataset into two equally populated categories. Depending on the case study, these categories were high/low, good/bad, optimal/non-optimal, *etc.* This simple classification allows for a simple benchmark: random guessing. The minimum criterion the LLM should outperform on the test set is to do better than random guessing. This random guess corresponds to the situation where we have zero knowledge of the system. We will refer to this experiment as the “base case”. Hence, any model that does better might be of practical use. In addition, we also compared the performance of LLMs with that of two “traditional” ML models, *i.e.*, random forest (RF) and XGBoost. Even before training and using these models, the potential advantages of LLMs became apparent here. As these traditional models require numeric inputs, an additional step was often necessary to convert the features of the received datasets.

If our base case model outperformed the benchmark, the next step is to make the LLM more useful. In most practical applications, one has more data on poor-performing materials than optimal materials. However, for fine-tuning LLMs, one also needs a reasonable number of materials above the performance threshold, distinguishing poor from top-performing materials. This may require more data than we have available for a specific case study and may require us to optimize the model further. This part will be specific to each case study.

We follow the same fine-tuning method as in our previous work (the reader is referred to the original work^7^ for details on the fine-tuning) except that we now used GPT-J-6B, Llama-3.1-8B, and Mistral-7B. In this procedure, the chemical context is formatted in a single representation as a question (Table 1). The binary class is given as a numeric value, *i.e.*, 0 or 1, representing the respective chemical property.

**Table 1:** Example of a prompt used to fine-tune an LLM. This example is a typical classification problem where we have split the dataset into two groups. In the example, we have a 〈Property〉 of a 〈Material〉, which can either be high or low. What is high or low is determined by the threshold we want to use. In practice, we want the model to predict 0 or 1, so in all training sets, we see that good/bad, single phase/two phases, for example, will be translated to 0 or 1

Representation	Completion	Real
What is 〈Property〉 of 〈Materia〉?	0 or 1	Low or high

The first iteration used default fine-tuning hyperparameter values (see ESI Note 2†). This allowed us to gain some insights into whether such an approach can be used as a black box without expertise in using LLMs or if some tweaking is needed to get sufficiently accurate results. After analyzing the first result, in some case studies, increasing the number of epochs, *i.e.*, the times the model sees the training data, significantly increased the model's performance. This gives us insights into the fine-tuning procedure. This second step typically requires some more experience with these LLMs.

## 3 Results and discussion – case studies

### 3.1 Materials and properties

Following a bottom-up approach, most chemical applications start from fundamental research of the structure–property relationships. Therefore, it is no surprise that a thorough understanding of the structures at hand is necessary before proceeding to field-specific applications. However, the chemical space is vast and complex, and finding optimal solutions often requires extensive screening and analysis. Alternatively, chemical properties can be predicted from the structural features of a molecule.^16^ We demonstrate how our approach can help predict chemical properties in various case studies and more effectively guide computational and experimental research.

#### 3.1.1 Adhesive energy of polymers

As a first example, we experimented with a computational dataset of polymers and their respective adhesive free-energy on a polymer surface (see ESI Note 3.1†).^17^ Here, the question arises if we can predict the adhesive free-energy from any hypothetical copolymer sequence. The polymers in the dataset were chains of 20 monomers, described as either an “A” or a “B” unit. We used the sequence string of the polymers to predict the balanced binary classification of the adhesive free-energy (*i.e.*, if this free energy is high or low). The fine-tuned LLM Llama provided an accuracy of 96%, which is notably above the random baseline (50%) and slightly higher than the performance of random forest (90%) and XGBoost (94%) models.

The remarkable aspect of these results is that we have a hypothetical model polymer for which simulations compute the free energies. Yet, the LLM can correlate a sequence of 20 (arbitrarily chosen) characters of the type “A” and “B” to the free energy, suggesting no potential data leakage.

#### 3.1.2 Properties of monomers

Focusing on a more standardized and widespread descriptor of molecular structures, we investigated the Simplified Molecular-Input Line-Entry System (SMILES) notation. These textual strings capture the elemental composition, bonds, branches, and stereochemistry of chemical compounds. The monomer database, computationally generated by Schneider *et al.*,^18^ served as an ideal test case to validate the synergy of SMILES and LLMs (see ESI Note 3.2†). Schneider *et al.*^18^ obtained from simulation many different properties, including the glass transition temperature, cohesive energy density, squared radius of gyration, and density of a wide range of monomers. We obtained four unique binary classification case studies by taking the median for every property. We fine-tuned LLMs to predict the specific property from the monomer's SMILES. For all cases, an accuracy above 75% (average accuracy of 84% over the four properties) with non-optimized hyperparameters was obtained for the GPT-J model (similar performances were obtained with Llama (83%) and Mistral (83%)). The fine-tuned LLMs even outperformed traditional ML models.

#### 3.1.3 Melting point of molecules

The following case study concerned the prediction of the melting point of small molecules (see ESI Note 3.3†). The 274,983 structures were all represented by their SMILES notation and IUPAC name. Therefore, in this particular example, we further explored the chemical representation and how it affects the quality of the predictions.

As the melting point of many chemicals is reported, we first studied how well ChatGPT (OpenAI's GPT-3.5) can classify the melting point as high or low. Using the front-end interface, we prompted “What is the melting point of 〈name of molecule〉?” and saw that only 50% of the time it predicted this correctly, which is no better than random guessing. In contrast, models trained on the IUPAC name reached an accuracy of 66%. Interestingly, our fine-tuned models trained on the SMILES of the molecules could predict the melting point with an accuracy of 69% (GPT-J). The fine-tuned model proves to compete with traditional ML models.

#### 3.1.4 Dynamic viscosity of molecules

In this case study, we also used the SMILES notation of some molecules. Our objective was to predict their dynamic viscosity with fine-tuned LLMs (see ESI Note 3.4†).

As the dynamic viscosity value of many chemicals is also reported, we evaluated (*via* ChatGPT) how well OpenAI's GPT-3.5 model can classify the viscosity as high or low. Our prompt was “What is the dynamic viscosity of 〈name of molecule〉?” Our results showed that viscosity was not better predicted than random guessing, with an accuracy of 55% when the chemical name was provided as input to the model.

In contrast, for a median split balanced dataset, with a training set size of 80 examples and 30 fine-tuning epochs, the fine-tuned LLM model GPT-J reached an accuracy of 80% for binary classification (which is comparable to traditional ML models). We also trained a model to predict whether a chemical had a dynamic viscosity in the top 28% of the values in the dataset. After reducing the dataset size to obtain a balanced dataset, we also obtained a predictive accuracy of 80% (GPT-J) using a training set of 50 data points by increasing the number of fine-tuning epochs to 140.

#### 3.1.5 Microstructural properties of magnesium alloys

Due to their lightweight, Mg alloys gain popularity in structural applications where weight saving is of importance.^19^ Besides a small portion produced with powder-metallurgical processing,^20–22^ the majority of these alloys are cast and subsequently subjected to thermo-mechanical treatment to obtain a microstructure corresponding to a suitable property profile.^23,24^ To understand the connection between process routes, their specific processing parameters, and microstructure evolution, we can use the LLM's modular and versatile way of featurizing to include all relevant parameters, independent of the production route (see ESI Note 3.5†).

We found that the fine-tuned models can deal with incomplete and multivariable inputs, reaching accuracies of 94% (Mistral, comparable to traditional ML models) to predict the classification of the material either belonging to the class high or low amount of second phases. Interestingly, we acquired similar accuracies when only using the production route to represent the model irrespective of the individual process parameters. Despite the small dataset, the LLM is able to catch the material science properties and classify them accordingly.

#### 3.1.6 Phase separation propensity of proteins

Phase separation of proteins and other biomolecules is recognized as an important intracellular process that affects cellular compartmentalization and regulation.^25^ However, the mechanisms that drive biomolecular phase behavior are still under active investigation. Saar *et al.*^26^ performed an *in silico* study to understand the link between protein sequence and its liquid–liquid phase separation (LLPS). This is an interesting challenge from a data-science perspective, as the protein sequences are long strings of letters, each representing a single amino acid. Such non-numeric input often requires additional data pre-processing steps and/or dedicated statistical techniques. Saar *et al.*^26^ developed binary classifiers based on extracted physical features of the protein sequence and a word embedding of the sequence made using a word2vec model. From these sequence-based embeddings, the model was able to classify proteins based on their propensity to undergo LLPS. Identifying proteins capable of undergoing LLPS into protein-rich biomolecular condensates is important for understanding cellular function and pathology.

This is an interesting case for an LLM model, as a protein represented by a string, like RRGDGRRRG…GGGRGQGGRGR, can be inputted directly in the prompt (see ESI Note 3.6†). We obtained accuracies reaching 95% (GPT-J) for models that distinguish proteins on their phase separation propensity, which is similar to the accuracy obtained by Saar *et al.*.^26^ We want to stress that no extra data manipulation was needed. The protein sequence as received was used as input for the prompt, again demonstrating the versatility of LLMs.

In addition, we carried out some experiments in which we changed the original sequences (*e.g.*, making them shorter or creating randomized sequences of the same letter/amino acid composition). The most interesting observation was that a model trained on randomized sequences of the same letters resulted in a relatively small drop in accuracy from 95% to 86%, which shows that a significant part (but not all) of the predictive ability can be obtained from the protein's sequence composition without any positional information of sequence order. Interestingly, the addition of positional information was also not found to increase the performance in predicting the apparent and shear modulus of materials.^27^

#### 3.1.7 Structure of nanoparticles

Developing new nanomaterials for energy technologies requires a deep understanding of the intricate relation between material properties and atomic structure. Solving the atomic structure of nanomaterials from their X-ray total scattering data is challenging. Generative models such as Conditional Variational Autoencoder (CVAE) have been proposed to obtain valid chemical structures from the scattering pattern.^28,29^ In this case study, we predict the structure type and number of atoms in nanomaterials from their scattering pattern. Predicting these values accurately is easier than solving the structure, and LLMs are more convenient for researchers than CVAEs as they are purely based on natural language.

This is also an interesting case for an LLM model since the scattering pattern consists of a very long series of numbers represented as a string. With our approach, we obtained an accuracy of 97% (Mistral) to predict the structure type of nanoparticles from scattering patterns simulated from 7 highly unbalanced structure types with between 5 and 100 atoms (30 epochs, 1800 data points). We found that, for complex input variables, where the information is embedded along long sequences, using a relatively large training set size, the fine-tuned model can predict an unbalanced dataset with 7 classes. However, if the number of training data points is very low (200), the fine-tuned model is not even predictive on a balanced dataset.

For the prediction of the number of atoms in the nanomaterial, we obtained accuracies of 98% (Mistral) and 93% (Llama) for datasets with 4 and 10 balanced classes, respectively.

These results are comparable to those obtained with traditional ML models for the 4-class dataset but notably superior to those obtained with ML models for the more challenging 10-class classification task. However, from a practical point of view, given the interest in predicting the number of atoms with very high accuracy, we also developed a regression model. The LLM regression models predicted the number of atoms with an *R*^2^ of 99% (Llama and Mistral) and a maximum absolute error (MAE) below one atom (for comparison, *R*^2^ of random forest and XGBoost was 93% and 94%, respectively, while MAE was 5.1 and 4.7, respectively), *i.e.*, LLMs showed an excellent performance.

#### 3.1.8 Melting temperature of triacylglycerols

Fats and oils are important ingredients for various industries, from food to cosmetics. They are primarily composed of triacylglycerols (TAGs). The chemistry of these TAGs influences physical properties, such as the melting point. In this case study, we aimed to predict the melting point from the chemical composition of TAGs.

The various notations of 211 TAGs provided an interesting test case to examine the influence of the representation on the predictions. We compared the IUPAC name, InChI code, Omega notation, and SMILES of the TAGs. Interestingly, with similar accuracies of 92% (GPT-J and Llama), we see excellent performance, slightly higher than that obtained with “traditional” ML models (86–88%).

**Fig. 1:**
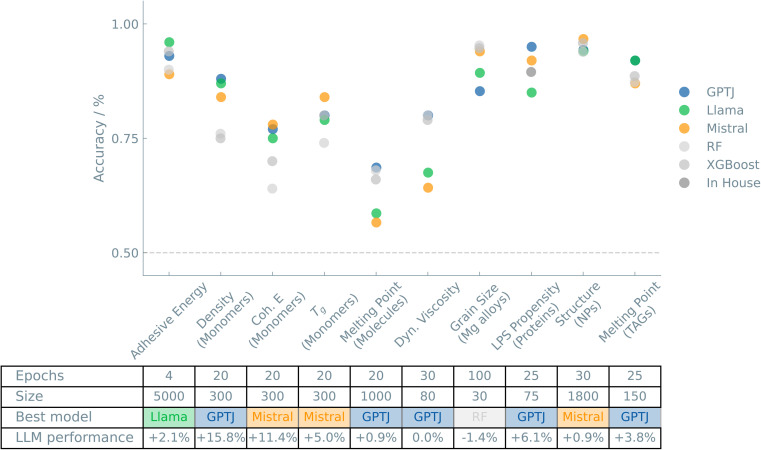
Overview of the “Materials and properties” case studies. The accuracies of binary classifiers are plotted for the “Materials and properties” case studies. Three different LLMs (GPT-J, Llama, and Mistral) and two traditional ML models (random forest (RF) and XGBoost) are compared. The dashed line indicates the zero-rule baseline of 50% accuracy. The table summarizes the number of epochs (for fine-tuning the LLMs), training set size, the best-performing model, and the relative difference between the best LLM and the best “traditional” model.

Fig. 1 shows an overview of the results of the case studies on Materials and properties.

### 3.2 Reactions and synthesis

Going beyond chemical properties, we explored the potential of our methodology in predicting reaction outcomes. Predicting reaction outcomes is a field in which conventional theory has made little progress. The complexity and specificity of chemical reactions make developing a general theoretical framework extremely challenging.^30^ Practically, indicating the success rate or yield could prioritize the synthesis of top candidates or omit protocols that lead to certain failures, thereby saving resources, time, and money.

#### 3.2.1 Activation energy of cycloadditions

A first example in this regard is based on a previous study that screened the influence of substituents of one of the reactants on the activation energy of bioorthogonal tetrazine-alkene cycloadditions. From a total of 966 different reactants, the free energy barrier was computed using density functional theory (DFT, see ESI Note 4.1†). We took the median free energy as the threshold for ‘good’ reactants for the specific reaction and the SMILES notation to represent the molecule in question. With a training set size of 500 examples, a fine-tuned GPT-J model reached an accuracy of 94%, significantly higher than random guessing, *i.e.*, 50% for a balanced dataset, and competitive with traditional ML approaches, *i.e.*, random forest (89%) and XGBoost (91%).

This case study is a great example of how machine learning, specifically LLMs, can significantly impact expensive computational studies. This model can be used as a first screening to filter out poor-performing structures. More expensive calculations can then be used for a more detailed analysis of well-performing structures.

#### 3.2.2 Free energy of catalyzed cleavage reaction

In the same category of organic reaction, a reaction dataset on a Ni-catalyzed aryl ether cleavage reaction was explored^31^ (see ESI Note 4.2†). Similarly, a large computational study was performed to gain knowledge about the efficiency of a set of catalysts. Our LLM-based approach has the potential to minimize expensive calculations and predict whether new molecules might be a suitable candidate for this reaction. As we know the chemical structure of the catalysts, we can base our predictions on the SMILES notation as inputs. For a median split base case, an accuracy of 88% was reached (GPT-J).

While excellent in performance, the scientific relevance of evaluating these systems based on a single threshold is limited. Rather, a small range of continuous descriptor values is often considered ‘good.’ All values above and below this range are then considered ‘poor.’ From the dataset of catalysts, only 3.8% was labeled a ‘good’ catalyst. As a result, we were forced to reduce our training set significantly to get a balanced dataset. Nevertheless, even with a training size of 100 data points, the model was able to classify 79% (GPT-J) of the test data correctly.

An interesting future strategy would be to use an LLM model combined with more expensive quantum calculations. Initially, one would aim for a band of ‘good’ structures that is broader than one would like from a catalysis point of view but more balanced to get a more accurate model. Then, if we get more ‘good’ materials, we will retrain the model with a narrower band.

#### 3.2.3 Yield of catalytic isomerization

A catalytic isomerization was examined in this case study (see ESI Note 4.3†). The dataset is part of a scoping study where the catalytic activity of PtBr_2_ was assessed. Apart from the starting material, all reaction conditions were kept constant. The amount of data is limited, with 16 experimental entries. In addition, the noise in the data is expected to be rather high as neither the starting material nor the product is very stable. We were interested in predicting the success of the isomerization based on the yield (>50%). Even after optimizing hyperparameters, no valuable models could be created. Despite the low predictive power of the models, this case study exemplifies an untouched application of LLMs in chemical research, namely scoping studies. Such studies are traditionally performed to gain a thorough understanding of whether a reaction is successful and how efficient the reaction is. While the amount of different substrates tested can be large, they often still screen a small fraction of the chemical space. Predicting the outcome of similar structures based on a set of experimentally assessed reactions could accelerate material discovery.

#### 3.2.4 Kinetics of polymerization

This case study focused on an experimental kinetic polymer screening (see ESI Note 4.4†). The dataset contained 23 entries with different monomer–monomer concentration combinations, each with their experimental measured kinetic profile. Our objective was to predict the polymerization rate from the reaction conditions, *i.e.*, we used the monomer representation and the monomer concentration as input variables. Given the smaller size of the dataset, hyperparameters were modified slightly to obtain acceptable performances. When comparing different representations of the monomer, we see that fine-tuned models based on the SMILES of the monomer (accuracy of 76% with GPT-J) do better than models based on the IUPAC name of the monomers (accuracy of 57% with GPT-J). Using the SMILES notation to represent the molecules, the Llama model provided a slightly higher accuracy (83%). We also noticed that reactions with a polymerization rate close to the binary split threshold, *i.e.*, the median of the dataset, remained difficult to predict.

Not only are high-throughput kinetic screenings an excellent way to gain in-depth insights into reaction mechanisms, but they also produce datasets that can be used to train ML models and guide further research and development. Here, only two reaction parameters were varied. Combining screenings in a multi-parameter landscape with predictive models could accelerate polymer synthesis optimizations.

#### 3.2.5 Photocatalytic water spliting activity of MOFs

Following with reactions, in this case, we explored a dataset containing 95 MOF structures with different properties related to photocatalytic water splitting, as obtained from DFT calculations (see ESI Note 4.5†). Such DFT calculations require significant computational resources.

In this study, we predicted various photocatalytic properties of MOFs, thereby assessing whether a given material has the right band alignments for water splitting and absorbs visible light. We used the elemental composition of the MOF's linker and metal node to represent the material. The fine-tuned LLMs could successfully predict the various properties with accuracies higher than 90%.

#### 3.2.6 Photocatalytic CO2 conversion activity of MOFs

We explored another example of the use of MOFs, in this case study for photocatalytic CO_2_ conversion (see ESI Note 4.6†). The dataset (*n* = 77) contained the catalyst system (metal source, linker, phase, sacrificial agent, and cocatalyst), the band gaps of the MOF and cocatalyst, and the photocatalytic activity studied. This data allowed us to investigate the use of different parameters in the prompt as predictors of the photocatalytic activity of MOFs. When we used the catalytic system, we obtained an accuracy of 65% (GPT-J). When we combined the SMILES notation and the catalytic system parameters in the prompt, for a total of six features as predictors, the accuracy increased to 68% (GPT-J). However, the predictions of this model were not better than random guessing for samples with high values of photocatalytic activity. We probably need more data to predict the outcome of complex processes such as photocatalysis. On the other hand, adding the values of band gaps, conducting band, and valence band to the prompt, *i.e.*, seven extra features, did not increase accuracy (58%, GPT-J).

#### 3.2.7 Success of MOF synthesis

In this case related to the synthesis of materials, an interesting dataset of MOFs was investigated (see ESI Note 4.7†). The objective here was to predict the success of the synthesis of a MOF given experimental parameters extracted from reaction protocols. Interestingly, the majority of reported protocols often lead to the desired product, hindering the creation of a balanced dataset and subsequently making unbiased predictions hard.

This quantity issue is reflected in the provided dataset, which has only 25 different reaction conditions. Taking a yield of 20% as the success threshold could create a fairly balanced dataset. After training for just ten epochs on a training set size of 20 examples, the fine-tuned models could not recognize the prompt/completion structure and thus failed to output a meaningful prediction. Using a training set size of 20 and increasing the number of epochs to 50 leads to the expected binary responses, *i.e.*, 0 or 1, with an average accuracy of 89% (GPT-J).

**Fig. 2:**
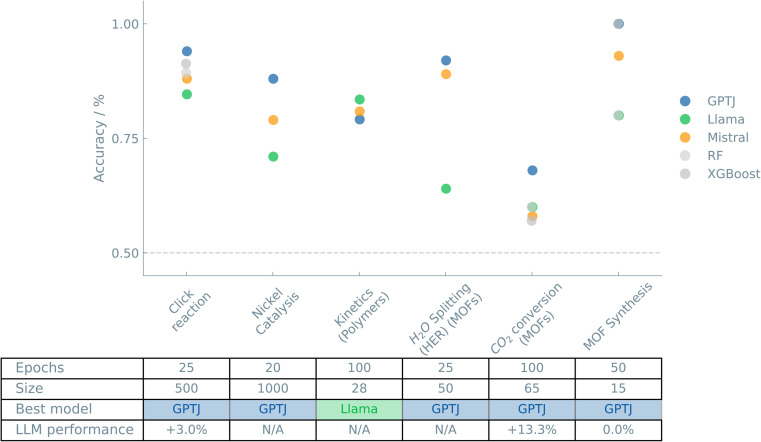
Overview of the “Reactions and synthesis” case studies. The accuracies of binary classifiers are plotted for the “Reactions and synthesis” case studies. Three different LLMs (GPT-J, Llama, and Mistral) and two traditional ML models (random forest (RF) and XGBoost) are compared. The dashed line indicates the zero-rule baseline of 50% accuracy. The table summarizes the number of epochs (for fine-tuning the LLMs), training set size, the best-performing model, and the relative difference between the best LLM and the best “traditional” model.

Fig. 2 shows an overview of the results of the case studies on Reactions and synthesis.

### 3.3 Systems and applications

Here, we explore the potential of LLMs to predict the outcomes of different systems and applications.^32^ Knowing the effect of processing parameters on process performance can help to optimize a system and increase its efficiency. Gaining knowledge of the influence of the experimental conditions on the results can allow us to tailor the process according to our specific objectives.

#### 3.3.1 Gas uptake and diffusion of MOFs

In this case, an extended version of the SMILES notation of MOFs was explored.^33^ Here, the MOFid, a combined string of the SMILES of the individual building blocks that construct a MOF, served as the chemical descriptor in predicting its gas uptake and diffusion (see ESI Note 5.1†).^34^ In an extensive computational study, Daglar and Keskin^33^ simulated hydrogen, nitrogen, helium, and methane gas uptake and diffusion in more than 5000 structures, which served as our dataset. These eight individual case studies yielded an average accuracy of 68% for predicting the different properties. Notably, these results are in line with the models in the original work.

Some MOFs (*e.g.*, ZIFs) have different isomers with the same chemical building blocks. Therefore, it is interesting to investigate whether adding further details on the structure in the prompt will improve learning. Apart from the MOFid and the uptake and diffusion values, Daglar and Keskin^33^ included 20 additional simulated features of the MOF structures, all of which are numeric and grouped based on their chemical and physical relevance. We used a combined feature vector (per group) to create a prompt for predicting the binary class, *i.e.*, above or below the median, for helium diffusion. In the first experiment, we included the largest cavity diameter, pore limiting diameter, and the pore size ratio, *i.e.*, group A. Secondly, a prompt with the density, pore volume, porosity, and surface area, *i.e.*, group B, was created. For these “single group” experiments, we obtained an accuracy of 68% and 62%, respectively, for groups A and B. Only when we combined groups A and B, thus creating a prompt with seven features, we obtained a performance improvement (73% accuracy). Adding eight extra features related to the elemental composition increased the accuracy to 77%. We tested various models for predicting helium diffusion and saw very comparable results among the three tested LLMs.

#### 3.3.2 Hydrogen storage capacity of metal hydrides

A potential replacement for fossil fuels is hydrogen. One of the disadvantages of hydrogen compared to fossil fuels is its low energy density. Finding ways to store hydrogen is, therefore, an important research theme. Metal hydrides are a promising class of materials for their capacity to store hydrogen.^35^

The heat of formation of a metal hydride is often used as an indicator for their potential hydrogen storage use. Theoretically, this value is related to the equilibrium pressure. We, therefore, started our experiment by validating if fine-tuned LLMs could capture this relation. The ML-HydPARK dataset created by Witman *et al.*^36^ contains 430 metal hydrides with their respective heat of formation and equilibrium pressure (see ESI Note 5.2†). In these initial experiments, we used the median heat of formation as the threshold for the binary classification. We fine-tuned an LLM that could answer the question “Is the heat of formation, and thus its potential for hydrogen storage, of a metal hydride with an equilibrium pressure of 〈value〉 high or low?”. Such models indeed predicted the heat of formation from a material's equilibrium pressure with an accuracy of 76% (GPT-J).

In an alternative approach, we hypothesized that the metal in the material is an indicator of success. We substituted the equilibrium pressure with the elemental formula of the material and repeated the training. Instead of a numeric feature, we now describe the material with a simple textual string, fully exploiting the potential of LLMs. These binary classification models performed significantly better, with an accuracy of 85% (GPT-J). A possible explanation for this increase in performance might be rooted in the augmented information present in the chemical composition of the material. When we combined both the pressure and the chemical formula in the feature vector, we saw a slightly higher accuracy than with the model trained on only pressure values (reaching an accuracy value of 86% with the Llama model), suggesting that the additional chemical information had extra predictive power.

From a practical point of view, a realistic threshold for defining promising materials would be useful. As suggested by the literature, this range (heat of formation values between −40 kJ mol^−1^ to −20 kJ mol^−1^) created a slightly unbalanced dataset. Nevertheless, acceptable performances were still achieved with accuracies of 75% (GPT-J).

#### 3.3.3 CO_2_ adsorption of biomass-derived adsorbents

In this example, we investigated a dataset on the synthesis of activated carbons from different biomass precursors for CO_2_ capture by adsorption processes^37^ (see ESI Note 5.3†). From these data, we analyzed two binary classification case studies to predict whether the BET surface area and CO_2_ adsorption capacity of biomass-based adsorbents are high or low.

The dataset contains data on 33 biomass precursors and ten activating agents. We fine-tuned LLMs to predict the BET surface area and CO_2_ adsorption capacity from the biomass precursor, activation conditions, adsorbent textural properties, and adsorption conditions. An interesting aspect of this example is that, unlike in conventional machine learning models that require conversion to numerical values, the biomass precursor's name and the activating agent's chemical formula were entered as textual strings into the model.

By taking the median as the threshold to classify adsorbent materials, an accuracy of 90% (Mistral, comparable to traditional ML models) was obtained for the prediction of the CO_2_ adsorption capacity from the precursor activation conditions, activated carbon textural properties, and adsorption conditions, with non-optimized hyperparameters. Since the dataset is smaller (training set of 65 data points), we had to increase the number of finetuning epochs from 30 to 140 to predict the BET surface area from the precursor activation conditions with an accuracy of 76% (Llama). We also found that models trained without the precursor name performed slightly worse than models trained on the full feature vector, indicating that the model could also learn some trends associated with the biomass name. For these tasks, LLMs performance is comparable to that of “traditional” ML models.

Under a more practical classification, we also evaluated a threshold value that would allow us to predict which materials are really ‘good,’ which forced us to reduce the training set to obtain a balanced dataset. Under these conditions, CO_2_ adsorption capacity was predicted with an accuracy of 82% (GPT-J) by increasing the number of fine-tuning epochs to 100. Likewise, using a smaller dataset, BET surface area was predicted with 75% (GPT-J) accuracy by increasing the number of fine-tuning epochs to 200.

#### 3.3.4 Thermal desalination of water

In this case study, we focused on the thermal desalination of saline or brackish water sources (see ESI Note 5.4†). Knowing the behavior of thermal desalination units is crucial to optimize their design.^38^ Here, we evaluated two case studies to predict the Gain Output Ratio (GOR), which is a measure of the thermal energy utilization efficiency, and the specific heat transfer surface, which will determine the size of the plant of a solar desalination system from the number of effects and the steam temperature.

The dataset was split to obtain a balanced binary classification problem. The relatively small size of the dataset forced us to use a maximum training set size of 25 example prompts. The first model with non-optimized hyperparameters showed no predictive power. By increasing the number of epochs from 30 to 100, we obtained an accuracy of 87% (Mistral) for the specific heat transfer surface and 100% for GOR (Mistral), which are values comparable to “traditional” ML models.

We also trained binary classification models using unbalanced datasets to simulate a more realistic case for finding top-performing conditions. The model did not perform better than the random guessing baseline of 80% to predict the specific heat transfer surface, given an accuracy of 80% (GPT-J), but showed acceptable performance in predicting GOR, with 93% accuracy (GPT-J).

#### 3.3.5 Detection response of gas sensors

Following with applications, in this example, we analyzed a dataset on the sensing behavior of gas sensors (see ESI Note 5.5†). Here, the objective was to predict the detection response of gas sensors by training a model using data from 56 different experimentally analyzed conditions, varying three design parameters: sensor type (*i.e.*, core–shell and composite sensors), zinc oxide concentration, and operating temperature.

A binary classification model trained on 45 example prompts (100 epochs) could predict whether a given sensor was in the top half performing conditions with 89% accuracy (GPT-J, obtaining similar results with other LLMs and “traditional” ML models).

#### 3.3.6 Stability of gas sensors

In the field of gas sensor applications, we also explored a dataset on the long-term stability of gas sensors (ESI Note 5.6†). In this case, the objective was to predict whether a SnO_2_-based gas sensor is stable or not as a function of the type of dopant material, its dosage, and the calcination temperature during synthesis. By accurately predicting stability, a more efficient search for ideal sensors could accelerate the field of gas sensing.

With a rather small dataset of 19 data points, this case study tested the limits on the size of the training set. With slightly optimized hyperparameters by increasing the number of epochs to 120, a predictive accuracy of 71% (GPT-J) was achieved for a binary classification model that was able to predict whether a sensor was stable, *i.e.*, had a response loss between days 5 and 15 of less than 12%.

#### 3.3.7 Gasification of biomass

A dataset on the biomass gasification process was also used to validate our predictive framework in applications,^39^ such as thermochemical conversion of biomass to produce energy carriers. The data described the H_2_/CO ratio in the syngas obtained from the gasification of solid biomasses (see ESI Note 5.7†). Here, we are interested in predicting whether the gasification process of a given biomass gives a H_2_/CO ratio higher than 1.8, meaning that it is suitable for fuel and chemical synthesis. Since data points with such values only represent 30% of the overall dataset, we were forced to reduce its size to obtain a balanced dataset. With a relatively small training set of 25 data points, we increased the number of epochs to 140 to obtain an accuracy of 70% (GTP-J), *i.e.*, higher than the random guess value of 50%.

**Fig. 3:**
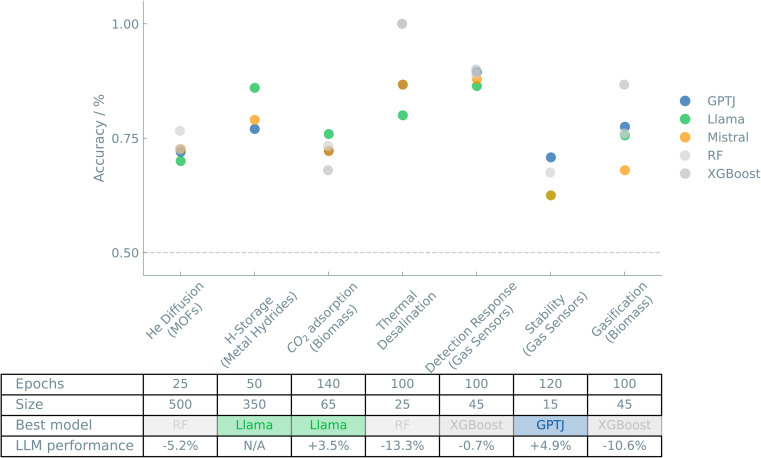
Overview of the “Systems and applications” case studies. The accuracies of binary classifiers are plotted for the “Systems and applications” case studies. Three different LLMs (GPT-J, Llama, and Mistral) and two traditional ML models (random forest (RF) and XGBoost) are compared. The dashed line indicates the zero-rule baseline of 50% accuracy. The table summarizes the number of epochs (for fine-tuning the LLMs), training set size, the best-performing model, and the relative difference between the best LLM and the best “traditional” model.

Fig. 3 shows an overview of the results of the case studies on Systems and applications.

## 4 Discussion

In chemistry, we mostly have to deal with a limited amount of data. Hence, it is essential to use leverage in any machine-learning approach applied to chemistry. In an LLM, one leverages the linguistic nuances, patterns, and knowledge captured in correlations harvested from large quantities of internet text. But it is not only knowledge, as is illustrated with the example with the hypothetical polymer notation; in this case the LLMs acts as a flexible probabilistic (n-gram-like) language model. This new source of data, combined with using natural language to interface with the model, makes this approach, potentially, more powerful than machine learning models trained only on conventional data sources.

In this work, we tried to obtain some insights into the performance of such LLMs by looking at 22 case studies describing many different systems, ranging from predicting simple thermodynamic properties to device performance. The obvious question is whether it works. In this section, we try to answer these questions in parts.

We must remember that the original corpus of text used to train these models was not specifically curated for chemical questions. It is remarkable that we can create specific solutions for a range of chemical subfields, spanning from a molecular level to reaction kinetics to high-end applications.

In most of these case studies, the LLMs demonstrated their ability to predict basic structure–property relationships. Various cases concerning reactions showcased that LLMs can predict reaction outcomes and yield determination, thereby facilitating reaction optimizations, scoping studies, or catalyst designs. In our applied chemistry cases, the versatility of our approach was further underscored by predicting system parameters, thereby assisting the optimization of real-life chemical processes.

Our results also make clear that the LLM approach works best with a reasonably balanced dataset. However, in practice, one is often interested in the (small) subset of top-performing materials, and we observe that the training set quickly becomes too unbalanced to make sufficiently accurate predictions. The solution to this problem is to start with a model trained on a less narrow window. We typically observe that depending on the size of the dataset, this approach is better than random guessing. So, for problems that are too complex or for which we do not have any intuition, we already gain. If we then collect more data in the region of interest, we can narrow the window, making the model increasingly useful.

Our study provided useful insights into more specific issues related to featurization, feature importance, size of the dataset, and model used.

### 4.1 Featurization

Can we translate the prediction of the properties of a material or a chemical reaction into a set of simple questions and answers that can be used to fine-tune an LLM? Conventional machine learning requires featurization, *i.e.*, converting the chemical system into a feature vector that quantifies the similarity between systems one wants to compare. Especially in experimental datasets, feature extraction is not as obvious as it might seem. Indeed, many tools and molecular fingerprints exist, but these often pose a burden for non-experts aiming to integrate machine learning into their workflows.

**Fig. 4:**
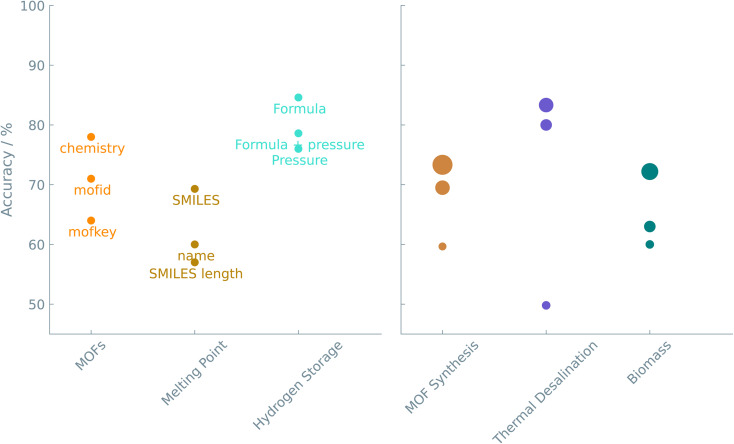
Accuracy of the different representations. The color indicates the particular case study. The same color coding is used in Fig. 5 to compare between all case studies. In the left figure, the annotations are the representations used in the prompt. In the right figure, the size of the circles is related to the number of epochs used. In these examples, the results shown were obtained from fine-tuning the GPT-J model.

As the case studies show, translating a chemical question into a prompt for fine-tuning is straightforward. The primary challenge is choosing how to represent a material or chemical. One can try one of a number of different representations or even use a combination of such representations (see Fig. 4). Standardized notations like SMILES can be exploited to represent chemical structures in LLMs. The readability of SMILES strings makes them convenient for researchers and chemical toolkits to interpret. We show that text-based descriptors like SMILES (see ‘Melting point of molecules’ study), MOFid (see ‘Gas uptake and diffusion of MOFs’ study), or even non-standardized strings (see ‘Adhesion energy of polymers’ and ‘Structure of nanoparticles’ studies) perform well in connecting structural information with physical/chemical properties or reaction outcomes. However, as Alampara *et al.*^40^ pointed out, adding structural information does not always give better results.

Machine learning approaches become even more powerful when dealing with multiple variables. Thus, we extended our prompts with additional data to allow for multi-variable predictions. For instance, in the ‘Hydrogen Storage Capacity of Metal Hydrides’ study, we combined molecular information and equilibrium pressure in one prompt to predict a material's heat of formation. Interestingly, we noticed that this longer prompt outperformed the model that was only trained on the pressure data. Similar trends are also seen in the ‘Gas Uptake and Diffusion of MOFs’ and ‘Photocatalytic Water Splitting Activity of MOFs’ studies. This methodology becomes particularly interesting for predicting the experimental success of a synthetic reaction. Reported reaction protocols are generally described as a combination of textual (*e.g.*, reagents, solvent system) and numeric (*e.g.*, reaction time, temperature) data. In the ‘CO_2_ Adsorption of Biomass-derived Adsorbents’ study, the dataset consisted of 8 variables used to fine-tune the models. Again, models trained without one of the textual variables performed slightly worse, highlighting the synergy between text-based data and LLMs.

### 4.2 Feature importance

Feature importance is often an interesting analysis of a trained ML model to know which features carry the most weight in the model. Synthetic chemists also do this daily by asking, for example, “Which parameter do I most likely need to vary to get the desired result?”. Combining natural language and machine learning, *i.e.*, using LLMs, could facilitate this process and create an understandable approach to optimizing chemical systems. By iteratively removing one particular feature in the multi-variable prompt and assessing the accuracy of the resulting fine-tuned model, the influence of the respective parameter on the final objective can be evaluated. We used this approach in the ‘Gas Uptake and Diffusion of MOFs’ dataset. We saw high accuracies when structures were represented as 20 individual features. By removing descriptors, we noticed a drop in accuracies, hinting at the importance of the omitted descriptors on the final predictions.

### 4.3 Size of the dataset

**Fig. 5:**
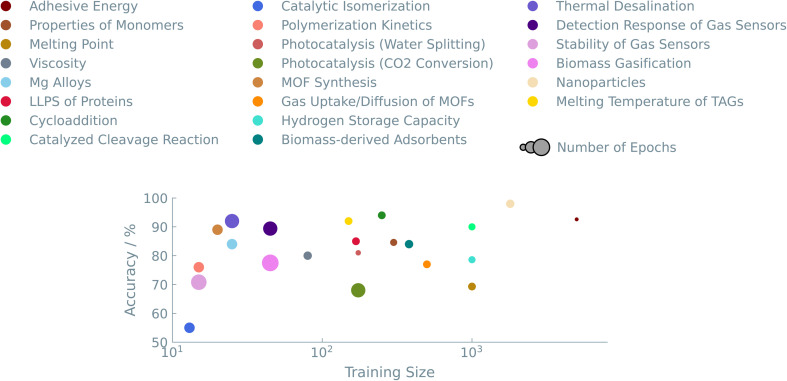
Accuracy as a function of the size of the dataset. The color indicates the case study, and the size of the circles is related to the number of epochs used. The represented results were obtained from fine-tuning the GPT-J model.

Data quantity, as well as quality, plays an important role in fine-tuning LLMs. We explored datasets ranging from as few as 20 to as many as 5000 entries. Fig. 5 summarises the accuracies we obtained for the different datasets. We see that our approach works well for datasets in the low-data regime. A consistent trend over all experiments suggests that models trained on larger datasets have excellent predictive performance. We typically get accuracies above 80%, allowing us to make balanced training sets if one wants to identify a (much smaller) sub-set of interesting materials.

On the other end of the plot, *i.e.*, really small datasets (<20 data points), the LLMs initially have difficulties predicting any meaningful output. For experiments with small datasets, we slightly optimized the hyperparameters to increase the performance of the models. By increasing the number of times that the models see the training data, *i.e.*, the number of epochs during training, the performance of these models came close to large dataset models in terms of accuracy (see Fig. 4 (right)).

### 4.4 Model selection

**Fig. 6:**
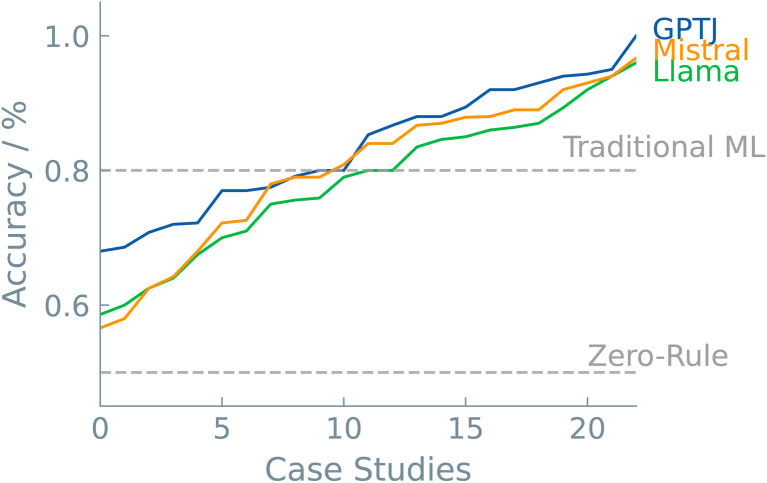
LLM comparison over case studies. The accuracies of all the studied case studies are plotted in increasing order for all three LLM models. Dashed lines show the zero-rule baseline of 50% and the average accuracy of the “traditional” ML models, *i.e.*, 80%.

A last discussion point is dedicated to model selection. As with all machine learning applications, the particular model can significantly impact the predictive outcome. This is also the case in LLMs. The pool of LLMs is increasing at a fast pace. The community is growing, and prominent players in the AI landscape are now creating their own base models. Each of these models is trained on a different corpus of text, each with different parameters. In this work, we selected three models to fine-tune and compare their predictive performance from chemical datasets. In addition, the results were benchmarked against two commonly used “traditional” ML models, *i.e.*, random forest and XGBoost. In Fig. 6, the case studies are plotted with increasing accuracy for each model. Interestingly, there were no significant differences among the different LLMs. We see that, in general, LLMs can compete with traditional ML models. In the majority of the case studies (>80%), the best LLM outperformed traditional ML models. The different types of case studies made it clear that superior non-LLMs are often found in ‘Systems and Applications’ datasets (Fig. 3). One possible explanation could lie in the greater specificity of the problem available data during pre-training of the LLMs. The higher performance of LLMs fine-tuned on SMILES notations, a more prevalent and intuitive representation, supports this hypothesis.

## 5 Conclusions

For most, if not all, of these 22 case studies, the LLMs performed (much) better than random guessing and generally better than “traditional” ML models. This is a remarkable result, given that the LLMs were not pre-trained on the bulk of scientific literature. In addition, our effort in making a predictive model using an LLM is modest.

We focused on binary classifications that provide a simple ‘yes’ or ‘no’ answer. We see such tasks as a first step; if the LLM did not outperform random guessing, we would conclude that there has been no learning. If we can sufficiently accurately predict such a simple classification, one can proceed to develop a regression model as the next step. Yet, even binary classifications can be useful, especially in experimental settings where a continuous value is often unnecessary to streamline decision-making. An accurate binary classifier can already facilitate various aspects of today's research. For example, ML-based screenings of a particular chemical system can significantly reduce computational resources or experimental work, *e.g.*, “Is it worth doing this experiment?”.

Even a modest accuracy can be helpful if the alternative is random guessing or complex field-specific ML models. Moreover, we also show that these models improve significantly if more data is collected. In this context, we must mention the importance of balanced datasets. In most practical cases, there are many more failures than successful experiments. Hence, in our training, we had to reduce the training set to have a reasonably balanced dataset. If we were to use literature data, we would have the opposite problem. In most, if not all, studies, only successful results are published. Machine learning, like human learning, learns even more from its failures.^41^ Thus, if we want to take full advantage of the tools explored here, we need to rethink how data are reported.^42^

In addition to the remarkable performances of the trained models, we also want to stress that natural language in ML models facilitates various aspects of the case studies. By obviating the need to featurize the chemical system, this use of textual descriptors of molecules points to an attractive alternative interface to chemical knowledge suitable for non-experts. Moreover, we noticed that natural language greatly improves scientific interpretation, effective discussions, and communication between different research fields.

## Data availability

The datasets and Jupyter Notebooks used in this work are available at https://github.com/JorenBE/GPT-Challenge.

## Author contributions

The author contributions are provided in the ESI.†

## Conflicts of interest

K. M. J. and A. S. A. have been contractors for OpenAI (as part of the red teaming network). K. L. S. is a consultant for Transition Bio.

## Supplementary Material

SC-OLF-D4SC04401K-s001
