# Supplementary material for: Assessment of fine-tuned large language models for real-world chemistry and material science applications
Source: Chem Sci. 2024 Nov 22;16(2):670–84. doi: 10.1039/d4sc04401k (PMC11629507; doi:10.1039/d4sc04401k)
Supplement: SC-016-D4SC04401K-s001 [file SC-016-D4SC04401K-s001.pdf]

Supplementary Information (SI) for Chemical Science.
This journal is © The Royal Society of Chemistry 2024

# Supporting Information: Assessment of Fine-Tuned Large Language Models for Real-World Chemistry and Material Science Applications

Joren Van Herck [1,**], María Victoria Gil [1,2,**], Kevin Maik Jablonka [1, 3, 4,**], Alex Abrudan [5], Andy S. Anker [6,7], Mehrdad Asgari [8], Ben Blaiszik [9,10], Antonio Buffo [11], Leander Choudhury [12], Clemence Corminboeuf [13], Hilal Daglar [14], Amir Mohammad Elahi [1], Ian T. Foster [9,10], Susana Garcia [15], Matthew Garvin [15], Guillaume Godin [16], Lydia L. Good [5,17], Jianan Gu [18], Noémie Xiao Hu [1], Xin Jin [1], Tanja Junkers [19], Seda Keskin [14], Tuomas P.J. Knowles [5,20], Ruben Laplaza [13], Michele Lessona [11], Sauradeep Majumdar [1], Hossein Mashhadimoslem [21], Ruaraidh D. McIntosh [22], Seyed Mohamad Moosavi [23], Beatriz Mouriño [1], Francesca Nerli [24], Covadonga Pevida [2], Neda Poudineh [15], Mahyar Rajabi-Kochi [23], Kadi L. Saar [5], Fahimeh Hooriabad Saboor [25], Morteza Sagharichiha[26], KJ Schmidt [9], Jiale Shi [27,28], Elena Simone [11], Dennis Svatunek [29], Marco Taddei [24], Igor Tetko [16, 30], Domonkos Tolnai [18], Sahar Vahdatifar [26], Jonathan Whitmer [28,31], D.C. Florian Wieland [18], Regine Willumeit-Römer [18], Andreas Züttel [32], and Berend Smit [1,*]

[1] Laboratory of molecular simulation (LSMO), Institut des Sciences et Ingénierie Chimiques, École Polytechnique Fédérale de Lausanne (EPFL), Rue de l'Industrie 17, CH-1951 Sion, Switzerland
[2] Instituto de Ciencia y Tecnología del Carbono (INCAR), CSIC, Francisco Pintado Fe 26, 33011 Oviedo, Spain
[3] Laboratory of Organic and Macromolecular Chemistry (IOMC), Friedrich Schiller University Jena, Humboldtstrasse 10, 07743 Jena, Germany
[4] Helmholtz Institute for Polymers in Energy Applications Jena (HIPOLE Jena), Lessingstrasse 12-14, 07743 Jena, Germany
[5] Yusuf Hamied Department of Chemistry, University of Cambridge, Cambridge CB2 1EW, United Kingdom
[6] Department of Energy Conversion and Storage, Technical University of Denmark, DK-2800 Kgs. Lyngby, Denmark
[7] Department of Chemistry, University of Oxford, Oxford OX1 3TA, United Kingdom
[8] Department of Chemical Engineering & Biotechnology, University of Cambridge, Philippa Fawcett Drive, Cambridge CB3 0AS, United Kingdom
[9] Department of Computer Science, University of Chicago, Chicago, IL 60637, United

States
[10]Data Science and Learning Division, Argonne National Laboratory, Lemont, IL 60439, United States
[11]Department of Applied Science and Technology (DISAT) , Politecnico di Torino, 10129 Turino, Italy
[12]Laboratory of Catalysis and Organic Synthesis (LCSO), Institute of Chemical Sciences and Engineering (ISIC), École Polytechnique Fédérale de Lausanne (EPFL), CH-1015 Lausanne, Switzerland
[13]Laboratory for Computational Molecular Design (LCMD), Institute of Chemical Sciences and Engineering (ISIC), École Polytechnique Fédérale de Lausanne (EPFL), CH-1015 Lausanne, Switzerland
[14]Department of Chemical and Biological Engineering, Koç University, Rumelifeneri Yolu, Sariyer, 34450 Istanbul, Turkey
[15]The Research Centre for Carbon Solutions (RCCS), School of Engineering and Physical Sciences, Heriot-Watt University, Edinburgh, EH14 4AS, United Kingdom
[16]BIGCHEM GmbH, Valerystraße 49, 85716 Unterschleißheim, Germany
[17]Laboratory of Chemical Physics, National Institute of Diabetes and Digestive and Kidney Diseases, National Institutes of Health, Bethesda, Maryland 20892, United States
[18]Institute of Metallic Biomaterials, Helmholtz Zentrum Hereon, Geesthacht, Germany
[19]Polymer Reaction Design group, School of Chemistry, Monash University, Clayton VIC 3800, Australia
[20]Cavendish Laboratory, Department of Physics, University of Cambridge, Cambridge CB3 0HE, United Kingdom
[21]Department of Chemical Engineering, University of Waterloo, Waterloo, N2L3G1, Canada
[22]Institute of Chemical Sciences, School of Engineering and Physical Sciences, Heriot-Watt University, Edinburgh, EH14 4AS, United Kingdom
[23]Chemical Engineering & Applied Chemistry, University of Toronto, Toronto, Ontario M5S 3E5, Canada
[24]Dipartimento di Chimica e Chimica Industriale, Unitá di Ricerca INSTM, Universitá di Pisa, Via Giuseppe Moruzzi 13, 56124 Pisa, Italy
[25]Chemical Engineering Department, University of Mohaghegh Ardabili, P.O. Box 179, Ardabil, Iran
[26]Department of Chemical Engineering, College of Engineering, University of Tehran, Tehran, Iran
[27]Department of Chemical Engineering, Massachusetts Institute of Technology, Cambridge, MA 02139, United States
[28]Department of Chemical and Biomolecular Engineering, University of Notre Dame, Notre Dame, Indiana 46556, United States.

[29]Institute of Applied Synthetic Chemistry, TU Wien, Getreidemarkt 9, 1060, Vienna, Austria
[30]Institute of Structural Biology, Molecular Targets and Therapeutics Center, Helmholtz Munich - Deutsches Forschungszentrum für Gesundheit und Umwelt (GmbH), Ingolstädter Landstraße 1, 85764 Neuherberg, Germany
[31]Department of Chemistry and Biochemistry, University of Notre Dame, Notre Dame, Indiana 46556, United States.
[32]Laboratory of Materials for Renewable Energy (LMER), Institut des Sciences et Ingénierie Chimiques, École Polytechnique Fédérale de Lausanne (EPFL), Rue de l'Industrie 17, CH-1951 Sion, Switzerland
[**]These authors contributed equally
[*]Berend.Smit@epfl.ch

November 27, 2024

# Contents


**1 Model Selection** **7**

**2 Methods** **8**

**3 Materials and Properties** **9**

3.1 Adhesive Energy of Polymers . . . 9
3.1.1 Scientific Background . . . 9
3.1.2 Dataset . . . 10
3.1.3 LLM Results . . . 10
3.2 Properties of Monomers . . . 15
3.2.1 Scientific Background . . . 15
3.2.2 Dataset . . . 15
3.2.3 LLM Results . . . 17
3.3 Melting Point of Molecules . . . 21
3.3.1 Scientific Background . . . 21
3.3.2 Dataset . . . 21
3.3.3 LLM Results . . . 21
3.4 Dynamic Viscosity of Molecules . . . 28
3.4.1 Scientific Background . . . 28
3.4.2 Dataset . . . 28
3.4.3 LLM results . . . 29
3.5 Microstructural Properties of Magnesium Alloys . . . 36
3.5.1 Scientific Background . . . 36
3.5.2 Dataset . . . 36
3.5.3 LLM results . . . 38
3.6 Phase Separation Propensity of Proteins . . . 42
3.6.1 Scientific Background . . . 42
3.6.2 Dataset . . . 42
3.6.3 LLM results . . . 44
3.7 Structure of Nanoparticles . . . 50
3.7.1 Scientific Background . . . 50
3.7.2 Dataset . . . 50
3.7.3 LLM results . . . 51
3.8 Melting Temperature of triacylglycerols . . . 72
3.8.1 Scientific Background . . . 72
3.8.2 Dataset . . . 72
3.8.3 LLM Results . . . 73

**4 Reactions and Synthesis** **77**
4.1 Activation Energy of Cycloadditions . . . 77
4.1.1 Scientific Background . . . 77
4.1.2 Dataset . . . 78
4.1.3 LLM results . . . 78
4.2 Free Energy of Catalyzed Cleavage Reaction . . . 82
4.2.1 Scientific Background . . . 82
4.2.2 Dataset . . . 82
4.2.3 LLM results . . . 84
4.3 Yield of Catalytic Isomerization . . . 88
4.3.1 Scientific Background . . . 88
4.3.2 Dataset . . . 88
4.3.3 LLM results . . . 89
4.4 Kinetics of Polymerization . . . 92
4.4.1 Scientific Background . . . 92
4.4.2 Dataset . . . 93
4.4.3 LLM results . . . 94
4.5 Photocatalytic Water Splitting Activity of MOFs . . . 99
4.5.1 Scientific Background . . . 99
4.5.2 Dataset . . . 100
4.5.3 LLM results . . . 101
4.6 Photocatalytic Carbondioxide Conversion Activity of MOFs . . . 104
4.6.1 Scientific Background . . . 104
4.6.2 Dataset . . . 104
4.6.3 LLM results . . . 105
4.7 Success of MOF Synthesis . . . 117
4.7.1 Scientific Background . . . 117
4.7.2 Dataset . . . 117
4.7.3 LLM results . . . 118

**5 Systems and Applications** **123**
5.1 Gas Uptake and Diffusion of MOFs . . . 123
5.1.1 Scientific Background . . . 123
5.1.2 Dataset . . . 124
5.1.3 LLM results . . . 124
5.2 Hydrogen Storage Capacity of Metal Hydrides . . . 131
5.2.1 Scientific Background . . . 131
5.2.2 Dataset . . . 132
5.2.3 LLM results . . . 132
5.3 Carbondioxide Adsorption of Biomass-derived Adsorbents . . . 137
5.3.1 Scientific Background . . . 137

5.3.2 Dataset . . . 138
5.3.3 LLM results . . . 139
5.4 Thermal Desalination of Water . . . 159
5.4.1 Scientific Background . . . 159
5.4.2 Dataset . . . 160
5.4.3 LLM results . . . 161
5.5 Detection Response of Gas Sensors . . . 173
5.5.1 Scientific Background . . . 173
5.5.2 Dataset . . . 174
5.5.3 LLM results . . . 175
5.6 Stability of Gas Sensors . . . 182
5.6.1 Scientific Background . . . 182
5.6.2 Dataset . . . 183
5.6.3 LLM results . . . 184
5.7 Gasification of Biomass . . . 190
5.7.1 Scientific Background . . . 190
5.7.2 Dataset . . . 191
5.7.3 LLM results . . . 191

**Author contributions** **200**

**References** **201**

# 1 Model Selection

For this study, we performed fine-tuning experiments using three different large language models (LLMs): GPT-J-6B, Llama-3.1-8B-Instruct, and Mistral-7B-Instruct.

Firstly, we used the open-source GPT-J 6B parameter model,[1,2] which was trained on the Pile, a large-scale curated dataset created by EleutherAI.[3] From pretests, we know that the results generally match the ones we find using GPT-3 models available via the OpenAI API. However, the GPT-J models tend to be more sensitive to the tuning parameters.

In addition, we benchmarked a model from the Llama family, provided by Meta.[4] We did initial comparison experiments between the Llama-3.1-8B model and the Llama-3.1-8B-Instruct model to explore the opportunities of the Instruct model for our task. An Instruct model is developed on a pre-trained model to improve the practical use of the LLM, e.g., to follow instructions and question-answering. Indeed, from eight preliminary tests, we do see that, on average, the Llama-3.1-8B-Instruct model performs 6% better (Table 1). We, therefore, went forward with the LLama-3.1-8B-Instruct model for more in-depth experiments of all the case studies.

As a third model, we also performed our experiments with a Mistral model.[5] Similarly to the Llama model, we used the Instruct fine-tuned version Mistral-7B-Instruct.

In the text from now on, the models GPT-J-6B, Llama-3.1-8B-Instruct, and Mistral-7B-Instruct are referred to as GPT-J, Llama, and Mistral, respectively.

We benchmarked the fine-tuned LLMs against "traditional" ML models. Two commonly used models were selected for this purpose: random forest (RF) and XGBoost. The features of the provided datasets were converted into numeric values when required and used as input for these models. The original numerical values were used as is. Categorical values were converted using one-hot encoding. SMILES notations of molecules were converted into Morgan fingerprints.

**Table 1. Overview of accuracy (%) results of fine-tuning Llama-3.1-8B and Llama-3.1-8B-Instruct on different datasets.** For each dataset, all hyperparameters were kept constant for fair comparison (see specific dataset in SI for details on hyperparameters).

| Dataset | Llama-3.1-8B | Llama-3.1-8B-Instruct |
|---|---|---|
| Adhesive Free Energy | 0.80 | **0.96** |
| Density (monomers) | 0.84 | **0.85** |
| Cohesive Energy (monomers) | 0.80 | **0.96** |
| Squared radius of gyration (monomers) | 0.85 | **0.91** |
| Glass Transition Temperature (monomers) | 0.85 | **0.90** |
| Melting Point | 0.55 | **0.59** |
| Grain Size (Mg alloys) | **0.92** | 0.89 |
| Dynamic Viscosity | **0.73** | 0.71 |
| Average | 0.79 | **0.85** |

## 2 Methods

We used 8-bit quantization[6] and 8-bit optimizers[7] in addition to the Low-Rank Adaptation of Large Language Models (LoRA) technique[8] (LoRA parameters: r=16, lora_alpha=32, and lora_dropout=0.05) to use the models on our hardware. We follow the same fine-tuning method as in our previous work.[9] The datasets and Jupyter Notebooks with the results can be found in `https://github.com/JorenBE/GPT-Challenge`. In these Notebooks, we used the fine-tuning framework of Jablonka et al.[9], using the `chemlift` package, which can be installed from `https://github.com/lamalab-org/chemlift`.

As a base case, we trained binary classification models using balanced datasets to predict whether the target value is above or below the median. To train these binary classification models, the dataset was split into two classes of equal size based on the target variable using the `qcut` function of the `pandas` Python library. Where the dataset size was sufficient, we set the test set constant to 50 while varying the size of the training set. Rather than seeking to optimize every individual case study, we looked for trends over the wide range of case studies. For the fine-tuning hyperparameters, we used 20 epochs and a learning rate of 0.0003 unless otherwise specified. The temperature was set to 0.0 for all experiments.

# 3 Materials and Properties

This section describes the case studies regarding the properties of materials.

## 3.1 Adhesive Energy of Polymers

The dataset was provided by: Jiale Shi and Jonathan Whitmer[1]

### 3.1.1 Scientific Background

The realm of polymers is a complex playing ground. Finding an optimal material requires a deep understanding of the building blocks, i.e., monomers, and rigorous investigations into the physical parameters. One such feature that needs to be properly tuned is the material's interactions with various surfaces. Its importance can be noticed in various aspects of everyday life; think of the paintings on the wall or the coatings on our cars. Various forces and phenomena drive all these interactions and profoundly affect how the polymeric material adheres to or is repelled by a surface (Figure 2).

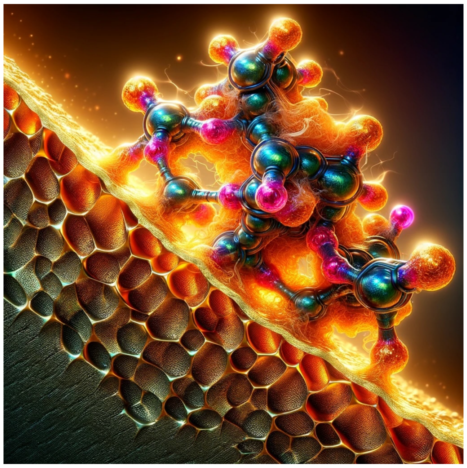

**Figure 1.** AI generated representation of a polymer chain adhering to a surface.

Understanding these interactions is thus crucial for tailoring material properties and improving product performance for specific applications. Multiple computational studies suggest a strong correlation between the polymer sequence and the surface adhesion. Also, various machine learning methods have been utilized to predict polymer properties based on the polymer structure. Shi et al.[10] combined both methodologies to quantitatively study the effect of the polymer sequence on the adhesive free-energy with a surface. Using molecular dynamics simulations, the authors created an extensive database of 20,000 AB copolymers, unique in their sequence and respective adhesion properties. With support vector regression models, they successfully predicted the adhesion free-energy from the sequence information of the copolymer. Our GPT approach focused on the classification of this adhesive free-energy.

[1] Department of Chemical and Biomolecular Engineering, University of Notre Dame, Notre Dame, Indiana 46556, United States.

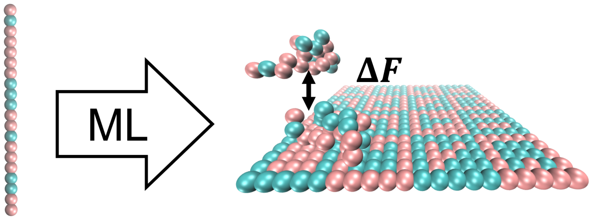


**Figure 2. Illustration of the adhesive free-energy $\Delta F$.** Different types of beads created unique copolymer sequences. MD simulations were used to compute the adhesive free-energy $\Delta F$ of the sequences.

### 3.1.2 Dataset

The dataset contains copolymer sequences. The polymer chains are sequences of 20 monomers of type A or type B (e.g., for ABBABAABBBABABBABBBA). These polymer chains are modeled as flexible 20-bead linear chains, with $20^2$ unique structures. A square lattice of 20 beads was created to study the polymer-surface interaction.

The adhesive free-energy $\Delta F$ was calculated using molecular dynamics simulations for the polymer chains interacting with the surface (see Shi et al.[10] for details on the simulations). We obtained 16,000 entries for initial testing, further referred to as Dataset I (see the distribution of the adhesive free-energy values in Figure 3). An unseen subset of 4,000 polymer sequences (Dataset II) was later used as a hold-out dataset to validate the performance of previously trained models.

We used a simple prompt template shown in Table 2 for experiments to predict adhesive free-energy.

**Table 2. Example prompts and completions for predicting the adhesive free-energy $\Delta F$ of block copolymers.** `<copolymer>` is the placeholder for the sequence of the polymer chain.

| prompt | completion | experimental |
|---|---|---|
| Example of training data | | |
| `What is the ΔF of <copolymer>?` | 0 | Low |
| `What is the ΔF of <copolymer>?` | 1 | High |

### 3.1.3 LLM Results

**Base Case** For the binary classification models, we split the dataset I into two equally sized classes based on their adhesive free-energy $\Delta F$ values as an initial test. Entries with values higher than the median of 8.20 $k_B$T are labeled “1”, and entries with lower values are labeled

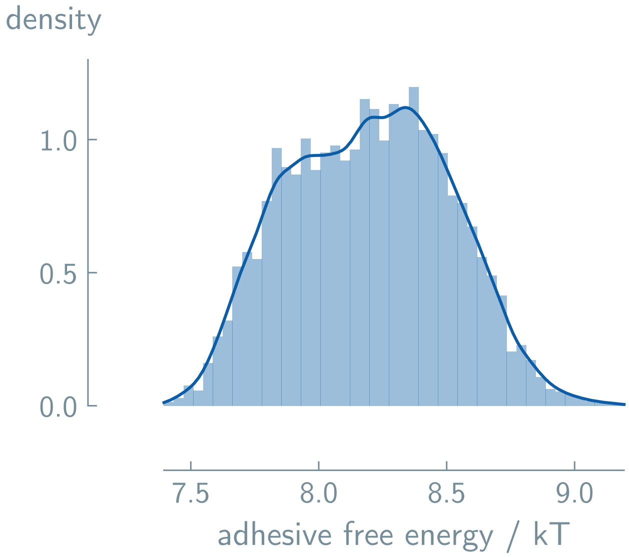


**Figure 3. Distribution of adhesive free-energies in the dataset.** The median adhesive free-energy is 8.20 $k_B$T.

"0". We performed a learning curve analysis on this binary classification. In contrast to the original work, where the sequence was converted to numeric values, we trained the models of the original representation of "A" and "B" beads. Dataset I was relatively large in numbers, which made it possible to train the model on 5,000 entries. The number of test data remained constant over all runs, i.e., 50. The number of epochs was set to 4 since we have a large dataset. Three unique runs were performed for every pair of training size/epoch experiments to get the average metrics. As the dataset is balanced, we can assume an accuracy of 50% as the zero-rule baseline, i.e., random guessing.

Three LLMs, i.e., GPT-J, Llama, and Mistral, were fine-tuned. We notice that all three models are comparable in performance. A maximum accuracy of 96% was reached with the Llama model (Figure 4 and Table 3). In addition, the fine-tuned LLMs were compared with "traditional" ML models (XGBoost and random forest (RF)). We can conclude that LLMs can in general compete with these models.

We tested the GPT-J model (training set size of 5,000 and 4 epochs) on a holdout dataset (Dataset II) to eliminate the possibility of data leakage. Our models never saw these 4,000 sequences. Figure 5 shows the confusion matrix of the experiment. The performance on the predictions of these adhesive free energies was similar to the initial test data, i.e., accuracy of 88.1%.

**Table 3. Overview of results of LLMs and "traditional" ML predicting the binary class of the adhesive free-energy.** Three runs were performed to get the metrics average. All LLMs were fine-tuned with 4 epochs and a learning rate of 0.003. For the random forest (RF) and XGBoost models, `optuna` was used for hyperparameter optimization. Maximum performances are highlighted in bold.

| Size | Model | Accuracy | F1 Macro | F1 Micro | Kappa |
|---|---|---|---|---|---|
| 500 | **GPT-J (LLM)** | **0.88** | **0.88** | **0.88** | **0.76** |
| | Llama (LLM) | 0.86 | 0.86 | 0.86 | 0.72 |
| | Mistral (LLM) | 0.84 | 0.85 | 0.85 | 0.69 |
| | RF | 0.81 | 0.81 | 0.81 | 0.62 |
| | XGBoost | 0.85 | 0.85 | 0.85 | 0.69 |
| | Zero-rule | 0.50 | 0.50 | 0.50 | 0.00 |
| 5000 | GPT-J (LLM) | 0.93 | 0.93 | 0.93 | 0.85 |
| | **Llama (LLM)** | **0.96** | **0.96** | **0.96** | **0.92** |
| | Mistral (LLM) | 0.89 | 0.89 | 0.89 | 0.79 |
| | RF | 0.90 | 0.90 | 0.90 | 0.80 |
| | XGBoost | 0.94 | 0.94 | 0.94 | 0.87 |
| | Zero-rule | 0.50 | 0.50 | 0.50 | 0.00 |

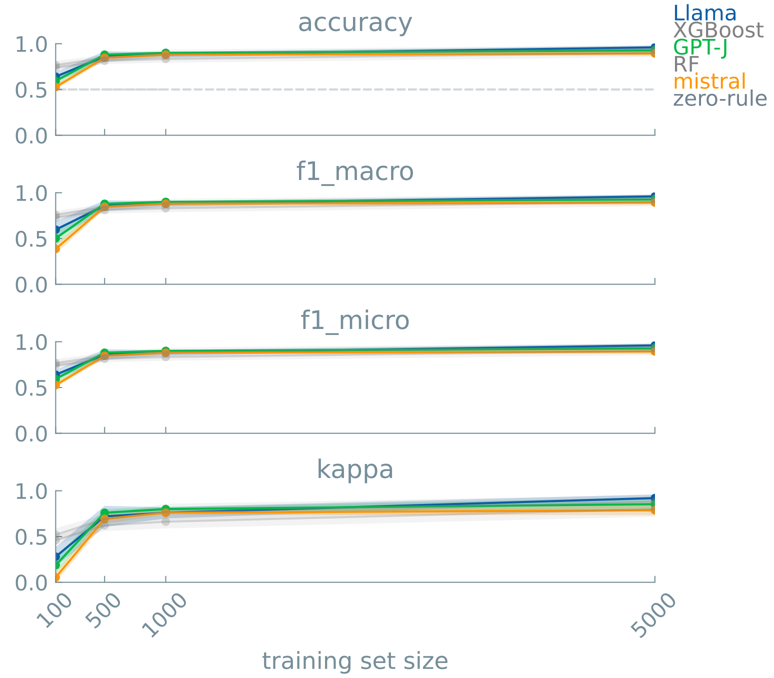


**Figure 4. Learning curve analyses of binary classifications of the adhesive free-energy using different models.** Three LLMs (GPT-J, Llama, and Mistral) and two "traditional" models (XGBoost and random forest (RF) were trained to predict the binary class of the adhesive free-energy of polymers. We used 50% as a random guess accuracy (dashed line), representing the zero rule baseline, i.e., a model that always predicts the most common class. Three runs were performed for each model to get the metric's average and standard deviation. Subsets of Dataset I were used to train and test the models, with only the sequence of the polymer chain as input. The fine-tuned Llama model reached the maximum accuracy of 96% (training set size of 5,000 and 4 epochs).

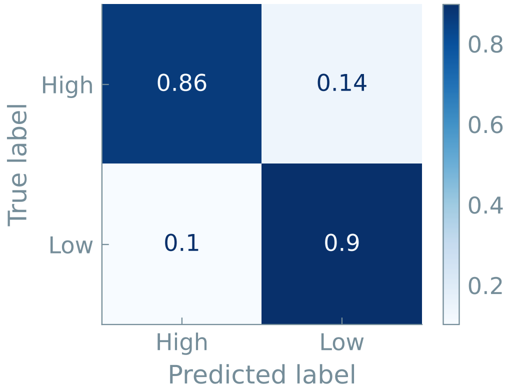


**Figure 5. Normalized confusion matrix on the hold-out test data.** The GPT-J model was trained on 5,000 training examples from Dataset I. Polymer sequences of the complete Dataset II ($n = 4,000$) were used to validate the model. An overall accuracy of 88.1% was reached.

### 3.2 Properties of Monomers

The dataset was provided by: KJ Schmidt, Ben Blaiszik and Ian T. Foster[2]

#### 3.2.1 Scientific Background

A challenge in designing polymers is that there are a near-infinite number of monomers to choose from. One can expect that some of these polymers' key performance indicators are correlated with their monomers' properties. However, to discover these relations, one needs a database with a diverse set of different properties of potential monomers.

The first steps towards generating such a database for monomers that can be used to synthesize polymers of the (meth)acrylate and (meth)acrylamide families was reported by Schneider et al.[11]. They used molecular dynamics simulations to predict a range of properties of these monomers. In particular, they focused on cohesive energy density, glass transition temperature, squared radius of gyration, and density (see Schneider et al.[11] for details).

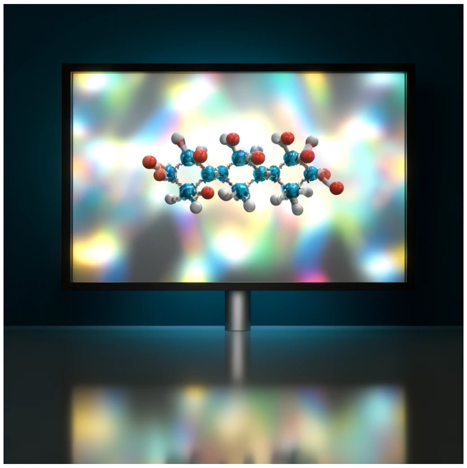

**Figure 6.** AI generated representation of designing polymers.

In addition, Schneider et al.[11] used these simulation results to develop an active learning approach to generate data more efficiently for new monomers. We are interested in developing a model that could predict these properties of a given monomer. In our approach, the monomers will be represented in the Simplified Molecular Input Line-Entry System (SMILES) notation. This textual string captures the atomic composition, bonds, branches, aromaticity, and stereochemistry of the chemical. It serves as an ideal test case to search for the limits of Large Language Models (LLMs).

#### 3.2.2 Dataset

The dataset generated by Schneider et al.[11] has 410 small molecules of the methacrylate and methacrylamide families, of which the molecular properties were calculated using molecular dynamics simulations. From the eight properties that were computed by Schneider et al., four were selected to validate our LLM approach: glass transition temperature, cohesive energy, squared radius of gyration, and density (see the original publication[11] for details on these simulations). The distribution of these data is shown in Figure 7.

[2]Department of Computer Science, University of Chicago, Chicago, IL 60637, United States

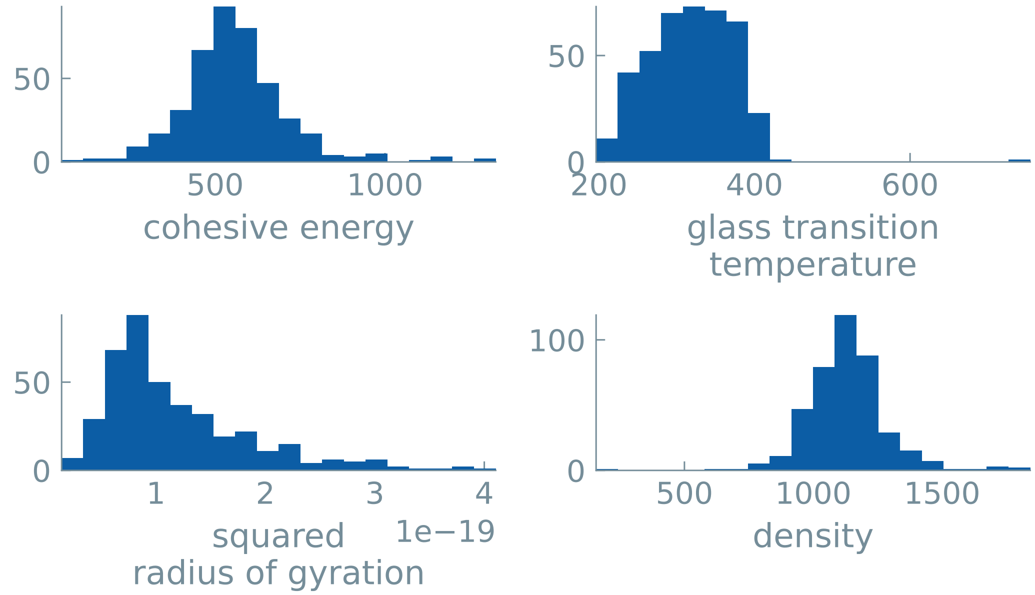


**Figure 7. Distribution of the four properties in the dataset used in this work.** The median of cohesive energy, glass transition temperature, squared radius of gyration, and density is 550 MPa, 319 K, $9.7 \times 10^{-20}$ Å, and 1132 $\mathrm{kg\,m^{-3}}$, respectively.

For our simple binary classification models, every property was split into two equally sized bins. Here, the threshold was the median of the different properties, i.e., 550 MPa, 319 K, $9.7 \times 10^{-20}$ Å, and 1132 $\mathrm{kg\,m^{-3}}$ for the cohesive energy, glass transition temperature, squared radius of gyration, and density, respectively.

Table 4 shows the prompt template we used to fine-tune our models.

**Table 4. Example prompts and completions for predicting the properties of monomers.** `property>` serves as a placeholder for glass transition temperature, cohesive energy, squared radius of gyration, or density. `<SMILES>` represents the SMILES notation of the monomer.

| prompt | completion | experimental |
|---|---|---|
| Example of training data | | |
| `What is the <property> of <SMILES>?` | 0 | Low |
| `What is the <property> of <SMILES>?` | 1 | High |

### 3.2.3 LLM Results

**Base case** As initial tests, we created separate binary classifications for every property of interest. We split the dataset into two equally sized classes based on the median of the respective property: class '0,' if lower than the median, and class '1,' if higher than the median. We performed learning curve analyses for all four properties. We used the SMILES notation as the representation of the molecules and trained separate models to predict this binary class of a single monomer property. As the total dataset contained 410 entries, our maximum number of training data was set to 300. The number of test data remained constant over all runs, i.e., 50. The number of epochs was set to 20 for all experiments. For every pair of training size experiments, three unique runs were performed to get the average metrics (Figures 8a, 8b, 9a and 9b).

**Table 5. Overview of results of LLMs and "traditional" ML predicting the binary class of the density of monomers.** Three runs were performed to get the metrics average. All LLMs were fine-tuned with 20 epochs and a learning rate of 0.003. For the random forest (RF) and XGBoost models, optuna was used for hyperparameter optimization. Maximum performances are highlighted in bold.

| Size | Model | Accuracy | F1 Macro | F1 Micro | Kappa |
|---|---|---|---|---|---|
| 300 | **GPT-J (LLM)** | **0.88** | **0.88** | **0.88** | **0.76** |
| | Llama (LLM) | 0.87 | 0.87 | 0.87 | 0.75 |
| | Mistral (LLM) | 0.84 | 0.84 | 0.85 | 0.69 |
| | RF | 0.76 | 0.76 | 0.76 | 0.52 |
| | XGBoost | 0.75 | 0.75 | 0.75 | 0.51 |
| | Zero-rule | 0.50 | 0.50 | 0.50 | 0.00 |

Three LLMs, i.e., GPT-J, Llama, and Mistral, were fine-tuned. We notice that all three models are comparable in performance. Maximum accuracies of 88% and 91% were reached with the GPT-J model for the prediction of the density and the squared radius of gyration, respectively (Table 5 and Table 7). Maximum accuracies of 78% and 84% were reached with the Mistral model for the prediction of the cohesive energy and the glass transition temperature, respectively (Table 6 and Table 8). Still, all LLMs gave similar performances, with no large differences. In addition, the fine-tuned LLMs were compared with "traditional" ML models (XGBoost and random forest (RF)). We can conclude that LLMs can in general compete with these models, as almost all LLMs performed better than these ML models.

**Table 6. Overview of results of LLMs and "traditional" ML predicting the binary class of the cohesive energy of monomers.** Three runs were performed to get the metrics average. All LLMs were fine-tuned with 20 epochs and a learning rate of 0.003. For the random forest (RF) and XGBoost models, `optuna` was used for hyperparameter optimization. Maximum performances are highlighted in bold.

| Size | Model | Accuracy | F1 Macro | F1 Micro | Kappa |
|---|---|---|---|---|---|
| 300 | GPT-J (LLM) | 0.77 | 0.77 | 0.77 | 0.54 |
| | Llama (LLM) | 0.75 | 0.75 | 0.75 | 0.50 |
| | **Mistral (LLM)** | **0.78** | **0.78** | **0.78** | **0.56** |
| | RF | 0.64 | 0.64 | 0.65 | 0.30 |
| | XGBoost | 0.70 | 0.70 | 0.70 | 0.40 |
| | Zero-rule | 0.50 | 0.50 | 0.50 | 0.00 |

**Table 7. Overview of results of LLMs and "traditional" ML predicting the binary class of the squared radius of gyration of monomers.** Three runs were performed to get the metrics average. All LLMs were fine-tuned with 20 epochs and a learning rate of 0.003. For the random forest (RF) and XGBoost models, `optuna` was used for hyperparameter optimization. Maximum performances are highlighted in bold.

| Size | Model | Accuracy | F1 Macro | F1 Micro | Kappa |
|---|---|---|---|---|---|
| 300 | **GPT-J (LLM)** | **0.91** | **0.91** | **0.91** | **0.82** |
| | Llama (LLM) | 0.91 | 0.91 | 0.91 | 0.81 |
| | Mistral (LLM) | 0.86 | 0.86 | 0.86 | 0.72 |
| | RF | 0.89 | 0.89 | 0.89 | 0.79 |
| | XGBoost | 0.88 | 0.88 | 0.88 | 0.76 |
| | Zero-rule | 0.50 | 0.50 | 0.50 | 0.00 |

**Table 8. Overview of results of LLMs and "traditional" ML predicting the binary class of the glass transition temperature of monomers.** Three runs were performed to get the metrics average. All LLMs were fine-tuned with 20 epochs and a learning rate of 0.003. For the random forest (RF) and XGBoost models, `optuna` was used for hyperparameter optimization. Maximum performances are highlighted in bold.

| Size | Model | Accuracy | F1 Macro | F1 Micro | Kappa |
|---|---|---|---|---|---|
| 300 | GPT-J (LLM) | 0.80 | 0.80 | 0.80 | 0.60 |
| | Llama (LLM) | 0.79 | 0.79 | 0.79 | 0.59 |
| | **Mistral (LLM)** | **0.84** | **0.84** | **0.84** | **0.68** |
| | RF | 0.74 | 0.74 | 0.74 | 0.48 |
| | XGBoost | 0.80 | 0.80 | 0.80 | 0.60 |
| | Zero-rule | 0.50 | 0.50 | 0.50 | 0.00 |

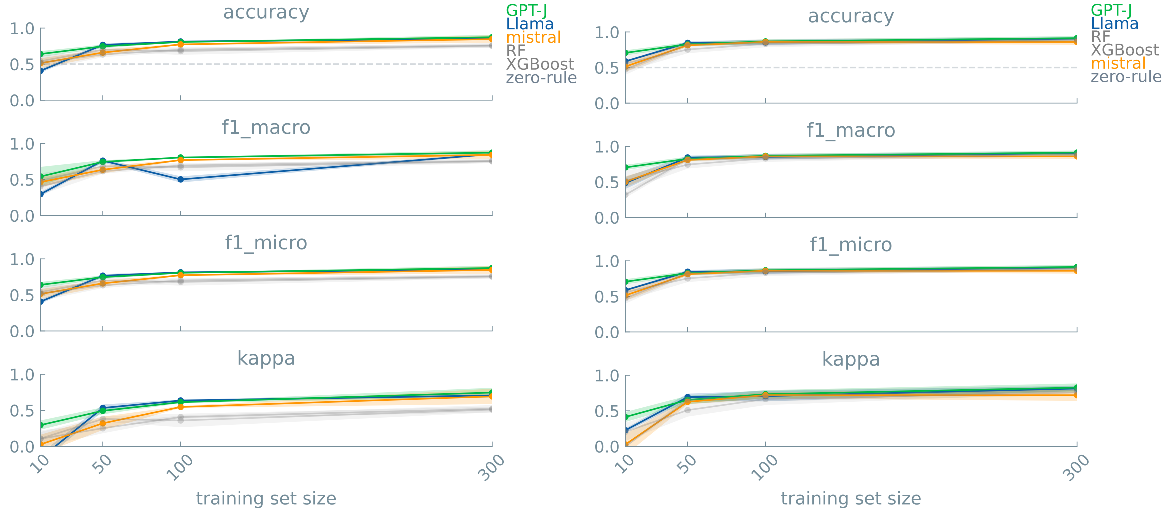


**(a) Density of monomers.** **(b) Squared radius of gyration of monomers.**

**Figure 8. Learning curve analysis of predictions for the density (a) and the squared radius of gyration (b) of monomers using different models.** Three LLMs (GPT-J, Llama, and Mistral) and two traditional ML models (XGBoost and random forest (RF)) were validated on predicting the binary class of the monomer's property. We used 50% as a random guess accuracy (dashed line), representing the zero rule baseline, i.e., a model that always predicts the most common class. Three runs were performed for each model to get the metric's average and standard deviation. A maximum accuracy of 88% of the GPT-J model and 91% of the GPT-J model were reached for the density and the squared radius of gyration, respectively, using a training set size of 300 and 20 epochs.

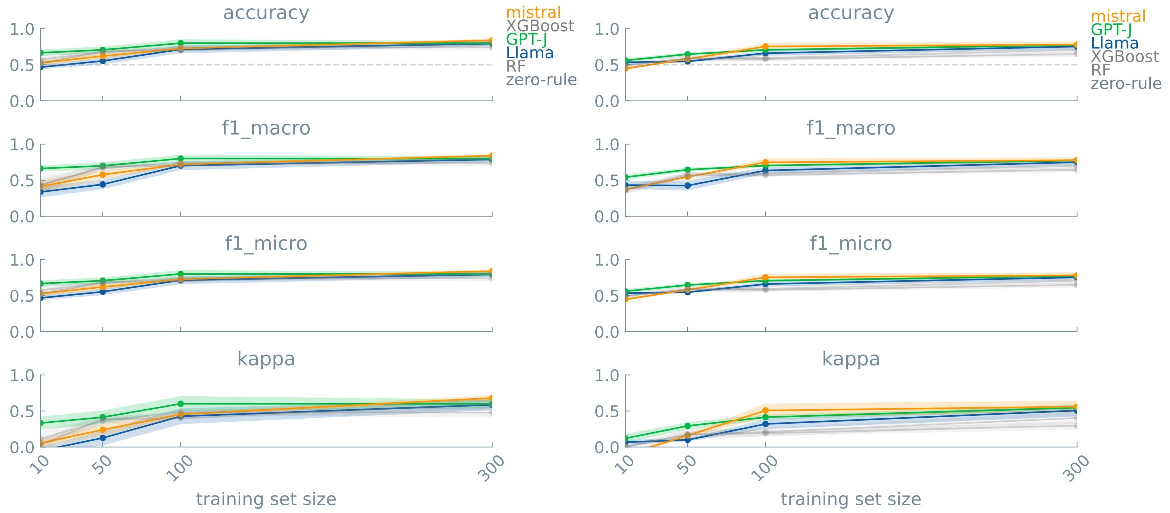


**(a) Glass Transition Temperature.** **(b) Cohesive Energy.**

**Figure 9. Learning curve analysis of glass transition temperature (a) and cohesive energy (b) of monomers using different models.** Three LLMs (GPT-J, Llama, and Mistral) and two traditional ML models (XGBoost and random forest (RF)) were validated on predicting the binary class of the monomer's property. We used 50% as a random guess accuracy (dashed line), representing the zero rule baseline, i.e., a model that always predicts the most common class. Three runs were performed for each model to get the metric's average and standard deviation. A maximum accuracy of 84% of the Mistral model and 78% of the Mistral model were reached for the glass transition temperature and the cohesive energy, respectively, using a training set size of 300 and 20 epochs.

## 3.3 Melting Point of Molecules

The dataset was provided by: Igor Tetko and Guillaume Godin[3]

### 3.3.1 Scientific Background

The melting point of molecules holds great significance in chemistry in particular due to their importance for characterisation of chemical solubility. Accurately predicting these values is, therefore, a focus of many research groups. Due to the complexity of the process, computational methods are still struggling with the task at hand. On the other hand, given a set of experimentally obtained melting points, machine-learning approaches could circumvent the need for tedious measurements. Still, descriptors, often hard to obtain, need to be included to represent the small molecules. Tetko et al.[12] combined various datasets and ML models in a comprehensive study which was further extended by melting points mined from patents.[13] They reported an average RMSE of 31 °C to 36 °C on melting point predictions of small molecules. Here, we aimed to predict melting points solely from the name and chemical structures of molecules using LLMs.

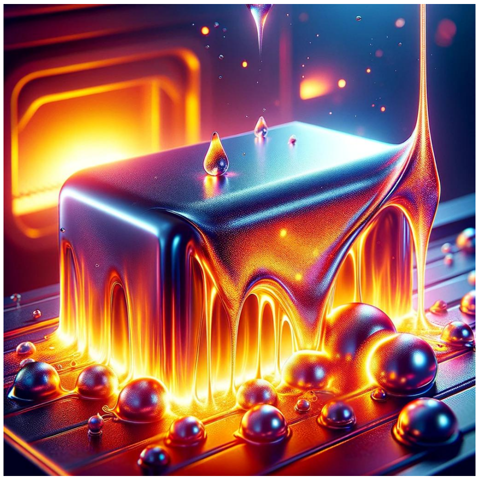

**Figure 10.** AI generated representation of a metallic material transitioning from solid to liquid under intense heat.

### 3.3.2 Dataset

The dataset contains 274,983 small molecules represented in the SMILES notation, their common chemical name, and their melting points. The distribution of the melting point values is shown in Figure 11. The dataset was split on the median of the melting point (147 °C) to create a balanced binary classification.

An example of the prompt template used in the experiments is shown in Table 9. Table 10 shows the different representations of the molecules used in the experiments.

### 3.3.3 LLM Results

**GPT-3.5 benchmark** We first benchmarked the chemical knowledge of OpenAI's GPT-3.5 on melting points via ChatGPT. For 20 randomly selected entries, we prompted: "What is the estimated melting point of <*name of molecule* > ?" The average absolute difference between

[3]BIGCHEM GmbH, Valerystraße 49, 85716 Unterschleißheim, Germany

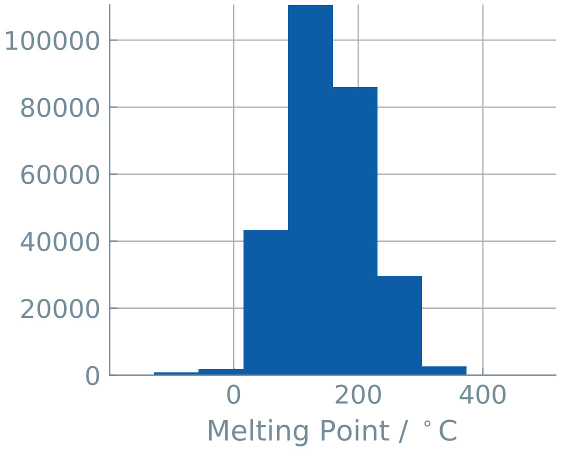


**Figure 11. Distribution of the melting points of the dataset.** The median melting point is 147 °C.

**Table 9. Example prompts and completions for predicting the melting point of molecules.** `<molecule>` serves as a placeholder for the representation of the molecule as shown in Table 10.

| prompt | completion | experimental |
|---|---|---|
| `What is the Melting point of <molecule>?` | 0 | Low |
| `What is the Melting point of <molecule>?` | 1 | High |

the reported value and the output of GPT-3.5 was 60.4 °C. If we classify this estimated GPT-3.5 output, i.e., higher or lower than 147 °C, we notice that, compared to the reported values, there was an agreement in $< 50\%$ of the cases. These results motivated the fine-tuning of LLMs in an attempt to increase the predictive power.

**Base Case** As an initial test, we split our dataset into two equally sized classes based on the melting point values. Entries with values higher than the median of 147 °C are labeled '1', and entries with lower values are labeled '0'. We performed learning curve analyses for this binary classification of the melting point using different models (Figure 12 and Table 11). For all models, the number of epochs is constant, i.e., 20. Three LLMs, i.e., GPT-J, Llama, and Mistral, were fine-tuned on the SMILES notation of the molecules. We notice that a maximum accuracy of 69% was reached with the GPT-J model. In addition, the fine-tuned LLMs were compared with "traditional" ML models (XGBoost and random forest (RF)). For these experiments, the SMILES of the molecules were converted into Morgan fingerprints.

**Table 10. Representation of the molecules.** Two example entries illustrate the different representations of the molecules.

| Representation | Example 1 | Example 2 |
|---|---|---|
| Name | N-morpholinomethyl-2-(2-pyridyl)thiopropanamide | 1-(3-Chloro-4-ethylcinnamamido)hydantoin |
| SMILES | O1CCN(CC1)CNC(C(C)C2=NC=CC=C2)=S | ClC=1C=C(C=CC(=O)NN2C(=O)NC(=O)C2)C=CC1CC |
| shuffled SMILES | OCNC2CNCCCCC)=(1)==C((C)CCN12SC= | =C))C(CC2C2CCNC=CCCCC==O=((1)lCNN=1)C=OO( |
| Length | 32 | 41 |
| Melting Point / °C (reported) | 69.0 | 232.5 |
| Bin | 0 | 1 |
| Melting Point / °C (GPT-3.5) | 90-120 | 200 |

We can conclude that LLMs can in general compete with these models.

We then compared the influence of different representations of the molecules using the GPT-J model. We notice that the model trained on 1,000 training points with the SMILES as the molecule's descriptor reaches an accuracy of 69% (Figure 13). Models trained on the chemical name performed slightly worse, with a maximum accuracy of 66%. If we combine both the name and the SMILES in one prompt, we even get a slight increase in accuracy to 71% (Figure 14).

**Table 11. Overview of results of LLMs and "traditional" ML predicting the binary class of the melting point of small molecules.** Three runs were performed to get the metrics average. All LLMs were fine-tuned with 20 epochs and a learning rate of 0.003. For the random forest (RF) and XGBoost models, optuna was used for hyperparameter optimization. Maximum performances are highlighted in bold.

| Size | Model | Accuracy | F1 Macro | F1 Micro | Kappa |
|---|---|---|---|---|---|
| 1000 | **GPT-J (LLM)** | **0.69** | **0.69** | **0.68** | **0.37** |
| | Llama (LLM) | 0.59 | 0.57 | 0.59 | 0.17 |
| | Mistral (LLM) | 0.57 | 0.56 | 0.57 | 0.13 |
| | RF | 0.68 | 0.67 | 0.68 | 0.34 |
| | XGBoost | 0.66 | 0.66 | 0.66 | 0.31 |
| | Zero-rule | 0.50 | 0.50 | 0.50 | 0.00 |

**Learning chemistry** Next, we checked whether the models truly learn from the chemistry, i.e., the elemental and structural information from the SMILES. We used the length of the SMILES string to train the binary classifier. This description holds no chemical information, rather it represents the number of individual atoms and thus the size of the molecule. Indeed, the lower accuracy obtained (58%) hints that the LLMs could make better predictions when a chemically relevant context is given. An accuracy of 61% was obtained from models trained on shuffled SMILES notation for each entry (see Table 10) (i.e., the elements present,

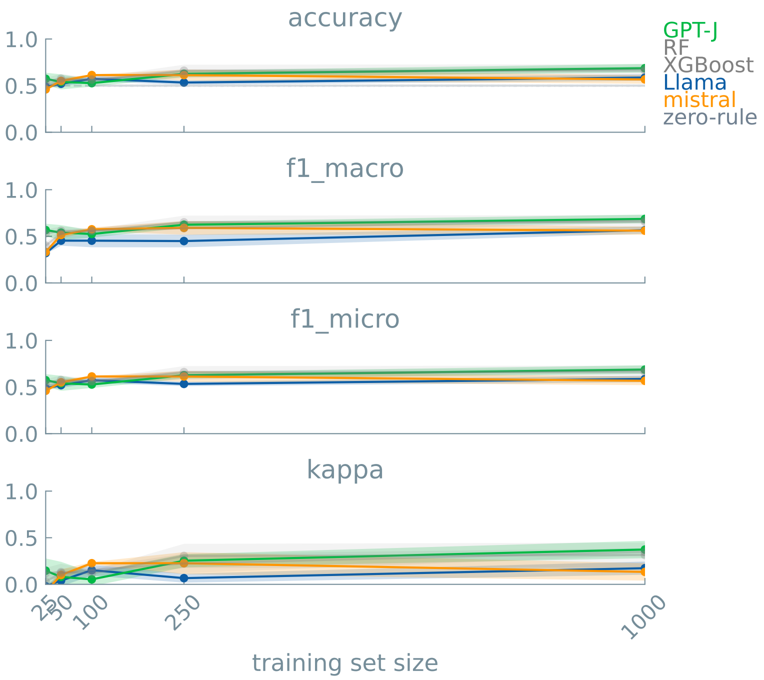


**Figure 12. Learning curve analyses of binary classification of the melting point using different models.** Three LLMs (GPT-J, Llama, and Mistral) and two traditional ML models (XGBoost and random forest (RF)) were validated on predicting the binary class of the adhesive free-energy of polymers. We used 50% as a random guess accuracy (dashed line), representing the zero rule baseline. Three runs were performed for each model to get the metric's average and standard deviation. The fine-tuned GPTJ model reached the maximum accuracy of 69% (training set size of 1,000 and 20 epochs).

without any info of the bonds), which further underpins the need for a chemical context to make useful predictions (Figure 14). Figure 15 shows the confusion matrices for the models trained using a training set of 1,000 data points and 20 epochs. We can also see the worse prediction when using the length of the SMILES string, as well as when using the shuffled SMILES notation.

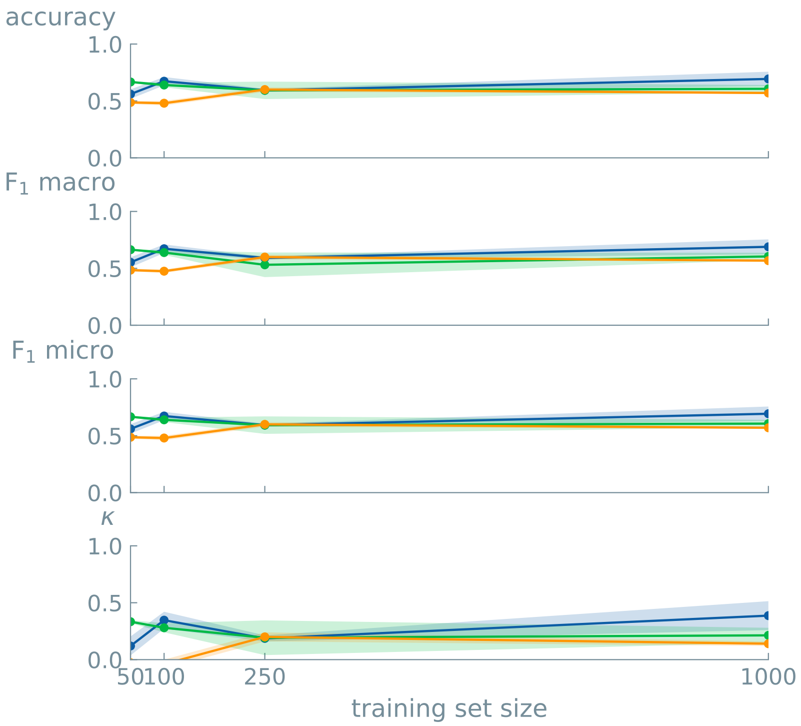


**Figure 13. Learning curve analysis for predictions on the melting point of the small molecules.** For all experiments, the GPT-J model was fine-tuned. The small molecules were represented in SMILES (blue), a common chemical name (green), or the length of the SMILES (orange). Three separate models were trained and tested, where the average and the standard deviation were plotted. All models were trained with 25 epochs. A maximum accuracy of 69% was reached for a training set size of 1,000 and 25 epochs when the GPT-J model was trained on the SMILES of the molecules.

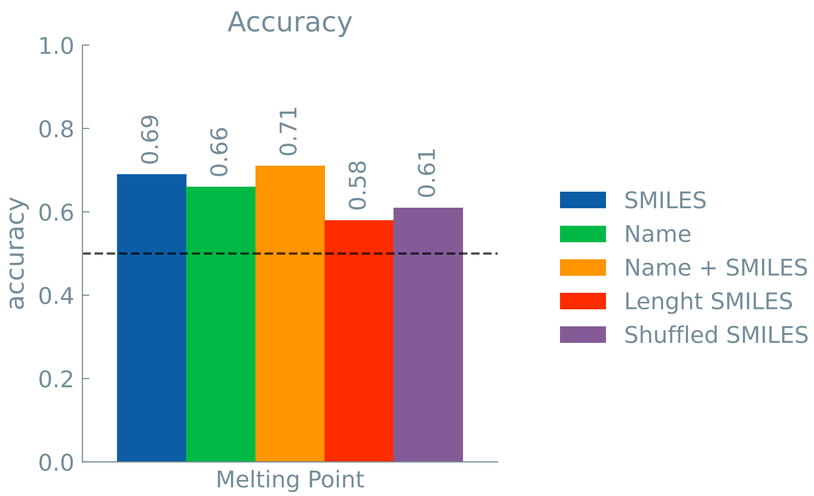


**Figure 14. Accuracies for binary classification based on five different representations of the molecules.** The same workflow was used for all four representations: GPT-J model, training set size of 1,000, 25 epochs, test set size of 50, and average metric over three different models. The dotted black line represents the accuracy of the baseline model of random guessing, i.e., 50%.

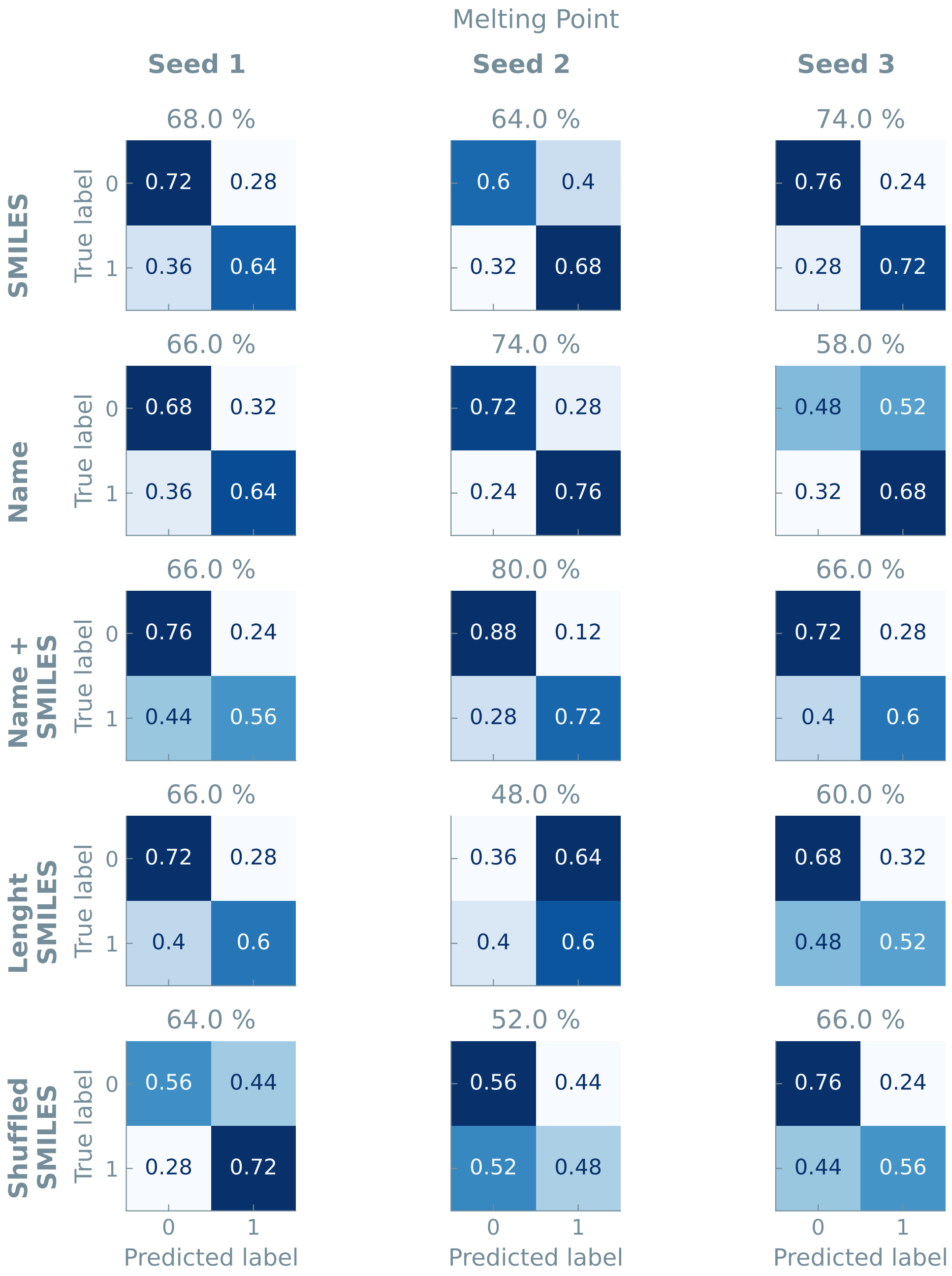


**Figure 15. Normalized confusion matrices of the melting point classifiers.** Three experiments, i.e., seeds, were performed for every representation. All GPT-J models were trained on a training set size of 1,000 data points and 25 epochs. The accuracy of the model is given above every confusion matrix.

### 3.4 Dynamic Viscosity of Molecules

The dataset was provided by: Mahyar Rajabi-Kochi and Mohamad Moosavi[4]

#### 3.4.1 Scientific Background

The dynamic viscosity of fluids is a critical parameter in numerous scientific and engineering disciplines. Understanding and predicting this property is essential for designing and optimizing various processes, such as fluid transport in pipelines, cooling applications, and lubrication systems. Due to the complex behavior of fluids under different temperature and pressure conditions, experimental measurements of dynamic viscosity are invaluable. However, these data can inform and refine theoretical models or computational simulations aimed at predicting fluid behavior. Machine learning techniques, when applied to well-curated datasets of dynamic viscosity, can bypass complex rheological computations, which are often resource-intensive. For instance, by leveraging such datasets, we can develop predictive models that estimate viscosity based on molecular composition and thermodynamic conditions. Several notable studies utilized an aggregation of diverse viscosity data and machine learning models to achieve significant accuracy improvements in predicting the viscosity of complex mixtures, underscoring the practical benefits of these datasets in both experimental settings and large-scale industrial applications.[14]

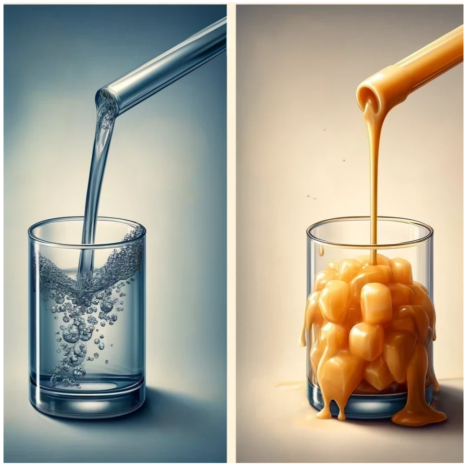

**Figure 16.** AI generated representation of the viscosity of molecules.

#### 3.4.2 Dataset

We compiled a dataset from the NIST database and supplemented it with recently published experimental works.[15,16] This dataset includes data points for 100 pure fluids and the corresponding dynamic viscosity at 298.15 Kelvin. Each fluid is represented by its SMILES string, facilitating straightforward chemical structure identification and further computational analysis. Therefore, we predict the value of the viscosity as a function of SMILES. The distribution of the viscosity values in the dataset is shown in Figure 17.

We used a simple prompt template, shown in Table 12, for experiments to predict dynamic viscosity of molecules.

[4]Chemical Engineering & Applied Chemistry, University of Toronto, Toronto, Ontario M5S 3E5, Canada

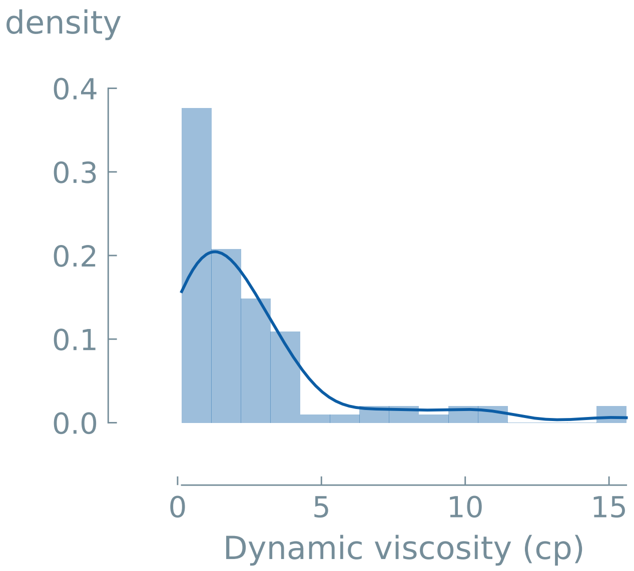


**Figure 17. Distribution of the dynamic viscosity values in the dataset.** The median dynamic viscosity is 1.7 cp.

**Table 12. Prompt templates and completions for predicting the dynamic viscosity of molecules.** `<SMILES>` is the placeholder for the SMILES notation of the molecules.

| prompt | completion | experimental |
|---|---|---|
| `What is the viscosity of <SMILES>?` | 0 | Low |
| `What is the viscosity of <SMILES>?` | 1 | High |

### 3.4.3 LLM results

**GPT-3.5** We first evaluated the chemical knowledge of OpenAI's GPT-3.5 on viscosity values via ChatGPT. For 100 entries, we prompted: "What is the viscosity of <*name of molecule*> ?" The results showed that viscosity is predicted with 55% accuracy (considering a threshold of 1.7 cp) from the name of the chemical, i.e., the prediction is no much better than random guessing.

**Base Case** To train a binary classification model, we split the dataset into two classes of equal size based on the dynamic viscosity value separated by the median, i.e., dynamic viscosity threshold of 1.7 cp. We fine-tuned three LLMs, i.e., GPT-J, Llama, and Mistral, and we also trained two "traditional" ML models, i.e., XGBoost and random forest (RF), for comparison purposes. To train RF and XGBoost, the SMILES of the molecules were converted into

Morgan fingerprints. Table 13 and Figure 18 show that the models trained with 30 epochs perform much better than random guess (shown by the dashed line). The highest accuracy was achieved with the GPT-J and RF models (80%) for a training set of 80 data points. XGBoost provided a similar accuracy (79%). Slightly lower accuracy values were obtained with Llama (68%) and Mistral (64%).

**Table 13. Overview of the accuracy results of LLM and "traditional" ML models for binary classification (balanced classes) of the dynamic viscosity of molecules.** Five runs were performed to get the metrics average. LLMs were fine-tuned with 30 epochs and a learning rate of 0.0003. Maximum performances are highlighted in bold.

| Size | Model | Accuracy | F1 Macro | F1 Micro | Kappa |
|---|---|---|---|---|---|
| 80 | **GPT-J (LLM)** | **0.80** | **0.79** | **0.80** | **0.60** |
| | Llama (LLM) | 0.68 | 0.66 | 0.68 | 0.35 |
| | Mistral (LLM) | 0.64 | 0.61 | 0.64 | 0.28 |
| | **RF** | **0.80** | **0.79** | **0.80** | **0.60** |
| | XGBoost | 0.79 | 0.79 | 0.79 | 0.58 |
| | Zero-rule | 0.50 | 0.50 | 0.50 | 0.00 |

As an example, Figure 19 shows an averaged (over five independent runs) normalized confusion matrix for the GPT-J model trained using a training set of 80 data points and 30 epochs. We can see the good prediction performance of the results, although the model fails more often in predicting samples labeled '1'.

**Real Split** To simulate a more realistic case, we trained a binary classification model using an unbalanced dataset to predict dynamic viscosity values within the top 28% highest values in the dataset (dynamic viscosity threshold = 3 cp). Three LLMs (GPT-J, Llama, and Mistral) and two "traditional" ML models (XGBoost and RF) were also fine-tuned with an unbalanced dataset using 30 epochs. Figure 20 shows that the LLM models do not perform better than random guess (shown by the dashed line), obtaining an accuracy of 69-72% when using a training set of 80 data points and 30 epochs. RF and XGBoost models perform only slightly better than random guess (82 and 79%, respectively).

As an example, Figure 21a shows an averaged (over five independent runs) normalized confusion matrix for the GPT-J model trained with this unbalanced dataset using a training set of 80 data points and 30 epochs. We can see that the model fails to predict samples labeled '1'.

However, we obtained better predictions of the viscosity values higher than 3 cp when we used a balanced dataset created by undersampling the majority class (label = 0) at the cost of reducing the size of the dataset. An accuracy of 80% was achieved using a training set of 50 data points after increasing the number of fine-tuning epochs to 140, which is

similar to that achieved with the initial balanced 50/50% larger dataset. The normalized confusion matrix in Figure 21b shows that the proportion of right predictions is above the random guess also in the case of a smaller balanced dataset. If we compared these results with those in Figure 19, we can see that a slightly higher accuracy to predict samples labeled '0' is achieved when using more training data points.

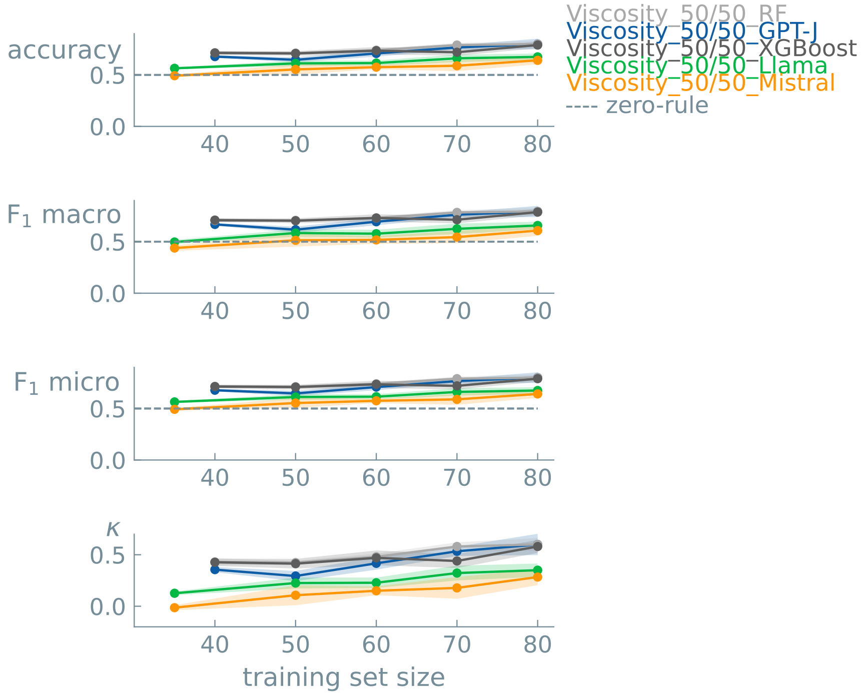


**Figure 18. Learning curves for binary classification models (balanced classes) for the dynamic viscosity of molecules.** Data points indicate the mean value of five different experiments. Error bands show the standard error of the mean. We used 50% as random guess accuracy (dashed line), representing the zero rule baseline, i.e., a model that always predicts the most common class. Accuracy: GPT-J=0.800±0.052, Llama=0.675±0.032, Mistral=0.642±0.040, random forest=0.800±0.031, XGBoost=0.790±0.031 (LLM epochs = 30, LLM learning rate = 0.0003, random forest and XGBoost=default parameters, training set size = 80 data points).

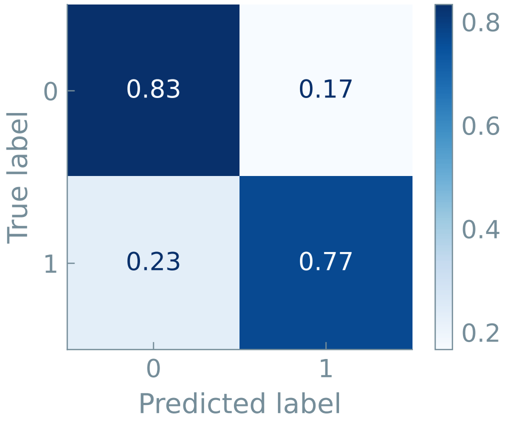


**Figure 19. Normalized confusion matrix, averaged over five independent runs, on the holdout test data for dynamic viscosity prediction with the GPT-J model.** Models were trained using a training set of 80 data points and 30 epochs (accuracy = 80%).

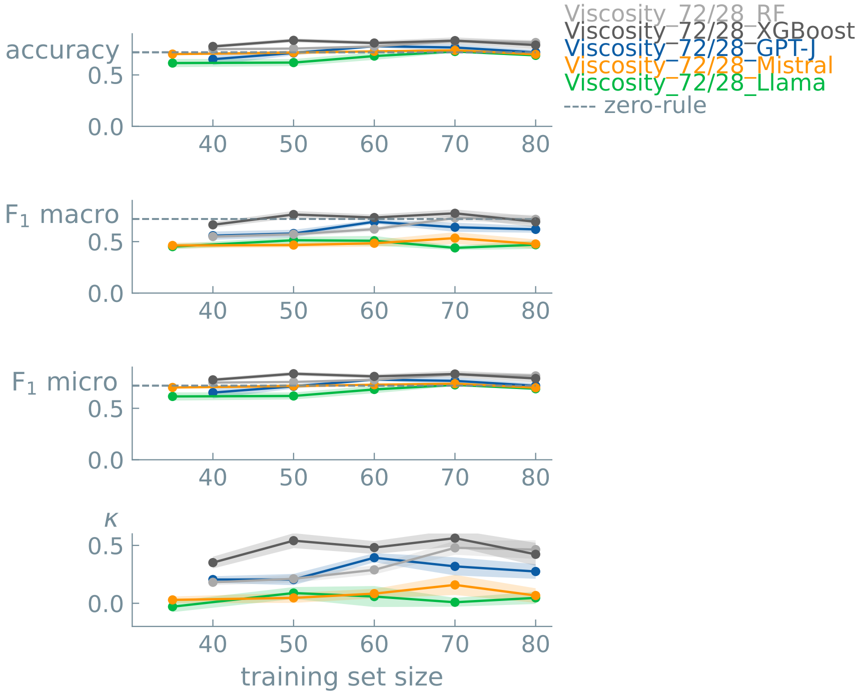


**Figure 20. Learning curves for binary classification models (unbalanced classes, 72/28%) for the dynamic viscosity of molecules.** Data points indicate the mean value of five different experiments. Error bands show the standard error of the mean. We used 72% as a random guess accuracy (dashed line), representing the zero rule baseline, i.e., a model that always predicts the most common class. Accuracy: GPT-J=0.719±0.024, Llama=0.690±0.010, Mistral=0.700±0.016, random forest=0.815±0.026, XGBoost=0.790±0.041 (LLM epochs = 30, LLM learning rate = 0.0003, random forest and XGBoost=default parameters, training set size = 80 data points).

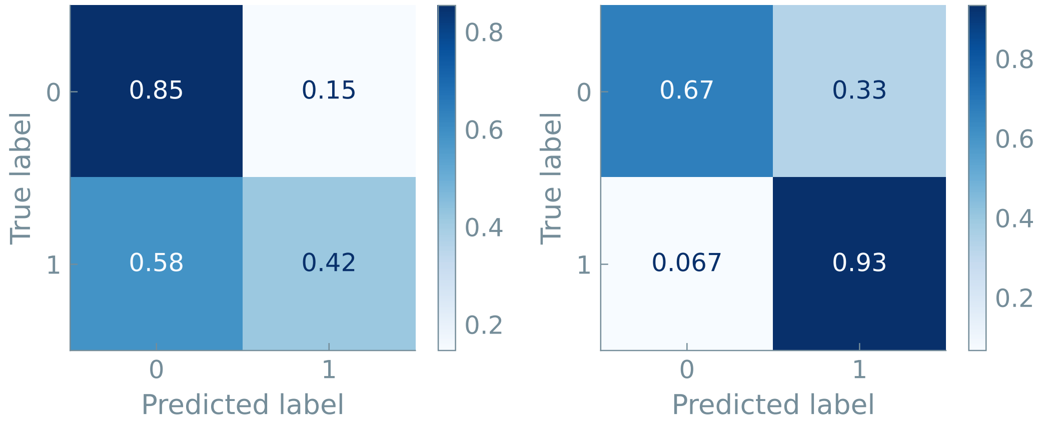


**(a)** **(b)**

**Figure 21. Normalized confusion matrix, averaged over five independent runs, on the holdout test data for dynamic viscosity prediction with the GPT-J model.** Models were trained using an 'unbalanced' dataset with 72% of labels equal to '0', a training set of 80 data points and 30 epochs (accuracy = 72%) (a), and using a 'balanced' dataset with a training set of 50 data points and 140 epochs (accuracy = 80%) (b).

## 3.5 Microstructural Properties of Magnesium Alloys

The dataset was provided by: Jianan Gu, Domonkos Tolnai, D.C. Florian Wieland, and Regine Willumeit-Römer[5]

### 3.5.1 Scientific Background

Due to their light weight, Mg alloys gain popularity in structural applications where weight saving is of importance.[17] Furthermore, owing to their degradation under physiological conditions, specific Mg alloys are being used as degradable implants.[18] Besides a small portion produced with powder-metallurgical processing,[19–21] the majority of these alloys are cast and subsequently subjected to thermo-mechanical treatment to obtain a microstructure corresponding to a suitable property profile.[22,23] During service in a corrosive environment, stress corrosion cracking becomes the limiting factor of the service life, as the simultaneous mechanical- and corrosion-load leads to early failure due to crack initiation and growth at relatively low stress levels.[24] To understand this process, thorough knowledge of the connection of processing parameters, alloy composition, and microstructure is necessary, which can be vastly different for the different processing methods.

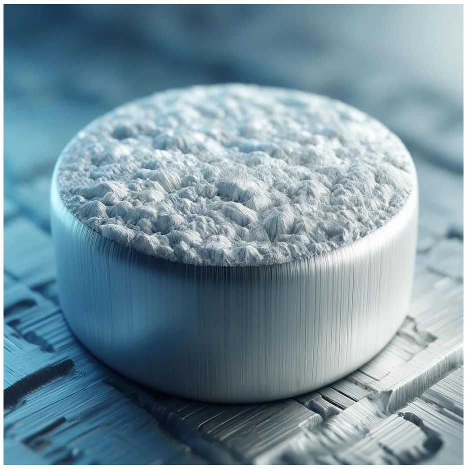

**Figure 22.** AI generated representation of an example of a magnesium alloy.

The herein-used dataset was compiled from the literature and collects important production parameters and the resulting microstructural properties[25–33] A major problem is the establishment of a generalized model for the prediction of the microstructural properties because each production route has unique process parameters. This makes traditional approaches, like e.g. random forest methods, hard to apply. Here we collect the alloy composition, production route parameters, along with the grain size and the fraction of the secondary phase. The later two parameters have an important impact on the magnesium alloy's mechanical and corrosive properties.

### 3.5.2 Dataset

The dataset contains 81 Mg alloys with their respective microstructure features for different process routes:

- **Extruded:** the alloy is forced through a die.

[5]Institute of Metallic Biomaterials, Helmholtz Zentrum Hereon, Geesthacht, Germany

- **Heat-treated:** the alloy is heated to a specific temperature for a given time and rapidly quenched down to room temperature.
- **As-cast:** the molten alloy is poured into a mold and allowed to solidify.
- **Equal channel angular extrusion (ECAE):** extrusion process in which the alloy is pressed through a die with channels intersection at an angle of 90°.

Nine additional process parameters are reported for the relevant alloys:

- **homogenized temperature:** temperature of the homogenization treatment,
- **homogenized time:** time of the homogenization treatment,
- **solutionized temperature:** temperature of the solution heat treatment,
- **solutionized time:** time of the solution heat treatment,
- **extrusion temperature:** temperature of the extrusion process,
- **extrusion speed:** speed of the extrusion process,
- **extrusion ratio:** area of the billet vs. area of the shape in the extrusion process.
- **ECAE temperature:** temperature of the ECAE process, and
- **ECAE pass:** temperature of the ECAE process.

Additionally, 13 element columns (Fe, Cu, Ni, Nd, Zn, Ca, Al, Sn, Mn, Si, Gd, Y, and Zr) are included in the dataset, describing the elemental composition of the materials as their respective weight percentage in the alloy.

In Table 14, the number of missing values per production route is summarized. The process type is highly unbalanced, with most materials manufactured via 'extrusion' and only two reported cases of ECAE. Moreover, it can be seen that the available data is variable over all production routes.

**Table 14. Missing values counts (n) for each production route and data column.**

| Process | n | $T_{homo}$ | $t_{homo}$ | $T_{sol}$ | $t_{sol}$ | $T_{ex}$ | $v_{ex}$ | $Ratio_{ex}$ | $T_{ECAE}$ | $Pass_{ECAE}$ |
|---|---|---|---|---|---|---|---|---|---|---|
| Extruded | 43 | 28 | 28 | 9 | 9 | 0 | 0 | 0 | 43 | 43 |
| Heat-treated | 13 | 7 | 7 | 0 | 0 | 13 | 13 | 13 | 13 | 13 |
| As-cast | 23 | 23 | 23 | 23 | 23 | 23 | 23 | 23 | 23 | 23 |
| ECAE | 2 | 0 | 0 | 2 | 2 | 0 | 0 | 0 | 0 | 0 |

We are interested in predicting the binary class of both the grain size and the fraction of the secondary phase. From the quick visualization of the data, we can see that both values are highly dependent on the production route (Figures 23 and 24). Lower grain sizes and lower 'second phase' values are mostly linked to an extrusion process. In contrast, higher grain sizes come from heat-treated and as-cast procedures, while higher 'second phase' values are mostly linked to as-cast processes.

An example of the prompt template used in the experiments for predicting grain size or 'second phase' of Mg alloys is shown in Table 15.

**Table 15. Example prompts and completions for predicting microstructural properties of Mg alloys.** `<Mg alloy>` serves as a placeholder for the representation of the Mg alloy process route.

| prompt | completion | experimental |
|---|---|---|
| Example of training data | | |
| `What is the grain size` *or* `second phase of <Mg alloy>?` | 0 | Low |
| `What is the grain size` *or* `second phase of <Mg alloy>?` | 1 | High |

### 3.5.3 LLM results

We used a training size of 25 and 50 epochs for the following experiments and comparisons. For these initial experiments, GPT-J was fine-tuned. When we train a model to predict the binary class of the 'second phase' (median = 1.14) on all the parameters, we get an accuracy of 79.3%. If we train a model to predict the 'second phase' on only the process route, we get an accuracy of 78.0%. We can conclude that the model mostly learns from the process production procedure, and not necessarily from all the other parameters.

However, if we then filter only the 43 entries made with the extrusion process and predict the binary class (new median of 0.65), we see that the accuracy of the model is 53.7%, i.e., not able to make any concrete predictions.

On the other hand, if we train a model to predict the binary class of the 'grain size' (median = 25.7) on all the parameters, we get an accuracy of 84.0%. If we train a model to predict the grain size on only the process route, we get an accuracy of 94.0%. We can conclude that the model mostly learns from the production process, and not from all the other parameters. The addition of extra parameters even leads to a significant decrease in model performance.

However, if we then filter only the 43 entries made with the extrusion process and predict the binary class (new median of 12.1), we see that the accuracy of the model is 55.0%, not

being able to make any concrete predictions in this case either.

We further compared different models on their predictive performance of the 'grain size'. We trained three different LLMs, i.e., GPT-J, Llama, and Mistral, and two "traditional" models (random forest (RF) and XGBoost) (Table 16). All the available parameters were used to represent the Mg alloys. We see that all LLMs provide similar accuracies (average of 89%). In comparison with "traditional" ML models, LLMs perform on average slightly worse for this dataset.

**Table 16. Overview of results of LLMs and "traditional" ML predicting the binary class of the grain size.** Three runs were performed to get the metrics average. All LLMs were fine-tuned with 25 epochs and a learning rate of 0.003. For the random forest (RF) and XGBoost models, `optuna` was used for hyperparameter optimization. Maximum performances are highlighted in bold.

| Size | Model | Accuracy | F1 Macro | F1 Micro | Kappa |
|---|---|---|---|---|---|
| 30 | GPT-J (LLM) | 0.85 | 0.85 | **0.85** | 0.63 |
| | Llama (LLM) | 0.89 | 0.89 | 0.89 | 0.79 |
| | Mistral (LLM) | 0.94 | 0.94 | 0.94 | 0.88 |
| | **RF** | **0.95** | **0.95** | **0.95** | **0.90** |
| | XGBoost | 0.95 | 0.95 | 0.95 | 0.89 |
| | Zero-rule | 0.50 | 0.50 | 0.50 | 0.00 |

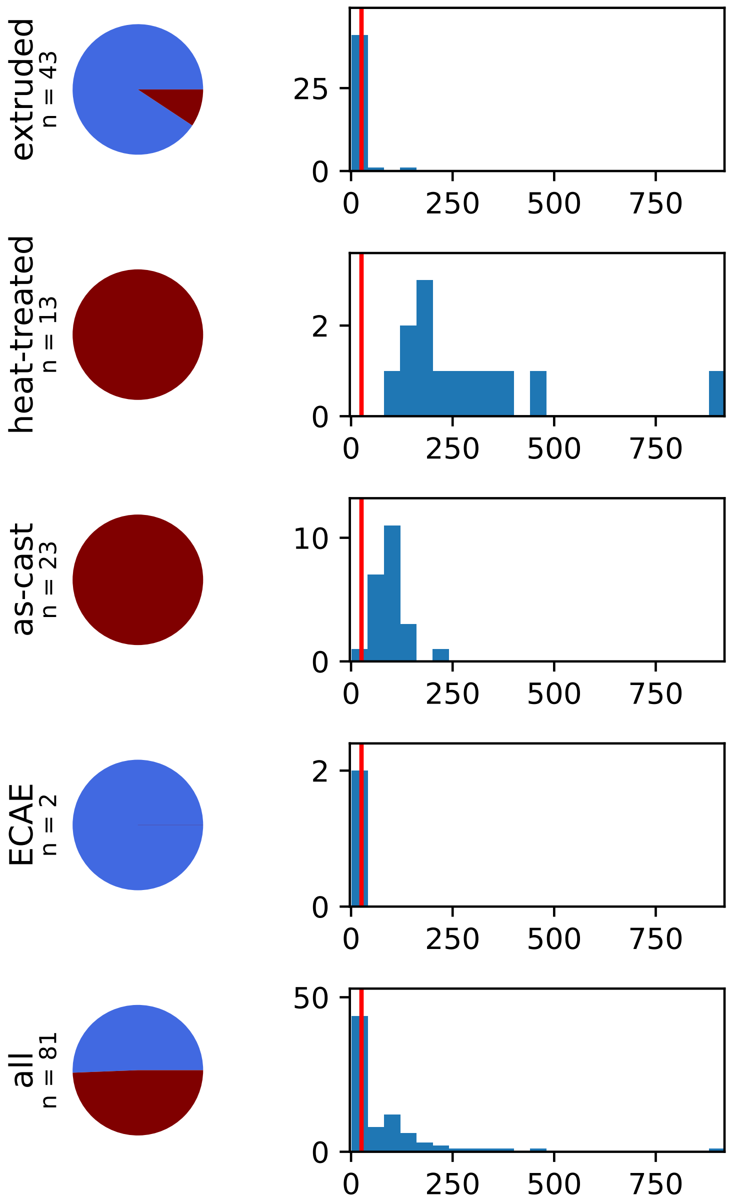


**Figure 23. Grain size analysis.** Subsets of the complete dataset are analyzed based on the production route. Pie charts display the proportion of lower grain size values (blue, size lower than median = 25.7) and higher grain size values (red, size higher than median = 25.7) in the subset. Histograms represent the distribution of the values. The red line in the subset's histogram represents the median grain size of the complete dataset, i.e., 27.7.

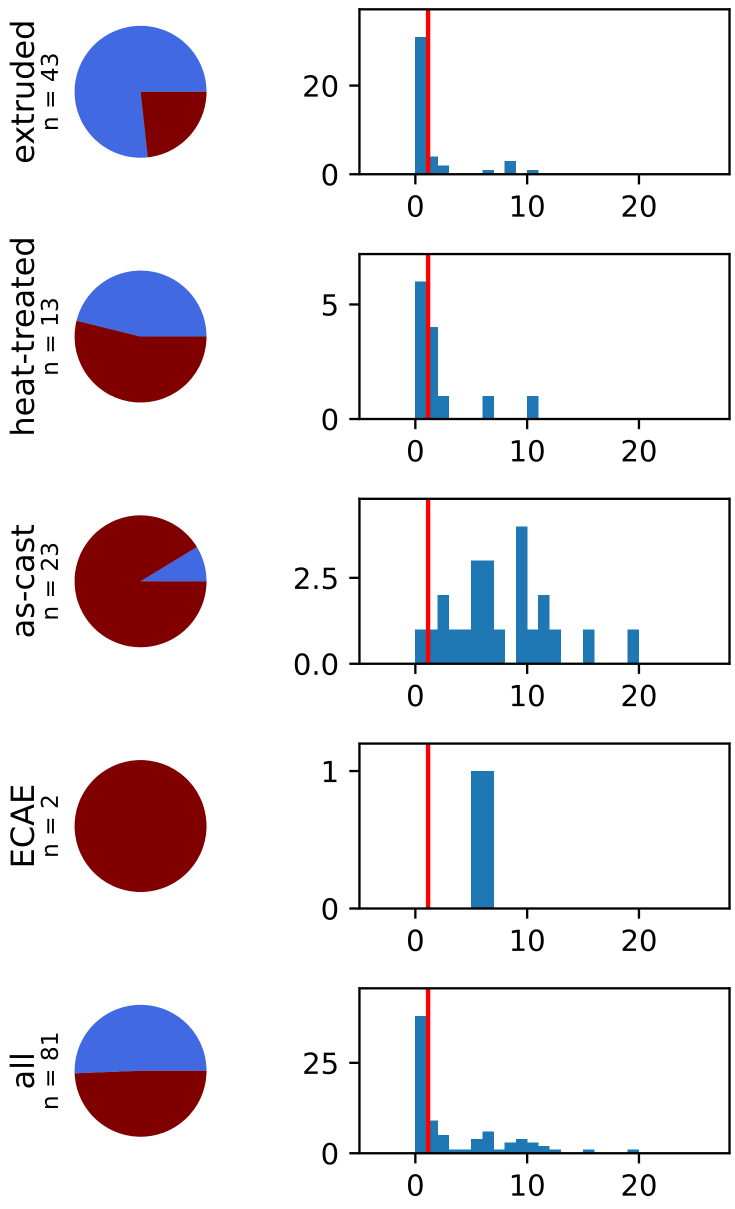


**Figure 24. 'Second phase' analysis.** Subsets of the complete dataset are analyzed based on the production route. Pie charts display the proportion of lower 'second phase' values (blue, lower than median = 1.14) and higher 'second phase' values (red, higher than median = 1.14) in the subset. Histograms represent the distribution of the values. The red line in the subset's histogram represents the median 'second phase' value of the complete dataset, i.e., 1.14.

### 3.6 Phase Separation Propensity of Proteins

The dataset was provided by: Lydia L. Good, Alex Abrudan, Tuomas P.J. Knowles[6]

#### 3.6.1 Scientific Background

Predicting phase separation propensity for proteins is important for understanding their contributions to cellular organization and function (Figure 26).[34] In such a phase separation, one can observe the formation of a protein-rich biomolecular condensate and a protein-poor phase. These condensates emerge from the natural propensity of cellular proteins to cluster, driven by specific sequences and biophysical interactions. To map out the relationship between the sequences of proteins and their tendency to undergo phase separation, Saar et al.[35] have compiled a dataset comprising proteins with diverse phase separation behaviors and analyzing them for commonalities in their sequences and biophysical properties. In addition, they developed binary classifiers based on extracted physical features of the protein sequence and an embedding of the sequence made using a word2vec model,[36] which can be used to compare predictive performances. The reader is referred to the original publication for details on the author's methodology on the word2vec model.[35]

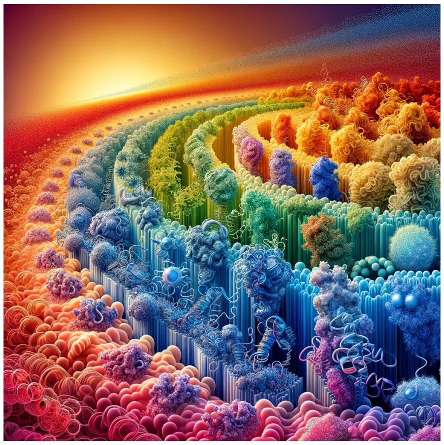

**Figure 25.** AI generated representation of the formation of protein-rich biomolecular condensates.

#### 3.6.2 Dataset

The final dataset is constructed from two publicly available datasets, the LLPSDB[37] and the PDB.[38] After cleaning and filtering of the LLPSDB, Saar et al.[35] divided the remaining structures into two datasets. The first one contained proteins with an experimental evidence documenting their homotypic phase separation at physiologically-relevant concentrations (below 100 $\mu$M) (LLPS+), while the other contained proteins that phase separate only under more extreme conditions (concentrations above 100 $\mu$M) (LLPS-). All structures in the PDB dataset are highly unlikely to phase separate.

[6] Yusuf Hamied Department of Chemistry, University of Cambridge, Cambridge CB2 1EW, United Kingdom

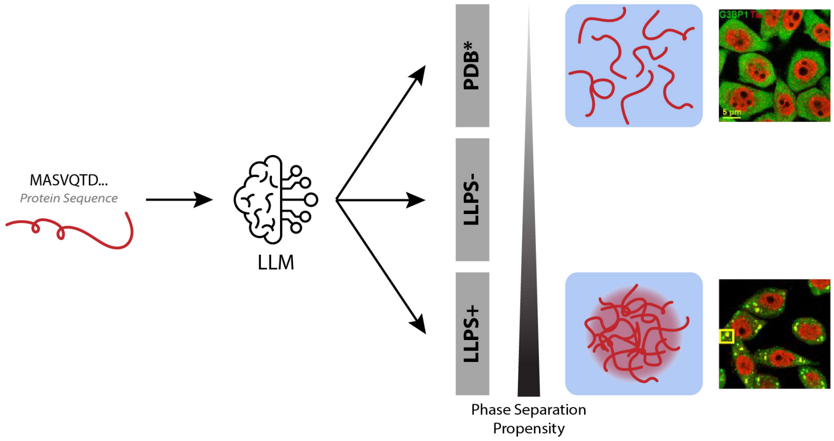


**Figure 26. LLMs to predict phase separation of proteins.** The GPT model was tasked with classifying protein sequences based on their propensity to undergo liquid-liquid phase separation, a de-mixing process that underlies the formation of membrane-less organelles within cells. Saar et al.[35] classified the protein sequences used for fine-tuning and testing the model into three groups (LLPS+, LLPS-, PDB*) based on available experimental evidence for their propensity to undergo homotypic phase separation. Microscopy images at the right hand side, which show the presence and absence of protein phase separation in cells, are adapted from Tsai et al.[39].

The distribution of these three datasets is shown in Figure 27. The imbalance was accounted by sampling the larger classes to match the size of the smallest class. For a detailed description of the dataset construction, the reader is referred to the original publication.[35]

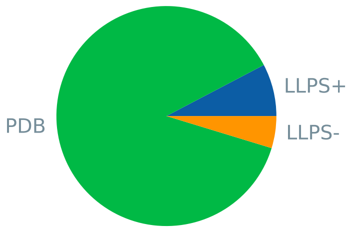


**Figure 27. Distribution of the three datasets.** In total, there are 1783 sequences (PDB 1,562 and LLPSDB 221). From LLPSDB, 84 sequences (4.7%) have a low propensity to LLPS (LLPS-) and 137 sequences (7.6%) have a high propensity to LLPS (LLPS+). All sequences from PDB (1,562 sequences, 87.6%) are highly unlikely to LLPS (LLPS-).

An example of the prompt used for the fine-tuning is shown in Table 17.

**Table 17. Example prompts and completions for predicting the propensity of proteins to phase separate.** `<protein>` serves as a placeholder for the representation of the amino acid sequence (see Table 18).

| prompt | completion | experimental |
|---|---|---|
| | Example of training data | |
| `What is the propensity to phase separate of <protein>?` | 0 | Low |
| `What is propensity to phase separate of <protein>?` | 1 | High |

**Table 18. Representation of the sequences.** Two example entries illustrate the four different representations of the sequence.

| | Example 1 | Example 2 |
|---|---|---|
| Sequence | RRGGGFG...SGDGYN | SKDHVN...GSRGGS |
| First 20 AA | RRGDGRRRGGGGRGQGGRGR | SKDHVNRIIESLEKSSSSEP |
| Length | 40 | 260 |
| Shuffled | GGFGDS...GRKTNG | TSDDYA...SITGGS |

### 3.6.3 LLM results

As in the original publication, the first experiments aimed to train a classifier that could distinguish the different datasets. A random subset of the most populated dataset (unlikely to LLPS), equal in size to the least populated dataset (some evidence of LLPS), was taken to ensure a final balanced dataset. For instance, 84 random entries from the PDB and the full LLPS+ dataset ($n$ = 84) were used to ensure training on a balanced dataset, thus obtaining less biased predictions for the binary classification. Three different LLMs, i.e., GPT-J, Llama, and Mistral, were fine-tuned to compare their predictive power with the in-house ML models of Saar et al.Saar et al. [35]. For the LLM models, the final metric was the average accuracy over three different training/test cycles. The benchmark corresponding to random guessing is an accuracy of 50%. For all three cases, all LLMs show similar accuracies to Saar et al. [35]'s model (Table 19).

Next, we experimented with the following representations of the protein sequence in the prompt (see Table 18 for examples):

**Table 19. Overview of the performance of the different models with the full sequence as the representation.** A comparison with models trained on two different featurization strategies, i.e., engineered features (EF) and single-layer language model (LM), from Saar et al.[35] was made. The LLMs (GPT-J, Llama, and Mistral) were trained on a training set size of 75 and 25 epochs. The accuracy was taken over three seeds.

| | LLPS-/PDB* | LLPS+/LLPS- | LLPS+/PDB* |
|---|---|---|---|
| # per class | 84 | 84 | 137 |
| Total dataset size | 168 | 168 | 274 |
| Training Size | 75 | 75 | 75 |
| Test Size | 50 | 50 | 50 |
| GPT-J Accuracy | 83% | 64% | **95%** |
| Llama Accuracy | 81% | 64% | 85% |
| Mistral Accuracy | 82% | 59% | 92% |
| LM Accuracy | 85% | **65%** | 89% |
| EF Accuracy | **87%** | 63% | 90% |

- The full sequence.
- The total length of the sequence, i.e., the number of amino acids in the sequence.
- Only the first 20 amino acids, i.e., the first 20 letters of the full sequence. Using only the first 20 amino acids interrogates the attention window of the model to determine whether the model has a limited attention window or considers the whole sequence, as transformers are supposed to do.
- A random shuffle of the full sequence, i.e., the same letters representing the amino acids, but now in a random order. Shuffling interrogates whether the model only learns correlations about the amino acid composition of sequences or if positional correlations are also learned.

The same training/testing workflow was used for every representation. The GPT-J model was used for these comparisons. From the accuracies plotted in Figure 28, we see similar trends in the LLPS-/PDB* and LLPS+/PDB* classifications. The full sequence has the highest performance, indicating that the models learn from the sequences' full chemical/biological context. In contrast, the sequence length does not hold any relevant information and is indeed also reflected in the lowest accuracy. The LLPS-/LLPS+ classification has similar metrics for all representations. Interestingly, we see that the LLMs obtain comparable performances to the models of Saar et al.[35] (Figure 28, dotted line (EF model)). Figures 29 to 31 show the confusion matrices of the LLPS-/PDB*, LLPS-/LLPS+, and LLPS+/PDB* classifiers, respectively.

The results of the LLPS+/PDB classifier indicate that with the characteristic amino acid composition of the disordered regions alone, we can distinguish the two classes since the "shuffled sequence" model performance is relatively close to the performance of the "sequence" model. The LLPS-/PDB classification looks like a harder problem. However, the lower accuracy could also be caused by data quality issues since the LLPS dataset is smaller and trickier to create and possibly more heterogeneous in nature. Lastly, the LLPS+/LLPS- classification is definitely a harder problem since both datasets contain partially disordered proteins, and the model cannot capture the factors that make disordered proteins more prone to LLPS, so an accuracy of 64% is not far from a random model.

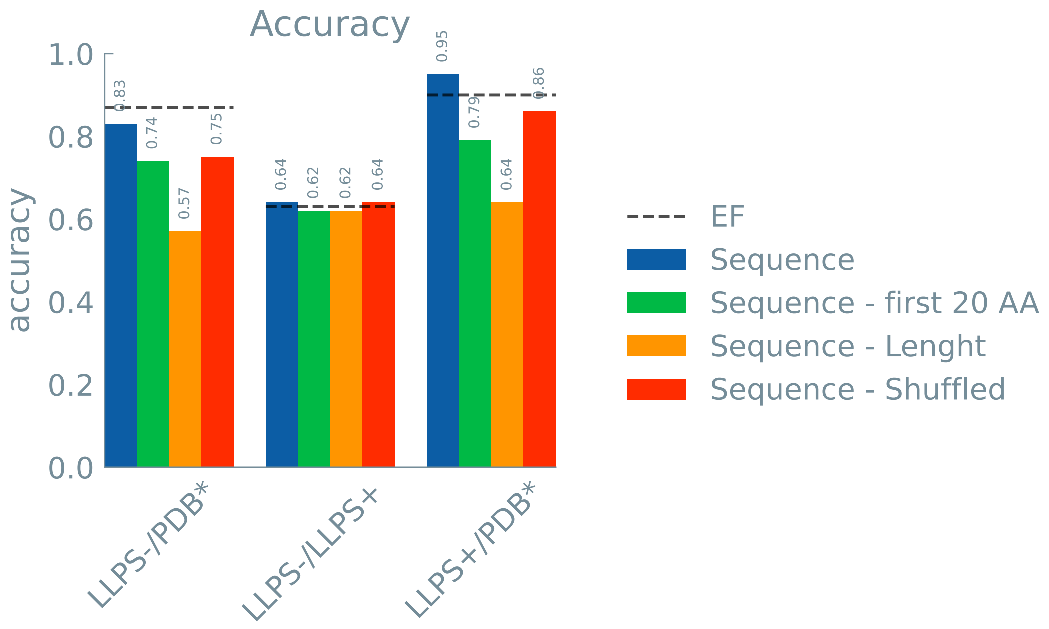


**Figure 28. Accuracies for binary classification for the different datasets trained on four different representations.** GPT-J was used as the base model. The same workflow was used for all four representations: training set size of 75, 25 epochs, test set size of 50, and average metric over three different models. The dotted black line represents the accuracy of the EF model of Saar et al.[35].

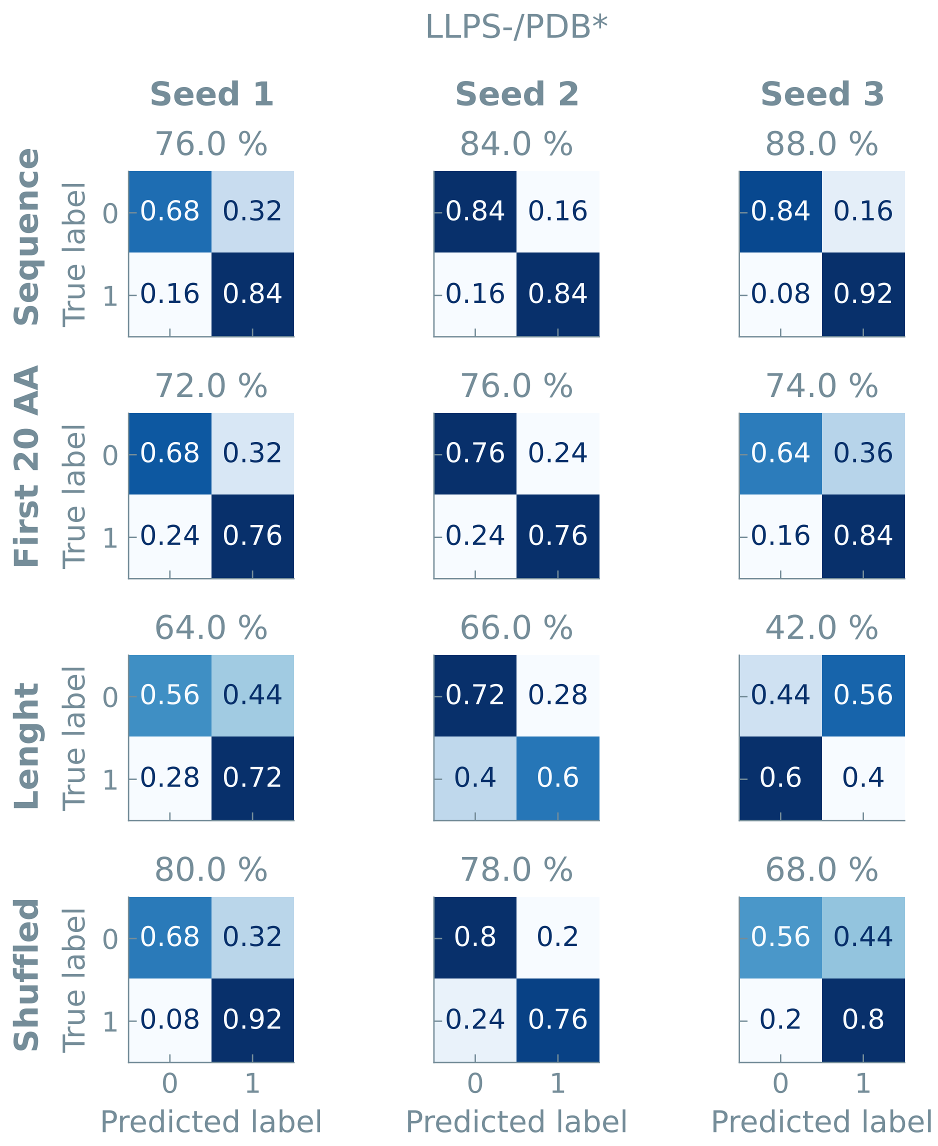


**Figure 29. Normalized confusion matrices of the LLPS-/PDB* classifiers.** Three experiments, i.e., seeds, with the GPT-J model were performed for every representation. All models were trained on a training set size of 75 and 25 epochs. The accuracy of the model is given above every confusion matrix.

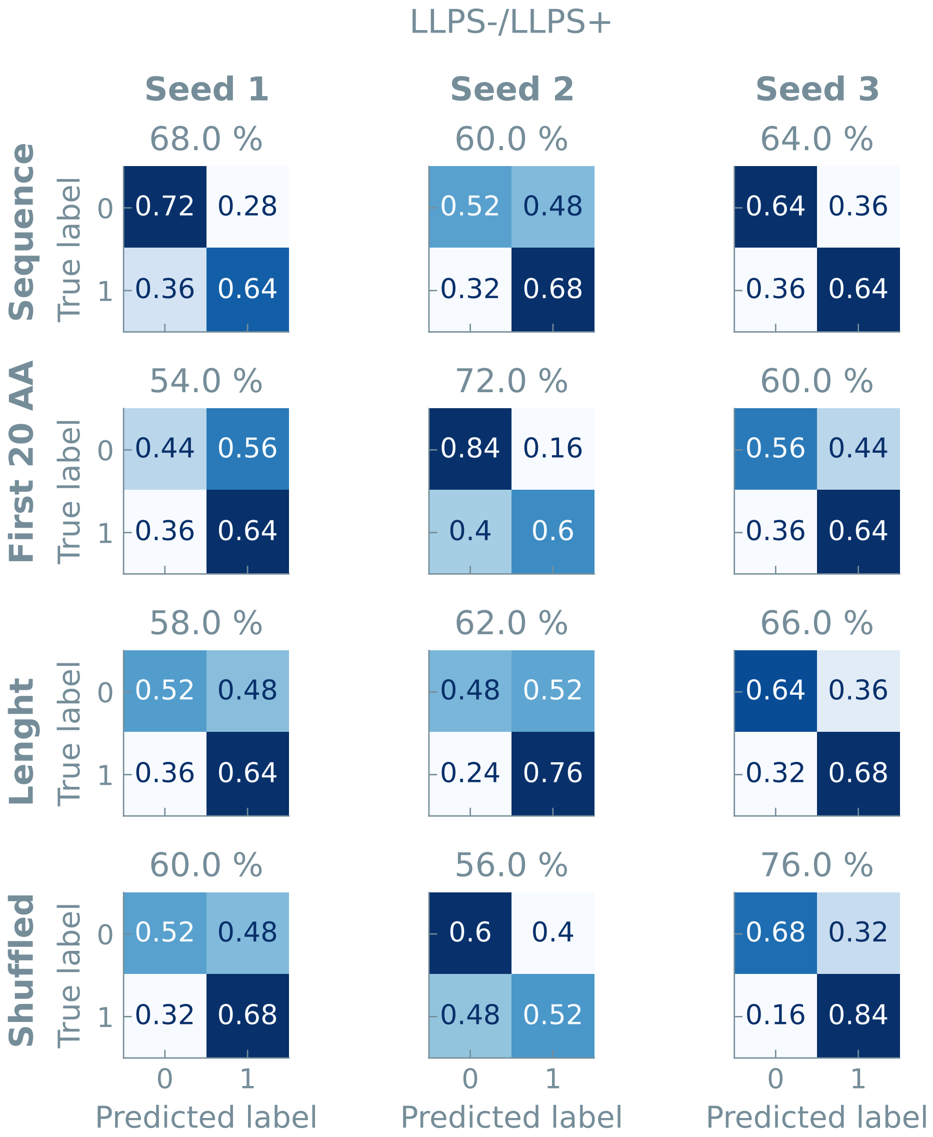


**Figure 30. Normalized confusion matrices of the LLPS-/LLPS+ classifiers.** Three experiments, i.e., seeds, with the GPT-J model were performed for every representation. All models were trained on a training set size of 75 and 25 epochs. The accuracy of the model is given above every confusion matrix.

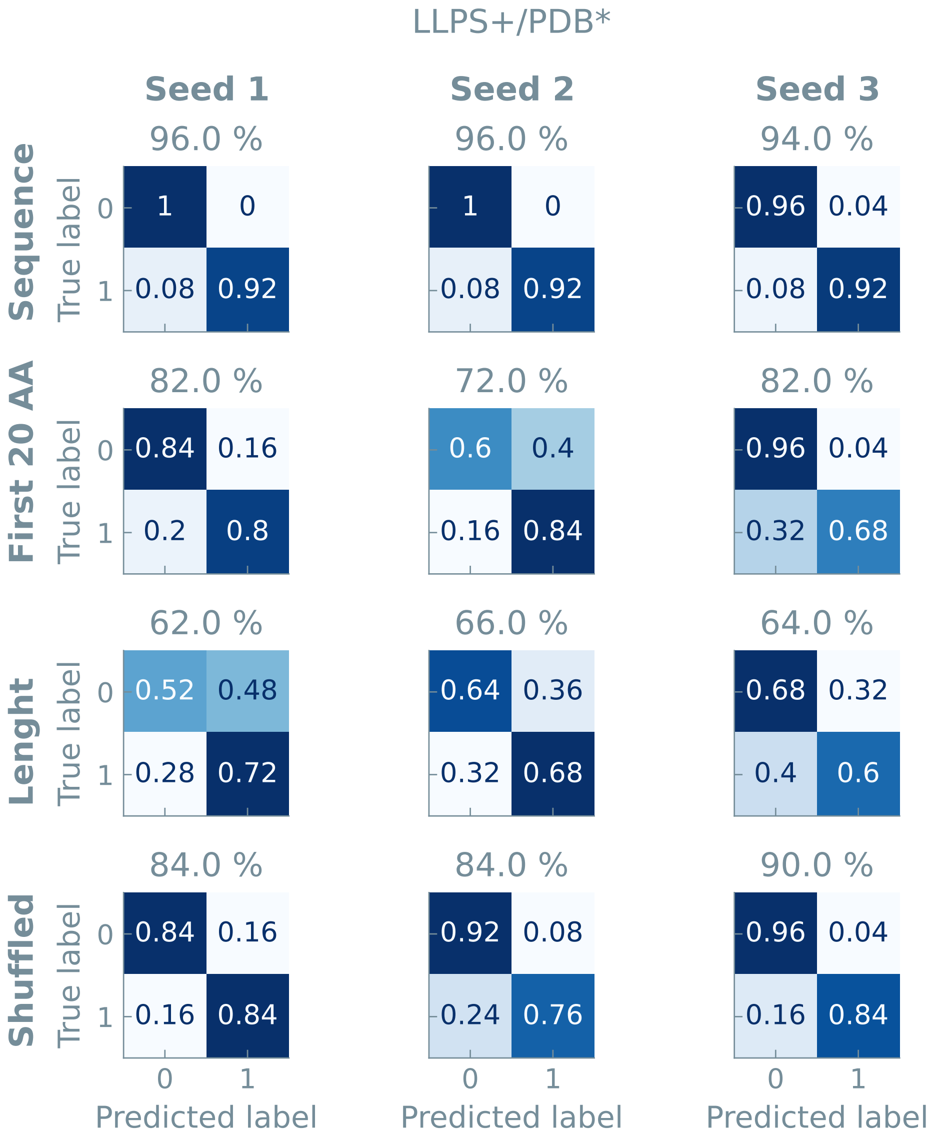


**Figure 31. Normalized confusion matrices of the LLPS+/PDB* classifiers.** Three experiments, i.e., seeds, with the GPT-J model were performed for every representation. All models were trained on a training set size of 75 and 25 epochs. The accuracy of the model is given above every confusion matrix.

## 3.7 Structure of Nanoparticles

The dataset was provided by: Andy S. Anker[7]

### 3.7.1 Scientific Background

Developing new nanomaterials for energy technologies depends on understanding the intricate relation between material properties and atomic structure. It is, therefore, crucial to be able to characterize the atomic structure in nanomaterials routinely. A promising method to elucidate size-dependent atomic structure of nanomaterials is Pair Distribution Function (PDF) analysis.[40] The PDF can be obtained through Fourier transformation of x-ray total scattering data and represents a histogram of all interatomic distances in the sample (see Figure 33). Going from the distance information in the PDF to a chemical structure is an unassigned distance geometry problem (uDGP), and solving this is often the bottleneck in nanostructure analysis. A Conditional Variational Autoencoder (CVAE) has been proposed to automatically solve the uDGP to obtain valid chemical structures from the scattering pattern.[41,42]

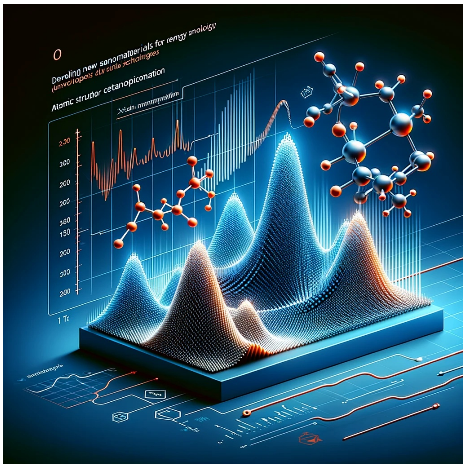

**Figure 32.** AI generated representation of the scattering of a nanoparticle.

In this work, we explore the potential of an LLM model to predict the structure type and number of atoms in nanomaterials from their scattering pattern. Predicting these values accurately is an easier task than solving the structure, and LLMs are more convenient to use for researchers than CVAEs, as they are purely based on natural language.

### 3.7.2 Dataset

The dataset includes 1957 scattering patterns of nanoparticles with seven different structure types (simple cubic (SC), body-centered cubic (BCC), face-centered cubic (FCC), hexagonal closed packed (HCP), decahedral (Dec), icosahedral (Ico), and octahedral (Oct)). These nanomaterials have up to 100 atoms. In this work, we predict a nanomaterial's number of atoms and structure type from its scattering pattern, The input to the LLM is the scattering pattern represented as series of numbers in the form of a string obtained from the Pair Distribution Function (PDF), as shown in Figure 34. The length of the input scattering pattern, i.e., the

[7] Department of Energy Conversion and Storage, Technical University of Denmark, DK-2800 Kgs. Lyngby, Denmark

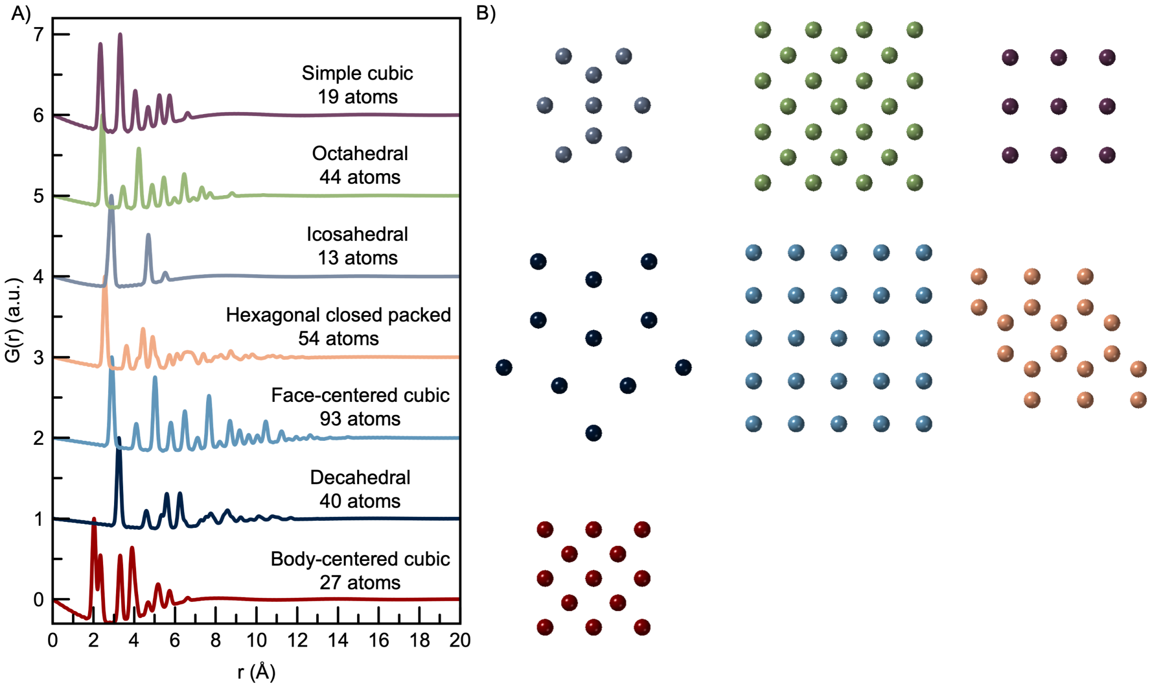


**Figure 33. Examples of simulated PDFs (A) from each one of the seven different structure types that are presented in the dataset in sizes between 5 and 100 atoms (B).**

number of points of the PDF representation, has been adjusted to comply with the model context window by uniformly sampling the entire scattering pattern. The distribution of the number of atoms and structure type values in the dataset is shown in Figure 35.

We used a simple prompt template, with prompts of the form shown in Table 20 for experiments to predict the number of atoms and structure type.

### 3.7.3 LLM results

**Structure type - Real Split** We used the original dataset, which contains seven different categories for this variable, to predict the structure type of nanoparticles. As it can be seen in Figure 35a, the distribution of data points in these categories is highly unbalanced.

For this dataset, Figure 36 shows that our GPT-J model trained with 20 epochs gives an accuracy of 93% for a training set of 1800 data points, which is significantly better than random guess (shown by the dashed line). To check if we could further improve the model's predictive performance, we did experiments with 30 epochs, but an almost similar accuracy was achieved (94%). This means the model can accurately predict the structure type despite using a highly unbalanced training set.

Figure 37 shows the averaged (over three independent runs) normalized confusion ma-

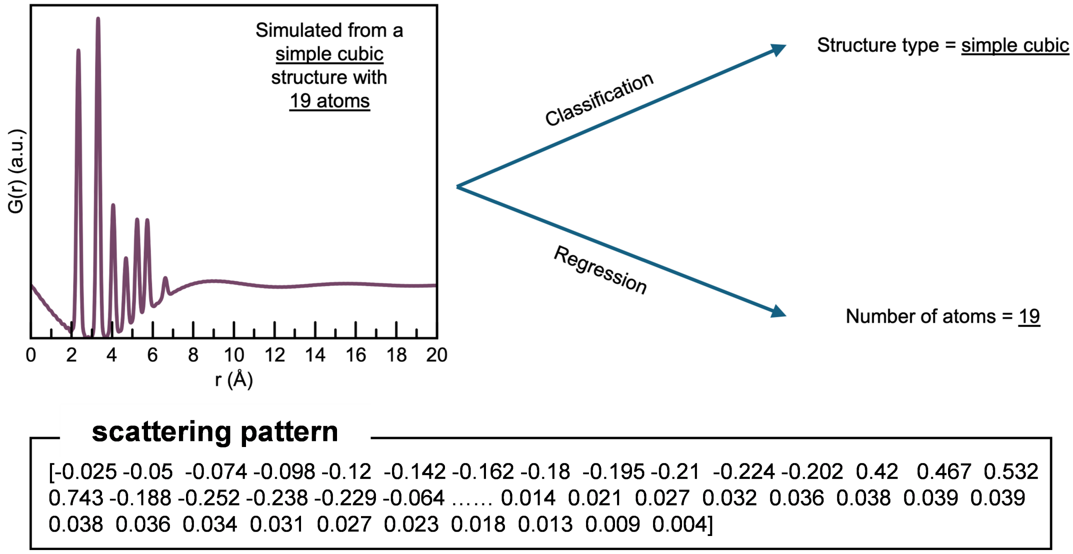


**Figure 34. Pair Distribution Function (PDF) obtained from a material and the corresponding scattering pattern string used as input to the LLM model.** In this work we perform the classification task of predicting the structure type of a material and the regression task of predicting the number of atoms from the obtained scattering pattern.

trix for the GPT-J model trained using a training set of 1800 data points, and 20 (Figure 37a) and 30 epochs (Figure 37b). We can see that the slight increase in the model's predictive performance when using 30 fine-tuning epochs is associated with higher precision to predict the less represented classes, i.e., Dec, Ico, and Oct.

We fine-tuned three LLMs, i.e., GPT-J, Llama, and Mistral, using 30 epochs. We also trained two "traditional" ML models, i.e., XGBoost and random forest (RF), for comparison purposes. Table 21 and Figure 38 show that the highest accuracy (97%) was obtained with the GPT-J model, but only slightly lower accuracy values were obtained with other models (94%).

For comparison purposes, we created a balanced 7-class dataset by sampling $n$ data points of each class, where $n$ represents the amount of data points of the least-populated class, i.e., 38 (Ico). This was done at the cost of reducing the size of the dataset to 266 data points. Figure 39 shows that the LLMs trained with 30 epochs give accuracy values of 43-56% for a training set of 200 data points, which is higher than random guess (shown by the dashed line) but much lower than the accuracy obtained for the prediction of the 7-class unbalanced dataset. The accuracy achieved with the RF and XGBoost models is higher (73% and 70%, respectively), but still lower than that obtained with the 7-class unbalanced dataset.

As an example, Figure 40 shows the averaged (over three independent runs) normalized

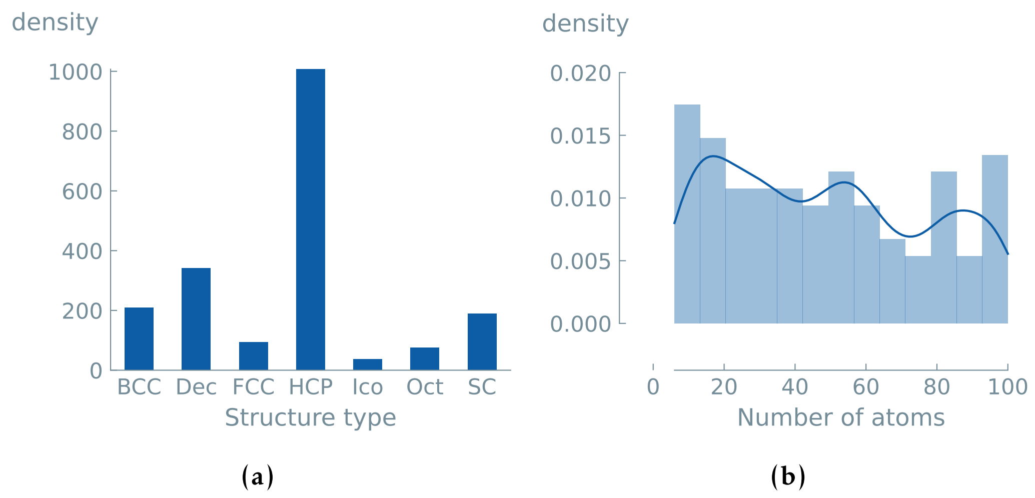


**Figure 35. Distribution of the structure type (a) and number of atoms (b) of nanomaterials in the dataset.** The nanoparticles in the dataset are distributed in 7 different structure classes, which are highly unbalanced. The median number of atoms of the nanomaterials in the dataset is 48.

confusion matrix for the GPT-J model trained using a training set of 200 data points and 30 epochs. We can see that the prediction accuracy for most of the categories is low. This indicates that we can develop a model to predict an unbalanced dataset with seven classes by using a relatively high training set size. However, when using a very low number of training data points, the fine-tuned model cannot predict a balanced dataset. The RF and XGBoost models seem to perform better with smaller datasets.

We also created balanced 5-class (Figure 41) and 4-class (Figure 42) datasets by undersampling the highest populated classes and removing the least populated classes, resulting in training dataset sizes of 425 and 700 data points, respectively. As an example, Figure 43 shows the averaged (over three independent runs) normalized confusion matrix for the GPT-J model trained using a 5-class balanced training set of 425 data points and 30 epochs (Figure 43a), and a model trained using a 4-class balanced training set of 700 data points and 30 epochs (Figure 43b). The results show that the LLMs predict four or five balanced classes with relatively high accuracy (92-96% and 83-87%, respectively). When we increase the size of the dataset, the performance of the LLMs becomes similar to that of the RF and XGBoost models. From these results, we can deduce the importance of the training set size in obtaining a high LLMs performance. Even for balanced datasets, larger training set sizes are necessary when complex inputs are given to the model.

**Number of atoms - Base Case** To train binary classification models, we first split the dataset into two classes of equal size based on the number of atoms separated by the median, i.e., 48

**Table 20. Example prompts and completions for predicting the structure type and number of atoms of nanoparticles.** `<scattering pattern>` serves as a placeholder for the numeric string of the nanomaterial's scattering pattern.

| prompt | completion | experimental |
|---|---|---|
| `What is the structure type of <scattering pattern>*?` | 0 | Category BCC |
| `What is the structure type of <scattering pattern>?` | 1 | Category Dec |
| `What is the structure type of <scattering pattern>?` | 2 | Category FCC |
| `What is the structure type of <scattering pattern>?` | 3 | Category HCP |
| `What is the structure type of <scattering pattern>?` | 4 | Category Ico |
| `What is the structure type of <scattering pattern>?` | 5 | Category Oct |
| `What is the structure type of <scattering pattern>?` | 6 | Category SC |
| `What is the number of atoms of <scattering pattern>?` | 0 | Low |
| `What is the number of atoms of <scattering pattern>?` | 1 | High |

* Scattering pattern example: "[0 0.001 -0.003 0.005 0.022 0.055 0.064 0.076 0.084 0.091 … 0.016 0.0012 0.010 0.008 0.009 0.008 0.008]"

**Table 21. Overview of accuracy results of LLMs and "traditional" ML models for binary classification (balanced classes) of the structure type.** Three runs were performed to get the metrics average. LLMs were fine-tuned with 30 epochs and a learning rate of 0.0003. Maximum performances are highlighted in bold.

| Size | Model | Accuracy | F1 Macro | F1 Micro | Kappa |
|---|---|---|---|---|---|
| 1800 | GPT-J (LLM) | 0.94 | 0.86 | 0.94 | 0.92 |
| | Llama (LLM) | 0.94 | 0.84 | 0.94 | 0.91 |
| | **Mistral (LLM)** | **0.97** | **0.94** | **0.97** | **0.95** |
| | RF | 0.94 | 0.83 | 0.94 | 0.91 |
| | XGBoost | 0.94 | 0.88 | 0.96 | 0.94 |
| | Zero-rule | 0.50 | 0.50 | 0.50 | 0.00 |

atoms.

Figure 44 shows that the GPT-J model trained with 20 epochs gives an accuracy of 94% for a training set of 1800 data points, which is significantly better than random guess (shown by the dashed line). To check if we could improve the model's predictive performance, we did experiments with 30 epochs, and a very high precision of 98% was achieved.

We also fine-tuned three LLMs, i.e., GPT-J, Llama, and Mistral, with 30 epochs, and two "traditional" ML models, i.e., XGBoost and random forest (RF), for comparison purposes. Table 22 and Figure 45 show that very high accuracies (100%) were obtained with the Llama, RF, and XGBoost models, but only slightly lower accuracy values were obtained with GPT-J and Mistral (98% and 99%, respectively).

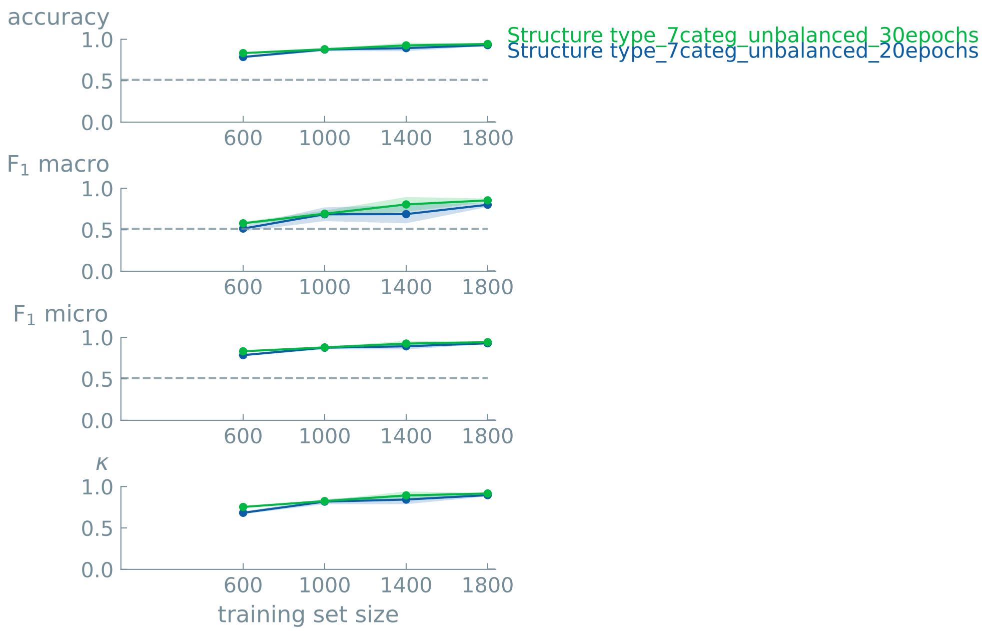


**Figure 36. Learning curves for 7-class classification GPT-J models (unbalanced classes) for the structure type fine-tuned with different number of epochs.** Data points indicate the mean value of three different experiments. Error bands show the standard error of the mean. We used 0.51 for random guess accuracy (dashed line). Accuracy = 0.943±0.003 (epochs = 30, learning rate = 0.0003, training set size = 1800 data points).

**Table 22. Overview of the accuracy results of LLMs and "traditional" ML models for binary classification (balanced classes) of the number of atoms of nanoparticles.** Three runs were performed to get the metrics average. LLMs were fine-tuned with 30 epochs and a learning rate of 0.0003. Maximum performances are highlighted in bold.

| Size | Model | Accuracy | F1 Macro | F1 Micro | Kappa |
|---|---|---|---|---|---|
| 1800 | GPT-J (LLM) | 0.98 | 0.98 | 0.98 | 0.96 |
| | **Llama (LLM)** | **1.0** | **1.0** | **1.0** | **1.0** |
| | Mistral (LLM) | 0.99 | 0.99 | 0.99 | 0.99 |
| | **RF** | **1.0** | **1.0** | **1.0** | **1.0** |
| | **XGBoost** | **1.0** | **1.0** | **1.0** | **1.0** |
| | Zero-rule | 0.50 | 0.50 | 0.50 | 0.00 |

As an example, Figure 46 shows an averaged (over three independent runs) normalized confusion matrix for the GPT-J model trained using a training set of 1800 data points and

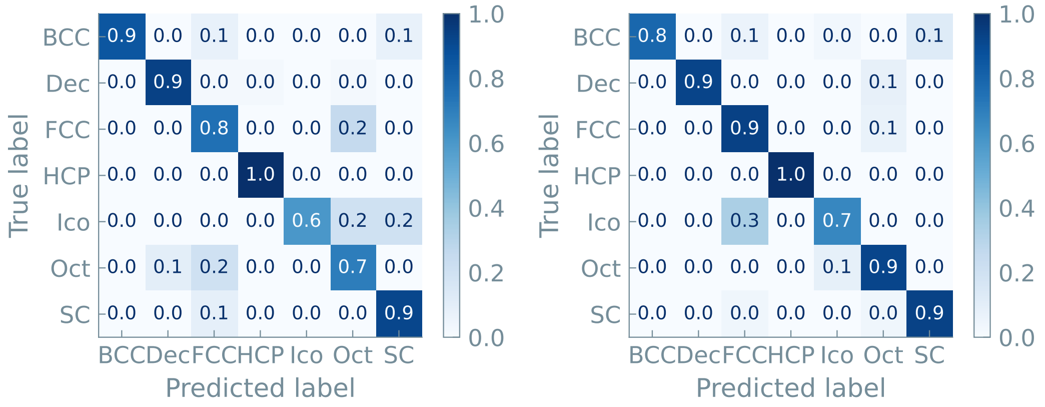


**(a)** **(b)**

**Figure 37. Normalized confusion matrix, averaged over three independent runs, on the holdout test data for structure type prediction with the GPT-J model.** Models were trained using an 'unbalanced' dataset with seven categories, a training set of 1800 data points, and 20 epochs (accuracy = 93%) (a) and 30 epochs (accuracy = 94%) (b).

30 epochs. We can see that the predictive performance of the model for binary classification of the number of atoms is very good.

**Number of atoms - Real Split** Since the binary classification of the number of atoms in nanoparticles is of no practical interest, we also trained classification models using datasets split into four and ten bins and regression models using datasets with continuous values.

To classify the dataset into a larger number of classes, we split it into four and ten equally sized bins. For four-class classification, Figure 47 shows that the GPT-J model trained with 30 epochs gives an accuracy of 90% for a training set of 1800 data points, which is much better than random guess (shown by the dashed line). This accuracy did not significantly increase when we used 60 epochs, achieving a value of 91%. For ten-class classification, Figure 48 shows that the model trained with 30 epochs achieves an accuracy of 83% for a training set of 1800 data points, which is also much better than random guess (shown by the dashed line). This accuracy slightly increased when we used 60 epochs, achieving a value of 85%.

We also fine-tuned three LLMs (GPT-J, Llama, and Mistral) and two "traditional" ML models (XGBoost and RF) for four-class and ten-class classification using 60 epochs. For four-class classification, Figure 49 shows that the highest accuracy (98%) was obtained with the Mistral model, but high accuracy values were also obtained with other models (88-91%). For ten-class classification, Figure 50 shows that the LLM models perform much better (accuracy values of 85-92%) than the "traditional" ML models (accuracy values of 76% with RF,

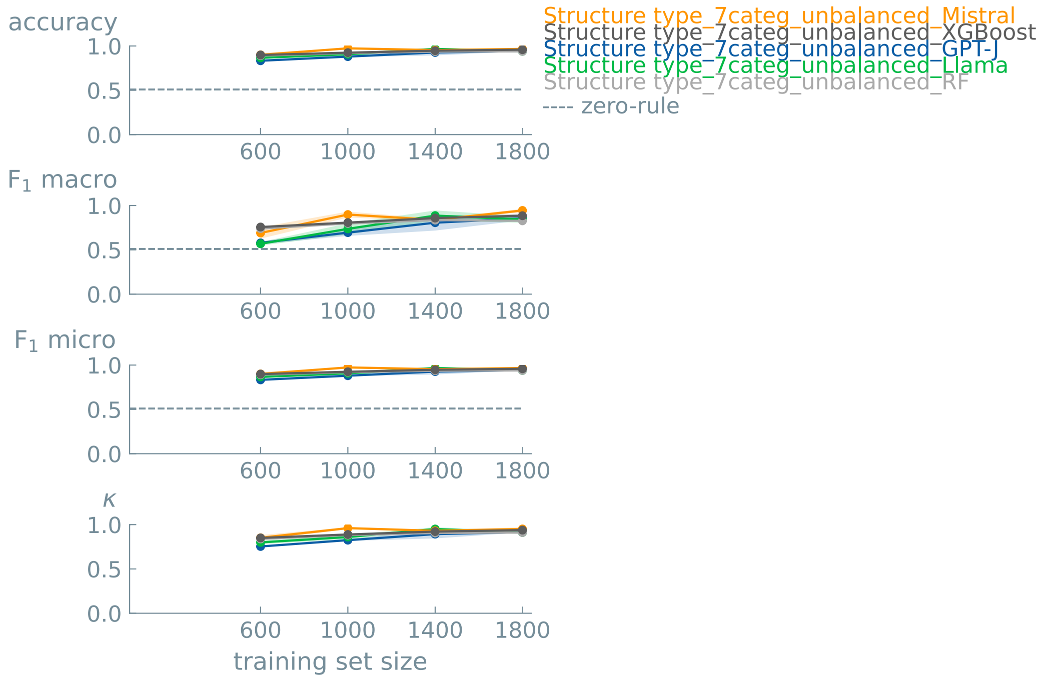


**Figure 38. Learning curves for 7-class classification models (unbalanced classes) for the structure type.** Data points indicate the mean value of three different experiments. Error bands show the standard error of the mean. We used 0.51 for random guess accuracy (dashed line). Accuracy: GPT-J=0.943±0.003, Llama=0.940±0.012, Mistral=0.967±0.013, random forest=0.939±0.003, XGBoost=0.958±0.008 (LLM epochs = 30, LLM learning rate = 0.0003, random forest and XGBoost=default parameters, training set size = 1800 data points).

and 68% with XGBoost).

As an example, Figure 51 also shows the good predictions obtained with the GPT-J model with four (Figure 51a) and ten (Figure 51b) classes in the dataset, since the majority of them are in the diagonal of the confusion matrix.

Finally, for regression, we use the regression approach of our original article,[9] i.e., direct text completion of rounded figures. In this case, a random train/test data split stratified on the target variable was applied using a threshold of 60 atoms. Figure 52 shows that the regression LLM models performs notably well in predicting the number of atoms of nanoparticles when using a training size of 1800 data points and 30 epochs ($R^2$ = 0.98-0.99, maximum absolute error (MAE) = 0.74-1.24, root mean squared error (RMSE) = 2.83-3.76). We can also see that the LLMs perform better than the “traditional” ML models for regression.

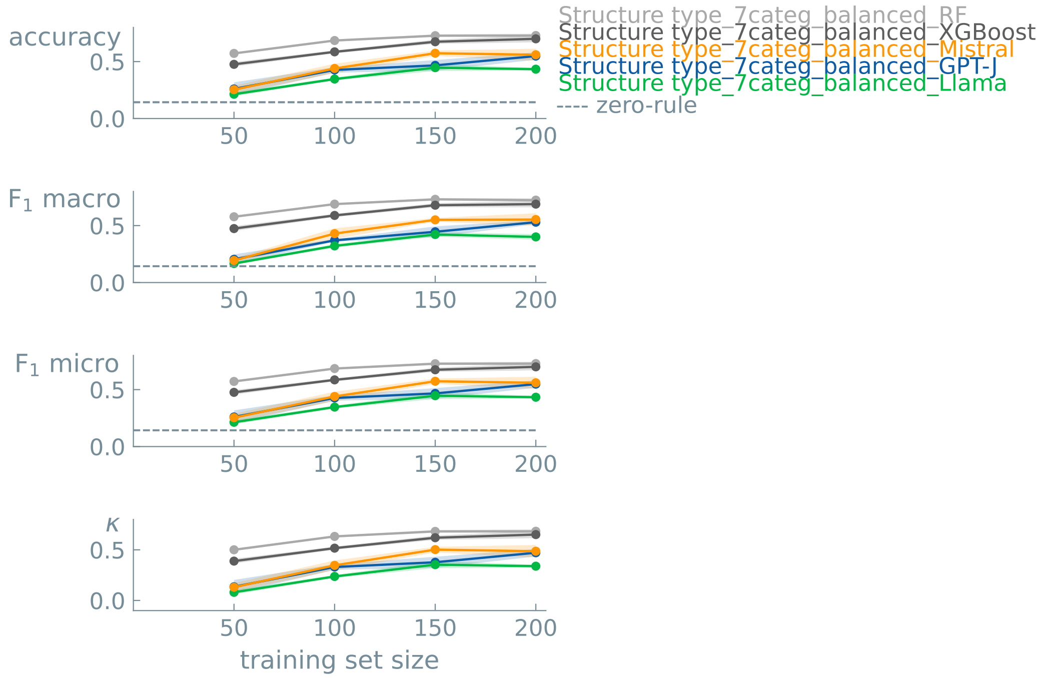


**Figure 39. Learning curves for 7-class classification models (balanced classes) for the structure type.** Data points indicate the mean value of three different experiments. Error bands show the standard error of the mean. We used 0.14 for random guess accuracy (dashed line). Accuracy: GPT-J=0.548±0.032, Llama=0.433±0.013, Mistral=0.560±0.053, random forest=0.729±0.018, XGBoost=0.700±0.030 (LLM epochs = 30, LLM learning rate = 0.0003, random forest and XGBoost=default parameters, training set size = 200 data points).

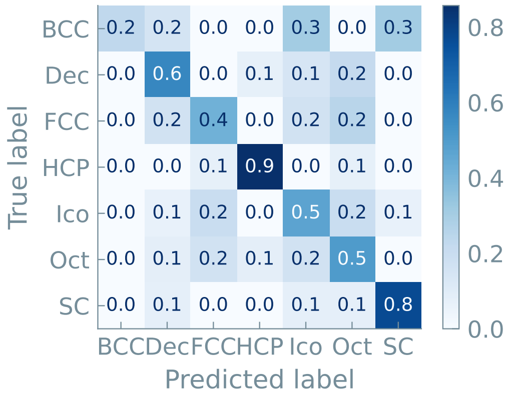


**Figure 40. Normalized confusion matrix, averaged over three independent runs, on the holdout test data for structure type prediction with the GPT-J model.** Models were trained using a 'balanced' dataset with seven categories, a training set of 200 data points, and 30 epochs (accuracy = 55%).

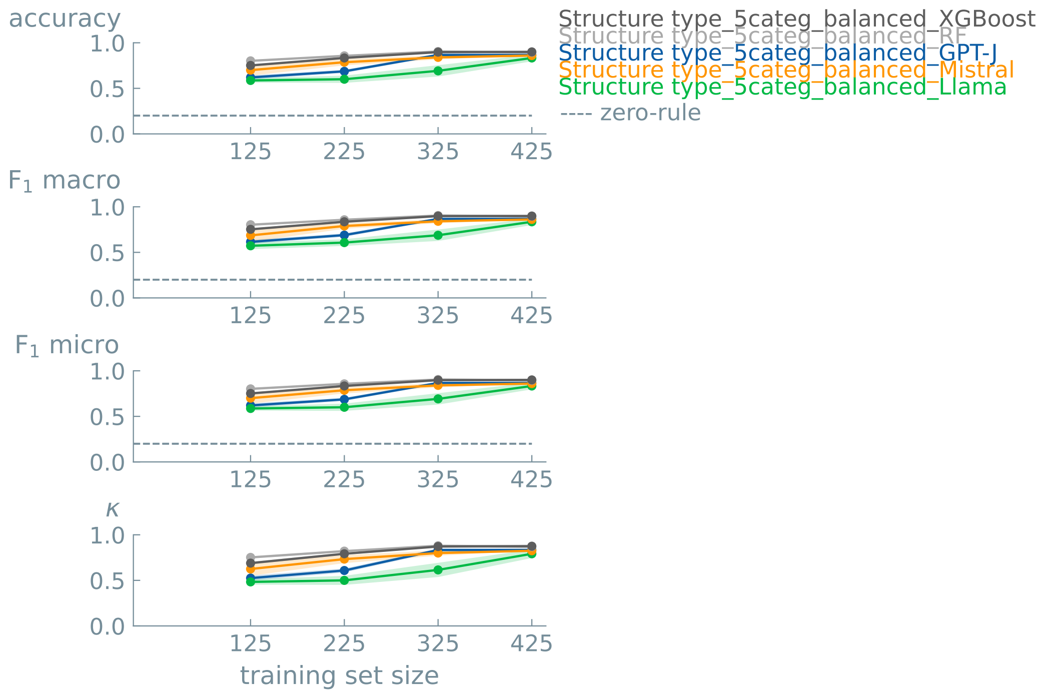


**Figure 41. Learning curves for 5-class classification models (balanced classes) for the structure type.** Data points indicate the mean value of three different experiments. Error bands show the standard error of the mean. We used 0.20 for random guess accuracy (dashed line). Accuracy: GPT-J=0.867±0.013, Llama=0.833±0.037, Mistral=0.860±0.000, random forest=0.896±0.018, XGBoost=0.900±0.014 (LLM epochs = 30, LLM learning rate = 0.0003, random forest and XGBoost=default parameters, training set size = 425 data points).

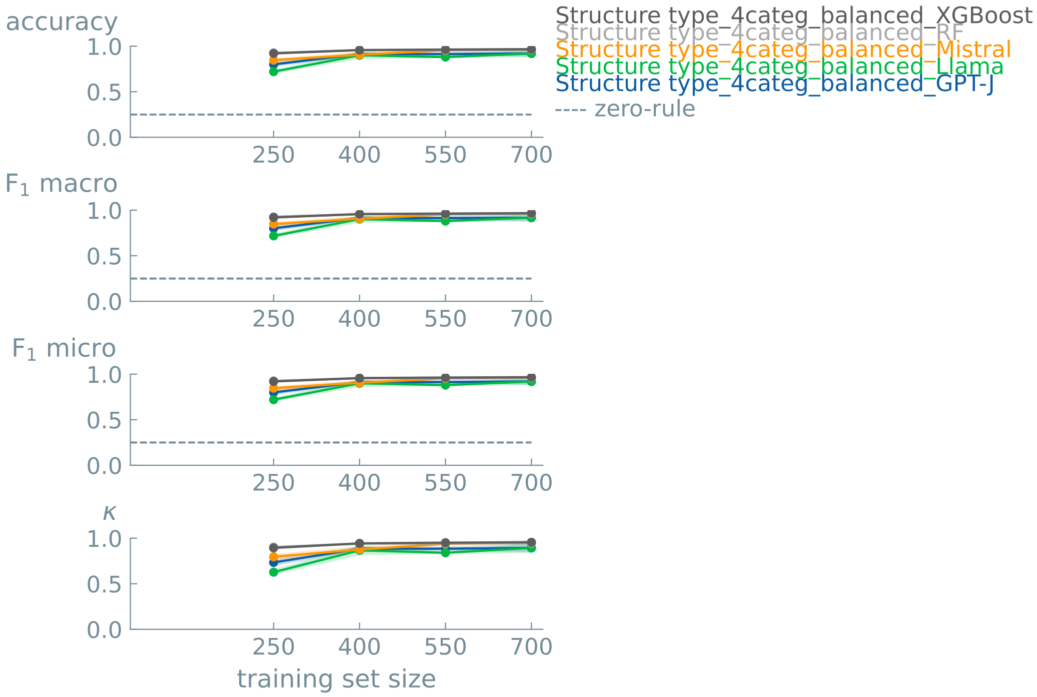


**Figure 42. Learning curves for 4-class classification models (balanced classes) for the structure type.** Data points indicate the mean value of three different experiments. Error bands show the standard error of the mean. We used 0.25 for random guess accuracy (dashed line). Accuracy: GPT-J=0.920±0.035, Llama=0.920±0.042, Mistral=0.960±0.000, random forest=0.960±0.011, XGBoost=0.967±0.014 (LLM epochs = 30, LLM learning rate = 0.0003, random forest and XGBoost=default parameters, training set size = 700 data points).

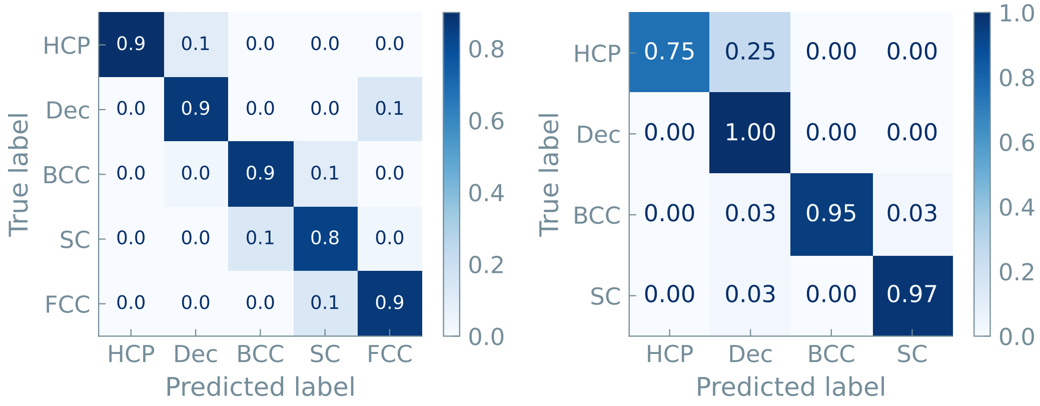


**(a)** **(b)**

**Figure 43. Normalized confusion matrix, averaged over three independent runs, on the holdout test data for structure type prediction with the GPT-J model.** Models were trained using a 'balanced' dataset with five categories, a training set of 425 data points, and 30 epochs (accuracy = 87%) (a), as well as a 'balanced' dataset with four categories, a training set of 700 data points, and 30 epochs (accuracy = 92%) (b).

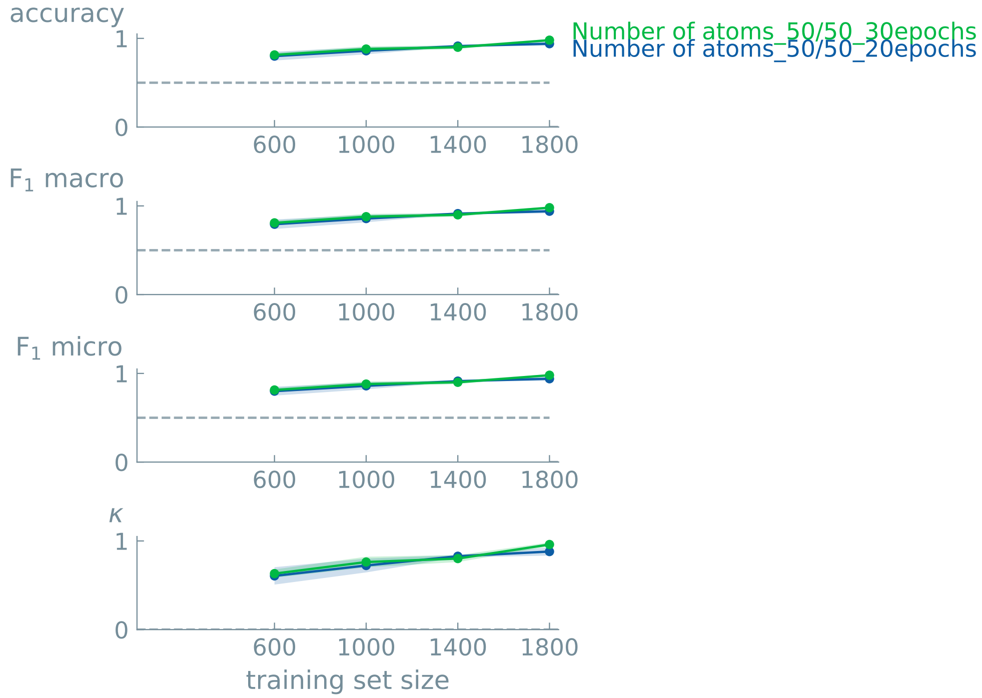


**Figure 44. Learning curves for binary classification GPT-J models (balanced classes) for the number of atoms of nanoparticles fine-tuned with different number of epochs.** Data points indicate the mean value of three different experiments. Error bands show the standard error of the mean. We used 0.50 as random guess accuracy (dashed line), representing the zero rule baseline, i.e., a model that always predicts the most common class. Accuracy = 0.980±0.012 (epochs = 30, learning rate = 0.0003, training set size = 1800 data points).

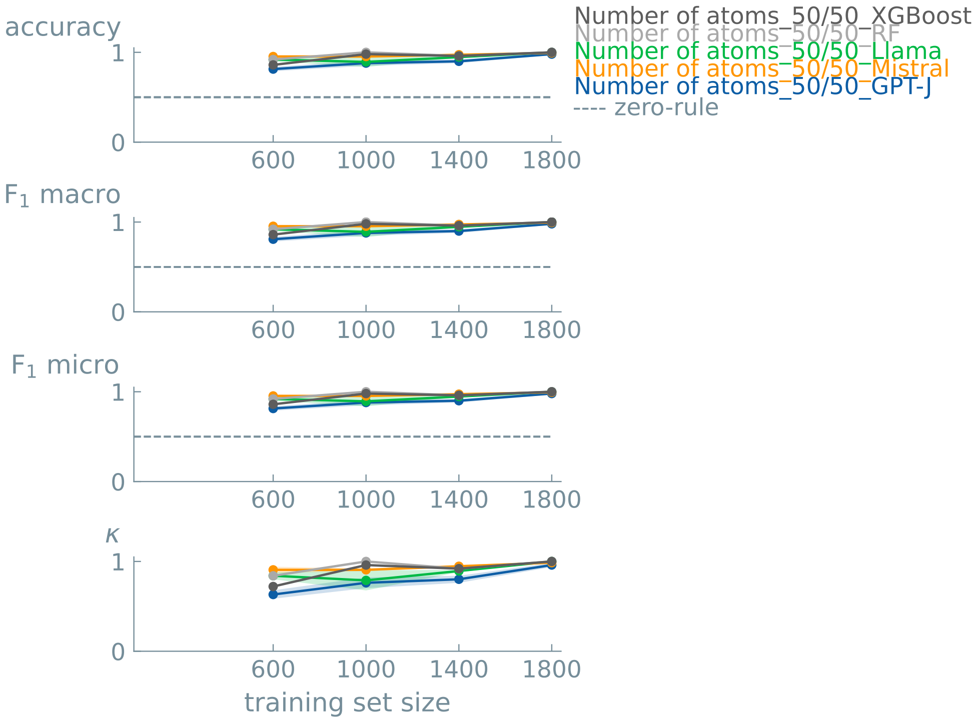


**Figure 45. Learning curves for binary classification models (balanced classes) for the number of atoms of nanoparticles.** Data points indicate the mean value of three different experiments. Error bands show the standard error of the mean. We used 0.50 as random guess accuracy (dashed line), representing the zero rule baseline, i.e., a model that always predicts the most common class. Accuracy: GPT-J=0.980±0.012, Llama=1.0±0.0, Mistral=0.993±0.007, random forest=1.0±0.0, XGBoost=1.0±0.0 (LLM epochs = 30, LLM learning rate = 0.0003, random forest and XGBoost=default parameters, training set size = 1800 data points).

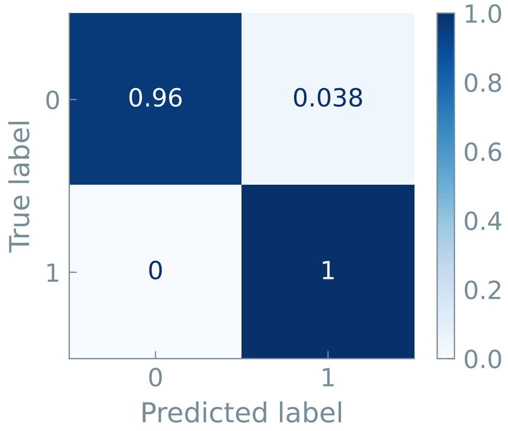


**Figure 46. Normalized confusion matrix, averaged over three independent runs, on the holdout test data for the number of atoms prediction with the GPT-J model.** Models were trained for binary classification using a training set of 1800 data points and 30 epochs (accuracy = 98%).

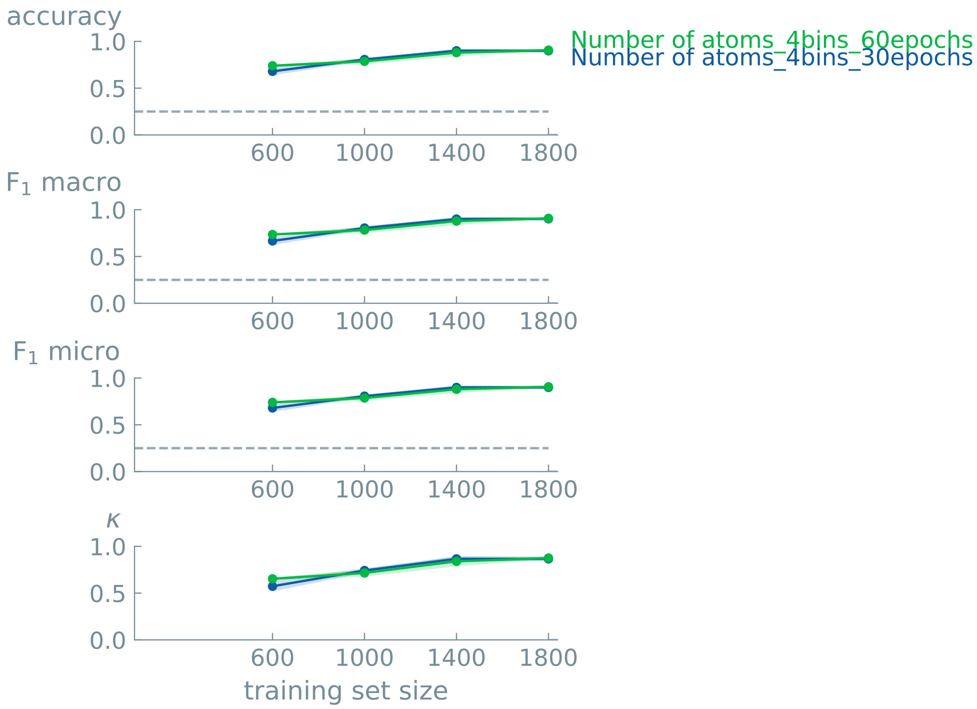


**Figure 47. Learning curves for 4-class classification GPT-J models for the number of atoms of nanoparticles.** Data points indicate the mean value of three different experiments. Error bands show the standard error of the mean. We used 0.25 as random guess accuracy (dashed line), representing the zero rule baseline, i.e., a model that always predicts the most common class. Accuracy = 0.907±0.007 (epochs = 60, learning rate = 0.0003, training set size = 1800 data points).

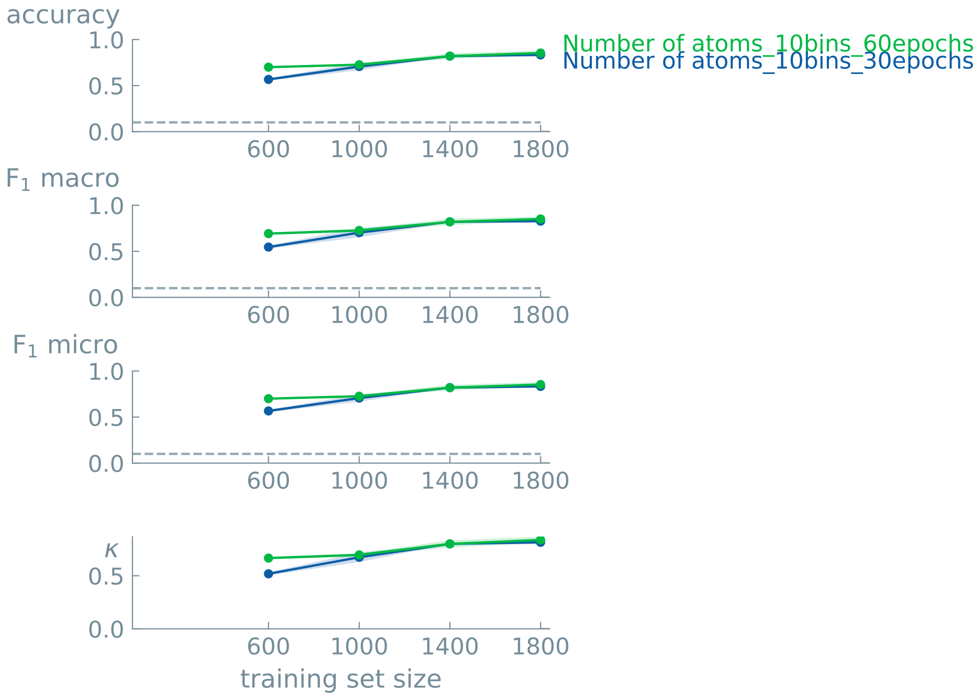


**Figure 48. Learning curves for 10-class classification GPT-J models for the number of atoms of nanoparticles.** Data points indicate the mean value of three different experiments. Error bands show the standard error of the mean. We used 0.10 as a random guess accuracy (dashed line), representing the zero rule baseline, i.e., a model that always predicts the most common class. Accuracy = 0.853±0.027 (epochs = 60, learning rate = 0.0003, training set size = 1800 data points).

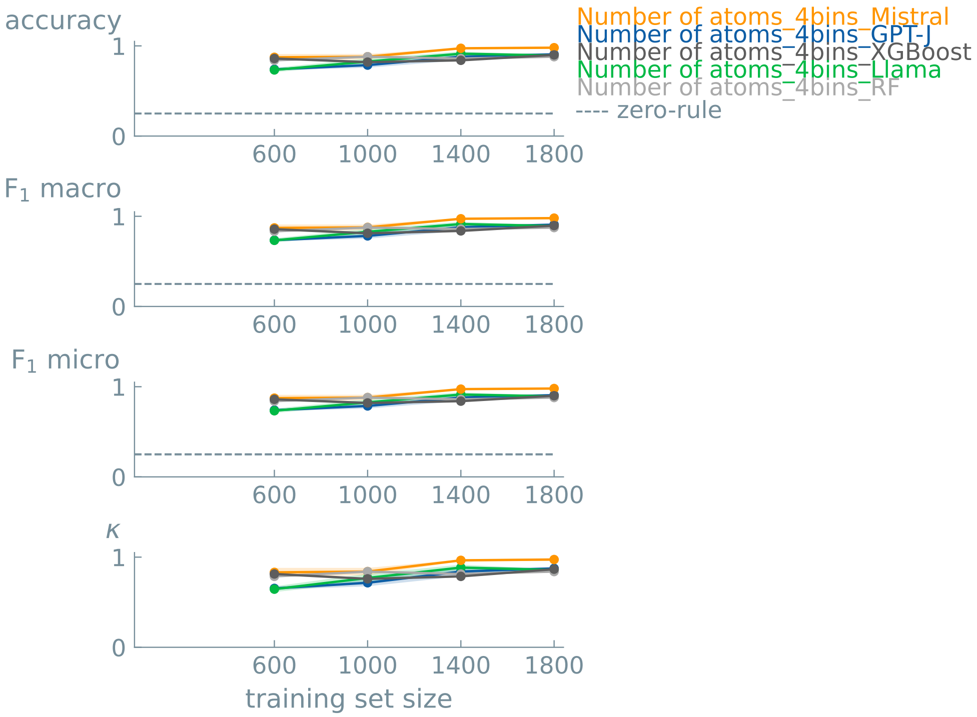


**Figure 49. Learning curves for 4-class classification models for the number of atoms of nanoparticles.** Data points indicate the mean value of three different experiments. Error bands show the standard error of the mean. We used 0.25 as random guess accuracy (dashed line), representing the zero rule baseline, i.e., a model that always predicts the most common class. Accuracy: GPT-J=0.907±0.007, Llama=0.893±0.007, Mistral=0.980±0.011, random forest=0.880±0.000, XGBoost=0.900±0.000 (LLM epochs = 60, LLM learning rate = 0.0003, random forest and XGBoost=default parameters, training set size = 1800 data points).

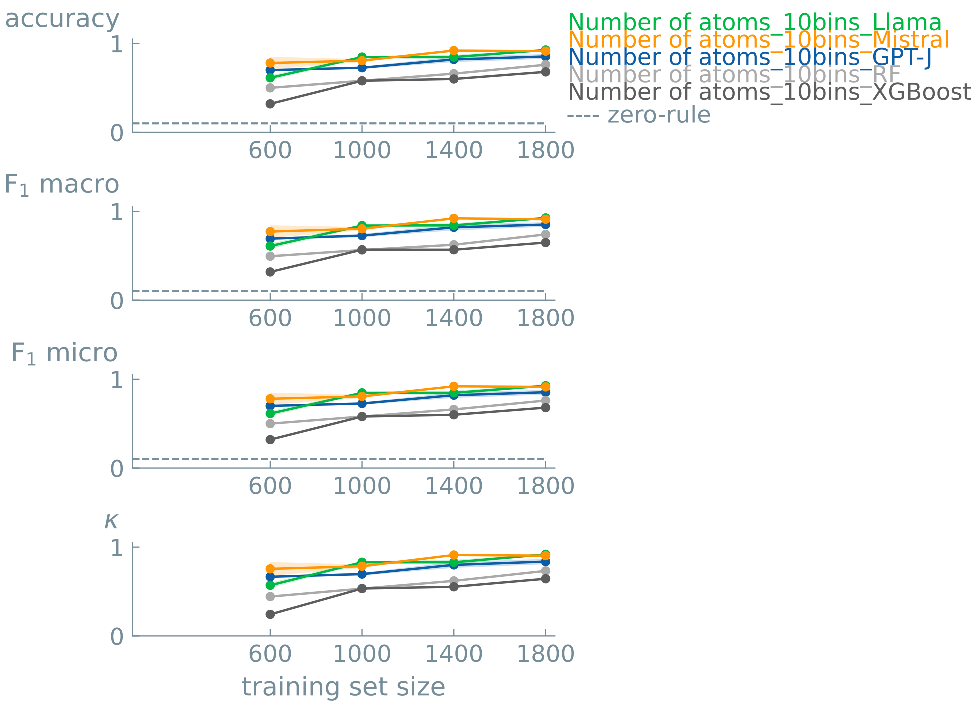


**Figure 50. Learning curves for 10-class classification models for the number of atoms of nanoparticles.** Data points indicate the mean value of three different experiments. Error bands show the standard error of the mean. We used 0.10 as a random guess accuracy (dashed line), representing the zero rule baseline, i.e., a model that always predicts the most common class. Accuracy: GPT-J=0.853±0.027, Llama=0.927±0.007, Mistral=0.913±0.007, random forest=0.760±0.000, XGBoost=0.680±0.000 (LLM epochs = 60, LLM learning rate = 0.0003, random forest and XGBoost=default parameters, training set size = 1800 data points).

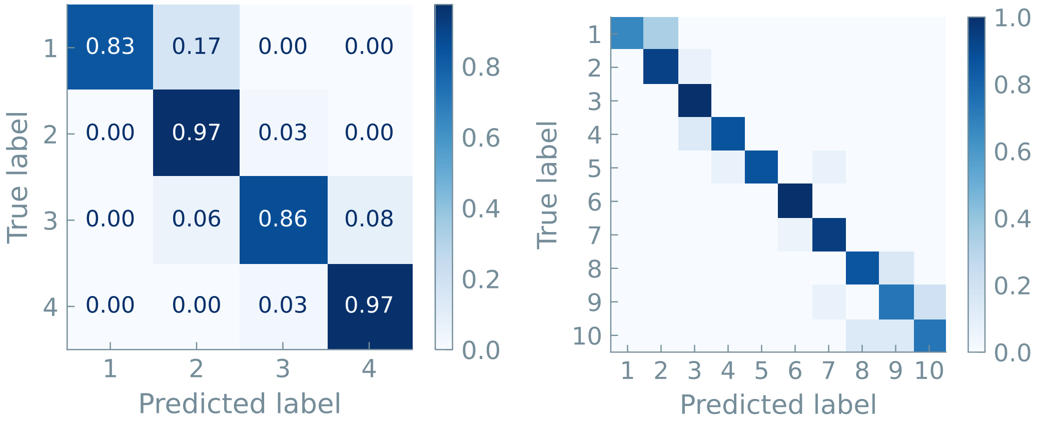


**(a)** **(b)**

**Figure 51. Normalized confusion matrix, averaged over three independent runs, on the holdout test data for the number of atoms of nanoparticles prediction with the GPT-J model.** Models were trained using 4-class (accuracy = 91%) (a) and 10-class (accuracy = 85%) (b) balanced datasets, training sets of 1800 data points, and 60 epochs.

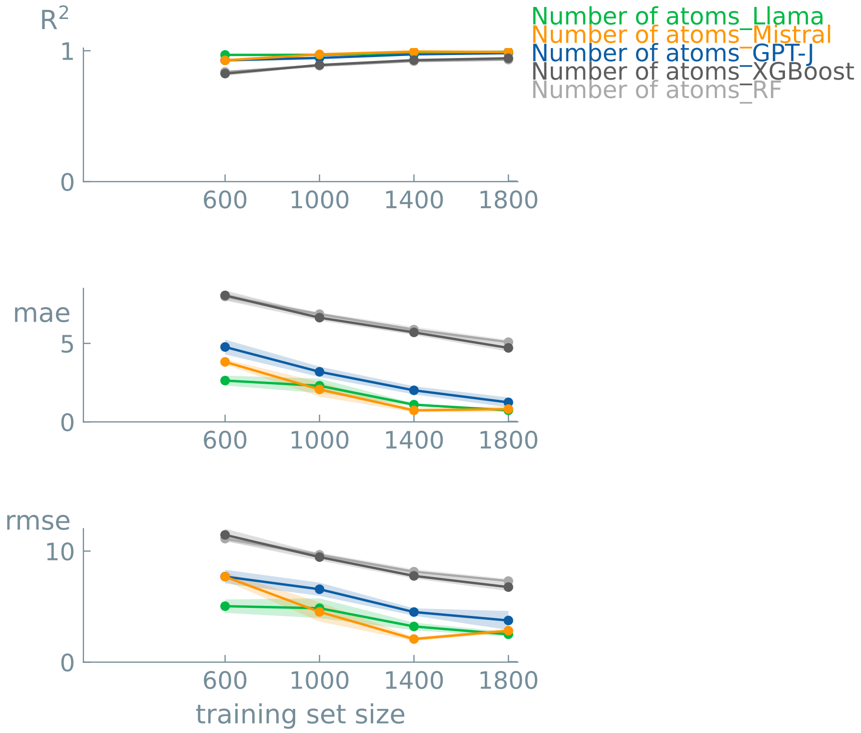


**Figure 52. Learning curves for regression models for the prediction of the number of atoms of nanoparticles.** Data points indicate the mean value of three different experiments. Error bands show the standard error of the mean. $R^2$: GPT-J=0.982±0.007, Llama=0.993±0.001, Mistral=0.991±0.001, random forest=0.932±0.003, XGBoost=0.942±0.006; MAE: GPT-J=1.24±0.31, Llama=0.74±0.02, Mistral=0.82±0.19, random forest=5.08±0.15, XGBoost=4.71±0.23; RMSE: GPT-J=3.76±0.85, Llama=2.51±0.21, Mistral=2.83±0.17, random forest=7.30±0.22, XGBoost=6.76±0.36 (LLM epochs = 30, LLM learning rate = 0.0003, random forest and XGBoost=default parameters, training set size = 1800 data points).

## 3.8 Melting Temperature of triacylglycerols

The dataset was provided by: Michele Lessona, Antonio Buffo and Elena Simone[8]

### 3.8.1 Scientific Background

Triacylglycerols (TAGs) are the primary components of natural fats and oils. Fats and oils are of crucial importance in food, cosmetic, and pharmaceutical applications. Natural fats are mixtures of several TAGs composed of different Fatty Acids (FAs), which vary in unsaturation level, chain length, and their relative position on the glycerol backbone. These variations affect both the thermal and structural properties of TAGs and, in turn, their behavior in solid fat mixtures. To predict how complex TAG mixtures crystallize and melt, different thermodynamic models based on the thermal properties of pure TAGs have been developed.[43–45] However, due to the chemical complexity of natural fats and the structural similarity of many natural TAG molecules, these are often difficult to extract and purify, meaning that the experimental data necessary to build thermodynamic models of TAG mixtures is not always available. Over the years, many predictive models for the estimation of melting temperature and enthalpy of pure TAGs[43,46–48] have been developed.

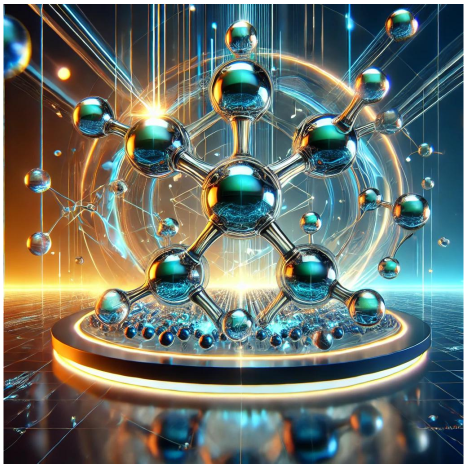

**Figure 53.** AI generated representation of the melting temperature of triacylglycerols.

### 3.8.2 Dataset

Literature data on the melting point and enthalpy of a wide selection of pure TAGs in their β-polymorph (the most thermodynamically stable form) were also collected and deposited in a publicly available database.[49] Nevertheless, the lack of reliable, pure TAG melting properties remains a key issue in lipid science. For this work, 211 TAGs were selected and represented using the commonly employed three-letter code (e.g., SSS POS, POP), with each letter corresponding to an FA. The TAGs were further identified by their common and IUPAC name, the omega and the delta nomenclature (which is very common for FAs), and their SMILES and InChi molecular descriptors. The melting temperature was found in the aforementioned experimental database.[49]

For every TAG, different representations were given:

- **Name** - Three letter name

[8] Department of Applied Science and Technology (DISAT) , Politecnico di Torino, 10129 Turino, Italy

- **iupac** - The proper IUPAC name of the TAG
- **iupac-common name** - The common name of the TAG
- **omega** - The omega notation of the TAG
- **delta** - The delta notation of the TAG
- **InChI** - The InChI notation of the TAG
- **SMILES** - The SMILES notation of the TAG

In addition, two physical properties of the TAGs were included:

- **melting enthalpy** - in $kJ\,mol^{-1}$
- **melting temperature** - in °C

The distribution of the melting temperature values in the dataset is shown in Figure 54.

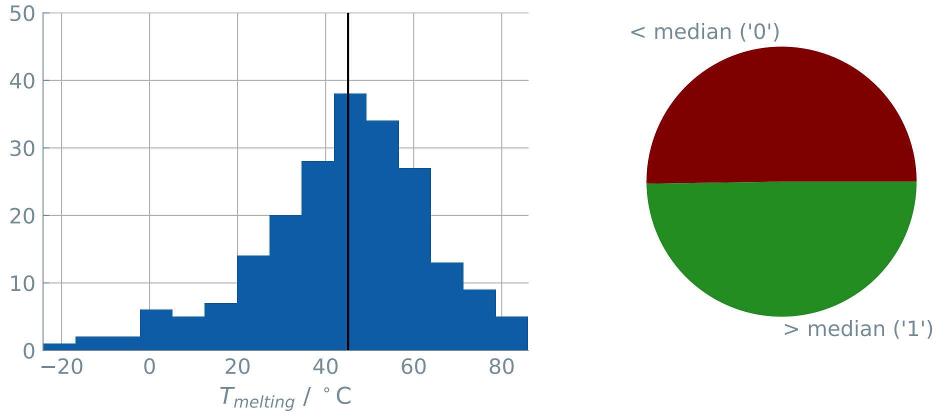


**Figure 54. Distribution of the melting temperature.** Left: The black line in the histogram represents the median melting temperature of 45.1 °C (n = 211). Right: A binary classification based on the median melting temperature was created.

We used a simple prompt template shown in Table 23 for experiments to predict the melting temperature of TAGs.

### 3.8.3 LLM Results

**Base Case** For the binary classification models, we split the dataset into two equally sized classes based on their melting point values. Entries with values higher than the median

**Table 23. Example prompts and completions for predicting the melting point of triacylglycerols (TAG).** `<TAG>` is the placeholder for the various TAG representations used.

| prompt | completion | experimental |
|---|---|---|
| Example of training data | | |
| `What is the melting point of <TAG>?` | 0 | Low |
| `What is the melting point of <TAG>?` | 1 | High |

of 45.1 °C are labeled "1", and entries with lower values are labeled "0". We performed a learning curve analysis on this binary classification. The number of test data remained constant over all runs, i.e., 50. The number of epochs was set to 25. Three unique runs with the GPT-J model were performed for every pair of training size/epoch experiments to get the average metrics. As the dataset is balanced, we can assume an accuracy of 50% as the zero-rule baseline, i.e., random guessing.

Firstly, we compared the influence of different representations of the TAGs on the performance of fine-tuning the GPT-J model (Figure 55). We do not see a significant difference in performance for training set sizes above 100, i.e., accuracies within 2% of each other. We do see subtle differences on the lower end of the plot, i.e., training set size of 10, where the InChI notation (accuracy of 88%) and SMILES notation (accuracy of 81%) perform better than the IUPAC name (accuracy of 60%).

Apart from the representation, we validated various base models. Three LLMs. i.e., GPT-J, Llama, and Mistral, were fine-tuned. We notice that all models show similar predictive performance. A maximum accuracy of 92% was reached with the GPT-J model (Figure 56 and Table 24). In addition, the fine-tuned LLMs were compared with "traditional" ML models (XGBoost and random forest (RF)). We can conclude that LLMs can, in general, compete with, and even outperform, these models.

**Table 24. Overview of results of LLMs and "traditional" ML predicting the binary class of the melting point.** Three runs were performed to get the metrics average. All LLMs were fine-tuned with 25 epochs and a learning rate of 0.003. For the random forest (RF) and XGBoost models, optuna was used for hyperparameter optimization. Maximum performances are highlighted in bold.

| Size | Model | Accuracy | F1 Macro | F1 Micro | Kappa |
|---|---|---|---|---|---|
| 150 | **GPT-J (LLM)** | **0.92** | **0.92** | **0.92** | **0.84** |
| | **Llama (LLM)** | **0.92** | **0.92** | **0.92** | **0.84** |
| | Mistral (LLM) | 0.87 | 0.87 | 0.87 | 0.75 |
| | RF | 0.86 | 0.86 | 0.86 | 0.72 |
| | XGBoost | 0.88 | 0.88 | 0.88 | 0.77 |
| | Zero-rule | 0.50 | 0.50 | 0.50 | 0.00 |

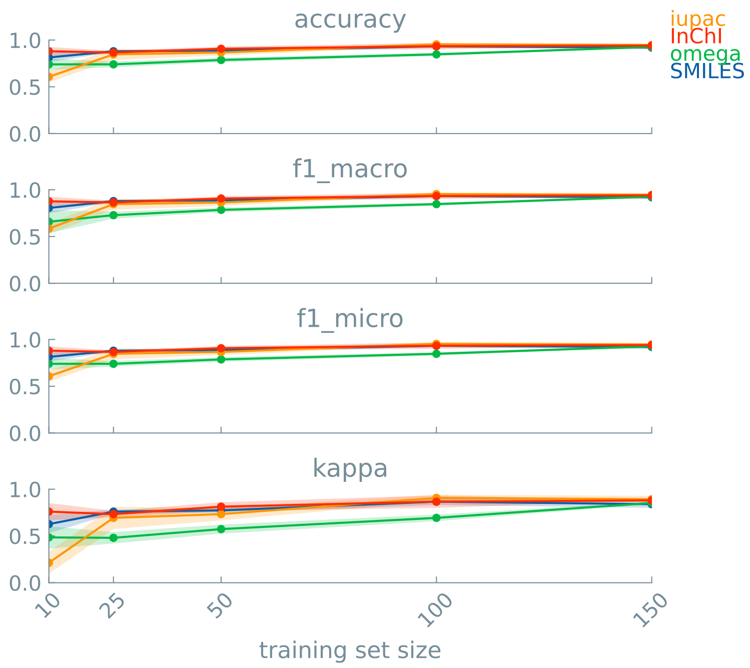


**Figure 55. Learning curve analyses of binary classification of the melting point using different representations.** Four representations of the TAGs (IUPAC, InChI, omega notation, and SMILES) were validated in predicting the binary class of the melting point of TAGs by fine-tuning the GPT-J model. Three runs were performed for each model to get the metric's average and standard deviation. The fine-tuned model trained on the IUPAC name reached the maximum accuracy of 95% (training set size of 150 and 25 epochs).

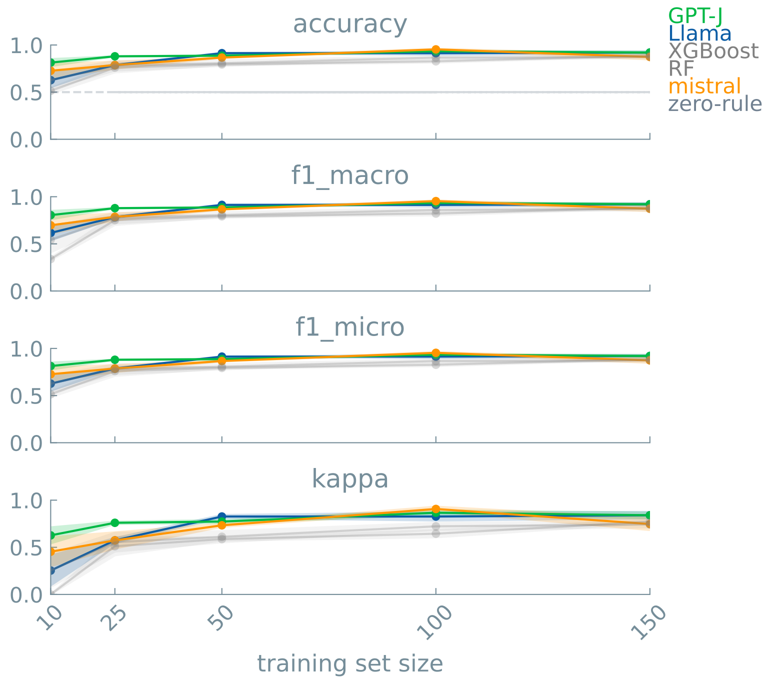


**Figure 56. Learning curve analyses of binary classification of the melting point using different models.** Three LLMs (GPT-J, Llama, and Mistral) and two traditional ML models (XGBoost and random forest (RF)) were validated on predicting the binary class of the melting point of TAGs. We used 50% as a random guess accuracy (dashed line), representing the zero rule baseline. Three runs were performed for each model to get the metric's average and standard deviation. SMILES of the TAGs were used as the input for the LLMs. Morgan Fingerprints of the TAGs were used as the input for the traditional ML models. The fine-tuned model reached the maximum accuracy of 92% (training set size of 150 and 25 epochs).

# 4 Reactions and Synthesis

This section describes case studies regarding reactions and synthesis procedures.

## 4.1 Activation Energy of Cycloadditions

The dataset was provided by: Dennis Svatunek[9]

### 4.1.1 Scientific Background

Click chemistry comprises chemical reactions that are highly efficient and selective.[50] These properties make them interesting for bioorthogonal chemistry, i.e., *in vivo* reactions that do not alter the native biochemical reactions. Among the chemical transformations, Diels-Alder reactions hold significant prominence. Notably, the cycloaddition involving 1,2,4,5-tetrazines and strained alkenes is a well-established system in this context. Nevertheless, their reaction kinetics are not fully understood. An interesting observation is that substitutes on the 3- and 6-positions of the tetrazine do affect the reaction rate significantly.

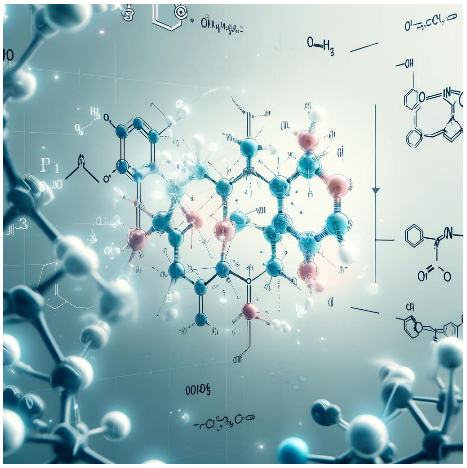

**Figure 57.** AI generated representation of the concept of click chemistry and Diels-Alder reactions.

A recent study by Houszka et al.[51] shed light on the effect of these substituents on the reactivity. They calculated the activation energy for tetrazine-alkene cycloadditions with various chemical functionalities on the 3- and 6- positions of the tetrazine, $R^1$ and $R^2$). These calculations showed that Frontier Molecular Orbital (FMO) interactions are not always the primary driving force. Also, lower distortion energies and increased electrostatic attraction have an influence (for a detailed discussion, see Houszka et al.[51]). Following this report, Svatunek recently published a dataset of reaction barriers for over 1,000 tetrazine derivatives to enable a systematic investigation into substituent effects.[52]

We were interested in whether we could predict the activation energy solely from the chemical formula with the help of a Large Language Model (LLM).

To represent the molecule, we used the Simplified Molecular Input Line-Entry System (SMILES) notation, which captures the atomic composition, bonds, branches, aromaticity, and stereochemistry. Their seamless integration into conventional machine learning meth-

[9]Institute of Applied Synthetic Chemistry, TU Wien, Getreidemarkt 9, 1060, Vienna, Austria

ods is somehow restricted by their non-numeric value and variable string length. In contrast, the inherent nature of LLMs allows textual input, regardless of its length.

### 4.1.2 Dataset

The dataset contains 966 molecules with their respective DFT-calculated barrier heights (free energy of activation in kcal $mol^{-1}$) for bioorthogonal click reactions. One reaction partner is kept the same (*trans*-cyclooctene), while substituents on the second (1,2,4,5-tetrazine) are varied. All molecules are represented by their SMILES notation. For the binary classification models, the dataset was split into two equally sized bins. Here, the threshold was the median of the free energy of activation, i.e., 14.35 kcal $mol^{-1}$. The distribution of the free energies of activation is shown in Figure 58.

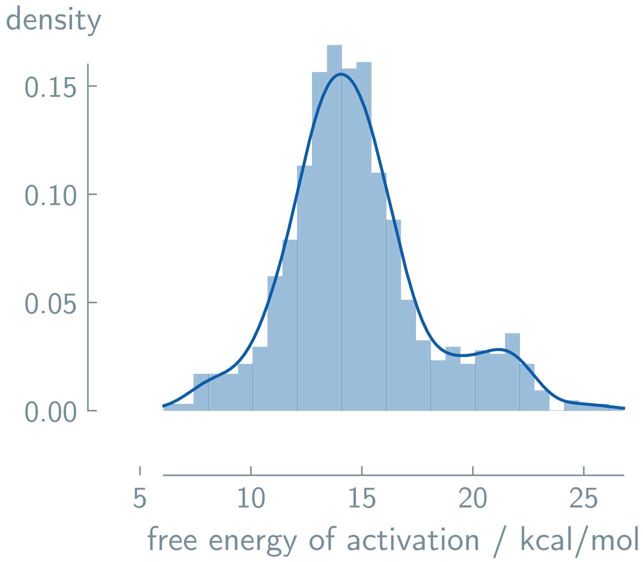


**Figure 58. Distribution of activation barrier heights in the dataset.** The median of free energy of activation is 14.35 kcal $mol^{-1}$.

Table 25 shows an example of the prompts we use to fine-tune our LLM model.

### 4.1.3 LLM results

**Base Case** As an initial test, we split our data set into two equally sized classes based on their free energy of activation value. Entries with values higher than the median of 14.35 kcal $mol^{-1}$ are labeled '1', and entries with lower values are labeled '0'. We performed a learning curve analysis for this binary classification of the free energy of activation. We used the SMILES notation to represent the tetrazine-molecule, containing different substituents

**Table 25. Example prompts and completions for predicting the free energy of activation of tetrazines.** ΔE refers to the free energy of activation. `<SMILES>` serves as a placeholder for the SMILES notation for the tetrazine.

| prompt | completion | experimental |
|---|---|---|
| Example of training data | | |
| `What is the ΔE of <SMILES>?` | 0 | Low |
| `What is the ΔE of <SMILES>?` | 1 | High |

on the $R^1$ and $R^2$ position. As the total dataset contained 966 entries, our maximum number of training data was set to 500. The number of test data remained constant over all runs, i.e., 50.

We first screened the number of epochs; 5, 15, and 25. Three unique runs were performed for every pair of training size-epoch experiments to get the average metrics (Figure 59). The GPT-J model was used for this screening.

We observed that the accuracy converges after a training set size of 250 and 15 epochs. Since we are dealing with binary classification of a balanced dataset, all accuracies above 50% are an improvement to random guessing. An accuracy of 94% clearly suggests that our GPT models perform well for this binary classification problem.

In a next step, we screened different models. Three LLMs, i.e., GPT-J, Llama, and Mistral, were fine-tuned. We notice that a maximum accuracy of 94% was reached with the GPT-J model (Figure 60 and Table 26). In addition, the fine-tuned LLMs were compared with "traditional" ML models (XGBoost and random forest (RF)). For these experiments, the SMILES notations were converted Morgan fingerprints. We see that those two models slightly outperform the Llama and Mistral models. However, we can still conclude that LLMs can in general compete with these models.

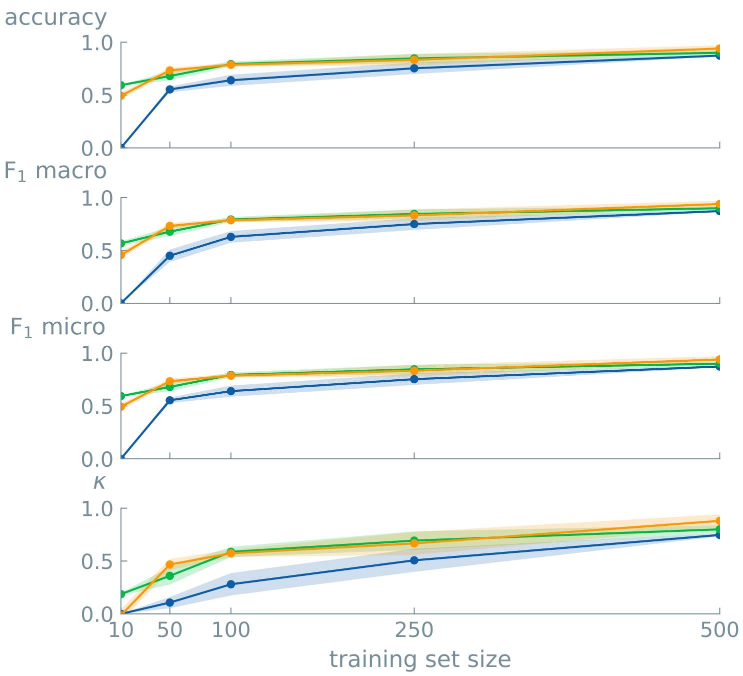


**Figure 59. Learning curve analysis for predictions on the free energy of activation of the bioorthogonal click reaction.** The blue, green, and orange lines represent 5, 15, and 20 epochs, respectively. Three separate models (GPT-J) were trained and tested, where the average and the standard deviation were plotted. A maximum accuracy of 94% was reached for a training set size of 500 and 25 epochs.

**Table 26. Overview of results of LLMs and "traditional" ML predicting the binary class of the activation energy.** Three runs were performed to get the metrics average. All LLMs were fine-tuned with 25 epochs and a learning rate of 0.003. For the random forest (RF) and XGBoost models, optuna was used for hyperparameter optimization. Maximum performances are highlighted in bold.

| Size | Model | Accuracy | F1 Macro | F1 Micro | Kappa |
|---|---|---|---|---|---|
| 500 | **GPT-J (LLM)** | **0.94** | **0.94** | **0.94** | **0.88** |
| | Llama (LLM) | 0.85 | 0.85 | 0.85 | 0.69 |
| | Mistral (LLM) | 0.88 | 0.88 | 0.88 | 0.76 |
| | RF | 0.89 | 0.89 | 0.89 | 0.79 |
| | XGBoost | 0.91 | 0.91 | 0.91 | 0.74 |
| | Zero-rule | 0.50 | 0.50 | 0.50 | 0.00 |

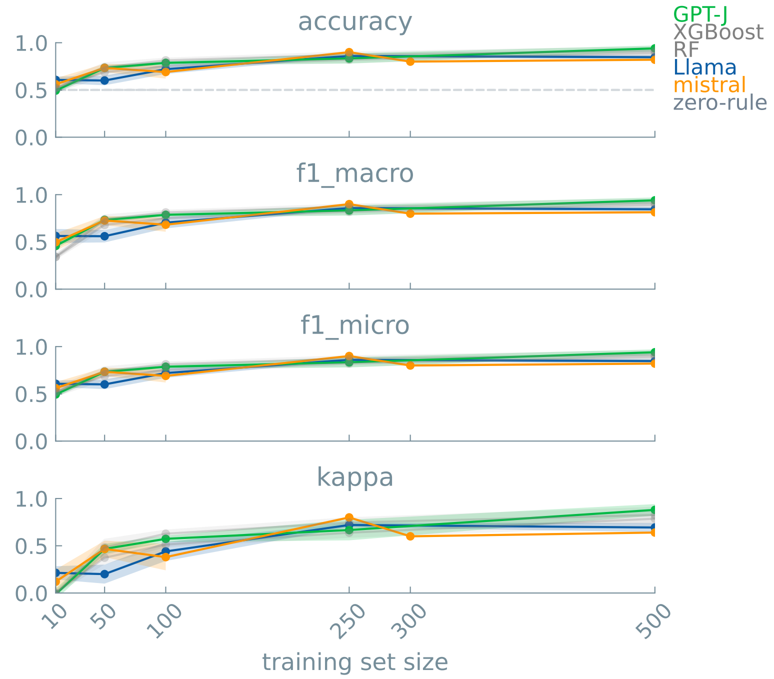


**Figure 60. Learning curve analyses of binary classification of the adhesive free-energy using different models.** Three base LLMs (GPT-J, Llama, and Mistral) and two traditional ML models (XGBoost and random forest (RF)) were validated on predicting the binary class of the free energy of activation of the bioorthogonal click reaction. We used 50% as a random guess accuracy (dashed line), representing the zero rule baseline. Three runs were performed for each model to get the metric's average and standard deviation. The fine-tuned GPT-J model reached the maximum accuracy of 94% (training set size of 500 and 25 epochs).

## 4.2 Free Energy of Catalyzed Cleavage Reaction

The dataset was provided by: Rubén Laplaza and Clemence Corminboeuf[10]

### 4.2.1 Scientific Background

Catalysis plays a crucial part in the chemical industry. Choosing the correct catalyst is often a complex task as it needs to balance between high reaction yields and low costs. A computationally efficient way of predicting the performance of a catalyst is with the help of linear scaling relationships.[53] Here, rather than computing the full free energy profile, only one value of an intermediate or transition state is used to establish linear correlations for the remaining free energies. These relationships can then be translated to a so-called volcano plot to visually compare the performance of the catalyst.[54]

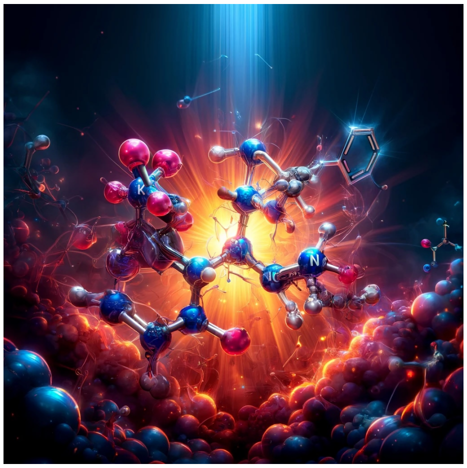

**Figure 61.** AI generated representation of a $C(sp^2)$–O cleavage reaction.

In this study, the reductive $C(sp^2)$–O cleavage reaction in an aryl ether compound was examined (more specifically, the reductive deoxygenation of 2-methoxynaphthalene with trimethylsilane).[55] This reaction is particularly relevant in the degradation of lignin into its smaller building blocks. The traditional two-step process is, however, ecologically and atom-economically unfriendly. Alternatively, a selective catalytic cleavage of the ether group from the arene moiety could be exploited (Figure 62). Two types of nickel catalysts have been proposed to facilitate the reaction: nickel catalysts bearing a phosphine and N-heterocyclic carbene ligand.

Our goal was to predict the catalytic activity of nickel-containing molecules for the aryl ether cleavage reaction. We determined the success of the catalysis based on the relative free energy of one specific intermediate ($\Delta G_{RRS}(4)$, see Cordova et al.[55] for details on the catalytic cycle). A dataset of 143,000 nickel complexes with their respective calculated free energy of intermediate 4 was created. LLMs were trained to predict this free energy from various molecular representations.

### 4.2.2 Dataset

As discussed above, the free energy of intermediate 4 of the catalysis cycle was our property of interest. All catalysts were nickel complexes with either phospines or N-heterocyclic carbenes as ligands. Substituents on the R/R'/R" positions were altered between 68 different

[10]Laboratory for Computational Molecular Design (LCMD), Institute of Chemical Sciences and Engineering (ISIC), École Polytechnique Fédérale de Lausanne (EPFL), CH-1015 Lausanne, Switzerland

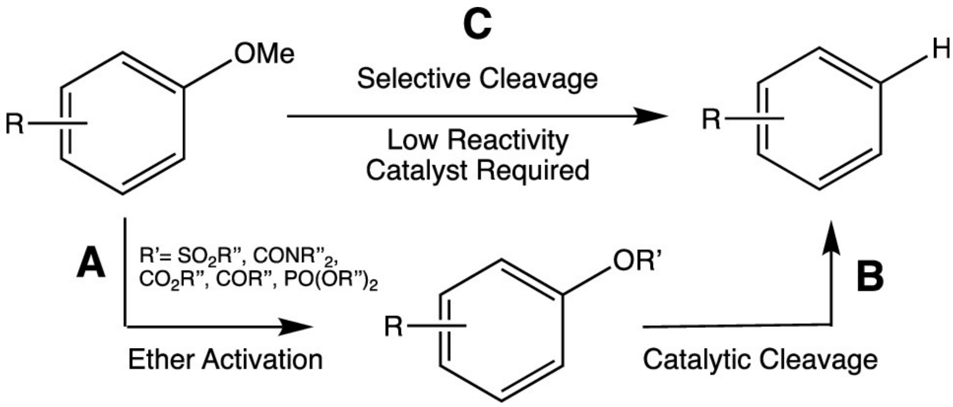


**Figure 62. Possible Routes to Aryl Ether Cleavage.** The current study examined if the performance of the catalyst for route C can be predicted from the SMILES notation of the catalyst. Adapted from Cordova et al.[55].

chemical fragments for the phospohine ligands, resulting in 54,740 unique molecules. For the carbenes, a similar combinatorial type placement for three groups resulted in 90,000 unique carbene ligands. DFT calculations were used to calculate the free energy of intermediate 4 relative to the initial energy at the beginning of the cycle ($\Delta G_{RRS}(4)$, see Cordova et al.[55] for details on the simulations).

As for the molecular representation of the catalysts, all substituents were converted to their SMILES string and combined to obtain the final structure. A subset of the full dataset was used to validate our LLM approach. The total number of entries was 1,423, with 770 species containing phosphine ligands and 653 containing carbenes ligands. The distribution of the free energy values of intermediate 4 of the catalysis cycle is shown in Figure 58.

Table 27 shows an example of the prompt we use to fine-tune our LLM model.

**Table 27. Example prompts and completions for predicting the relative free energy of intermediate 4 for the Ni-catalyzed reaction.** <SMILES> serves as a placeholder for the SMILES notation for the nickel-containing molecules.

| prompt | completion | experimental |
|---|---|---|
| Example of training data | | |
| `What is the relative free energy of <SMILES>?` | 0 | Low |
| `What is the relative free energy of <SMILES>?` | 1 | High |

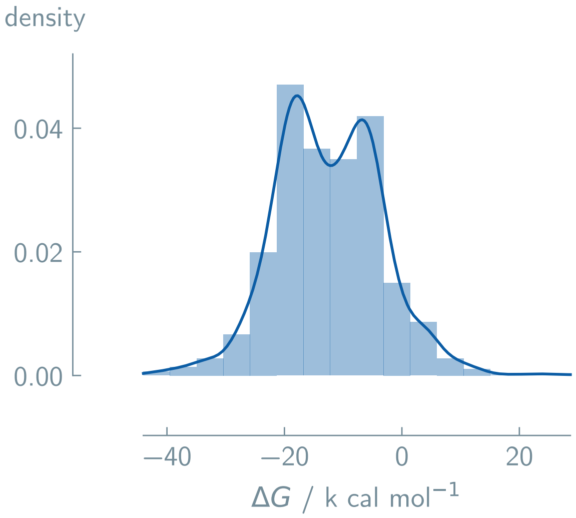


**Figure 63. Distribution of the relative free energy values of intermediate 4 in the dataset.** The median relative free energy is $-12.72\,\mathrm{kcal\,mol^{-1}}$.

### 4.2.3 LLM results

**Base Case** As an initial test, we performed a learning curve analysis for the prediction of our property of interest, i.e., the relative free energy of intermediate 4 $\Delta G_{RRS}(4)$. We used the SMILES notation as the representation of the catalysts and trained separate models to predict the binary class of the relative free energy. As the total dataset contained 1,423 entries, our maximum number of training data was set to 1000. The number of test data remained constant over all runs, i.e., 50. The number of epochs screened was 15 and 20. For every pair of training size-epoch experiments, three unique runs were performed to get the average metrics (Figure 64).

For all properties, we observed no significant difference between experiments with 15 and 20 epochs. We also see that the accuracy converges after a training set size of 100 for all cases. Note that, since we are dealing with binary classification of a balanced dataset, all accuracies above 50% are an improvement to random guessing.

In a next step, we screened different models. Three LLMs, i.e., GPT-J, Llama, and Mistral, were fine-tuned. We notice that a maximum accuracy of 88% was reached with the GPT-J model (Figure 65 and Table 28).

**Real split** Volcano plots describe the relationship between a descriptor of the catalytic cycle and the overall catalytic activity of the catalyst. They offer a great visualization of the performance of catalysts. The molecules at the peak of the plot are usually described as 'optimal' catalysts, whereas entries left or right of the peak have a suboptimal property or even

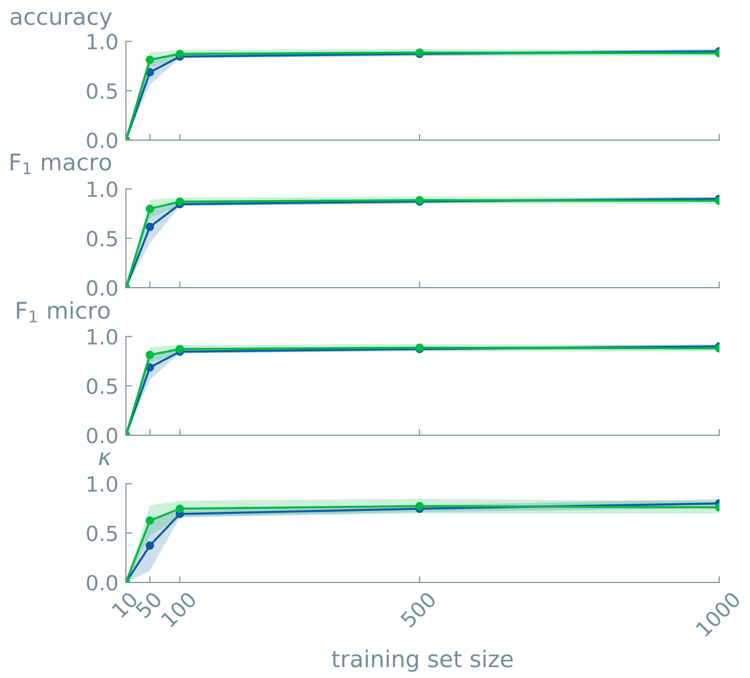


**Figure 64. Learning curve analysis of binary classifications of the relative free energy of intermediate 4.** Three runs were performed to get an average and standard deviation of the metric. Experiments were performed with the GPT-J model. Different numbers of epochs were analyzed, i.e., 15 (blue) and 20 (green). A maximum accuracy of 90% was reached for a training set size of 1,000 and 20 epochs.

**Table 28. Overview of results of LLMs predicting the binary class of the free energy of intermediate 4 of the described nickel catalysis.** Three runs were performed to get the metrics average. All LLMs were fine-tuned with 25 epochs and a learning rate of 0.003. Maximum performances are highlighted in bold.

| Size | Model | Accuracy | F1 Macro | F1 Micro | Kappa |
|---|---|---|---|---|---|
| 1000 | **GPT-J (LLM)** | **0.88** | **0.88** | **0.88** | **0.76** |
| | Llama (LLM) | 0.71 | 0.68 | 0.71 | 0.43 |
| | Mistral (LLM) | 0.79 | 0.78 | 0.79 | 0.59 |
| | Zero-rule | 0.50 | 0.50 | 0.50 | 0.00 |

exceed the optimal range (Figure 66).

For the catalytic cycle at hand, $\Delta G_{RRS}(4)$ values between $-36$ and $-30\,\mathrm{kcal\,mol^{-1}}$ ($\pm 3\,\mathrm{kcal\,mol^{-1}}$) were observed to be of particular interest. We used this range as a real-life

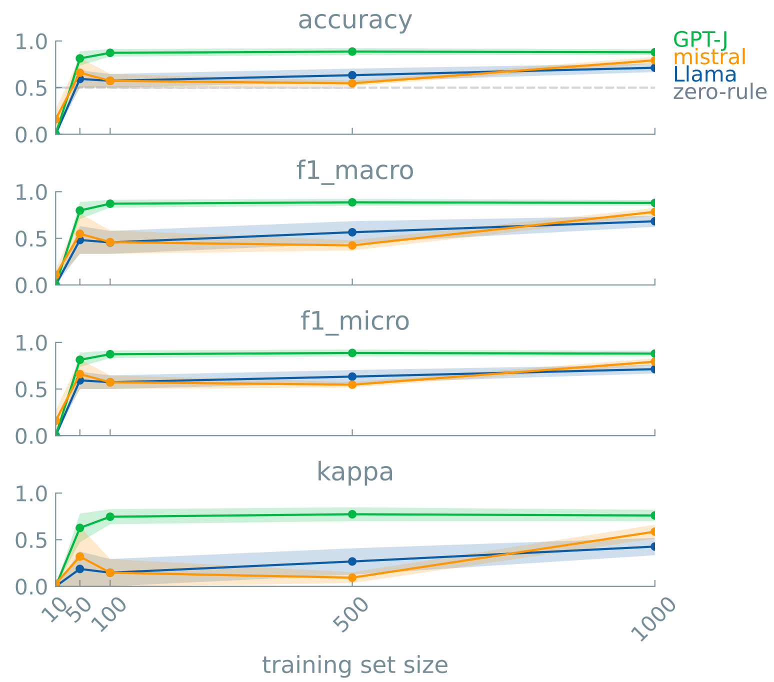


**Figure 65. Learning curve analyses of binary classification of the free energy of intermediate 4 of the described nickel catalysis using different models.** Three base LLMs (GPT-J, Llama, and Mistral) and two traditional ML models (XGBoost and random forest (RF)) were validated on predicting the binary class of the free energy of activation of intermediate 4 of the described nickel catalysis. We used 50% as a random guess accuracy (dashed line), representing the zero rule baseline. For each model, three runs were performed to get the metric's average and standard deviation. The fine-tuned GPT-J model reached the maximum accuracy of 88% (training set size of 1,000 and 25 epochs).

test case to predict whether a catalyst is suitable for the reaction. From the 1,423 entries, only 55 catalysts (3.8%) had a $\Delta G_{RRS}(4)$ value between −27 and −39 $\mathrm{kcal\,mol^{-1}}$. To create a balanced training set, we undersampled the 'non-optimal' catalyst class, i.e., random sample of 55 molecules was taken. Rather than 3 runs, we now performed 10 experiments, each with a different class sample, to increase variance.

Figure 67 shows the results of our LLM predictions with GPT-J using the same hyperparameters (i.e., 20 epochs and a learning rate of 0.0003). As we are working with a balanced dataset, the random guessing benchmark accuracy to beat is 50%. We could predict whether a catalyst is within the optimal range with an accuracy of 78.5% (average over 10 runs).

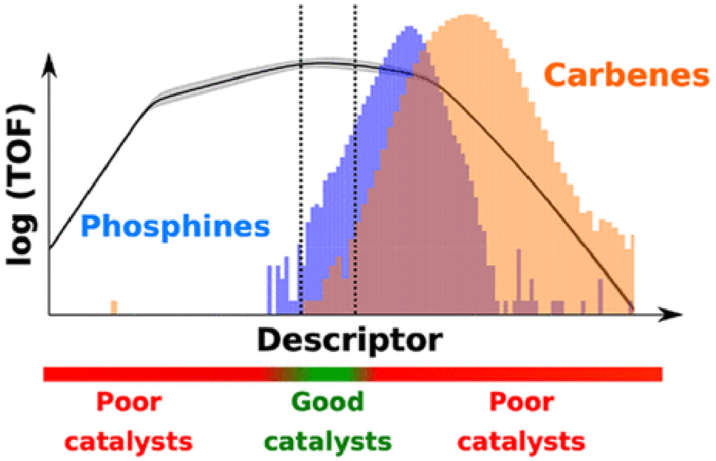


**Figure 66. TOF Volcano plot of the catalytic cycle.** The theoretical turnover frequency (TOF) is plotted over the descriptor value, here the relative free energy of intermediate 4 ($\Delta G_{RRS}(4)$). Ideal catalysts are located at the top of the plot. Adapted from Cordova et al.[55].

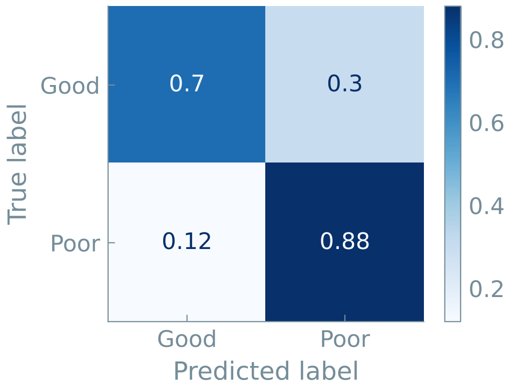


**Figure 67. Normalized confusion matrix of models predicting the performance of the nickel catalyst.** The confusion matrix contains the combined predictions of 10 individual training/test runs (GPT-J). A balanced dataset of 100 data points was used for training, number of epochs and the learning rate were set to 20 and 0.0003, respectively. The average accuracy over 10 unique models was 78.5%.

## 4.3 Yield of Catalytic Isomerization

The dataset was provided by: Leander Choudhury[11]

### 4.3.1 Scientific Background

Isomerization reactions are essential in organic synthesis and industrial processes because they allow for the transformation of molecules without changing the overall molecular composition. These reactions are often accelerated by catalysts, which include transition metal complexes. In the current study, the curator performed a scoping study of a wide range of isomerization reactions of cyclopropenes to 1,3-dienes, all catalyzed by Platinum(II) bromide ($PtBr_2$). These resulting 1,3-dienes are interesting building blocks in chemical synthesis.[56] Hence, predicting the success of these isomerization reactions could be an alternative to scoping studies.

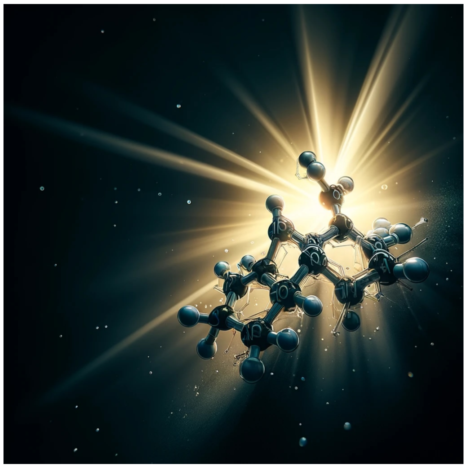

**Figure 68.** AI generated representation of a isomerization reaction catalyzed by Platinum(II) bromide.

### 4.3.2 Dataset

The dataset contains 16 experimental reaction protocols for catalytic isomerisation.[57] The starting material was the only variable in the reaction conditions, and as a result, the product also changed. Both the starting material and the product were represented in the SMILES notation. The reaction conditions were constant for all entries ($PtBr_2$ as catalyst, 1.2-DCE as solvent, 70 °C) and were, therefore, omitted in the prompt.

We aimed to predict the yield of the isomerization. These values ranged from 5% to 98%. Figure 69 shows the distribution of the yield values. The noise in the data could be rather high as both the starting material and the product are quite unstable. This is especially true for benzylic alcohols that tend to polymerize when subjected to small amounts of acid. Expected potential product losses from instability can be up to 15% in some cases, but are limited to 2% for most protocols. In our experiments, the median yield of 50.5% was taken as the threshold for a successful reaction. As this value can also be interpreted as a realistic experimental benchmark, we also took the trained model as the real-life case.

Table 29 shows an example of the prompts we use to fine-tune our LLM model.

[11] Laboratory for Computational Molecular Design (LCMD), Institute of Chemical Sciences and Engineering (ISIC), École Polytechnique Fédérale de Lausanne (EPFL), CH-1015 Lausanne, Switzerland

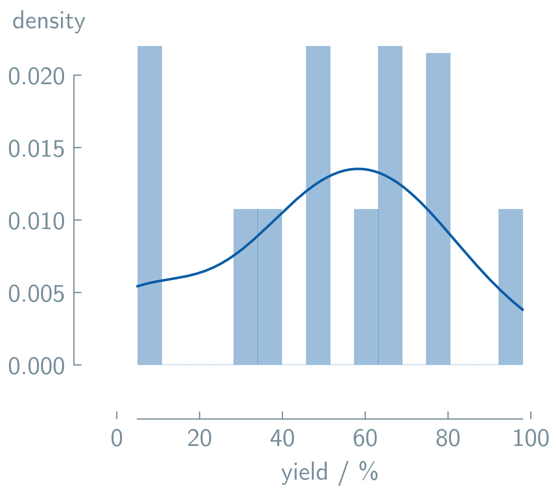


**Figure 69. Histogram of the yield of the catalytic isomerization reaction.** The median of yield is 50.5%.

**Table 29. Example prompts and completions for predicting yield of isomerisation.** `<SMILES>` serves as a placeholder for the representation of the structure.

| prompt | completion | experimental |
|---|---|---|
| Example of training data | | |
| `What is the yield of isomerization from <SMILES> to <SMILES>?` | 0 | Low |
| `What is the yield of isomerization from <SMILES> to <SMILES>?` | 1 | High |

### 4.3.3 LLM results

**Base Case** With only 16 entries, the provided dataset was rather small. We were thus limited in the training examples. This case study was again a search for the limits of the capabilities of our approach.

The first experiments with 25 epochs were not successful (Figure 70). The models could not capture the prompt/completion structure and returned invalid completions for the test data, e.g., '-9223372036854775808' as the completion.

Next, we increased the number of epochs to 50, hypothesizing that this would increase the fine-tuning performance. Indeed, we do see an increase in accuracy, i.e., 50%. However, as the baseline accuracy in our binary classification is 50%, we can conclude that this value does not represent any true predictive power. This is also reflected in a kappa value of 0.

We can only tell the model ’learned’ the prompt/completion format, as the outputs are now ‘0’ or ‘1’. Experiments using different LLMs, i.e., GPT-J, Llama, and Mistral, didn’t show improvement on the predictive power (Figure 71).

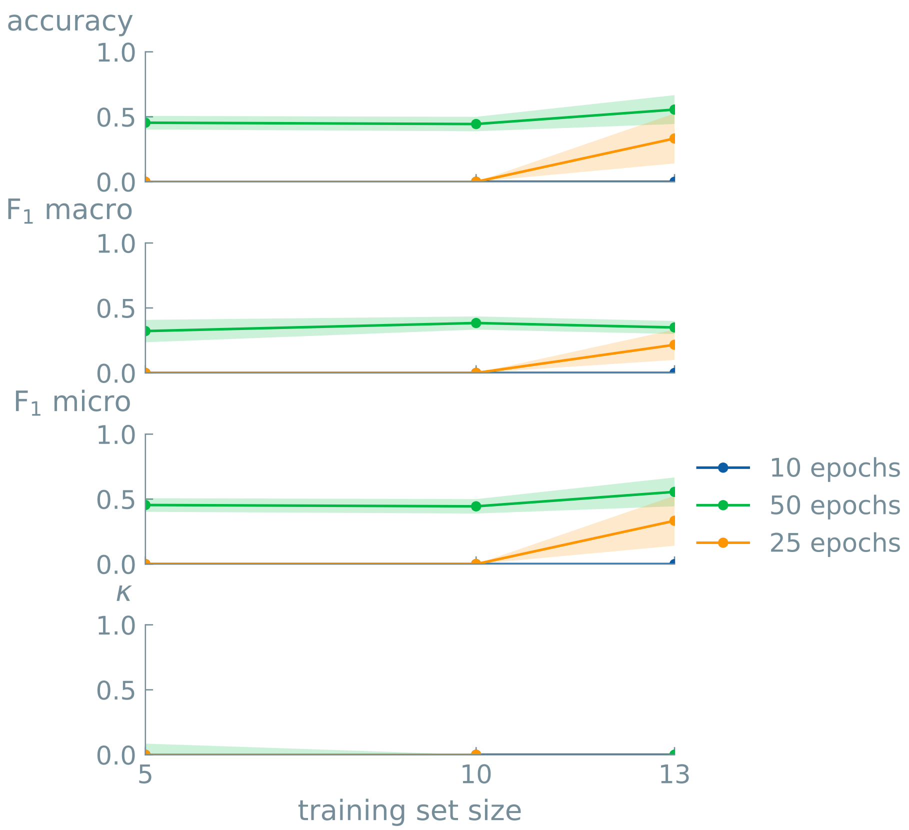


**Figure 70. Learning curve analysis of predictions for the success of the catalytic isomerization.** Models fine-tuned with 10 (blue), 25 (orange), and 50 (green) epochs were validated. The trained models were unable to predict the success of the isomerization.

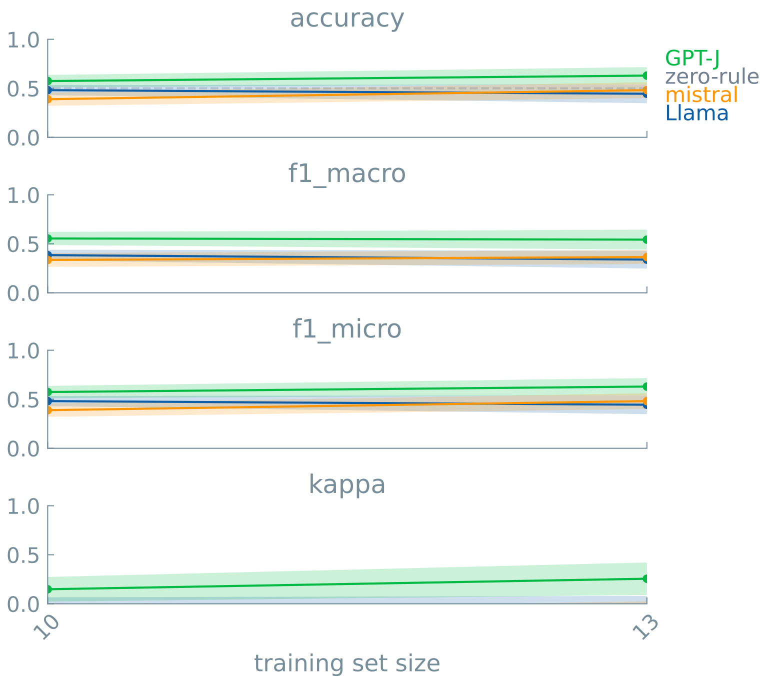


**Figure 71. Learning curve analyses of binary classifications of the success of the catalytic isomerization using different models.** Three LLMs (GPT-J, Llama, and Mistral) were validated on predicting the binary class of the the success of the catalytic isomerization. Three runs were performed for each model to get the metric's average and standard deviation.

## 4.4 Kinetics of Polymerization

The dataset was provided by: Joren Van Herck and Tanja Junkers[12]

### 4.4.1 Scientific Background

Flow chemistry is an attractive alternative to traditional batch reactions. It allows for continuous synthesis and dynamic changes in reaction conditions, e.g., changing reactants stoichiometry by altering respective flow rates. The small diameters of the reactor further improve mixing and heat transfer. Adding analytic tools in the stream of synthesis, i.e., inline analysis can monitor chemical transformations in real-time. These combined features make flow setups highly suitable for high-throughput kinetic screenings. As the residence time inside the flow reactor is related to the feed flow rate, it can be dynamically changed within one reactor space by simply altering the feed settings. Real-time data acquisition downstream of the reactor can continuously monitor the reaction product.

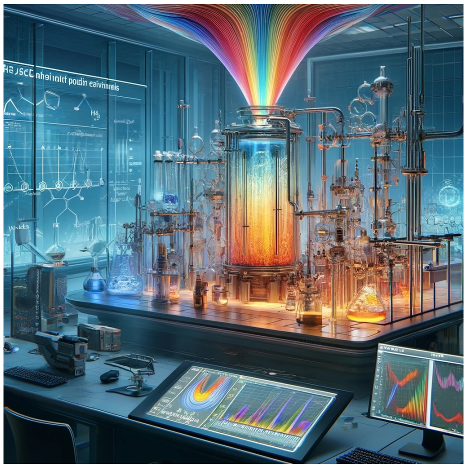

**Figure 72.** AI generated representation of a flow chemistry setup for polymer chemistry.

Recent advancements in high-throughput screenings greatly benefit the field of polymer chemistry. Collecting large datasets on the kinetics of polymerization gives better insight into the often complex underlying reaction mechanism and, subsequently, accelerates the search for optimal reaction conditions.

Van Herck et al.[58] developed a fully automated polymer synthesis platform for high-throughput kinetic screenings of reversible-addition fragmentation chain transfer (RAFT) radical polymerization. The setup comprises a flow reactor coupled to an inline bench-top NMR and online SEC. While the first continuously (scan every 3 seconds) monitors the monomer conversion, the latter measures the molecular weight distribution every three minutes. They performed kinetic screenings of eight different monomers, each under three different reaction conditions. The data from each screening was used to calculate the rate of polymerization.

In this study, we aimed to predict the rate of polymerization based on the monomer and the reaction conditions.

[12]Polymer Reaction Design group, School of Chemistry, Monash University, Clayton VIC 3800, Australia

### 4.4.2 Dataset

The dataset contains kinetic data of RAFT polymerizations.[58] Eight different monomers were screened. The monomers were all acrylates with variations in the ester side chain: methyl acrylate, ethyl acrylate, propyl acrylate, n-butyl acrylate, iso-butyl acrylate, 2-ethylhexyl acrylate, cyclohexyl acrylate, and dodecyl acrylate. Three monomer concentrations were screened for all monomers, i.e., 1 M, 2 M, and 4 M (4 M was not used for dodecyl acrylate). The dataset, therefore, resulted in 23 entries. For every reaction, the rate of polymerization was experimentally determined. The distribution of the data is shown in Figure 73. The median rate of polymerization was 0.033 M/s.

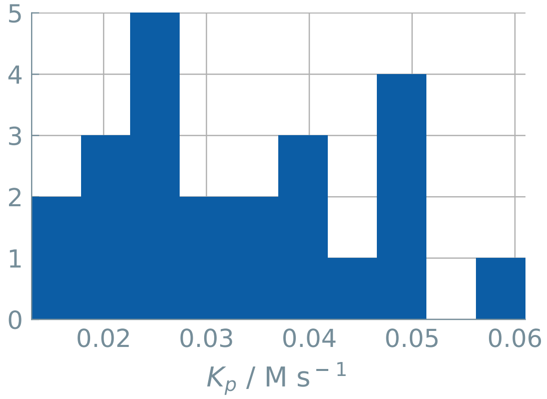


**Figure 73. Distribution of polymerization rate of the monomers in the dataset.** The median polymerization rate is 0.033 M/s

Table 30 shows an example of the prompt we use to fine-tune our LLM model.

**Table 30. Example prompts and completions for predicting the polymerization rate.** `<molar>` is a placeholder for the concentration used in the reaction, and `<monomer>` is the placeholder for the representation of the monomer as shown in Table 31.

| prompt | completion | experimental |
|---|---|---|
| | Example of training data | |
| `What is the polymerization rate of <molar> molar <monomer>?` | 0 | Low |
| `What is the polymerization rate of <molar> molar <monomer>?` | 1 | High |

**Table 31. Representation of the monomers.** Two example entries illustrate the different representations of the monomers.

| | Example 1 | Example 2 |
|---|---|---|
| name | ethyl acrylate (2 molar) | dodecyl acrylate (1 molar) |
| SMILES | CCOC(=O)C=C (2 molar) | CCCCCCCCCCCCOC(=O)C=C (1 molar) |
| length | 11 (2 molar) | 21 (1 molar) |
| rate | 0.026 | 0.035 |
| bin | 0 | 1 |

### 4.4.3 LLM results

**Base Case** The relatively small size of the dataset forced us to use a small training size. We performed the standard experiment with a training set size of 15 and a test set of 7. For all cases, we combined the representation of the monomer and the concentration. We aimed to predict the binary class of the rate of polymerization where the median value (0.033 M/s) served as the threshold. Three unique runs were performed for every experiment to get the average metrics. In initial experiments, we fine-tuned LLMs on different representations of the monomer, i.e., the IUPAC name, SMILES, and the length of the SMILES (number of atoms). The number of epochs was set to 100. We found that fine-tuned models based on the SMILES of the monomer (accuracy of 76%) do better than models based on the IUPAC name of the monomers (accuracy of 57%) and based on the length of the SMILES (accuracy of 57%).

To further examine the capability of LLMs on this small dataset, we performed a 'Leave One Out' analysis where we used all but one monomer set as training data and that one monomer set as only test data ($n = 3$). For instance, we left the methyl acrylate set, i.e., three different monomer concentrations, out of the training set, and, after fine-tuning the model, we predicted the binary class of these three reaction conditions. Five runs were performed for every train-test split. Three different LLMs, i.e., GPT-J, Llama, and Mistral, were validated. The results are summarized in Table 32. More detailed overviews per model are shown in Figure 74 for GPT-J, Figure 75 for Llama, and Figure 76 for Mistral. Here, the monomer on each row represents the test set ($n = 3$). For each monomer, the three different reaction conditions, i.e., monomer concentration in the columns, are predicted. The experiment is further split in the number of epochs used for the training. Each square represents the prediction of five fine-tuned models of the monomer-concentration-epoch combination. The color indicates the accuracy over the five runs where the darkest blue indicates five correct predictions and the lightest blue means zero correct predictions. We see that, in general, the number of epochs has a minor influence on the predictions. For the Mistral model, an increase of 8% in accuracy is observed when increasing the number of epochs from 25 to 100.

Looking closer, small individual improvements are seen for reactions with a polymerization rate around the threshold of 0.033 M/s. For instance, the GPT-J prediction of 2 M iso-butyl acrylate (with a rate of polymerization of 0.030) increased in accuracy from 25 to 50 epochs. These subtle changes hint that fine-tuned LLMs can indeed capture the relation between the reaction conditions and the rate of polymerization.

**Table 32. Overview of the 'Leave One Set Out' accuracy (%) results of LLMs predicting the binary class of the rate of polymerization of monomers.** The average accuracy over 8 monomers (each five runs) are reported. The maximum performance is highlighted in bold.

| Epochs | GPT-J | Llama | Mistral |
|---|---|---|---|
| 25 | 0.76 | 0.77 | 0.73 |
| 50 | 0.80 | 0.83 | 0.80 |
| 100 | 0.79 | **0.83** | 0.81 |

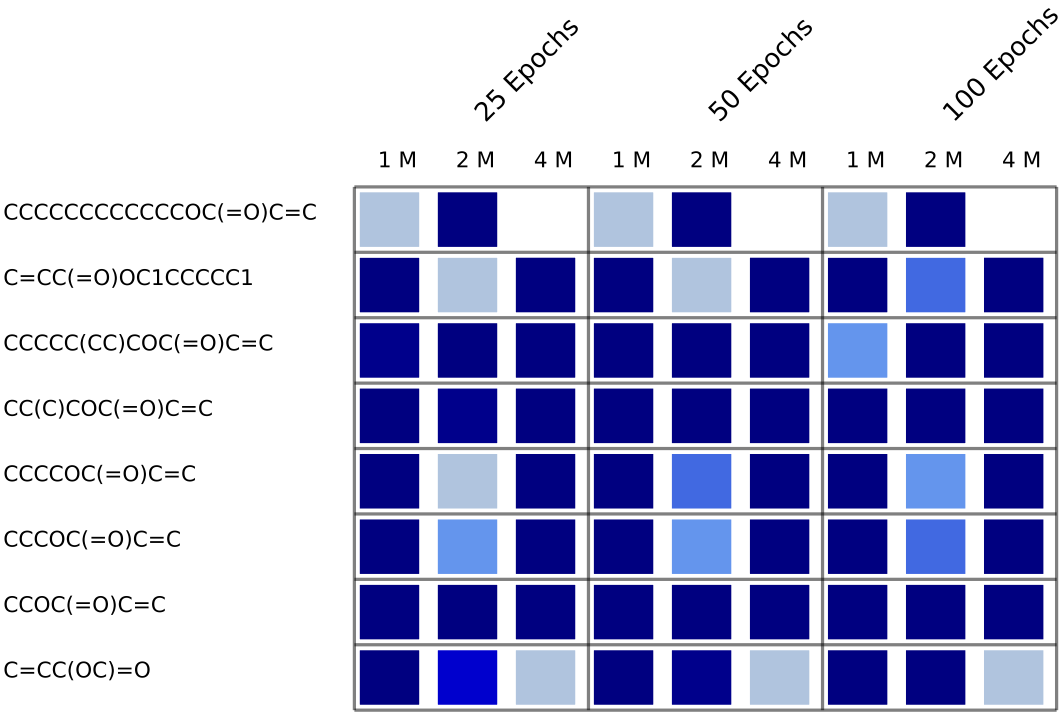


**Figure 74. Overview of models predicting the rate of polymerization (GPT-J).** Each square represents the predictions over five different seeds of a 'monomer', 'concentration' and 'epoch' combination. All monomers were represented by their SMILES notation. The color indicates the accuracy over the five runs where the darkest blue indicates five correct predictions and the lightest blue means zero correct predictions. Four, three, two and one correct predictions have intermediate blue colors. Average accuracies are 0.76, 0.80 and 0.79 for 25 epochs, 50 epochs and 100 epochs, respectively.

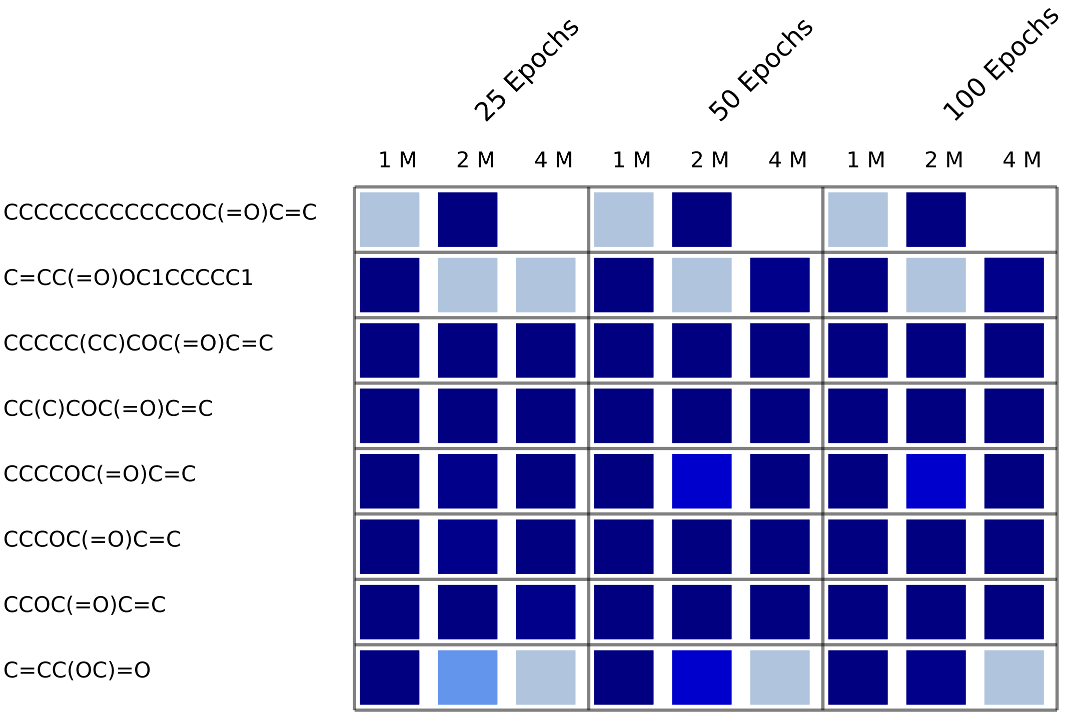


**Figure 75. Overview of models predicting the rate of polymerization (Llama).** Each square represents the predictions over five different seeds of a 'monomer', 'concentration' and 'epoch' combination. All monomers were represented by their SMILES notation. The color indicates the accuracy over the five runs where the darkest blue indicates five correct predictions and the lightest blue means zero correct predictions. Four, three, two and one correct predictions have intermediate blue colors. Average accuracies are 0.77, 0.83 and 0.83 for 25 epochs, 50 epochs and 100 epochs, respectively.

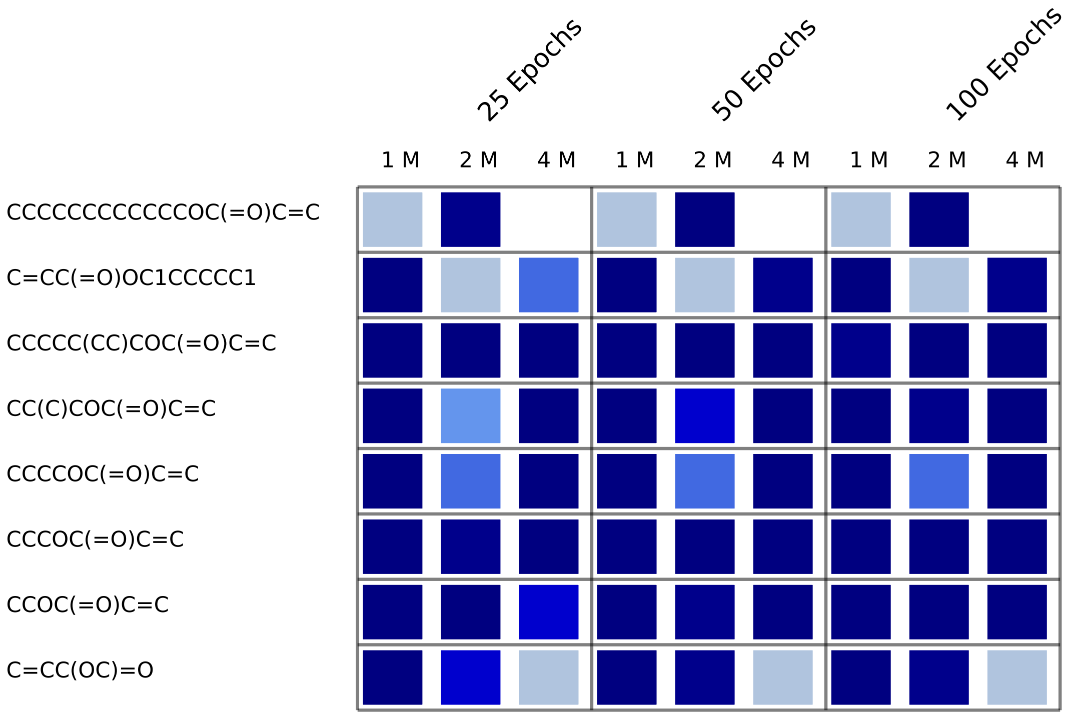


**Figure 76. Overview of models predicting the rate of polymerization (Mistral).** Each square represents the predictions over five different seeds of a 'monomer', 'concentration', and 'epoch' combination. All monomers were represented by their SMILES notation. The color indicates the accuracy over the five runs where the darkest blue indicates means five correct predictions and the lightest blue means zero correct predictions. Four, three, two, and one correct predictions have intermediate blue colors. Average accuracies are 0.73, 0.80, and 0.81 for 25 epochs, 50 epochs and 100 epochs, respectively.

## 4.5 Photocatalytic Water Splitting Activity of MOFs

The dataset was provided by: Beatriz Mouriño, Sauradeep Majumdar, and Xin Jin[13]

### 4.5.1 Scientific Background

As the practical implication of green energy becomes increasingly a reality, much attention has been given to photocatalysis in recent years. The everlasting sunlight could drive chemical transformations independent of fossil fuels. One notable example is water splitting mediated by a photocatalyst. The resulting hydrogen production could serve as a defining step towards a more sustainable hydrogen economy. It is a promising theory indeed, but finding a suitable material is the key to unlocking its success. Thanks to their inherent modular nature, Metal-Organic Frameworks (MOFs) and Covalent Organic Frameworks (COFs) are put high on the photocatalysis shortlist.[59] However, it is also exactly this modularity that results in millions of potential structures, and hence, careful selection rules are needed to filter the 'best' from the rest.

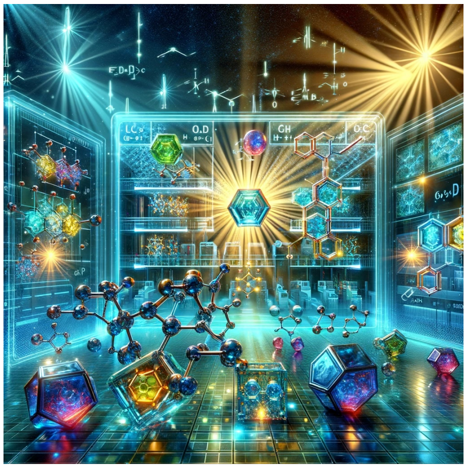

**Figure 77.** AI generated representation of the concept of photocatalysis for sustainable hydrogen production using Metal-Organic Frameworks (MOFs) and Covalent-Organic Frameworks (COFs).

The first obvious criterion is the ability to absorb visible light. Quantitatively, this translates itself into a bandgap that lies within the visible light range of 1.6 eV to 3.2 eV. Subsequently, upon absorption, the ionization potential (IP) and electron affinity (EA) should be straddling the potentials of the surface reactions, i.e., −4.4 eV to −5.6 eV for hydrogen evolution reaction (HER) and oxygen evolution reaction (OER), respectively. IP and EA can be estimated computationally by aligning the valence and conduction band edges to a reference vacuum potential. Lastly, the generated charge carriers ideally have limited recombination, thus increasing their lifetime. Similarly, a high mobility of electrons and holes is preferred to improve the material's overall efficiency.[60]

It is no surprise that experimental screening of a large number of MOFs/COFs is out of the picture. An alternative and more sustainable approach in the search for the perfect candidate is using high-throughput computational screening. For instance, the QMOF database, curated by Rosen et al.[61], contains over 15,000 MOFs. These structures successfully ran through a DFT workflow that calculated electronic properties. Recently, an identi-

[13] Laboratory of molecular simulation (LSMO), Institut des Sciences et Ingénierie Chimiques, École Polytechnique Fédérale de Lausanne (EPFL), Rue de l'Industrie 17, CH-1951 Sion, Switzerland

cal database for COFs was developed by Mouriño et al.[62].

Reticular chemistry also benefits from the increasing popularity of machine learning algorithms. The vast number of possible structures provide training data for the models. On the other hand, the ability to accurately and quickly predict properties in a complex parameter landscape greatly accelerates the identification of materials for specific applications. The ML-assisted search for MOFs that satisfy some basic conditions for photocatalytic water splitting will be discussed in the following experiments. By predicting important indicators for success, certain structures can be prioritized over others, both for in silico calculations and experimental validation.

### 4.5.2 Dataset

The dataset contains 95 MOFs. DFT was used to compute the photocatalytic properties. We aimed to predict the potential of the structure for water splitting based on three properties:

- whether band edges (aligned to vacuum) are straddling the HER redox potential ($-4.4$ eV),
- whether band edges (aligned to vacuum) are straddling the OER redox potential ($-5.6$ eV), and
- whether the band gap is within the visible light range (1.6 eV to 3.2 eV).

For all three cases, the elemental composition was used to predict the binary class of the property. We did not create a balanced dataset. Therefore, the percentage of the majority class was the random guess mark, i.e., 70%, 90%, and 60% for the HER, OER, and VIS case studies, respectively (fig. 78).

An example of the prompt we use to fine-tune our LLM model is shown in Table 33.

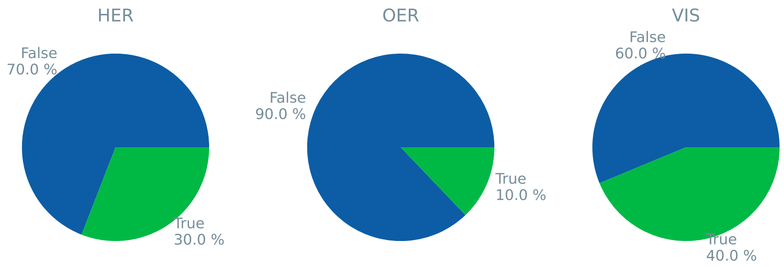


**Figure 78. Distribution of the three properties related to the photocatalytic activity of MOFs.** The pie plots show the distribution based on whether or not the band edges of a material straddle the redox potential in the HER and OER cases. For the VIS case study, the distribution is based on whether or not the band gap is within the visible light range.

**Table 33. Example prompts and completions for predicting photocatalytic properties of MOFs.** `<property>` is a placeholder for the HER redox potential, OER redox potential, or bandgap. `<MOF>` serves as a placeholder for the representation of the structure as shown in 34.

| prompt | completion | experimental |
|---|---|---|
| | Example of training data | |
| `What is the <property> of <MOF>?` | 0 | Low |
| `What is the <property> of <MOF>?` | 1 | High |

**Table 34. Representation of the MOF structures.** Two example entries illustrate the five different representations of the materials.

| | Example 1 | Example 2 |
|---|---|---|
| Linker elements | C, N, S | C, N |
| Node elements | Cu | Au, Cl |
| Linker + Node elements | C, N, Cu, S | Au, C, N, Cl |
| `mofid` | S=C1N=CC..._-2.00 | Cl[Au]..._pyrazolate_opt |
| `mofkey` | Cu.UO...H.MOFkey-v1.rtl | Au.G...R.MOFkey-v1.bto |

### 4.5.3 LLM results

**Real split** The provided dataset gave us three test cases, i.e., HER, OER, and VIS. The results for the different representations of the structures used are shown in Table 35 and Figure 79.

Firstly, the chemical elements of both the organic linker and the metal node were extracted and used separately as the input. From these basic representations, we can already make accurate models. We see that for the HER model, the elements of the organic linker have a slightly higher predictive power than the metal node. In contrast, the OER model based on the metal node outperforms its linker counterpart. A combined feature vector with both the elements of the metal node and the organic linker didn't have an improved effect on the performance and was, in all cases, similar to its model with the best single descriptor, i.e., linker and metal elements for HER and OER, respectively.

The `mofid` and the `mofkey`, i.e., a shorter, hashed version of the `mofid`, add extra structural information to the MOF representation.[63] We, however, see that there is no significant increase in performance. We can, therefore, conclude that only the elemental composition of the building blocks of the MOFs already holds enough information to predict the binary class of the photocatalytic properties. It is interesting to note that we outperform the random guessing baseline for the HER and VIS experiments. For the OER predictions, the accuracy

**Table 35. Overview of accuracies for binary classifications for HER, OER, and VIS.** Three runs were performed to get the metric's average. The GPT-J model was used. For all experiments, the training size and number of epochs were set to 50 and 25, respectively.

| Feature(s) | HER | OER | VIS |
|---|---|---|---|
| | Accuracy / % | | |
| Linker Elements | 0.86 | 0.83 | 0.89 |
| Node Elements | 0.79 | 0.89 | 0.74 |
| Linker + Node Elements | 0.86 | 0.89 | 0.90 |
| mofid | 0.90 | 0.83 | 0.93 |
| mofkey | 0.89 | 0.86 | 0.95 |
| Random Baseline | 0.70 | 0.90 | 0.60 |

of the LLM was no better than the random guessing baseline.

Three LLMs, i.e., GPT-J, Llama, and Mistral, were fine-tuned. The elements of both the linker and metal cluster were used as the representation of the MOFs. We notice that all three models are comparable in performance. A maximum accuracy of 95% was reached with the Mistral model for predicting the binary class of the VIS property. (Table 36).

**Table 36. Overview of results of LLMs predicting the binary class of photocatalytic properties of MOFs.** Three runs were performed to get the metrics average. All LLMs were fine-tuned with 25 epochs and a learning rate of 0.003. The training set size was 25. The linker and node elements were used as the representation of the MOFs. Maximum performances per property are highlighted in bold.

| **Target** | **Model** | **Accuracy** | **F1 Macro** | **F1 Micro** | **Kappa** |
|---|---|---|---|---|---|
| HER | **GPT-J (LLM)** | **0.92** | **0.92** | **0.92** | **0.83** |
| | Llama (LLM) | 0.64 | 0.63 | 0.64 | 0.28 |
| | Mistral (LLM) | 0.89 | 0.89 | 0.89 | 0.78 |
| OER | **GPT-J (LLM)** | **0.94** | **0.94** | **0.94** | **0.89** |
| | Llama (LLM) | 0.83 | 0.82 | 0.83 | 0.67 |
| | Mistral (LLM) | 0.78 | 0.77 | 0.78 | 0.56 |
| VIS | GPT-J (LLM) | 0.86 | 0.86 | 0.86 | 0.71 |
| | Llama (LLM) | 0.83 | 0.80 | 0.83 | 0.67 |
| | **Mistral (LLM)** | **0.95** | **0.95** | **0.95** | **0.90** |

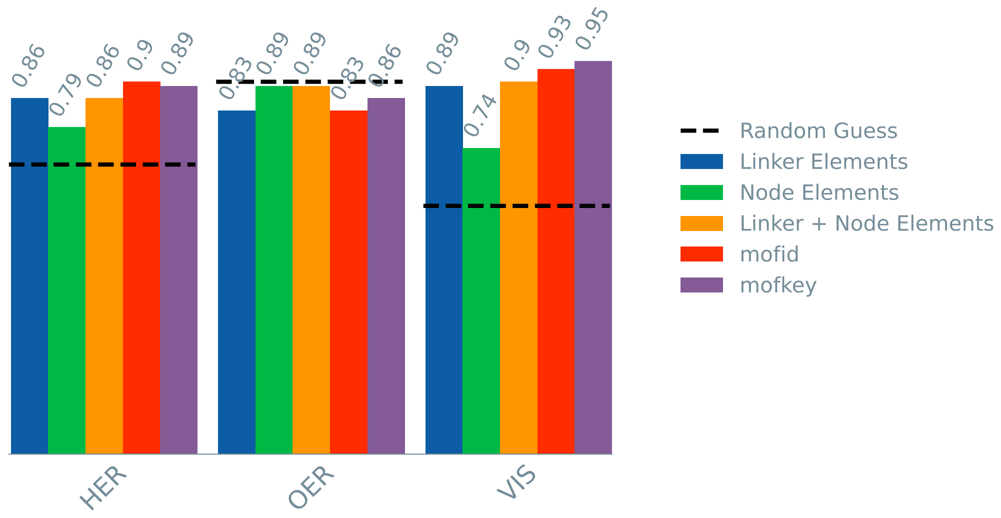


**Figure 79. Overview of accuracies of the binary classifications for HER, OER, and VIS trained on a realistic split based on different feature vectors.** Numbers above bars indicate accuracies. The dotted line indicates the accuracy of the random guessing baseline for all cases. Three runs with the GPT-J model were performed to get the metric's average and standard deviation. For all experiments, the training size and number of epochs were set to 50 and 25, respectively.

## 4.6 Photocatalytic Carbondioxide Conversion Activity of MOFs

The dataset was provided by: Matthew Garvin, Neda Poudineh, Susana Garcia[14] and Ruaraidh D. McIntosh[15]

### 4.6.1 Scientific Background

The current energy crisis and the challenges of global climate change force research in innovative and exciting directions. Promising avenues are those that are similar to nature's own processes. Take photosynthesis, for instance, where sunlight and carbon dioxide are converted into valuable energy. Thus, creating an artificial system that mimics this natural process has the potential to become a key solution to tackle our energy and environmental concerns.[64]

Although an efficiency comparable to that of the natural process is currently out of reach, metal-organic frameworks are among the most promising types of materials to achieve it.[65] Their flexibility in composition and structure allows tuning of their physical properties. Unfortunately, photocatalytic reactions are complex and, despite the flexibility in the synthesis of MOFs to obtain the optimal physical properties, a single MOF structure often fails to fulfill all the necessary requirements. Combining the MOF material with a semiconductor photocatalyst could, however, increase the overall photocatalytic efficiency.[66]

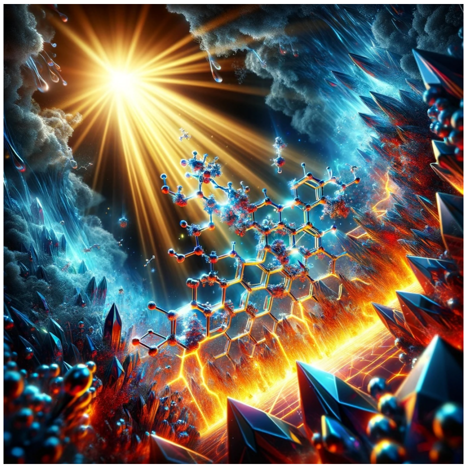

**Figure 80.** AI generated representation of the interaction of Metal-Organic Frameworks (MOFs) with a semiconductor photocatalyst under sunlight for enhanced photocatalytic efficiency.

In this study, we worked with a dataset of the photocatalytic activity of MOFs. We aimed to predict the efficiency of these photocatalytic systems based on various descriptors. Each entry consists of the composition of the MOF, the photocatalytic conversion studied and the band gaps of the MOF and cocatalyst.

### 4.6.2 Dataset

The dataset contains 77 data points, including information on the simplified Molecular Input Line-Entry System (SMILES) representation of MOFs, the catalytic system (including

[14]The Research Centre for Carbon Solutions (RCCS), School of Engineering and Physical Sciences, Heriot-Watt University, Edinburgh, EH14 4AS, United Kingdom

[15]Institute of Chemical Sciences, School of Engineering and Physical Sciences, Heriot-Watt University, Edinburgh, EH14 4AS, United Kingdom

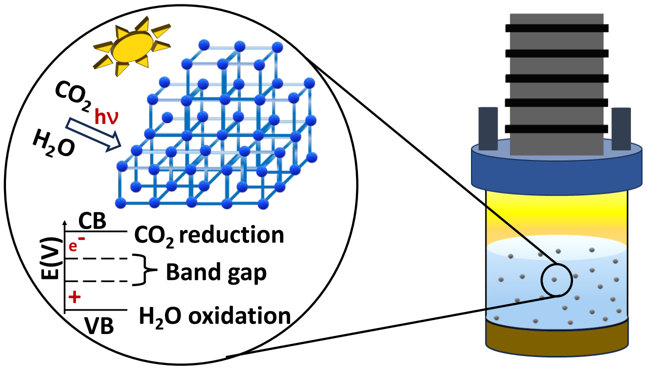


**Figure 81. A schematic of photocatalytic activities of Metal-Organic Framework materials for $CO_2$ photoreduction.**

metal source, linker, phase, sacrificial agent, and cocatalyst), the values of band gaps, conduction bands (CB) and valence bands (VB) for MOF and cocatalyst, as will as the products of the photocatalytic conversion (CO, $CH_4$, $H_2$, $CH_3COOH$, HCOOC, MeOH).

We predict the photocatalytic activity of MOFs. The distribution of the photocatalytic activity values in the dataset is shown in Figure 82. To predict the photocatalytic activity of MOFs, we compare the classification models trained with four different sets of input variables, as shown in Table 37. The first set includes the SMILES notation of the linker molecule along with the metal to represent the MOF structure, while the other sets include the catalyst system and the band gaps, CB, and VB for MOF and co-catalyst.

We used simple prompt templates, with prompts of the form shown in Table 38, for experiments to predict photocatalytic activity.

### 4.6.3 LLM results

**Base Case** To train the binary classification models, we split the dataset into two classes of equal size based on photocatalytic activity separated by the median, i.e., photocatalytic activity threshold of 39 $\mu mol\,h^{-1}\,g^{-1}$. For this dataset, we used 100 fine-tuning epochs, as otherwise, we obtained many NaN or invalid predictions with the GPT-J model.

Figure 83 shows that there are slight differences in the prediction of photocatalytic activity for the different sets of input variables studied with the GPT-J model. As shown in Figure 83, we find that these models perform slightly better than random guessing (shown by the dashed line). An accuracy of 58% was obtained when we used the “SMILES+metal” notation as the only input variable. The accuracy increased up to 65% when we used the catalyst system characteristics as predictors. When we combined the SMILES notation and the catalyst system parameters in the prompt (“catalyst+SMILES” set of input variables in Table 37), the accuracy increased to 68% for a training set of 65 data points. However, adding

**Table 37. Sets of input variables used to predict the photocatalytic activity of MOFs.**

| input variables | SMILES +metal | catalyst | catalyst +SMILES | catalyst +SMILES +band gaps |
|---|---|---|---|---|
| SMILES | X | | X | X |
| metal source | X | X | X | X |
| linker | | X | X | X |
| phase | | X | X | X |
| sacrificial agent | | X | X | X |
| cocatalyst | | X | X | X |
| bandgap for composite | | | | X |
| bandgap for MOF | | | | X |
| bandgap for cocatalyst | | | | X |
| CB for MOF | | | | X |
| CB for cocatalyst | | | | X |
| VB for MOF | | | | X |
| VB for cocatalyst | | | | X |

CB: conduction band; VB: valence band.

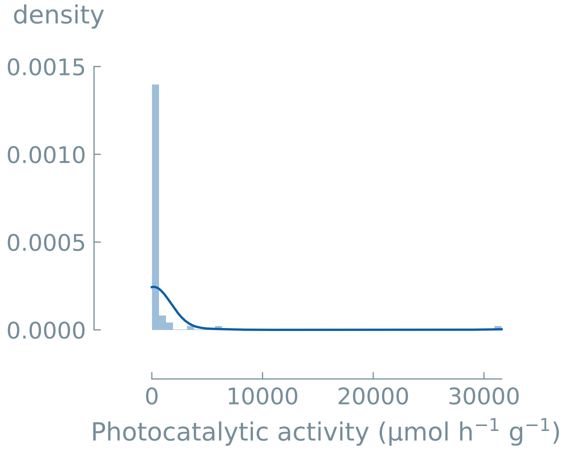


**Figure 82. Distribution of the photocatalytic activity of MOFs in the dataset.** The median of photocatalytic activity is 39 $\mu mol\,h^{-1}\,g^{-1}$.

the band gap, CB and VB values to the prompt ("catalyst+SMILES+band gaps" set of input variables in Table 37) did not result in an increase in accuracy (58%).

We fine-tuned three LLMs, i.e., GPT-J, Llama, and Mistral, using the "catalyst+SMILES" set of input variables, and we also trained two "traditional" ML models, i.e., XGBoost and random forest (RF), for comparison purposes. To train RF and XGBoost, SMILES were converted into Morgan fingerprints.Table 39 and Figure 84 show that the models trained with 100 epochs perform slightly better than random guess (shown by the dashed line). The higher accuracy was achieved with the GPT-J model (68%) for a training set of 65 data points. The performance was slightly lower with other models (57-60%).

As an example, Figure 85 shows an averaged (over five independent runs) normalized confusion matrix for the GPT-J model trained with the "catalyst+SMILES" set of input variables using a training set of 65 data points and 100 epochs. We can see that the predictions are not better than random guessing when we predict samples labeled '1'. We probably need more data to predict the outcome of complex processes such as photocatalysis, as a large number of variables, as well as products, are analyzed in this dataset.

**Real Split** To simulate a more realistic case, we trained binary classification models using unbalanced datasets to predict whether photocatalytic activity is within the top 27% highest values of the dataset (photocatalytic activity threshold = 200 $\mu mol\,h^{-1}\,g^{-1}$). Figure 86 shows that there are no relevant differences in the prediction of photocatalytic activity for the different sets of input variables studied with the GPT-J model. For all of them, Figure 86 shows that the models perform no better than random guessing (shown by the dashed line), achieving an accuracy of 73% when using a training set of 65 data points and 100 epochs

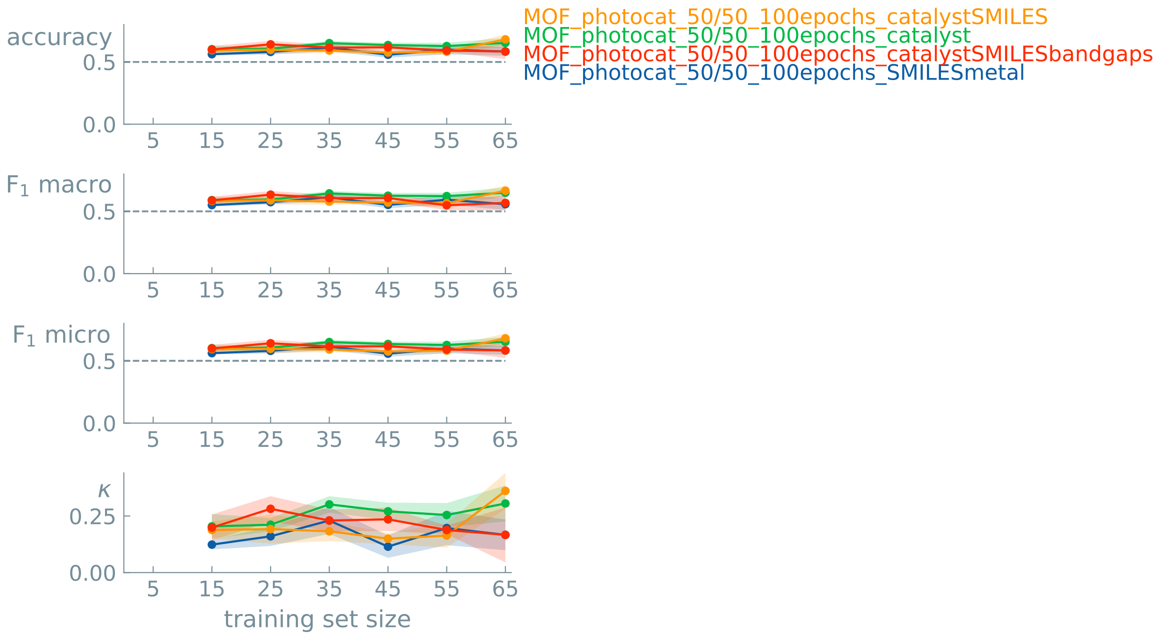


**Figure 83. Learning curves for binary classification GPT-J models (balanced classes) for the photocatalytic activity of MOFs as a function of the number of training points.** Data points indicate the mean value of five different experiments. Error bands show the standard error of the mean. We used 0.50 as random guessing accuracy (dashed line), which represents the zero rule baseline, i.e., a model that always predicts the most common class. Accuracy = 0.681±0.034 when the "catalyst+SMILES" set of input variables was used (epochs = 100, learning rate = 0.001, training set size = 65 data points).

when the "catalyst+SMILES" or the "catalyst+SMILES+band gaps" sets of input variables were used.

Three LLMs (GPT-J, Llama, and Mistral) and two "traditional" ML models (XGBoost and RF) were also fine-tuned with an unbalanced dataset using 100 epochs. Figure 87 shows that the models do not perform better than random guess (shown by the dashed line), obtaining an accuracy of 68-75% when using a training set of 65 data points and 100 epochs.

As an example, Figure 88a shows an averaged (over five independent runs) normalized confusion matrix for the GPT-J model trained with this unbalanced dataset using a training set of 65 data points and 100 epochs. We can see that it clearly fails to predict high values of the photocatalytic activity (i.e., label = 1), which is the least represented class in the dataset.

We also used a balanced dataset created by undersampling the majority class (label = 0) at the cost of reducing the size of the dataset to obtain a model for predicting the photocatalytic activity greater than 200 $\mu mol\,h^{-1}\,g^{-1}$. However, we obtained an accuracy of 60% when

we used the "catalyst+SMILES" set of input variables shown in Table 37), using a training set of 35 data points and 100 fine-tuning epochs. Although this accuracy is slightly better than random guessing, the normalized confusion matrix in Figure 88b shows that the model still fails to predict high values of the photocatalytic activity labeled '1'.

**Table 38. Prompt templates and completions.** These prompts were used to predict the photocatalytic activity of metal-organic frameworks (MOFs) to obtain different products from SMILES and metal, catalyst system, catalyst system together with SMILES, and catalyst system together with SMILES and band gaps, respectively, according to the different sets of input variables shown in Table 37.

| prompt | completion | experimental |
|---|---|---|
| What is the photocatalytic activity (in µmol $h^{-1}$ $g^{-1}$) for <product> of the <SMILES> catalyst made out of <metal source>? | 0 | Low |
| What is the photocatalytic activity (in µmol $h^{-1}$ $g^{-1}$) for <product> of the <SMILES> catalyst made out of <metal source>? | 1 | High |
| What is the photocatalytic activity (in µmol $h^{-1}$ $g^{-1}$) for <product> of a catalyst made out of <metal source> and <linker> in <phase> phase with <sacrificial agent> as a sacrificial agent and <cocatalyst> as a cocatalyst? | 0 | Low |
| What is the photocatalytic activity (in µmol $h^{-1}$ $g^{-1}$) for <product> of a catalyst made out of <metal source> and <linker> in <phase> phase with <sacrificial agent> as a sacrificial agent and <cocatalyst> as a cocatalyst? | 1 | High |
| What is the photocatalytic activity (in µmol $h^{-1}$ $g^{-1}$) for <product> of the <SMILES> catalyst made out of <metal source> and <linker> in <phase> phase with <sacrificial agent> as a sacrificial agent and <cocatalyst> as a cocatalyst? | 0 | Low |
| What is the photocatalytic activity (in µmol $h^{-1}$ $g^{-1}$) for <product> of the <SMILES> catalyst made out of <metal source> and <linker> in <phase> phase with <sacrificial agent> as a sacrificial agent and <cocatalyst> as a cocatalyst? | 1 | High |
| What is the photocatalytic activity (in µmol $h^{-1}$ $g^{-1}$) for <product> of the <SMILES> catalyst made out of <metal source> and <linker> in <phase> phase with <sacrificial agent> as a sacrificial agent and <cocatalyst> as a cocatalyst, with band gap for composite (in eV) of <band gap for composite>, with band gap for MOF (in eV) of <band gap for MOF>, with band gap for cocatalyst (in eV) of <band gap for cocatalyst>, with CB for MOF (in eV) of <CB for MOF>, with CB for cocatalyst (in eV) of <CB for cocatalyst>, with VB for MOF (in eV) of <VB for MOF>, and with VB for cocatalyst (in eV) of <VB for cocatalyst>? | 0 | Low |
| What is the photocatalytic activity (in µmol $h^{-1}$ $g^{-1}$) for <product> of the <SMILES> catalyst made out of <metal source> and <linker> in <phase> phase with <sacrificial agent> as a sacrificial agent and <cocatalyst> as a cocatalyst, with band gap for composite (in eV) of <band gap for composite>, with band gap for MOF (in eV) of <band gap for MOF>, with band gap for cocatalyst (in eV) of <band gap for cocatalyst>, with CB for MOF (in eV) of <CB for MOF>, with CB for cocatalyst (in eV) of <CB for cocatalyst>, with VB for MOF (in eV) of <VB for MOF>, and with VB for cocatalyst (in eV) of <VB for cocatalyst>? | 1 | High |

**Table 39. Overview of the accuracy results of LLMs and "traditional" ML models for binary classification (balanced classes) of the photocatalytic activity of MOFs using the "catalyst+SMILES" set of input variables.** Five runs were performed to get the metrics average. LLMs were fine-tuned with 100 epochs and a learning rate of 0.0003. Maximum performances are highlighted in bold.

| Size | Model | Accuracy | F1 Macro | F1 Micro | Kappa |
|---|---|---|---|---|---|
| 65 | **GPT-J (LLM)** | **0.68** | **0.67** | **0.68** | **0.36** |
| | Llama (LLM) | 0.60 | 0.54 | 0.60 | 0.20 |
| | Mistral (LLM) | 0.58 | 0.49 | 0.58 | 0.17 |
| | RF | 0.60 | 0.59 | 0.60 | 0.20 |
| | XGBoost | 0.57 | 0.51 | 0.57 | 0.13 |
| | Zero-rule | 0.50 | 0.50 | 0.50 | 0.00 |

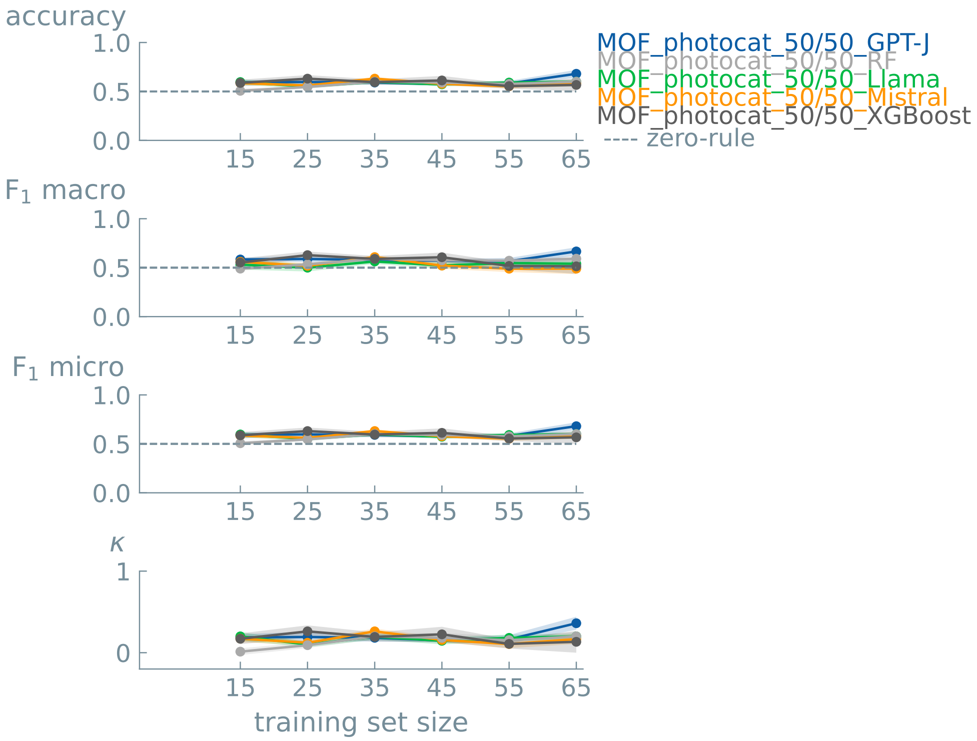


**Figure 84. Learning curves for binary classification models (balanced classes) for the photocatalytic activity of MOFs using the "catalyst+SMILES" set of input variables as a function of the number of training points.** Data points indicate the mean value of five different experiments. Error bands show the standard error of the mean. We used 0.50 as random guessing accuracy (dashed line), which represents the zero rule baseline, i.e., a model that always predicts the most common class. Accuracy: GPT-J=0.681±0.034, Llama=0.600±0.017, Mistral=0.583±0.030, random forest=0.600±0.027, XGBoost=0.567±0.067 (LLM epochs = 100, LLM learning rate = 0.0003, random forest and XGBoost=default parameters, training set size = 65 data points).

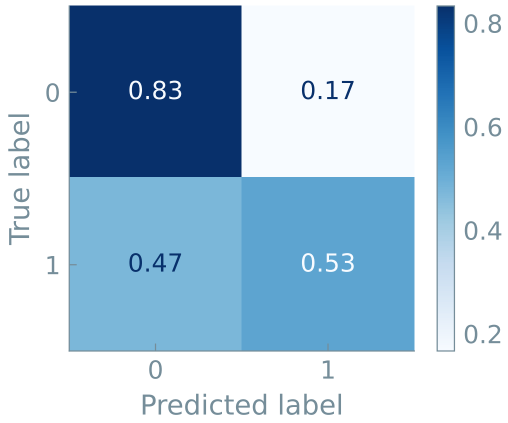


**Figure 85. Normalized confusion matrix, averaged over five independent runs, on the holdout test data for the prediction of the photocatalytic activity of MOFs with the GPT-J model.** Models were trained with the "catalyst+SMILES" set of input variables using a training set of 65 data points and 100 epochs (accuracy = 68%).

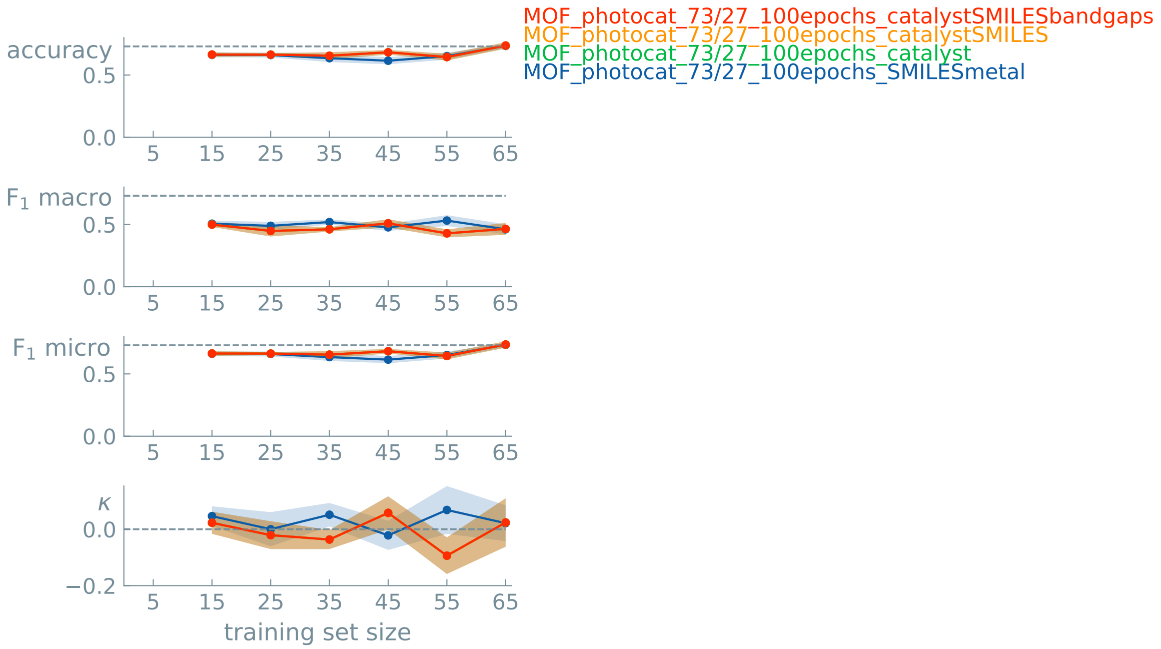


**Figure 86. Learning curves for binary classification GPT-J models (unbalanced classes, 73/27%) for the photocatalytic activity of MOFs as a function of the number of training points.** Data points indicate the mean value of five different experiments. Error bands show the standard error of the mean. We used 0.73 as random guessing accuracy (dashed line), which represents the zero rule baseline, i.e., a model that always predicts the most common class. Accuracy = 0.681±0.033 when the "catalyst+SMILES" set of input variables was used (epochs = 100, learning rate = 0.0003, training set size = 65 data points).

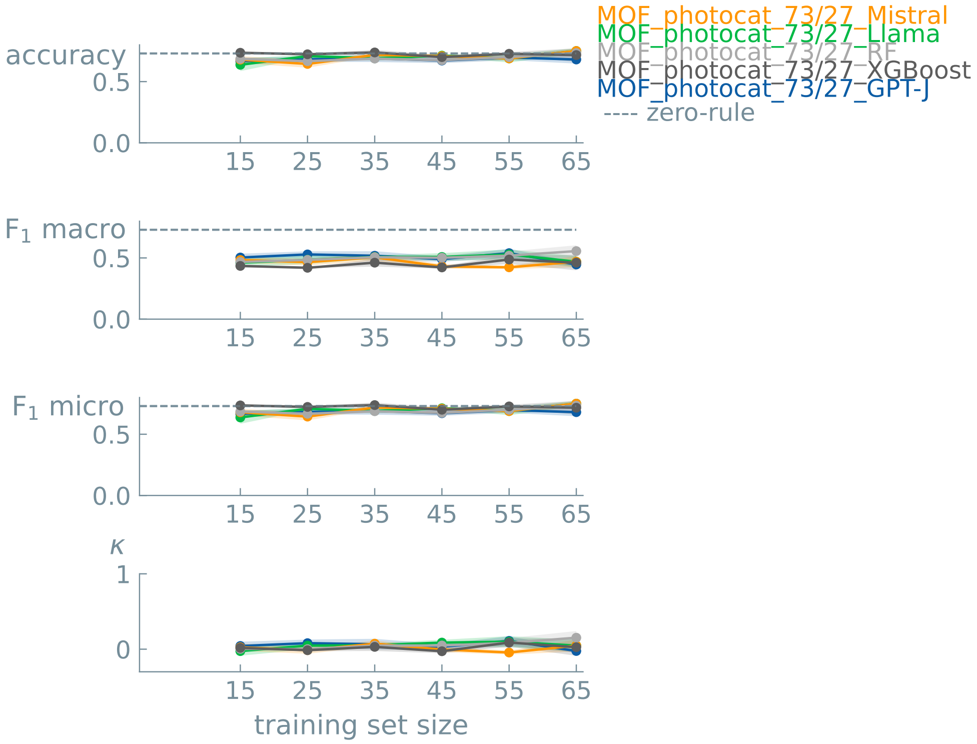


**Figure 87. Learning curves for binary classification models (unbalanced classes, 73/27%) for the photocatalytic activity of MOFs as a function of the number of training points.** Data points indicate the mean value of five different experiments. Error bands show the standard error of the mean. We used 0.73 as random guessing accuracy (dashed line), which represents the zero rule baseline, i.e., a model that always predicts the most common class. Accuracy: GPT-J=0.681±0.033, Llama=0.750±0.022, Mistral=0.750±0.022, random forest=0.733±0.030, XGBoost=0.717±0.056 ("catalyst+SMILES" set of input variables, LLM epochs = 100, LLM learning rate = 0.0003, random forest and XGBoost=default parameters, training set size = 65 data points).

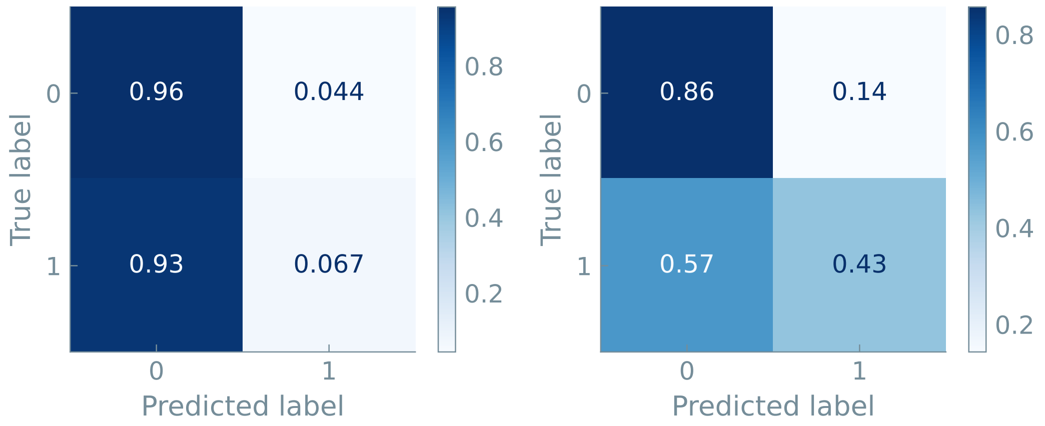


(a) (b)

**Figure 88. Normalized confusion matrix, averaged over five independent runs, on the holdout test data for the prediction of the photocatalytic activity of MOFs with the GPT-J model.** Models were trained with the "catalyst+SMILES" set of input variables using an ´unbalanced' dataset with 73% of labels equal to 0, a training set of 65 data points and 100 epochs (accuracy = 73%) (a), and using a 'balanced' dataset (50/50) with a training set of 35 data points and 100 epochs (accuracy = 60%) (b).

## 4.7 Success of MOF Synthesis

The dataset was provided by: Francesca Nerli and Marco Taddei[16]

### 4.7.1 Scientific Background

Synthetic chemistry is often seen as the basis of material science; the actual synthesis of a material is needed to do further characterization. Small changes in reaction conditions can have large impacts on the success or yield of the reaction. Finding the optimal parameters can improve chemistry and save time and money when upscaling the process. However, this search requires profound knowledge of the chemical background, time, and money.

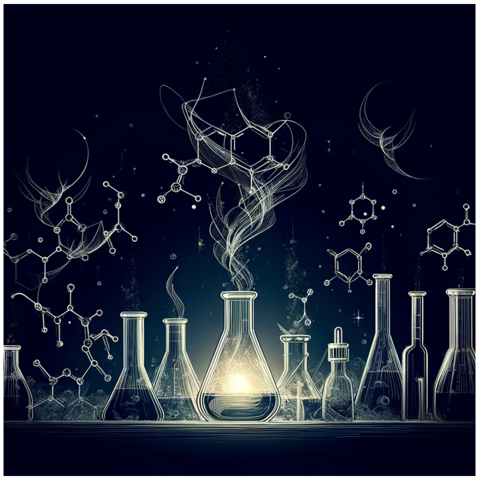

**Figure 89.** AI generated representation of the synthesis of Metal-Organic Frameworks (MOFs).

The following case study concerns the synthesis of metal-organic frameworks. In the last decades, this class of materials has gained much attention because of its versatility and potential applications in various fields. Also, the first challenge to overcome is the successful and effective synthesis of the material. Currently, many of these studies use an approach based on trial-and-error and chemical intuition. As trillions of combinations are possible, a more sustainable optimization workflow is of great interest.

Here, the focus is on optimizing the synthesis of Ce(IV)-based MIL-140A using 2,3,5-trifluoroterephthalate as the organic linker [hereafter F3_MIL-140A(Ce)]. The material was synthesized under 25 different conditions, and the yield and phase purity of the product were determined for every set of parameters. We aim to use this dataset of experimental protocols to predict the success of newly presented reaction conditions, thereby gaining a priori knowledge and helping experimentalists speed up optimization procedures.

### 4.7.2 Dataset

The dataset contains 26 experimental reaction protocols for the synthesis of F3_MIL-140A(Ce). Each entry has 12 variables, as listed below;

- **MeOH:$H_2O$** Ratio methanol/water.
- **CAN:F3BDC molar ratio** Cerium ammonium nitrate and 2,3,5-trifluoroterephthalic acid molar ratio.

[16]Dipartimento di Chimica e Chimica Industriale, Unitá di Ricerca INSTM, Universitá di Pisa, Via Giuseppe Moruzzi 13, 56124 Pisa, Italy

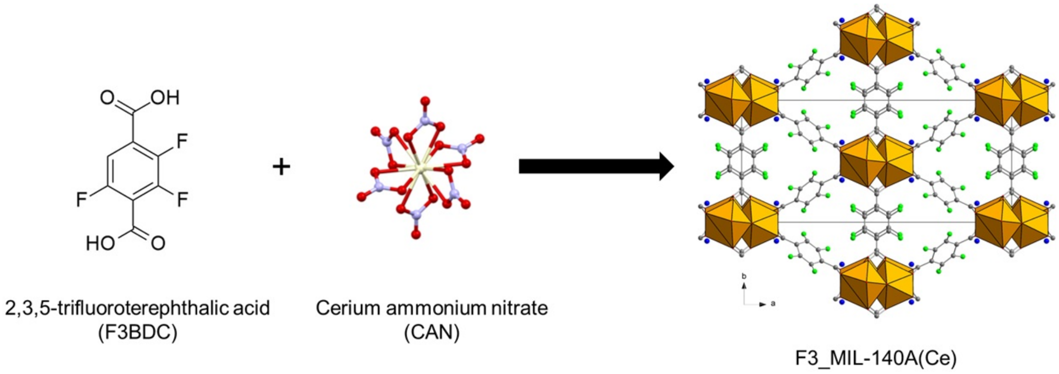


**Figure 90. Schematic representation of the synthesis of MIL-140A.**

- **F3BDC (mmol)** Amount of 2,3,5-trifluoroterephthalic acid.
- **CAN (mmol)** Amount of cerium ammonium nitrate.
- **T (°C)** Temperature of synthesis.
- **$HNO_3$ Equivalents** nitric acid equivalents.
- **V CAN (mL)** Volume of cerium ammonium nitrate solution.
- **V F3BDC (mL)** Volume of 2,3,5-trifluoroterephthalic acid solution.
- **Vfinal (mL)** Total volume.
- **Reaction time** Reaction time with unit included.

A successful synthesis is defined as one that affords at least 20% yield of phase-pure F3_MIL-140A(Ce), as determined by powder X-ray diffraction analysis. This results in a binary classification problem from a slightly unbalanced, rather small, dataset (Figure 91).

Table 40 shows an example of the prompt we use to fine-tune our LLM model.

### 4.7.3 LLM results

With only 26 entries, the provided dataset was rather small. We were thus limited in the training examples. This case study was truly a search for the limits of the capabilities of our approach. The first experiments with 25 epochs with only a few training examples ($n = 10$) were not successful. The models were unable to capture the prompt/completion structure and returned invalid completions for the test data, e.g., '-9223372036854775808' as the completion. The 0% accuracy in the learning curves should, therefore, be interpreted

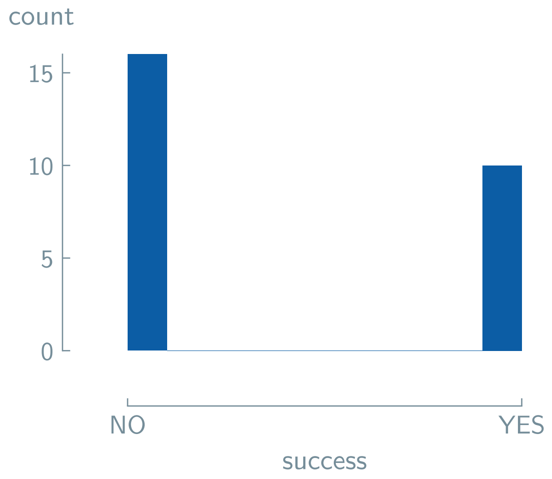


**Figure 91. Histogram of reaction success of 26 experimental reaction protocols for the synthesis of MIL-140A.**

as a result of unsuccessful parsing of the returned completion rather than a completely inaccurate fine-tuning of a model.

Next, we increased the number of epochs to 50, hypothesizing that this would increase the performance of the fine-tuning. Indeed, we have already obtained useful predictions on the test set in a very low data regime. Models trained on only five training examples but repeatably seeing the same data for 50 times, i.e., number of epochs, could capture the subtle relations between reaction conditions and reaction outcome. The average accuracy over 3 seeds was 89%.

In a next step, we screened different models. Three base LLMs, i.e., GPT-J, Llama, and Mistral, were fine-tuned. We notice that a maximum accuracy of 100% was reached with

**Table 40. Example prompts and completions for predicting the success of the synthesis of MIL-140A.** `<reaction conditions>` serves as the placeholder for the available reaction parameters for the synthesis.

| prompt | completion | experimental |
|---|---|---|
| Example of training data | | |
| `What is the success of synthesis of MIL-140A with <reaction conditions>` | 0 | Unsuccessful |
| `What is the success of synthesis of MIL-140A with <reaction conditions>` | 1 | Successful |

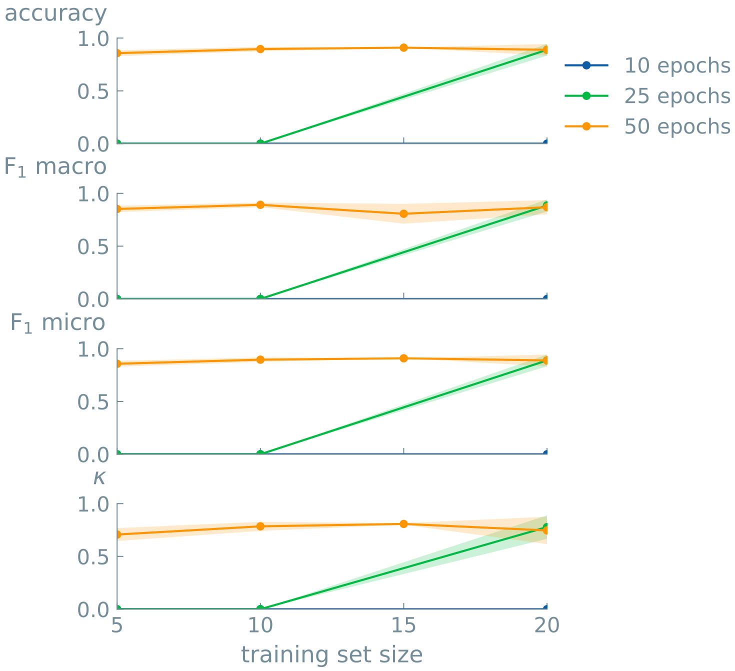


**Figure 92. Learning curve analysis of predictions for the success of the synthesis of MIL-140A.** Models fine-tuned with 10 (blue), 25 (green), and 50 (orange) epochs were validated. A maximum accuracy of 89% was reached for a training set of 20 and 50 epochs.

the GPT-J model (Figure 93 and Table 41). In addition, the fine-tuned LLMs were compared with "traditional" ML models (random forest (RF) and XGBoost). We see that the LLMs can compete with these models.

**Table 41. Overview of results of LLMs and "traditional" ML predicting the binary class of the success of a MOF synthesis.** Three runs were performed to get the metrics average. All LLMs were fine-tuned with 50 epochs and a learning rate of 0.003. For the random forest (RF) and XGBoost models, `optuna` was used for hyperparameter optimization. Maximum performances are highlighted in bold.

| Size | Model | Accuracy | F1 Macro | F1 Micro | Kappa |
|---|---|---|---|---|---|
| 15 | **GPT-J (LLM)** | **1.00** | **1.00** | **1.00** | **1.00** |
| | Llama (LLM) | 0.80 | 0.79 | 0.80 | 0.63 |
| | Mistral (LLM) | 0.93 | 0.92 | 0.93 | 0.85 |
| | RF | 0.80 | 0.78 | 0.80 | 0.61 |
| | **XGBoost** | **1.00** | **1.00** | **1.00** | **1.00** |
| | Zero-rule | 0.50 | 0.50 | 0.50 | 0.00 |

In addition, we tested the best-performing model (GPT-J) on a hold-out dataset that was only sent to us after the models were trained. We performed this extra test to minimize chances of data leakage while maximizing transparency towards the domain experts. Interestingly, the outcome for all ten different models was the same, predicting the correct outcome for 4 out of 5 reaction conditions (Figure 94).

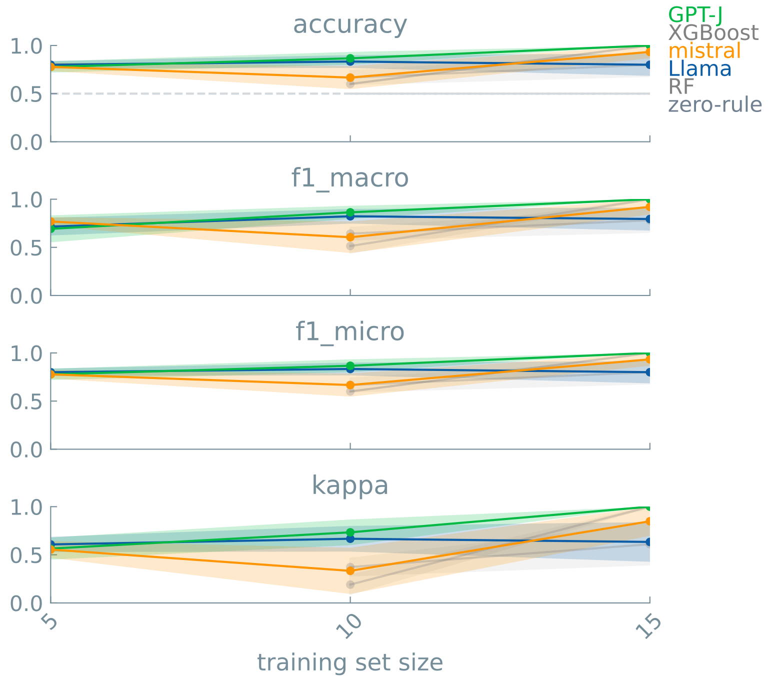


**Figure 93. Learning curve analyses of binary classifications of the success of the catalytic isomerization using different models.** Three LLMs (GPT-J, Llama, and Mistral) were validated on predicting the binary class of the the success of the MOF synthesis. Three runs were performed for each model to get the metric's average and standard deviation.

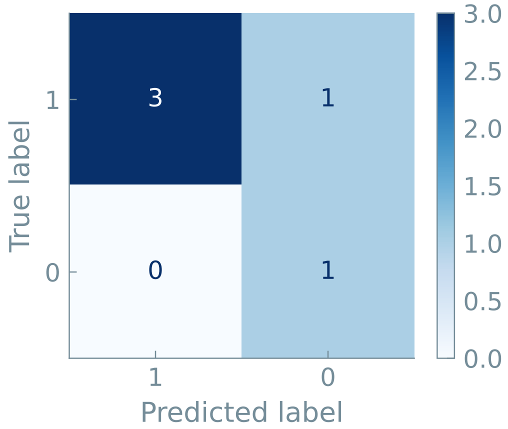


**Figure 94. Confusion matrix of predictions of unseen hold-out data.** GPT-J models were fine-tuned on 20 example prompts with 50 epochs. Ten different seeds all gave the same predictions. The labels '0' and '1' represent the success of the reaction outcome.

# 5 Systems and Applications

This is the section that describes case studies regarding chemical systems and applications.

## 5.1 Gas Uptake and Diffusion of MOFs

The dataset was provided by: Hilal Daglar and Seda Keskin[17]

### 5.1.1 Scientific Background

Metal-organic frameworks (MOFs) have proven to be successful in a plethora of applications.[67] From a synthesis point of view, MOFs consist of a metal part and an organic linker. The endless combinations of metal and ligand make trial-and-error as a strategy to find the best material unsustainable. Computer-aided searches, being molecular simulations or machine learning algorithms, are therefore an important cornerstone in identifying promising MOF structures.[68]

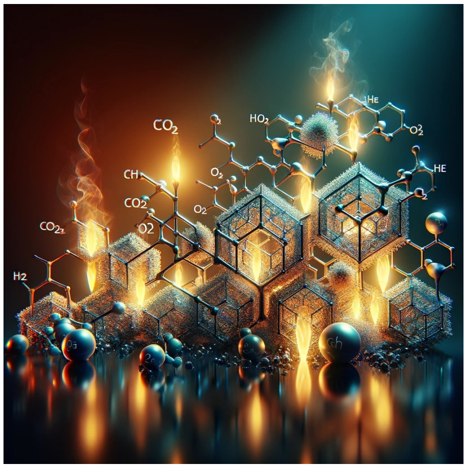


**Figure 95.** AI generated representation of Metal-Organic Frameworks (MOFs) in gas separation.

Their high porosity and large surface-to-volume ratio make this class of materials of great interest for gas separation, especially in natural gas processing. Given the current energy crisis, striving for maximal energy extraction from fuels is paramount. Removing contaminants, such as $CO_2$, is critical in this regard. Today, repeated distillation-compression cycling, cryogenic distillation, and pressure swing adsorption are widely used to achieve this goal. Advanced separation technologies based on porous materials, e.g., MOFs, are seen as an energy-efficient alternative. For a comprehensive overview of various applications, the reader is referred to the review of Li et al.[69].

Important physical metrics to evaluate the gas separation potential of a material are its uptake and diffusion. The ability to predict these properties could prioritize molecular simulations, synthesis, and, eventually, testing of their separation performance. Daglar and Keskin[70] successfully developed several models that could predict uptake and diffusion properties of He, $H_2$, $N_2$, and $CH_4$ gasses in MOFs. Training data consisted of simulated uptake and diffusion data of more than 5,000 MOFs. They used 19 different MOF descriptors, ranging from pore size to chemical composition, to investigate their respective influence on the

[17] Department of Chemical and Biological Engineering, Koç University, Rumelifeneri Yolu, Sariyer, 34450 Istanbul, Turkey

model's performance. These in-depth feature analyses and ML experiments are an excellent starting point to compare with our GPT methodology.

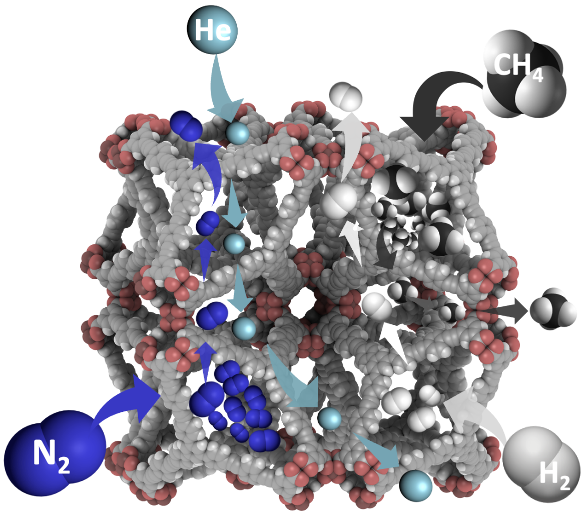


**Figure 96.** Gas uptake and diffusion in a MOF

### 5.1.2 Dataset

The dataset provided by Daglar and Keskin[70] is a subset of the CoRE MOF database.[71] To ensure the accessibility of the molecules studied, the database was filtered on MOFs with >3.8 Å and an accessible surface area (SA) >0 $m^2 g^{-1}$. Molecular dynamics simulations were used to calculate diffusion and uptake for the remaining MOFs. Further filtering of the structures was based on the self-diffusivities, i.e., $>1\times10^{-8}$ $cm^2 s^{-1}$ and statistical outliers for the individual gas molecules. Finally, the dataset contains 677 MOFs for training ML models for He, 2,715 MOFs for $H_2$, 5,215 MOFs for $CH_4$, and 5,224 MOFs for $N_2$ (Figure 97). These were used to train models for both their respective diffusion and uptake. For the base cases, the binary classification was based on the median diffusion and uptake of the MOFs, taken over the complete dataset of the guest molecule.

An example of the prompt template is shown in Table 42.

### 5.1.3 LLM results

**Base Case** In the first model iterations, we predicted the diffusion and uptake class solely from the `mofid` or `mofkey`. The mofid is an identifier that describes the chemistry and topology of MOF structures; the `mofkey` is the hashed version of the `mofid`.[63] Over the 8 test cases, i.e., He, $H_2$, $N_2$, and $CH_4$ uptake and diffusion, averages of 68% and 66% for the mofid and

**Table 42. Example prompts and completions for predicting the uptake or diffusion of MOFs.** `<MOF>` serves as a placeholder for the various descriptors used in this study (see Table 43 for details).

| prompt | completion | experimental |
|---|---|---|
| | Example of training data | |
| `What is the uptake` *or* `diffusion of <MOF>?` | 0 | Low |
| `What is the uptake` *or* `diffusion of <MOF>?` | 1 | High |

mofkey model, respectively, suggested no significant difference between the two representations on average (Table 44). While these are arguably not outstanding models, they still perform better than the baseline of 50%, and thus serve as a good starting point for further investigation.

Interestingly, similar conclusions were drawn from the original regression models, where the atomic structure was a weak predictor for gas diffusion and uptake. Feature correlation analysis revealed higher $CH_4$ diffusion for low-medium carbon percentage and high metal percentage, whereas weaker correlations were seen for He diffusion. As the `mofid` and `mofkey` primarily capture the atoms of the structures, our initial experiments align with the conclusions of Daglar and Keskin[70].

**Adding MOF characteristics** The original dataset contained additional features grouped by their physical/chemical relevance, i.e., pore volume, pore geometry, atom types, and chemical descriptors (Table 43). These features are computed directly from the crystal structure of the MOFs. Hence, we can only conduct a similar analysis from experimental data if the crystal structure is known.

Previous regression experiments were performed by the curators, where the stepwise addition of these groups made up the feature vector. Although the objective of the task is slightly different, i.e., regression versus classification, we opted to try the same methodology and hypothesized that similar trends in accuracy could be spotted. As test cases, we chose He diffusion and $CH_4$ diffusion as they showed interesting examples from the initial experiments.

Table 45 compares our GPT-J classification model and the regression model of Daglar and Keskin[70], where the accuracy and the $R^2$ serve as their respective metrics. At first glance, it might seem that the higher percentage in accuracy versus the regression's $R^2$ value indicates superior models. However, it is important to stress that these absolute values have different statistical meanings and we only use the table to discuss similar 'learning trends'. The regression models predict the uptake and diffusion values (and thus can be any number) from the given feature vector, where from the $R^2$ value of the true and predicted numbers reflects the performance of the individual models. As our GPT-J models address a classifi-

**Table 43. Sets of input variables used to represent the feature vector.**[70]

| group | feature |
|---|---|
| A | largest cavity diameter (Å)<br>pore limiting diameter (Å)<br>pore size ratio |
| B | density ($g/cm^3$)<br>pore volume ($cm^3/g$)<br>porosity<br>surface area ($m^2/g$) |
| C | carbon percentage<br>hydrogen percentage<br>nitrogen percentage<br>oxygen percentage<br>halogen (Br, Cl, F, I) percentage<br>metalloid (As, B, Ge, Te, Sb, Si) percentage<br>ametal (Se, S, P) percentage<br>metal percentage |
| D | total degree of unsaturation<br>degree of unsaturation<br>metallic percentage (#of metal/#of C atoms)<br>oxygen-to-metal ratio |

**Table 44. Overview of accuracies for binary classifications for all test cases.** Three runs were performed to get the metric's average and standard deviation. For all experiments, the training size and number of epochs were set to 500 and 25, respectively. The medians of the respective diffusion and uptake values were used as the threshold for creating the binary classes.

| | He | | $H_2$ | | $N_2$ | | $CH_4$ | |
|---|---|---|---|---|---|---|---|---|
| | Diff. | Uptake | Diff. | Uptake | Diff. | Uptake | Diff. | Uptake |
| **mofid** | | | | | | | | |
| Accuracy | 0.62 | 0.68 | 0.60 | 0.71 | 0.68 | 0.74 | 0.71 | 0.71 |
| Kappa | 0.24 | 0.37 | 0.20 | 0.42 | 0.36 | 0.48 | 0.42 | 0.42 |
| **mofkey** | | | | | | | | |
| Accuracy | 0.63 | 0.76 | 0.59 | 0.64 | 0.62 | 0.70 | 0.64 | 0.69 |
| Kappa | 0.26 | 0.52 | 0.18 | 0.29 | 0.25 | 0.41 | 0.20 | 0.39 |

cation problem (and thus the output can only be '1' or '0' in our case), their performance is assessed with the accuracy, i.e., the percentage of predicting the correct class of the instances.

Having said this, a first striking result can be seen for the He diffusion GPT-J model with only the pore volume parameters, i.e., group A (Table 43). Compared to the `mofid` or `mofkey` models from Table 44, there is a significant increase in accuracy, underpinning the learning capability of these models. Moreover, the accuracy increases upon adding more descriptors, a trend that can also be noted in the regression models. Also, for the $CH_4$ diffusion models, the model based on only group A is the worst for classification and regression. Adding more features increases the performance. Moreover, for both GPT-J test cases, the combination of groups A and B as a feature vector outperforms models based solely on one feature group. We further tested different LLMs in predicting the binary class of the He diffusion. For this comparison, we used the full feature vector, i.e. ABCD feature. We saw very comparable accuracies around 70%, also with traditional ML models (Figure 98).

**Table 45. Overview of main performance metrics.** The table shows the accuracy and $R^2$ value for helium diffusion and $CH_4$ diffusion for the binary classification GPT-J and regression models, respectively. Different descriptors were gradually added to the feature vector to investigate the influence of the MOF representation. In both GPT-J series, the median diffusion was used as the threshold for creating the binary classes.

| | He Diffusion | | $CH_4$ Diffusion | |
|---|---|---|---|---|
| | GPT-J | Regression | GPT-J | Regression |
| A | 68 | 41 | 66 | 31 |
| B | 62 | / | 72 | / |
| AB | 73 | 63 | 80 | 72 |
| ABC | 77 | 64 | 78 | 77 |
| ABCD | 70 | 65 | 81 | 78 |

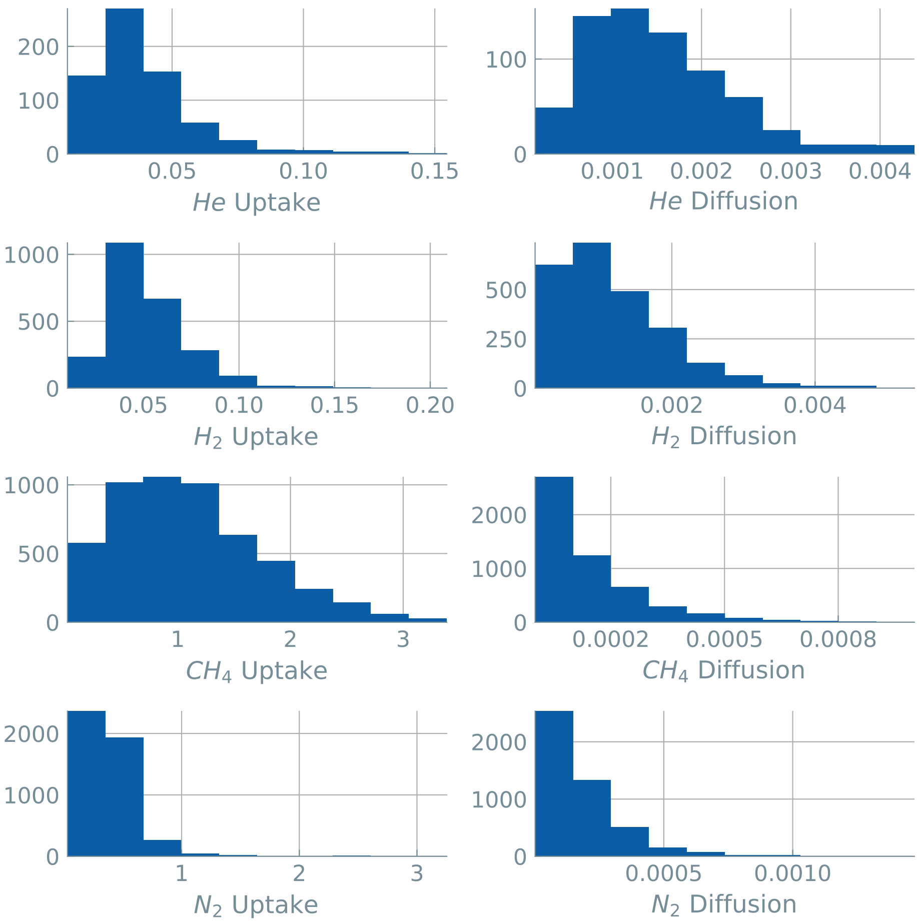


**Figure 97. Histograms of the eight properties studied, i.e., MOF uptake and diffusion for helium, hydrogen, methane, and nitrogen.**

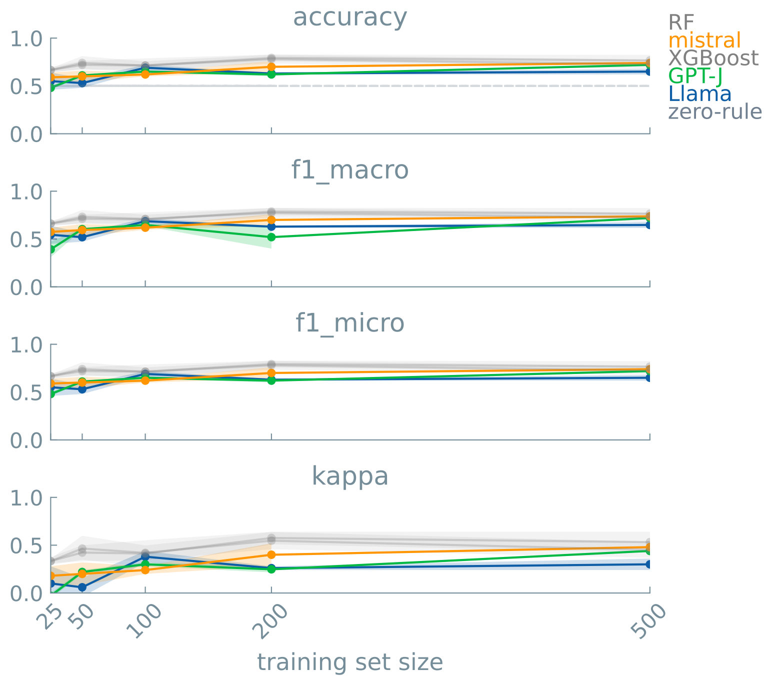


**Figure 98. Learning curve analyses of binary classification of the He diffusion using different models.** Three LLMs (GPT-J, Llama, and Mistral) and two traditional ML models (XGBoost and random forest (RF)) were validated on predicting the binary class of the He diffusion. We used 50% as a random guess accuracy (dashed line), representing the zero rule baseline, For each model, three runs were performed to get the metric's average and standard deviation. The fine-tuned Mistral model reached the maximum accuracy of 72% (training set size of 500 and 25 epochs).

## 5.2 Hydrogen Storage Capacity of Metal Hydrides

The dataset was provided by: Noémie Xiao Hu[18] and Andreas Züttel[19]

### 5.2.1 Scientific Background

Hydrogen is an interesting energy source with double the energy storage capacity of most conventional fuels.[72,73] However, the low density of hydrogen makes efficient storage challenging, from a practical side, but also regarding safety and costs. Potential solutions include pressurized gas, cryogenic liquid, or absorption to solid-state materials. The latter proves promising mainly because of their ab- and absorption reversibility and high hydrogen storage capacity. Within the class of solid-state materials, promising candidates come from metals that form metal hydrides upon the chemisorption of hydrogen.[74]

The search for an ideal metal hydride for hydrogen absorption is dual. The structure formed should be stable to prevent any hydrogen leakage during storage. On the other hand, the reverse reaction, i.e., desorption, needs to be easily provoked to effectively access the stored hydrogen. In other words, optimal stability is key to ensuring the most desirable adsorption-desorption process.

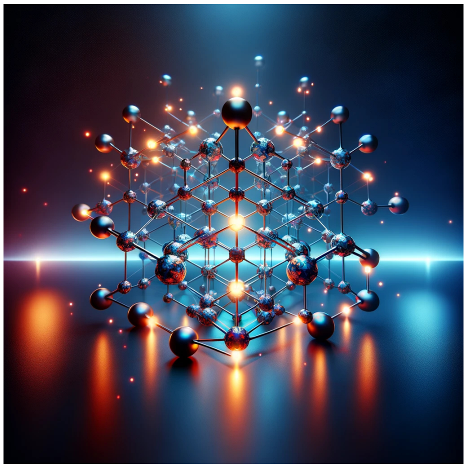

**Figure 99.** AI generated representation of hydrogen storage using metal hydrides.

The ability to predict the heat of absorption for a system could prioritize experimental validation and is, therefore, considered highly relevant in the endeavor to establish a sustainable hydrogen economy. Several models, both theoretical and (semi)-empirical, have been proposed. A notable example is the semi-empirical model of Griessen and Riesterer[75] based on the quantum mechanical parameters, i.e., Fermi energy. A more recent work was published by Witman et al.[76]. Using machine learning, they predicted the equilibrium pressure, i.e., how much hydrogen a material can deliver at room temperature. As there is a clear link between the equilibrium pressure and the heat of formation, our methodology serves as a useful tool for finding suitable hydrogen storage materials.

[18] Laboratory of molecular simulation (LSMO), Institut des Sciences et Ingénierie Chimiques, École Polytechnique Fédérale de Lausanne (EPFL), Rue de l'Industrie 17, CH-1951 Sion, Switzerland

[19] Laboratory of Materials for Renewable Energy (LMER), Institut des Sciences et Ingénierie Chimiques, École Polytechnique Fédérale de Lausanne (EPFL), Rue de l'Industrie 17, CH-1951 Sion, Switzerland

### 5.2.2 Dataset

The ML-HydPARK dataset created by Witman et al.[77] was used to train the models. This open-access database is a subset (<20%) of the HYDPARK database. Besides the metal hydride composition, each entry contains the equilibrium pressure (at 25 °C) and its heat of formation.

Considering the variety of heat of formation ranges in literature, the values between $-40\,\mathrm{kJ\,mol^{-1}}$ to $-20\,\mathrm{kJ\,mol^{-1}}$ are labeled as 'optimal' for all our experiments. This criterion results in 255 potential candidates (≈60% of the dataset) for hydrogen storage applications. The remaining structures, with heat of formation values both $>-20\,\mathrm{kJ\,mol^{-1}}$ or $<-40\,\mathrm{kJ\,mol^{-1}}$, are classified as 'sub ideal'.

The aforementioned correlation between the natural logarithm of the equilibrium pressure and heat of formation is illustrated in Figure 100. An elemental analysis of the structures is shown in Figure 101, where the bars' height and color scheme represent the element's absolute presence in the structures and the heat of formation binning, respectively. For this plot, the stoichiometry of the structures was neglected. For instance, $ZrFe_{1.8}Ni_{0.2}$ and $Mg_2Co_{0.25}Ni_{0.75}$ are both counted as 'Ni' containing structures. As the heat of formation is often indecisive within one element, predicting the hydrogen storage potential via the structural composition is a complex task. Table 46 shows an example of the prompt used to fine-tune the LLMs in our experiments.

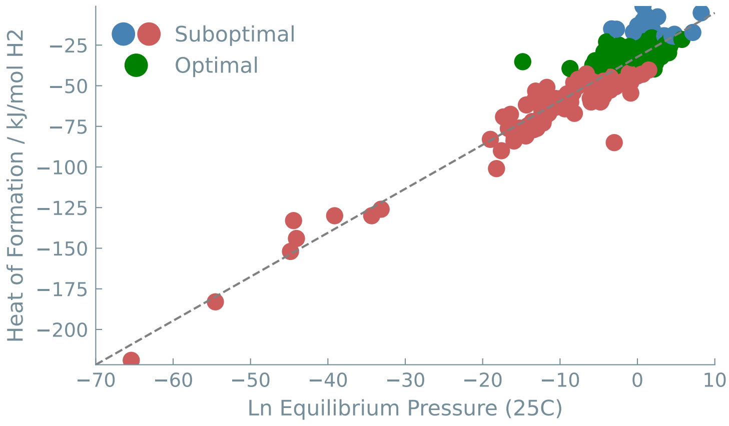


**Figure 100. Equilibrium pressure vs. heat of formation.** Structures with heat of formation between $-40\,\mathrm{kJ\,mol^{-1}}$ to $-20\,\mathrm{kJ\,mol^{-1}}$ were considered optimal.

### 5.2.3 LLM results

**Base Case** In the first binary classification problem, we predicted the binary class of the heat of formation value, i.e., lower or higher than the median of $-34.95\,\mathrm{kJ\,mol^{-1}}$, based only

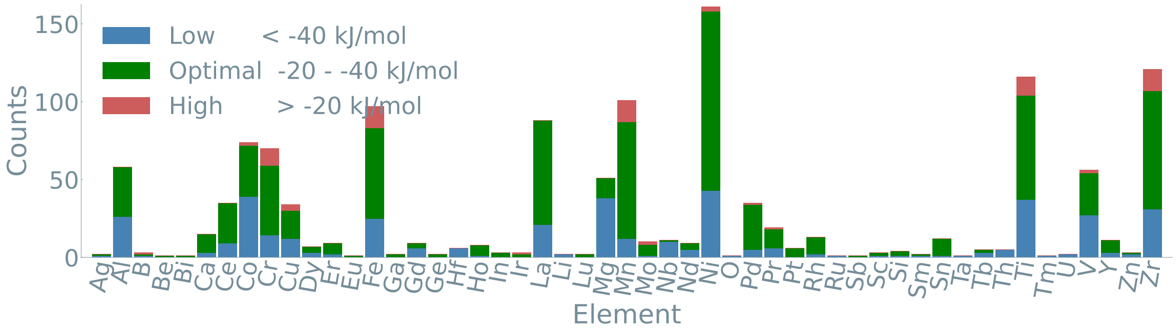


**Figure 101. Elemental analysis of the ML-HydPARK dataset.**

**Table 46. Example prompts and completions for predicting the heat of formation of hydrides.** `<Formula>` serves as a placeholder for the chemical formula of the hydride.

| prompt | completion | experimental |
|---|---|---|
| | Example of training data | |
| `What is the heat of formation of <Formula>` *`and/or`* `<Pressure>?` | 0 | Low |
| `What is the heat of formation of <Formula>` *`and/or`* `<Pressure>?` | 1 | High |

on the composition formula.

Intuitively, a trained chemist can try to interpolate the success of the material based on the chemical composition. For instance, if the material has element X, it will likely be better than a material containing element Y. However, for non-experts, this is often not as straightforward. One potential strategy that could be used is taking the dominant element of each material and accepting the dominant bin as the predicted outcome. For instance, asking a non-expert the success of the material $Zr_{0.2}Ti_{0.8}Cr_{1.8}$, Cr, as the dominant element, can naively be taken as the main feature, and looking at the elemental analysis, Figure 101 suggest that the material is likely optimal. Nevertheless, this analysis results in a predictive accuracy of 58%, further suggesting that a non-expert eye is not sufficient for material selection. On the other hand, our LLM approach allows for easy input of textual strings, so we can train the model on the full elemental composition. So rather than Cr, we now use the complete $Zr_{0.2}Ti_{0.8}Cr_{1.8}$ as the feature to predict the heat of formation. Fine-tuning the LLM exceeds the previous baseline of 58% by quite some margin, with an accuracy of 84.6%, suggesting that the model learns the subtle correlations in the composition of the materials (Table 47).

Similarly, we can create a 'lazy' baseline model with the equilibrium pressure as the only

feature by binning the outcome of a linear regression. For example, a ln($P$) of -10 would give a heat of formation of $-59\,\mathrm{kJ\,mol^{-1}}$, which is lower than the median of $-34.95\,\mathrm{kJ\,mol^{-1}}$, so binned as 'sub ideal.' An accuracy of 80.3% is calculated via this method. Again, if we train our models on only the equilibrium pressure, we get an accuracy of 76.0%, confirming the learning ability of these LLMs.

Lastly, if we combine both features and train the model on '`<composition name>` (with an equilibrium pressure of `<pressure>`),' no additional improvement is observed, with an accuracy of 78.6% (Figure 102).

**Table 47. Binary classification for the heat of formation.** All models (GPT-J) were trained on 350 examples and 50 epochs. The median was the threshold for the class split.

| Feature(s) | Accuracy / % |
|---|---|
| Formula | 84.6 |
| Pressure | 76.0 |
| Formula + Pressure | 78.6 |

We also compared the performance of three LLMs, i.e, GPT-J, Llama, and Mistral. We notice that all three models are comparable in performance. A maximum accuracy of 86% was reached with the Llama model (Figure 102 and Table 48).

**Table 48. Overview of results of LLMs predicting the binary class of the heat of formation of hydrides.** Three runs were performed to get the metrics average. All LLMs were fine-tuned with 25 epochs and a learning rate of 0.003. Maximum performances are highlighted in bold.

| **Size** | **Model** | **Accuracy** | **F1 Macro** | **F1 Micro** | **Kappa** |
|---|---|---|---|---|---|
| 350 | GPT-J (LLM) | 0.79 | 0.79 | 0.79 | 0.57 |
| | **Llama (LLM)** | **0.86** | **0.86** | **0.86** | **0.72** |
| | Mistral (LLM) | 0.79 | 0.79 | 0.79 | 0.59 |
| | Zero-rule | 0.50 | 0.50 | 0.50 | 0.00 |

**Real split** Next, a series of models were trained on the more realistic threshold. Here, only materials with heat of formation values between $-40\,\mathrm{kJ\,mol^{-1}}$ to $-20\,\mathrm{kJ\,mol^{-1}}$ are labeled 'ideal.' Lower or higher values are labeled 'sub ideal,' so a binary classification is preserved. These threshold values where taking based on suggestions in reported literature.[78,79] The split gives a slightly imbalanced dataset, with 60% 'sub ideal' cases, which therefore serves

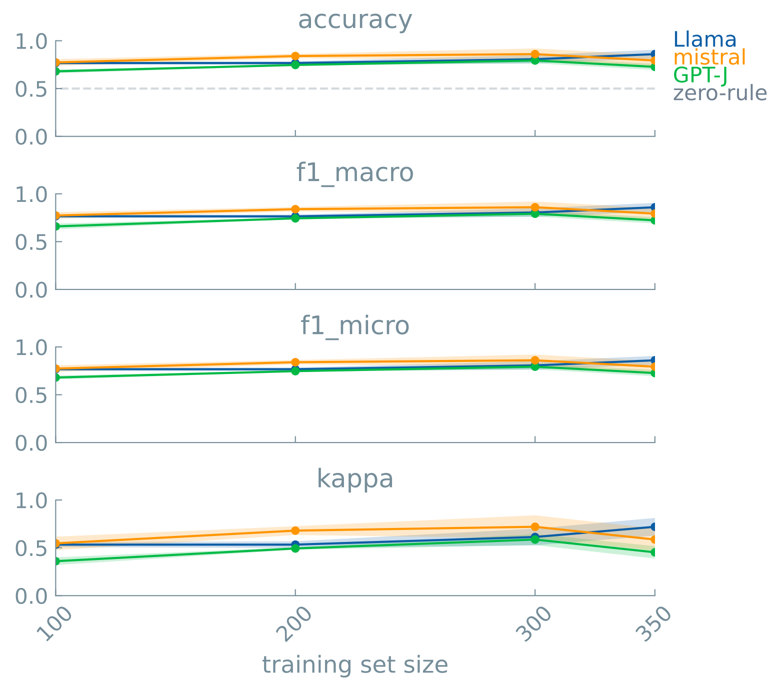


**Figure 102. Learning curve analyses of binary classification of the heat of formation of hydrides using different models.** Three LLMs (GPT-J, Llama, and Mistral) were validated on predicting the binary class of the heat of formation of hydrides. For each model, three runs were performed to get the metric's average and standard deviation. The fine-tuned Llama model reached the maximum accuracy of 86% (training set size of 350 and 50 epochs).

as our baseline. 'Lazy' predictions on the dominant element and with the regression on the equilibrium pressure binning give accuracies of 61.4% and 81%, respectively. The fine-tuning of LLMs for both the formula and the pressure as a single feature gives an accuracy of 70%. The combined feature vector, i.e., formula and pressure, does not significantly increase the performance of the models, i.e., an accuracy of 74.6% (Table 49). While this result is slightly worse than the regression on the equilibrium pressure, it still makes superior predictions from the elemental composition of the materials. As a non-expert, this situation can be of practical relevance as equilibrium pressure values are not always reported/accessible.

**Table 49. Binary classification for the heat of formation trained on a realistic split.** All models were trained on 300 examples and 50 epochs. Reported accuracies are averages of 3 individual runs. Values between $-40\,\mathrm{kJ\,mol^{-1}}$ to $-20\,\mathrm{kJ\,mol^{-1}}$ were labeled as 'optimal'; higher or lower heat of formation values were 'sub ideal'.

| Feature(s) | Accuracy / % |
|---|---|
| Formula | 70.6 |
| Pressure | 71.3 |
| Formula + Pressure | 74.6 |

## 5.3 Carbondioxide Adsorption of Biomass-derived Adsorbents

The dataset was provided by: Hossein Mashhadimoslem[20]

### 5.3.1 Scientific Background

Biomass-derived activated carbons for $CO_2$ capture in industry are a sustainable pathway to carbon neutrality. Although searching for microporous carbon adsorbents suitable as $CO_2$ adsorbents has been a relevant research topic for many years, large-scale deployment of bio-based adsorption processes still faces scientific and technological challenges.[80–82]

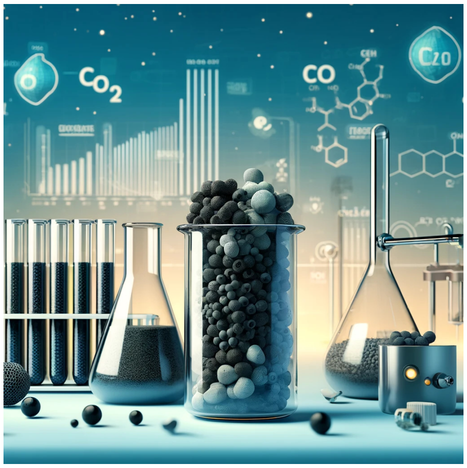


**Figure 103.** AI generated representation of biomass-derived activated carbons for $CO_2$ capture.

Promising attributes of carbon-based adsorbents include their low cost, high surface area, high ability to modify pore structure and functionalize the surface, and relative ease of regeneration.[83] However, implementing adsorption-based $CO_2$ capture on an industrial scale requires a material that can adequately handle real flue gas conditions. Due to their hydrophobic nature, carbon adsorbents exhibit high stability under wet conditions, making them promising candidates for post-combustion $CO_2$ capture applications. In addition, bio-based adsorbents have recently received considerable attention as sustainable and cost-effective materials for $CO_2$ capture, as they can be developed from renewable sources that are available worldwide at lower cost through relatively simple treatment processes.

The production of carbon adsorbents from biomass precursors involves physical or chemical activation to develop porosity through the reaction of the precursor with the activating agent.[84] The adsorption capacity of an activated carbon is mainly dependent on its pore structure. Every carbon precursor requires specific activation conditions. Since laboratory experiments are time-consuming and expensive, a model to predict the textural properties, such as BET surface area, as well as $CO_2$ adsorption capacity, of biomass-based activated carbons, could accelerate the development of adsorption processes on bio-derived adsorbents by helping to synthesize efficient adsorbents for $CO_2$ capture.

In this work, we use our LLMs approach to build a machine learning model to predict the BET surface area of a biomass-derived activated carbon from the biomass precursor and activation conditions. Likewise, we develop a model to predict the $CO_2$ adsorption capacity

[20]Department of Chemical Engineering, University of Waterloo, Waterloo, N2L3G1, Canada

of a biomass-derived activated carbon from the biomass precursor, activation conditions, textural properties, and adsorption conditions.

### 5.3.2 Dataset

We used the dataset collected by Mashhadimoslem et al.[85] on the synthesis of activated carbons from different biomass precursors for $CO_2$ adsorption. The dataset contains 33 biomass precursors and ten activating agents used to synthesize different activated carbons. These biomass-derived adsorbents are tested for $CO_2$ adsorption under varying pressure and temperature conditions, resulting in a dataset of 74 data points for BET surface area and 421 data points for $CO_2$ adsorption. The dataset contains information about the precursor activation process (biomass precursor, activating agent, activating agent/carbon weight ratio, and activation temperature) and adsorption conditions (pressure and temperature), along with the textural properties of the activated carbon (BET surface area and pore volume) and its $CO_2$ adsorption capacity.

We predict the BET surface area and $CO_2$ adsorption capacity of biomass-derived activated carbons. The distribution of the two variables is shown in Figure 104. We used the biomass precursor name, activating agent, activation agent/carbon ratio, and activation temperature as inputs to predict the BET surface area. To predict the $CO_2$ adsorption capacity of a biomass-derived activated carbon, we used as inputs the biomass precursor, activating agent, activating agent/carbon ratio, activation temperature, BET surface area, pore volume, adsorption pressure, and adsorption temperature.

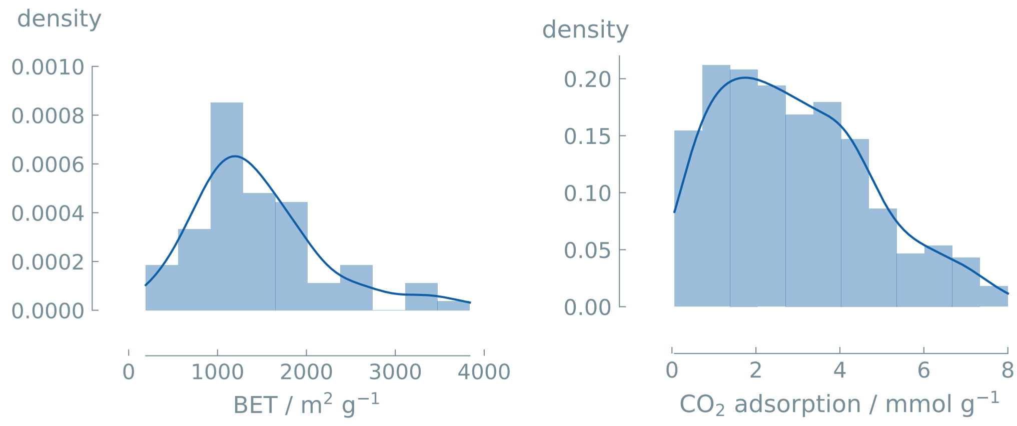


(a) (b)

**Figure 104. Distribution of the of the BET surface area (a) and $CO_2$ adsorption capacity (b) values in the dataset.** The median BET surface area is 1262 $m^2 g^{-1}$ and the median $CO_2$ adsorption capacity is 2.7 $mmol\,g^{-1}$.

We used a simple prompt template, with prompts of the form shown in Table 50 for

experiments to predict BET surface area and $CO_2$ adsorption capacity.

**Table 50. Example prompts and completions for predicting the BET surface area and $CO_2$ adsorption capacity.**

| prompt | completion | experimental |
|---|---|---|
| What is the BET surface area (m2/g) of an activated carbon synthesized from <biomass precursor> as precursor, activated with <activating agent> with an activating agent/carbon weight ratio of <activating agent/carbon ratio> at a temperature of <activation temperature> K? | 0 | Low |
| What is the BET surface area (m2/g) of an activated carbon synthesized from <biomass precursor> as precursor, activated with <activating agent> with an activating agent/carbon weight ratio of <activating agent/carbon ratio> at a temperature of <activation temperature> K? | 1 | High |
| What is the $CO_2$ adsorption capacity (mmol/g) of an activated carbon at <adsorption temperature> K and <adsorption pressure> bar, which has been synthesized from <biomass precursor> as precursor, activated with <activating agent> with an activating agent/carbon weight ratio of <activating agent/carbon ratio> at a temperature of <activation temperature> K, and has a BET surface area of <BET surface area> m2/g and a pore volume of <pore volume> cm3/g? | 0 | Low |
| What is the $CO_2$ adsorption capacity (mmol/g) of an activated carbon at <adsorption temperature> K and <adsorption pressure> bar, which has been synthesized from <biomass precursor> as precursor, activated with <activating agent> with an activating agent/carbon weight ratio of <activating agent/carbon ratio> at a temperature of <activation temperature> K, and has a BET surface area of <BET surface area> m2/g and a pore volume of <pore volume> cm3/g? | 1 | High |

### 5.3.3 LLM results

**BET surface - Base Case** To train the binary classification models, we split the dataset into two classes of equal size based on the BET surface area separated by the median, i.e., $1262\,m^2\,g^{-1}$. For this dataset, we tested 30 and 60 fine-tuning epochs with the GPT-J model, but they provided a high number of invalid predictions. Therefore, we tested more epochs, as shown in Figure 105. We find that the models trained with 140 and 200 epochs perform better than random guess (shown by the dashed line), with an accuracy of 72% for a training set of 65 data points and 140 epochs.

Therefore, three base LLMs, i.e., GPT-J, Llama, and Mistral, were fine-tuned using 140 epochs. We also trained two "traditional" ML models, i.e., XGBoost and random forest (RF), for comparison purposes. Table 51 and Figure 106 show that the highest accuracy (76%) was obtained with LLama. Only slightly lower performance values were obtained with other

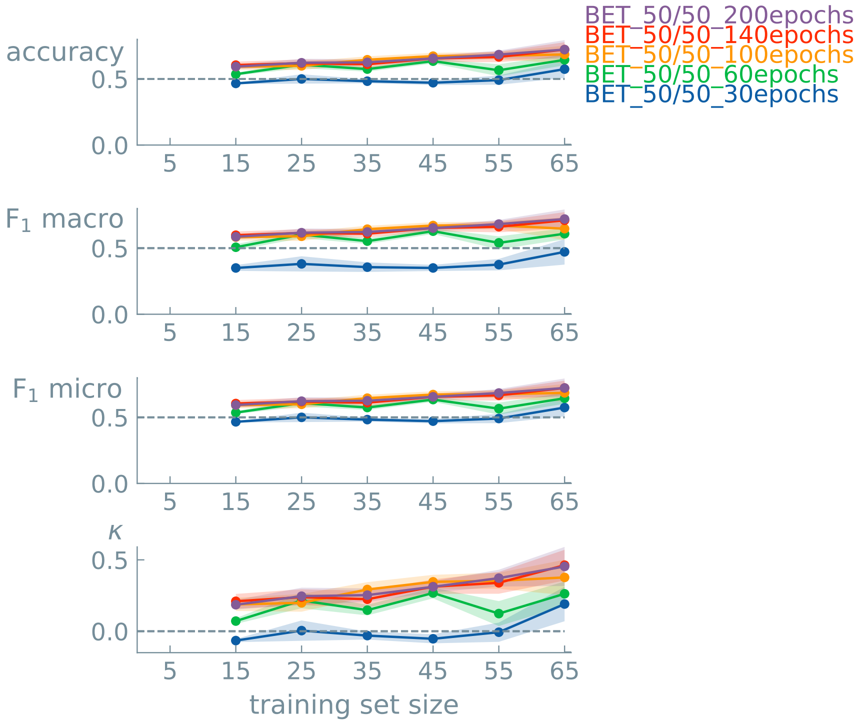


**Figure 105. Learning curves for binary classification GPT-J models (balanced classes) for the BET surface area of biomass-derived activated carbons fine-tuned with different number of epochs.** Data points indicate the mean value of five different experiments. Error bands show the standard error of the mean. We used 0.50 as random guess accuracy (dashed line), which represents the zero rule baseline, i.e., a model that always predicts the most common class. Accuracy = 0.722±0.056 (epochs = 140, learning rate = 0.0003, training set size = 65 data points).

models (accuracy of 72-73%), with a slightly lower accuracy value in the case of XGBoost (68%).

As an example, Figure 107 shows an averaged (over five independent runs) normalized confusion matrix for the GPT-J model trained using a training set of 65 data points and 140 epochs. We can see that the model fails more often in predicting samples with a high value of BET surface area.

**$CO_2$ adsorption capacity - Base Case** We also split the dataset into two classes of equal size based on the $CO_2$ adsorption capacity separated by the median, i.e., 2.7 mmol g$^{-1}$. We fine-tuned three LLMs (GPT-J, Llama, and Mistral) and two "traditional" ML models (XGBoost and RF) with the $CO_2$ adsorption capacity dataset using 30 epochs. Table 52 and Figure 108

**Table 51. Overview of the accuracy results of LLMs and "traditional" ML models for binary classification (balanced classes) of the BET surface area of biomass-derived activated carbons.** Five runs were performed to get the metrics average. LLMs were fine-tuned with 140 epochs and a learning rate of 0.0003. Maximum performances are highlighted in bold.

| Size | Model | Accuracy | F1 Macro | F1 Micro | Kappa |
| --- | --- | --- | --- | --- | --- |
| 65 | GPT-J (LLM) | 0.72 | 0.71 | 0.72 | 0.46 |
| | **Llama (LLM)** | **0.76** | **0.75** | **0.76** | **0.52** |
| | Mistral (LLM) | 0.72 | 0.71 | 0.72 | 0.45 |
| | RF | 0.73 | 0.73 | 0.73 | 0.47 |
| | XGBoost | 0.68 | 0.64 | 0.68 | 0.32 |
| | Zero-rule | 0.50 | 0.50 | 0.50 | 0.00 |

show the results for binary classification of the $CO_2$ adsorption capacity. The trained models perform much better than random guess (shown by the dashed line) for this variable. The highest accuracy (93%) was obtained with XGBoost. However, only slightly lower performance values were obtained with RF (92%) and Mistral (90%) for training sets of 380 data points.

**Table 52. Overview of the accuracy results of LLMs and "traditional" ML models for binary classification (balanced classes) of the $CO_2$ adsorption capacity of biomass-derived activated carbons.** Five runs were performed to get the metrics average. LLMs were fine-tuned with 30 epochs and a learning rate of 0.0003. Maximum performances are highlighted in bold.

| Size | Model | Accuracy | F1 Macro | F1 Micro | Kappa |
| --- | --- | --- | --- | --- | --- |
| 380 | GPT-J (LLM) | 0.84 | 0.84 | 0.84 | 0.69 |
| | Llama (LLM) | 0.83 | 0.83 | 0.83 | 0.66 |
| | Mistral (LLM) | 0.90 | 0.90 | 0.90 | 0.80 |
| | RF | 0.92 | 0.92 | 0.92 | 0.83 |
| | **XGBoost** | **0.93** | **0.93** | **0.93** | **0.87** |
| | Zero-rule | 0.50 | 0.50 | 0.50 | 0.00 |

As an example, Figure 109 shows an averaged (over five independent runs) normalized confusion matrix for the GPT-J model trained using a training set of 380 data points and 30 epochs. We see that the model can predict the two classes in the dataset quite well, although it fails more often in predicting samples with a low $CO_2$ capacity.

**BET surface area - Real Split** To estimate if the activation procedure of a biomass precursor can produce an activated carbon with a very high specific surface area, a binary classification GPT-J model was trained to predict whether the BET surface area is lower or higher than $1800\,m^2\,g^{-1}$. This implies using an unbalanced dataset since the data points with such a high BET surface area represent only 26% of our overall dataset.

Figure 110 shows that this model does not perform much better than random guess (shown by the dashed line), obtaining an accuracy of 86% when using a training set of 65 data points and 140 epochs.

Three LLMs (GPT-J, Llama, and Mistral) and two "traditional" ML models (XGBoost and RF) were also fine-tuned with an unbalanced dataset using 140 epochs. Figure 111 shows similar accuracy values for the LLMs and RF (80-86%) and a lower accuracy for XGBoost (76%) when trained using an unbalanced dataset.

As an example, Figure 112a shows an averaged (over five independent runs) normalized confusion matrix for the GPT-Jmodel trained with this unbalanced dataset using a training set of 65 data points and 140 epochs. We can see that the model clearly fails to predict high values of the BET surface area (i.e., label = 1), which is the least represented class in the dataset.

However, we obtained better predictions of the BET surface area values higher than $1800\,m^2\,g^{-1}$ when we used a balanced dataset created by randomly undersampling the majority class (label = 0) at the cost of reducing the size of the dataset. An accuracy of 75% was achieved using a training set of 30 data points and 200 fine-tuning epochs, similar to that obtained for the initial balanced 50/50% dataset. The normalized confusion matrix in Figure 112b shows better results were obtained with a smaller balanced dataset. We can deduce that we need more fine-tuning epochs to obtain better results than random guess when our dataset is smaller.

**$CO_2$ adsorption capacity - Real Split** We also trained a model to predict whether the $CO_2$ adsorption capacity of a biomass-derived adsorbent is lower or higher than $4\,mmol\,g^{-1}$, i.e., if it has a very high adsorption capacity. We also used an unbalanced dataset since the data points with such a high $CO_2$ adsorption capacity represent only 28% of our overall dataset. We fine-tuned three LLMs (GPT-J, Llama, and Mistral) and two "traditional" ML models (XGBoost and RF) with the $CO_2$ adsorption capacity dataset using 30 epochs. Figure 113 show that GPT-J does not perform better than random guess (shown by the dashed line), obtaining an accuracy of 74%. However, other models perform better than random guess for this variable. The highest accuracy (91%) was obtained with Mistral and RF, but high accuracy values were also obtained with Llama (86%) and XGBoost (89%) for training sets of 380 data points.

As an example, Figure 114a shows an averaged (over five independent runs) normalized confusion matrix for the GPT-J model trained with this unbalanced dataset using a training set of 380 data points and 30 epochs. The predictions are close to the random guess.

As in the case of the BET surface area, we also obtained better predictions of the $CO_2$ adsorption capacity values higher than $4\,\mathrm{mmol\,g^{-1}}$ when we used a balanced dataset created by undersampling the majority class (label = 0) (also reducing the size of the dataset). We needed to increase the number of fine-tuning epochs to obtain valid predictions. Thus, an accuracy of 82% was achieved using a training set of 200 data points and 100 epochs, which is similar to the accuracy obtained for the initial balanced 50/50% dataset. The normalized confusion matrix in Figure 114b shows better results obtained with a smaller balanced dataset if more fine-tuning epochs are used. We can see that the model can predict the two classes in the dataset quite well.

Given the promising results obtained for binary classification for the $CO_2$ adsorption capacity, we also trained classification models using datasets split into 4 and 10 bins and regression models using datasets with continuous values. To perform classification into a higher number of bins, we split the dataset into four and ten equally sized bins. For regression, we use the regression approach of our original article,[9] i.e., direct text completion of rounded figures. In this case, a random train/test data split stratified on the target variable was applied using a threshold of $5\,\mathrm{mmol\,g^{-1}}$ for the $CO_2$ adsorption capacity.

Figures 115 and 116 show a performance clearly above the random guess (shown by the dashed line) for both four-class and ten-class classification, respectively. In these cases, the LLM models show a slightly higher performance than RF and XGBoost. As an example, Figure 117 shows that GPT-J model provide good predictions for datasets with 4 (Figure 117a) and 10 (Figure 117b) classes since the majority of them are in the diagonal of the confusion matrix.

Finally, Figure 118 shows that the regression model also performs well in predicting $CO_2$ adsorption capacity when using a training size of 380 data points. In this case, the LLMs show an accuracy similar to that obtained with RF. However, a slightly better performance is obtained with XGBoost. In this case, unlike RF and XGBoost model, where the biomass precursor names and the activating agent's chemical formulas were encoded to numerical values, since LLMs allow text input, we trained the models using the biomass precursor's name and the activating agent's chemical formula an input variables.

For the prediction of $CO_2$ adsorption capacity, we also compare the regression GPT-J model trained with three different sets of input variables, as shown in Table 53. Figure 119 shows no relevant difference in the prediction of $CO_2$ adsorption capacity for the different sets of input variables for training sizes above 200 data points. Maximum $R^2$ values of 0.83–0.87 were obtained for a training set size of 380. This means that the fine-tuned GPT-J 6B model can successfully predict the $CO_2$ adsorption capacity of biomass-derived activated carbons from two different sets of input variables: (i) the textural properties of the activated carbon and the adsorption temperature and pressure, and (ii) the activation conditions of the biomass precursor together with the adsorption conditions. This is an interesting finding, as it means that we could estimate the $CO_2$ adsorption capacity of a biomass-derived adsorbent at selected adsorption conditions before synthesizing it, just by knowing the characteristics of the precursor and activation conditions.

**Table 53. Sets of input variables used to predict the $CO_2$ adsorption capacity of biomass-derived activated carbons using regression models.**

| input variables | all_inputs | wo_BETpore | only_BETpore |
|---|---|---|---|
| biomass precursor | X | X | |
| activating agent | X | X | |
| activating agent/carbon ratio | X | X | |
| activation temperature | X | X | |
| BET surface area | X | | X |
| pore volume | X | | X |
| adsorption pressure | X | X | X |
| adsorption temperature | X | X | X |

In addition, Figure 119 also shows the performance of the regression model after removing the precursor name in the case of the "all_inputs" and "wo_BETpore" sets of input variables ("all_inputs_woNAME" and "wo_BETporeNAME" lines, respectively). We can see that the performance is only slightly lower if the precursor name is not included within the input variables. This indicates that the model could learn some trends associated with the biomass precursor name to some certain extent, although its influence is low.

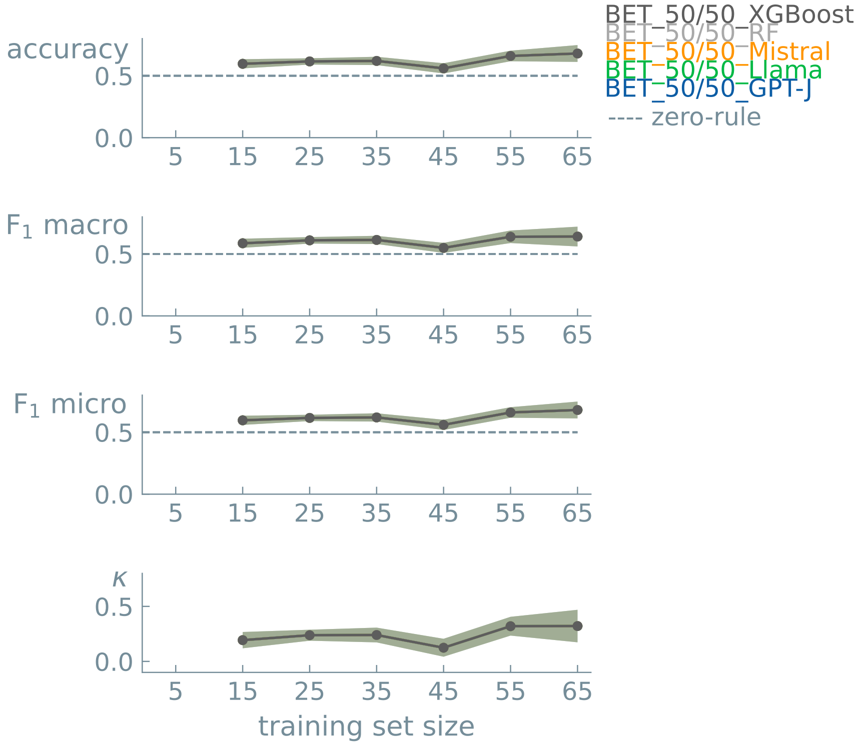


**Figure 106. Learning curves for binary classification models (balanced classes) for the BET surface area of biomass-derived activated carbons fine-tuned with different number of epochs.** Data points indicate the mean value of five different experiments. Error bands show the standard error of the mean. We used 0.50 as random guess accuracy (dashed line), which represents the zero rule baseline, i.e., a model that always predicts the most common class. Accuracy: GPT-J=0.722±0.056, Llama=0.759±0.053, Mistral=0.722±0.056, random forest=0.733±0.044, XGBoost=0.680±0.068 (LLM epochs = 140, LLM learning rate = 0.0003, random forest and XGBoost=default parameters, training set size = 65 data points).

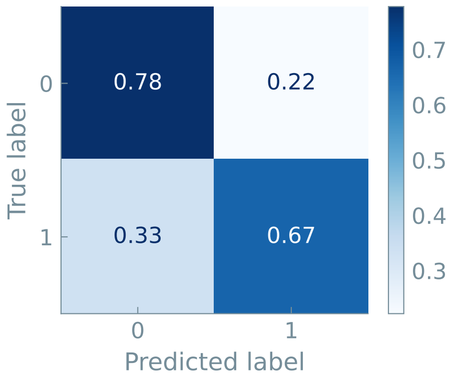


**Figure 107. Normalized confusion matrix, averaged over five independent runs, on the holdout test data for BET surface area prediction with the GPT-J model.** Models were trained using a training set of 65 data points and 140 epochs (accuracy = 72%).

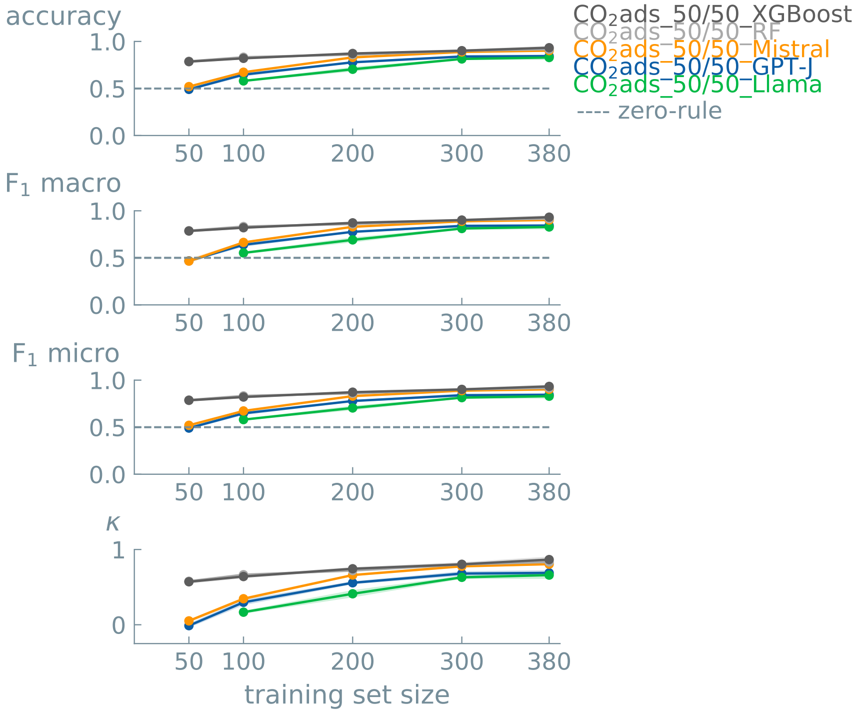


**Figure 108. Learning curves for binary classification models (balanced classes) for the $CO_2$ adsorption capacity of biomass-derived activated carbons.** Data points indicate the mean value of five different experiments. Error bands show the standard error of the mean. We used 0.50 as random guess accuracy (dashed line), which represents the zero rule baseline, i.e., a model that always predicts the most common class. Accuracy: GPT-J=0.844±0.018, Llama=0.829±0.025, Mistral=0.902±0.006, random forest=0.917±0.014, XGBoost=0.933±0.019 (LLM epochs = 30, LLM learning rate = 0.0003, random forest and XGBoost=default parameters, training set size = 380 data points).

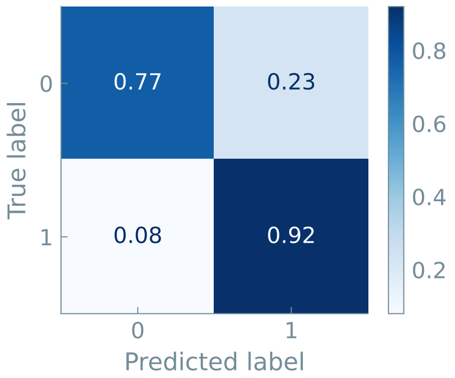


**Figure 109. Normalized confusion matrix, averaged over five independent runs, on the holdout test data for $CO_2$ adsorption capacity prediction with the GPT-J model.** Models were trained using a training set of 380 data points and 30 epochs (accuracy = 84%).

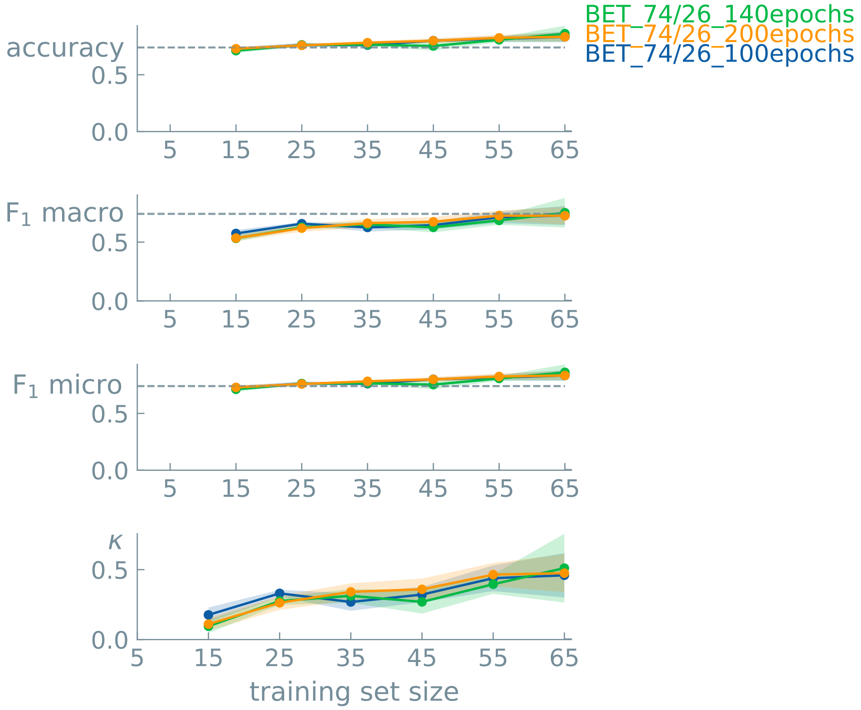


**Figure 110. Learning curves for binary classification GPT-J models (unbalanced classes, 74/24%) for the BET surface area of biomass-derived activated carbons fine-tuned with different number of epochs.** Data points indicate the mean value of five different experiments. Error bands show the standard error of the mean. We used 0.74 as random guess accuracy (dashed line), representing the zero rule baseline, i.e., a model that always predicts the most common class. Accuracy = 0.861±0.070 (epochs = 140, learning rate = 0.0005, training set size = 65 data points).

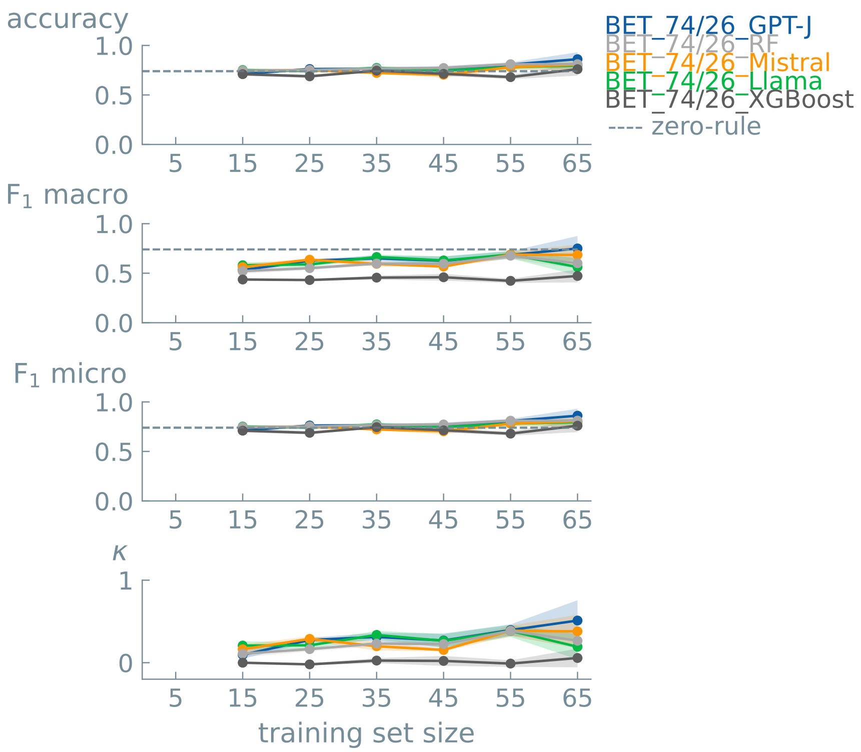


**Figure 111. Learning curves for binary classification models (unbalanced classes, 74/26%) for the BET surface area of biomass-derived activated carbons fine-tuned with different number of epochs.** Data points indicate the mean value of five different experiments. Error bands show the standard error of the mean. We used 0.74 as random guess accuracy (dashed line), representing the zero rule baseline, i.e., a model that always predicts the most common class. Accuracy: GPT-J=0.861±0.070, Llama=0.796±0.034, Mistral=0.806±0.053, random forest=0.811±0.017, XGBoost=0.760±0.065 (LLM epochs = 140, LLM learning rate = 0.0003, random forest and XGBoost=default parameters, training set size = 65 data points).

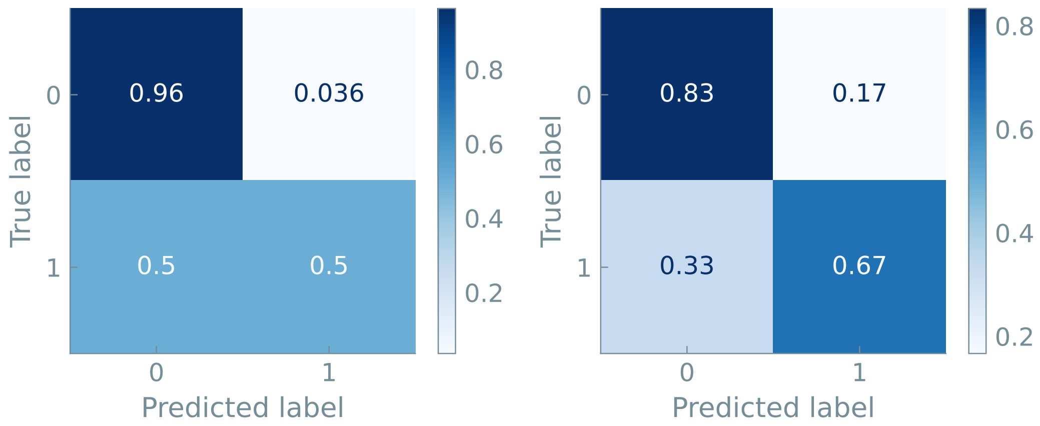


**(a)** **(b)**

**Figure 112. Normalized confusion matrix, averaged over five independent runs, on the holdout test data for BET surface area prediction with the GPT-J model.** Models were trained using an 'unbalanced' dataset with 74% of labels equal to 0, a training set of 65 data points, and 140 epochs (accuracy = 86%) (a), and using a 'balanced' dataset with a training set of 30 data points and 200 epochs (accuracy = 75%) (b).

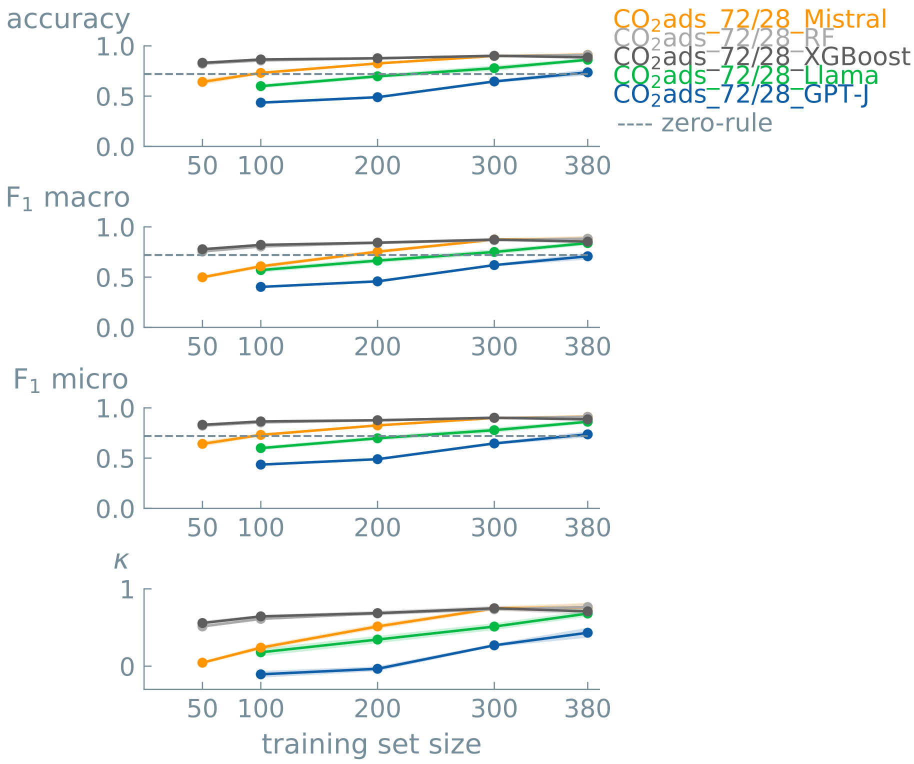


**Figure 113. Learning curves for binary classification models (unbalanced classes, 72/28%) for the $CO_2$ adsorption capacity of biomass-derived activated carbons.** Data points indicate the mean value of five different experiments. Error bands show the standard error of the mean. We used 0.72 as random guess accuracy (dashed line). Accuracy: GPT-J=0.737±0.030, Llama=0.862±0.019, Mistral=0.911±0.021, random forest=0.910±0.019, XGBoost=0.886±0.016 (LLM epochs = 30, LLM learning rate = 0.0003, random forest and XGBoost=default parameters, training set size = 380 data points).

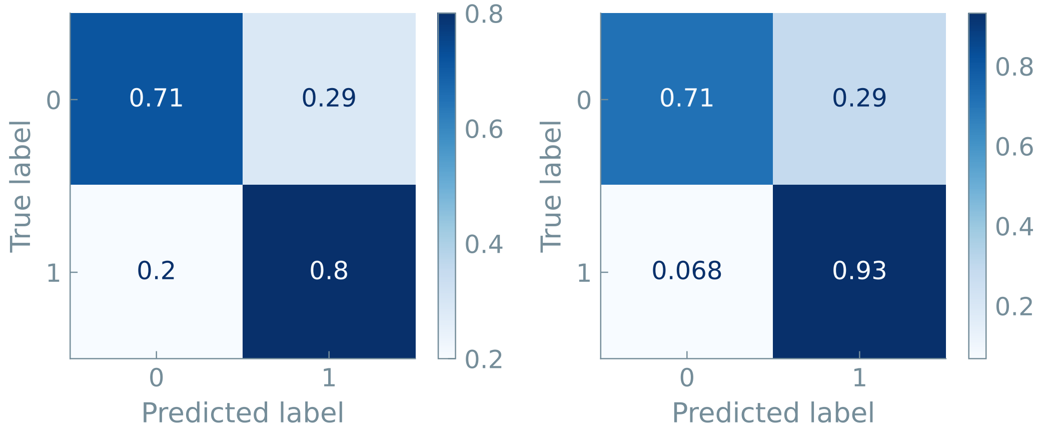


(a) (b)

**Figure 114. Normalized confusion matrix, averaged over five independent runs, on the holdout test data for $CO_2$ adsorption capacity prediction with the GPT-J model.** Models were trained using an 'unbalanced' dataset with 72% of labels equal to 0, a training set of 380 data points and 30 epochs (accuracy = 74%)(a), and using a 'balanced' dataset with a training set of 200 data points and 100 epochs (accuracy = 82%) (b).

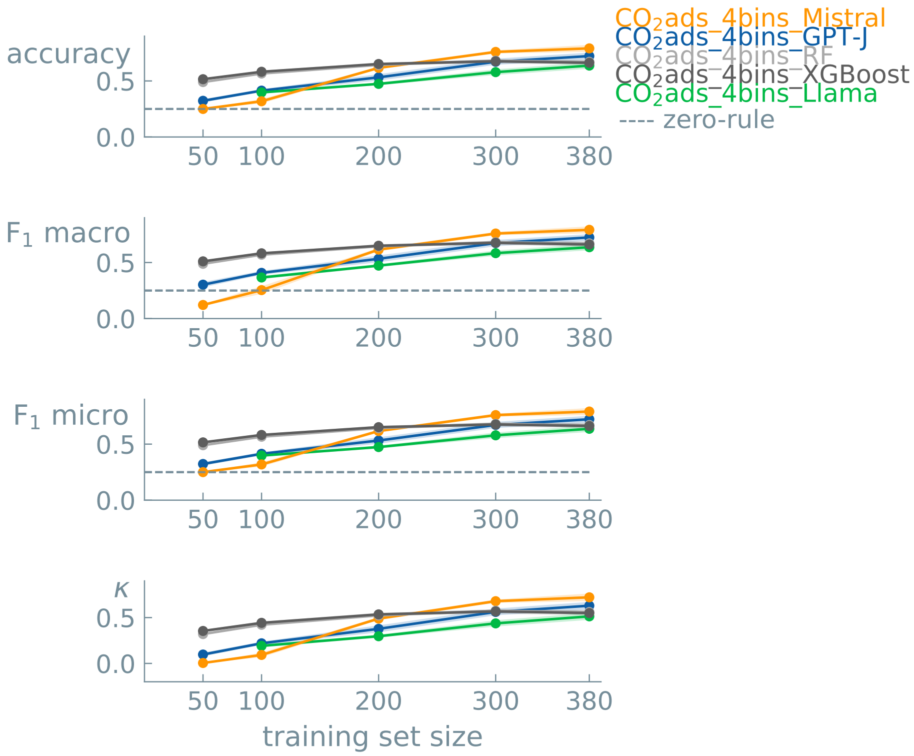


**Figure 115. Learning curves for 4-class classification models for the $CO_2$ adsorption capacity of biomass-derived activated carbons.** Data points indicate the mean value of five different experiments. Error bands show the standard error of the mean. We used 0.25 as random guess accuracy (dashed line). Accuracy: GPT-J=0.722±0.031, Llama=0.637±0.029, Mistral=0.791±0.031, random forest=0.671±0.019, XGBoost=0.662±0.018 (LLM epochs = 30, LLM learning rate = 0.0003, random forest and XGBoost=default parameters, training set size = 380 data points).

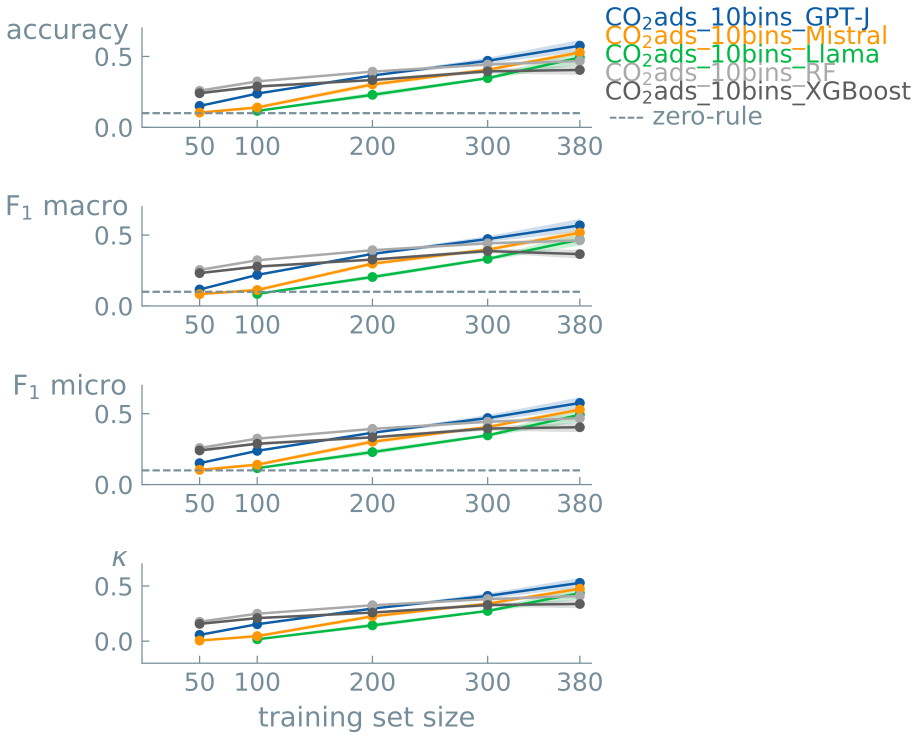


**Figure 116. Learning curves for 10-class classification models for the $CO_2$ adsorption capacity of biomass-derived activated carbons.** Data points indicate the mean value of five different experiments. Error bands show the standard error of the mean. We used 0.10 as random guess accuracy (dashed line). Accuracy: GPT-J=0.576±0.041, Llama=0.493±0.050, Mistral=0.528±0.017, random forest=0.468±0.027, XGBoost=0.405±0.035 (LLM epochs = 30, LLM learning rate = 0.0003, random forest and XGBoost=default parameters, training set size = 380 data points).

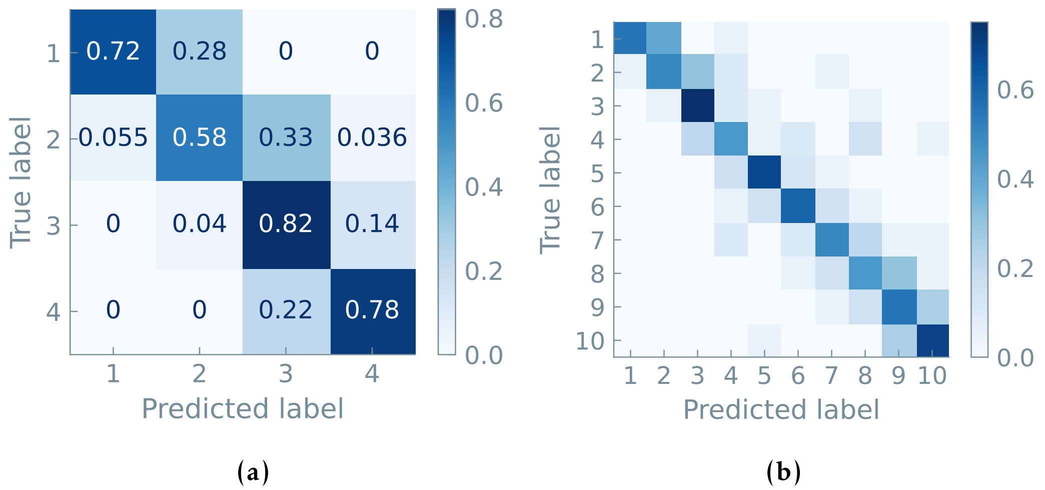


**Figure 117. Normalized confusion matrix, averaged over five independent runs, on the holdout test data for $CO_2$ adsorption capacity prediction with the GPT-J model.** Models were trained using 4-class (a) and 10-class (b) balanced datasets, training sets of 380 data points, and 30 epochs.

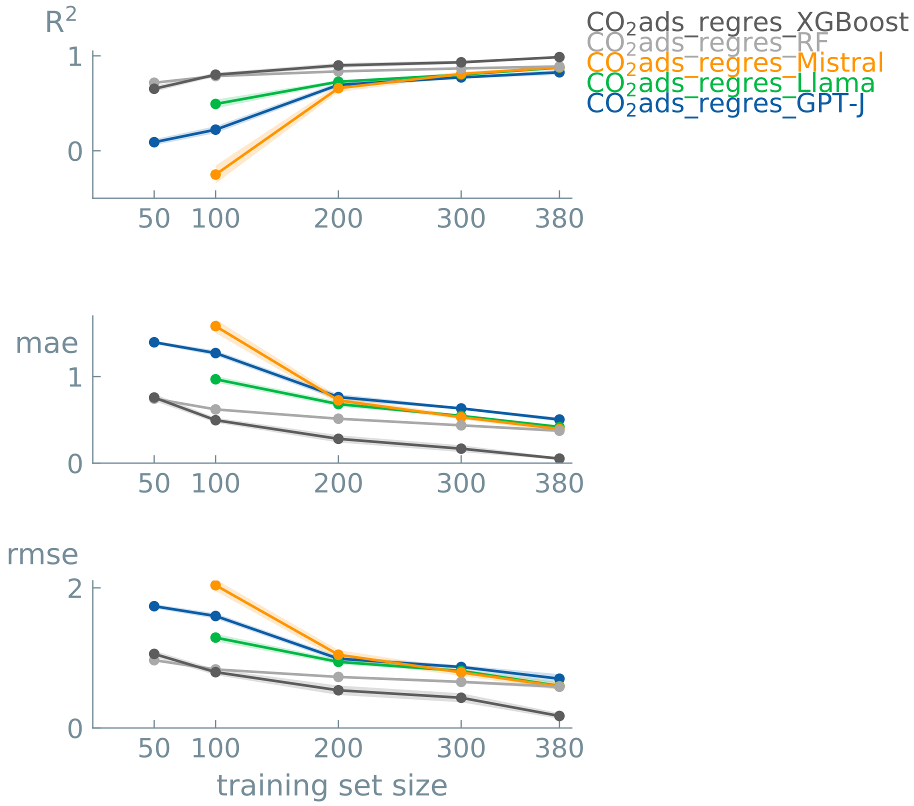


**Figure 118. Learning curves for regression models for the prediction of the $CO_2$ adsorption capacity of biomass-derived activated carbons.** Data points indicate the mean value of five different experiments. Error bands show the standard error of the mean. $R^2$: GPT-J=0.826±0.027, Llama=0.876±0.019, Mistral=0.879±0.016, random forest=0.888±0.012, XGBoost=0.987±0.006; MAE: GPT-J=0.50±0.02, Llama=0.42±0.02, Mistral=0.39±0.03, random forest=0.37±0.02, XGBoost=0.05±0.01; RMSE: GPT-J=0.70±0.06, Llama=0.60±0.05, Mistral=0.59±0.05, random forest=0.59±0.03, XGBoost=0.17±0.04 (LLM epochs = 30, LLM learning rate = 0.0001, random forest and XGBoost=default parameters, training set size = 380 data points).

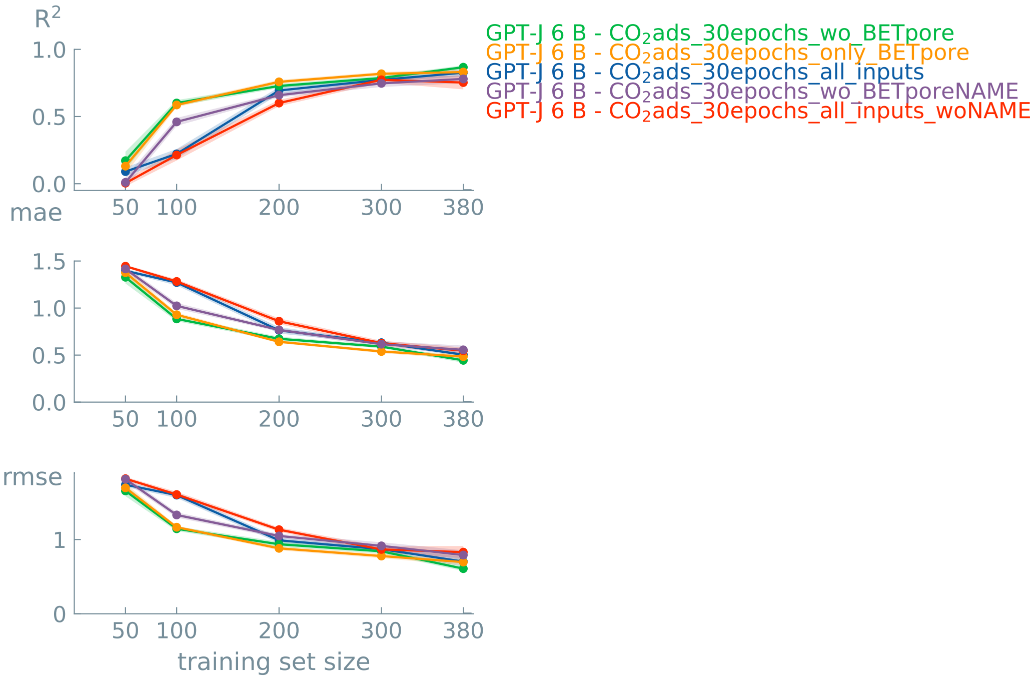


**Figure 119. Learning curves for regression GPT-J models for the prediction of the $CO_2$ adsorption capacity of biomass-derived activated carbons.** The blue line ("all_inputs") represents the results we obtained using as input the biomass precursor, activation conditions, textural properties of the activated carbon, and adsorption conditions. The green line ("wo_BETpore") represents the results we obtained using as input the biomass precursor, activation conditions, and adsorption conditions. The yellow line ("only_BETpore") represents the results we obtained using as input the textural properties of the activated carbon, and adsorption conditions. Data points indicate the mean value of five different experiments using a training set of 380 data points and 30 epochs. Error bands show the standard error of the mean. $R^2$ = 0.827±0.027 for "all_inputs". $R^2$ = 0.831±0.021 for "only_BETpore". $R^2$ = 0.867±0.014 for "wo_BETpore". $R^2$ = 0.773±0.022 for "all_inputs_woNAME". $R^2$ = 0.780±0.032 for "wo_BETporeNAME".

## 5.4 Thermal Desalination of Water

The dataset was provided by: Mehrdad Asgari[21] and Morteza Sagharichiha[22]

### 5.4.1 Scientific Background

Water desalination plays a key role in addressing the global challenge of water scarcity.[86] With freshwater sources dwindling due to factors like population growth, urbanization, and climate change, desalination provides a vital solution to meet the increasing demand for clean water. By removing salt and impurities from seawater or brackish water, desalination technologies offer a sustainable means of securing freshwater supplies, especially in arid regions where traditional water sources are limited. Additionally, desalination enables the utilization of alternative water sources, reducing dependence on finite freshwater resources and mitigating the impact of droughts and water shortages on communities, agriculture, and industries. As the need for accessible and reliable water continues to grow, investing in desalination technologies becomes increasingly critical for ensuring water security and supporting sustainable development worldwide.

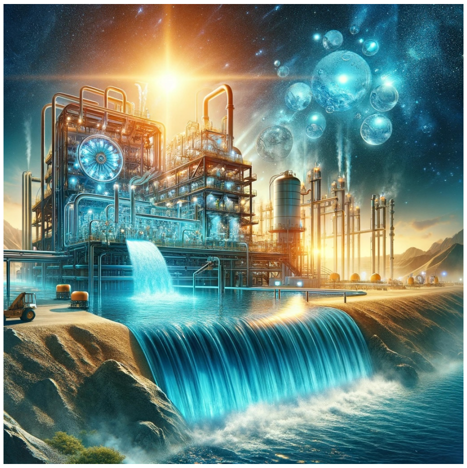

**Figure 120.** AI generated representation of a water desalination process.

Mathematical modeling has been widely used to simulate the behavior of thermal desalination units and optimize their design. However, the complexity of the process and the large number of parameters involved make it difficult to develop accurate models, which can limit the effectiveness of the optimization process. Machine learning can offer a promising approach to training models on large amounts of simulated data to predict the behavior of thermal desalination units. In this work, we develop a model using data from the simulation of thermal desalination units, specifically multiple effect evaporators (MEE) with thermal vapor compression (TVC) and a condenser, for brackish water desalination.[87]

In desalination systems, maximizing distilled water output per unit of energy input is crucial. Multi-effect desalination systems achieve this by distilling one unit of steam to produce another, which is then used in subsequent heat exchangers, known as *"effects"*. The evaluation index for these systems is the Gain Output Ratio (GOR), representing the ratio of the distillate's total latent heat of evaporation to thermal energy input.[88] A higher GOR

[21] Department of Chemical Engineering & Biotechnology, University of Cambridge, Philippa Fawcett Drive, Cambridge CB3 0AS, United Kingdom

[22] Department of Chemical Engineering, College of Engineering, University of Tehran, Tehran, Iran

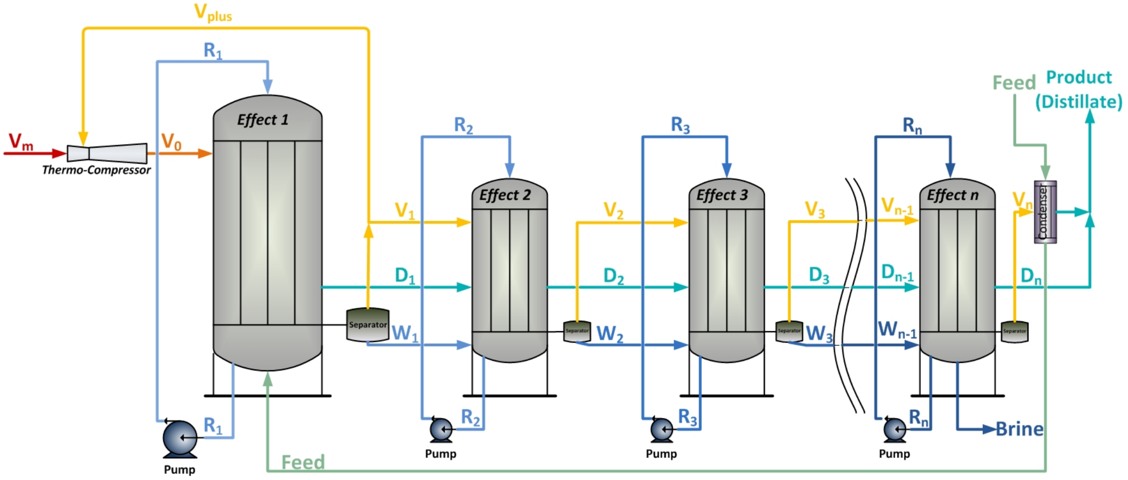


**Figure 121. Illustration depicting the schematic of a multiple effect evaporators plant.**[87] (Reproduced with permission, copyright 2014, Elsevier).

signifies greater thermal energy utilization efficiency. However, there's a trade-off between the number of effects and GOR, impacting variable costs, and the total surface area of heat exchangers, affecting fixed costs. To optimize heat exchangers, understanding how design parameters (e.g., number of effects) affect the required surface area and GOR is crucial. Models are built to predict the specific heat-transfer surface and GOR based on the number of effects and steam temperature. These variables influence the system's final cost: specific heat transfer surface determines plant size and fixed costs, while GOR influences steam consumption and variable costs.

In this study, we utilize data derived from a model we developed[87] to simulate the operation of feed-forward thermal desalination units. Figure 121 This model extensively incorporates mass and energy balances across all thermal desalination effects, accounting for key concepts in the field such as boiling point elevation (BPE) and the impact of non-condensable gases (NCGs) on pressure within the effects. By leveraging this model, critical parameters influencing desalination unit performance can be identified, paving the way for the creation of more efficient and cost-effective thermal desalination units to address the increasing demand for fresh water in water-scarce regions. Furthermore, the precise prediction capabilities offered by this model can streamline the design process, reducing cycle time and enhancing overall design efficiency.

### 5.4.2 Dataset

The dataset contains 30 data points, including information on the number of effects and steam temperature.

We predict the specific heat transfer surface and GOR. The distribution of both variables is shown in Figure 122. We used the number of effects and steam temperature as inputs to predict the specific heat transfer surface and GOR of multiple-effect evaporator systems.

We used a simple prompt template shown in Table 54, for our experiments to predict the specific heat-transfer surface and GOR.

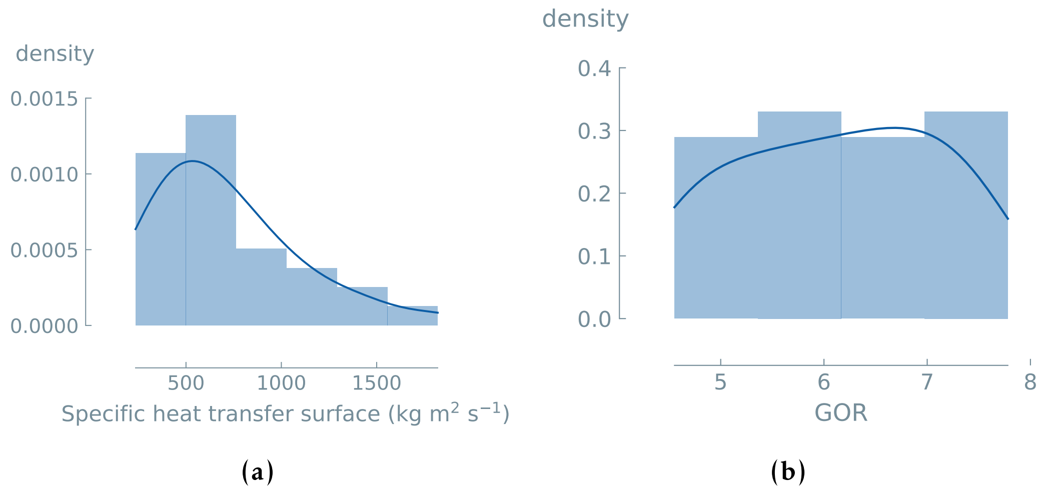


**Figure 122. Distribution of the specific heat transfer surface (a) and Gain Output Ratio (GOR) (b) of multiple-effect evaporator systems in the dataset.** The median specific heat transfer surface is 628 kg $m^2$ $s^{-1}$ and the median GOR is 6.2.

### 5.4.3 LLM results

**Specific heat transfer surface - Base Case** To train the binary classification models, we split the dataset into two classes of equal size based on the specific heat transfer surface separated by the median, i.e., specific heat transfer surface threshold of 628 kg $m^2$ $s^{-1}$. For this dataset, we increased the number of epochs to test whether we could obtain a better prediction than random guess with the GPT-J model.

As shown in Figure 123, we find that GPT-J models trained with 60 and 100 epochs perform much better than random guess (shown by the dashed line) when using training sizes larger than 10 data points, with an accuracy of 83% for a training set of 25 data points and 100 epochs.

Therefore, three base LLMs, i.e., GPT-J, Llama, and Mistral, were fine-tuned using 100 epochs. We also trained two "traditional" ML models, i.e., XGBoost and random Forest (RF), for comparison purposes. Table 55 and Figure 124 show that high accuracies were obtained with the LLM models (80-87%). However, in this case, very high accuracy values were obtained with RF and XGBoost (100%).

**Table 54. Example prompts and completions for predicting the specific heat transfer surface and GOR.**

| prompt | completion | experimental |
|---|---|---|
| `What is the specific heat transfer surface of a <number of effects> effects evaporator for water desalination with a steam temperature of <steam temperature>°C?` | 0 | Low |
| `What is the specific heat transfer surface of a <number of effects> effects evaporator for water desalination with a steam temperature of <steam temperature>°C?` | 1 | High |
| `What is the Gain Output Ratio (GOR) of a <number of effects> effects evaporator for water desalination with a steam temperature of <steam temperature>°C?` | 0 | Low |
| `What is the Gain Output Ratio (GOR) of a <number of effects> effects evaporator for water desalination with a steam temperature of <steam temperature>°C?` | 1 | High |

**Table 55. Overview of the accuracy results of LLMs and "traditional" ML models for binary classification (balanced classes) of the specific heat transfer surface.** Five runs were performed to get the metrics average. LLMs were fine-tuned with 100 epochs and a learning rate of 0.0003. Maximum performances are highlighted in bold.

| Size | Model | Accuracy | F1 Macro | F1 Micro | Kappa |
|---|---|---|---|---|---|
| 25 | GPT-J (LLM) | 0.83 | 0.79 | 0.83 | 0.63 |
| | Llama (LLM) | 0.80 | 0.76 | 0.80 | 0.57 |
| | Mistral (LLM) | 0.87 | 0.82 | 0.87 | 0.69 |
| | **RF** | **1.0** | **1.0** | **1.0** | **1.0** |
| | **XGBoost** | **1.0** | **1.0** | **1.0** | **1.0** |
| | Zero-rule | 0.50 | 0.50 | 0.50 | 0.00 |

As an example, Figure 125 shows an averaged (over five independent runs) normalized confusion matrix for the GPT-J model trained using a training set of 25 data points and 100 epochs. We can see that the model sometimes fails to predict the class with labels equal to

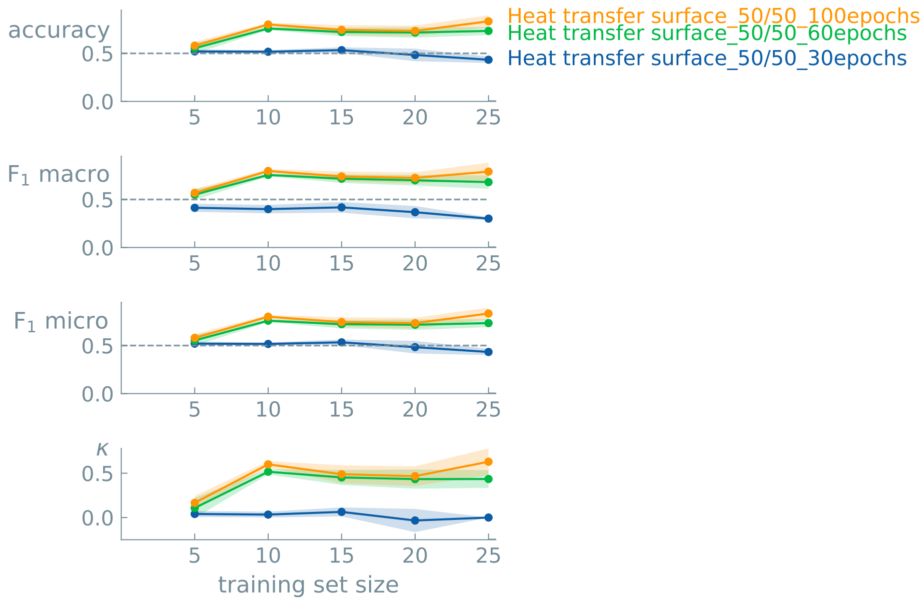


**Figure 123. Learning curves for binary classification GPT-J models (balanced classes) for specific heat transfer surface fine-tuned with different number of epochs.** Data points indicate the mean value of five different experiments. Error bands show the standard error of the mean. We used 0.50 as random guess accuracy (dashed line), representing the zero rule baseline, i.e., a model that always predicts the most common class. Accuracy = 0.833±0.062 (epochs = 100, learning rate = 0.0003, training set size = 25 data points).

zero.

**Gain Output Ratio (GOR) - Base Case** We also split the dataset into two classes of equal size based on the GOR values separated by the median, i.e., 6.2. Figure 126 shows the results for binary classification GPT-J models of GOR. Models trained with 60 and 100 epochs perform much better than random guessing (shown by the dashed line), reaching an accuracy value of 92% for a training set of 25 data points and 100 epochs.

We also fine-tuned three LLMs (GPT-J, Llama, and Mistral) and two "traditional" ML models (XGBoost and RF) with the $CO_2$ adsorption capacity dataset using 30 epochs. Table 56 and Figure 127 show that the trained models perform much better than random guess (shown by the dashed line). The highest accuracy (100%) was obtained with Mistral, but high accuracy values were also obtained with all other models (92-93%) for training sets of 25 data points.

As an example, Figure 128 shows an averaged (over five independent runs) normalized confusion matrix for the GPT-J model trained using a training set of 25 data points and 100

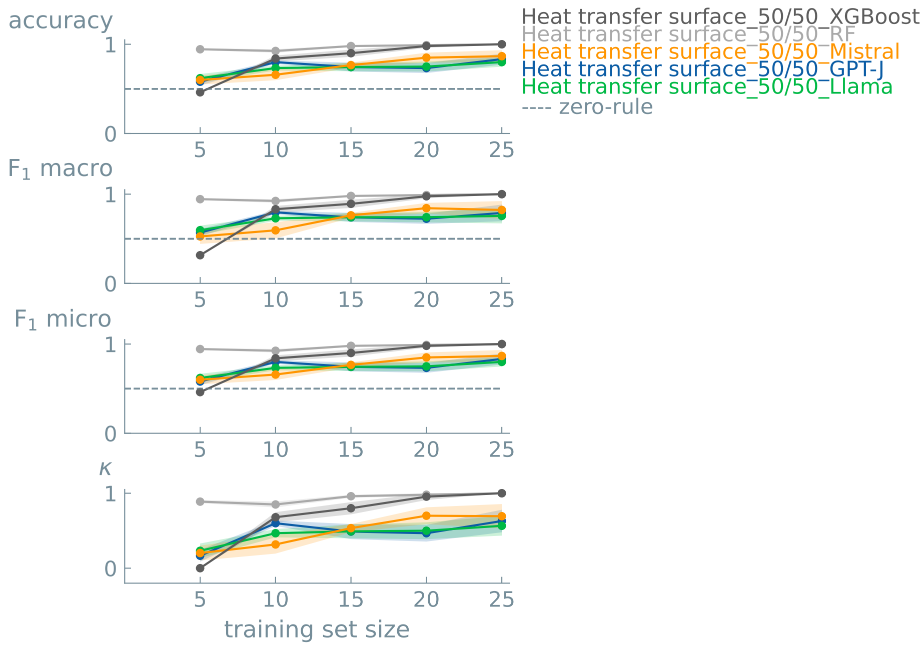


**Figure 124. Learning curves for binary classification models (balanced classes) for specific heat transfer surface.** Data points indicate the mean value of five different experiments. Error bands show the standard error of the mean. We used 0.50 as random guess accuracy (dashed line), representing the zero rule baseline, i.e., a model that always predicts the most common class. Accuracy: GPT-J=0.833±0.061, Llama=0.800±0.052, Mistral=0.867±0.067, random forest=1.0±0.0, XGBoost=1.0±0.0 (LLM epochs = 100, LLM learning rate = 0.0003, random forest and XGBoost=default parameters, training set size = 25 data points).

epochs. We can see that the model predicts the two dataset classes very well.

**Specific heat transfer surface - Real Split** To simulate a more realistic case, we trained binary classification models using unbalanced datasets to predict whether a multi-effect evaporator system has a specific heat transfer surface within the top 20% highest values of the dataset (specific heat transfer surface threshold = $1000\,\mathrm{kg\,m^2\,s^{-1}}$). We fine-tuned three LLMs (GPT-J, Llama, and Mistral) and two "traditional" ML models (XGBoost and RF) with the $CO_2$ adsorption capacity dataset using 30 epochs. Figure 129 shows that the GPT-J, Llama models perform no better than random guess (shown by the dashed line), achieving an accuracy of 80% when using a training set of 25 data points and 100 epochs. A slightly higher accuracy was obtained with Mistral (84%). In this case, the ML models achieved higher accuracy values (94% with RF, and 100% with XGBoost).

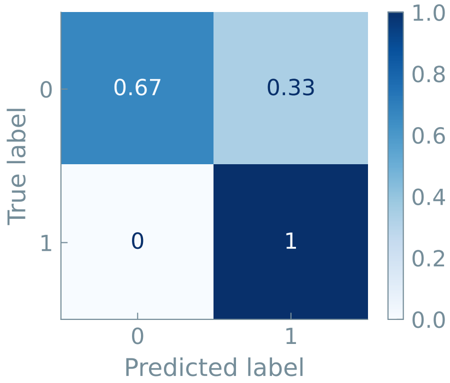


**Figure 125. Normalized confusion matrix, averaged over five independent runs, on the holdout test data for predicting specific heat transfer surface with the GPT-J model.** Models were trained using 25 data points and 100 epochs (accuracy = 83%).

**Table 56. Overview of the accuracy results of LLMs and "traditional" ML models for binary classification (balanced classes) of the Gain Output Ratio (GOR).** Five runs were performed to get the metrics average. LLMs were fine-tuned with 100 epochs and a learning rate of 0.0003. Maximum performances are highlighted in bold.

| Size | Model | Accuracy | F1 Macro | F1 Micro | Kappa |
|---|---|---|---|---|---|
| 25 | GPT-J (LLM) | 0.92 | 0.92 | 0.92 | 0.85 |
| | Llama (LLM) | 0.93 | 0.93 | 0.93 | 0.88 |
| | Mistral (LLM) | **1.0** | **1.0** | **1.0** | **1.0** |
| | RF | 0.92 | 0.91 | 0.92 | 0.83 |
| | XGBoost | 0.93 | 0.91 | 0.93 | 0.84 |
| | Zero-rule | 0.50 | 0.50 | 0.50 | 0.00 |

As an example, Figure 130 shows an averaged (over five independent runs) normalized confusion matrix for the GPT-J model trained with this unbalanced dataset using a training set of 25 data points and 100 epochs. We can see that the model fails to predict high values of the specific heat transfer surface (i.e., label = 1), which is the least represented class in the dataset.

**Gain Output Ratio (GOR) - Real Split** We also trained binary classification models using unbalanced datasets to predict whether a multi-effect evaporator system has a GOR value within the top 33% highest values of the dataset (GOR threshold = 6.8). We fine-tuned three LLMs (GPT-J, Llama, and Mistral) and two "traditional" ML models (XGBoost and RF) with

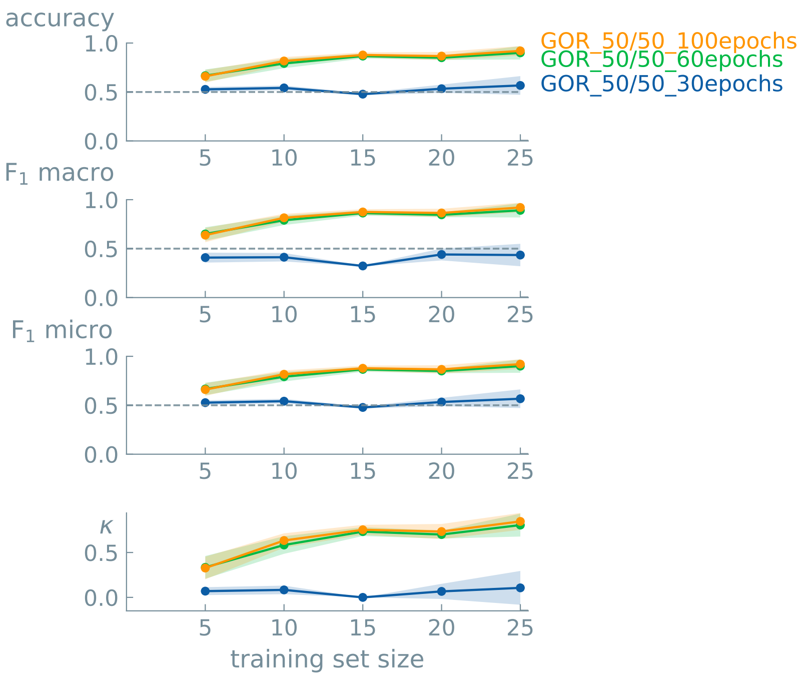


**Figure 126. Learning curves for binary classification GPT-J models (balanced classes) for Gain Output Ratio (GOR) fine-tuned with different number of epochs.** Data points indicate the mean value of five different experiments. Error bands show the standard error of the mean. We used 0.50 as random guess accuracy (dashed line), representing the zero rule baseline, i.e., a model that always predicts the most common class. Accuracy = 0.920±0.049 (epochs = 100, learning rate = 0.0003, training set size = 25 data points).

the $CO_2$ adsorption capacity dataset using 30 epochs. Figure 131 shows good performance for all models, with higher accuracy values than random guess (shown by the dashed line). Similar performance was obtained for all models (90-100%) when using a training set of 25 data points and 100 epochs.

As an example, Figure 132 shows an averaged (over five independent runs) normalized confusion matrix for the GPT-J model trained with this unbalanced dataset using a training set of 25 data points and 100 epochs. We can see that the model can still predict the two classes in the dataset quite well.

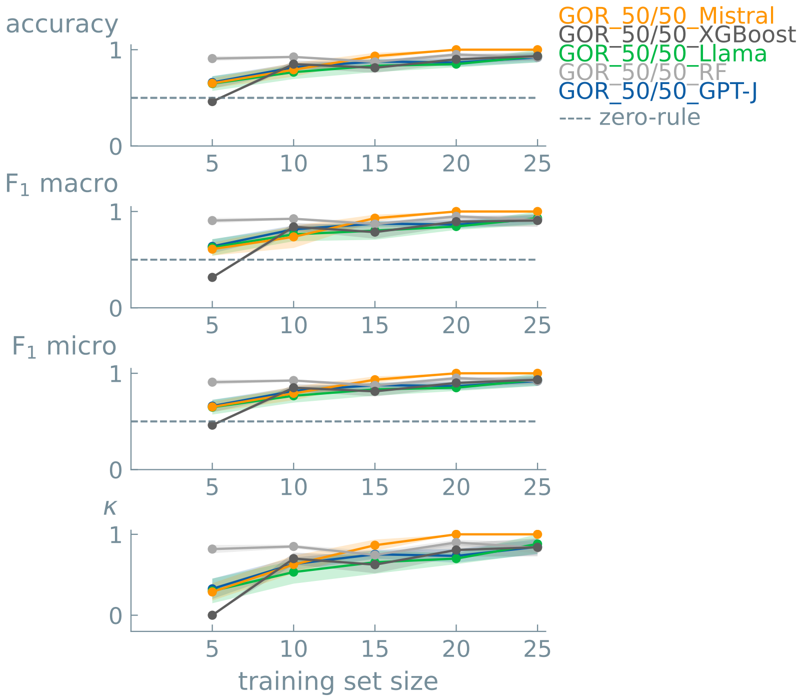


**Figure 127. Learning curves for binary classification models (balanced classes) for Gain Output Ratio (GOR).** Data points indicate the mean value of five different experiments. Error bands show the standard error of the mean. We used 0.50 as random guess accuracy (dashed line), representing the zero rule baseline, i.e., a model that always predicts the most common class. Accuracy: GPT-J=0.920±0.049, Llama=0.933±0.067, Mistral=1.0±0.0, random forest=0.920±0.033, XGBoost=0.933±0.044 (LLM epochs = 100, LLM learning rate = 0.0003, random forest and XGBoost=default parameters, training set size = 25 data points).

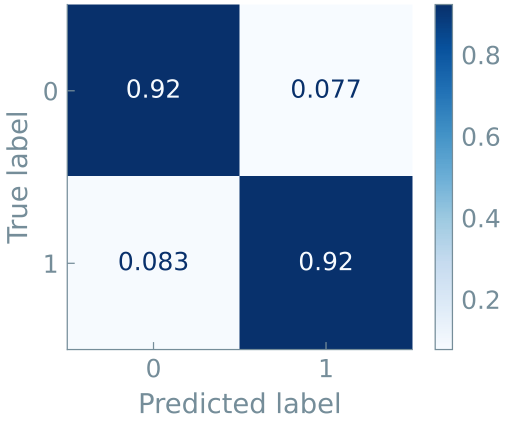


**Figure 128. Normalized confusion matrix, averaged over five independent runs, on the holdout test data for the prediction of Gain Output Ratio (GOR) with the GPT-J model.** Models were trained using 25 data points and 100 epochs (accuracy = 92%).

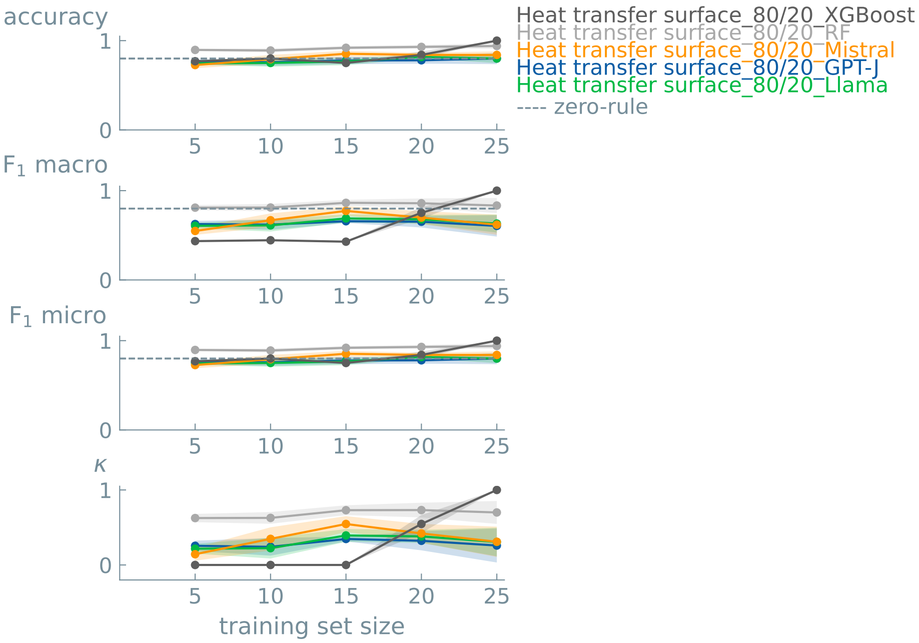


**Figure 129. Learning curves for binary classification models (unbalanced classes, 80/20%) for specific heat transfer surface.** Data points indicate the mean value of five different experiments. Error bands show the standard error of the mean. We used 0.80 as a random guess accuracy (dashed line), representing the zero rule baseline, i.e., a model that always predicts the most common class. Accuracy: GPT-J=0.800±0.063, Llama=0.800±0.052, Mistral=0.840±0.040, random forest=0.940±0.031, XGBoost=1.0±0.0 (LLM epochs = 100, LLM learning rate = 0.0003, random forest and XGBoost=default parameters, training set size = 25 data points).

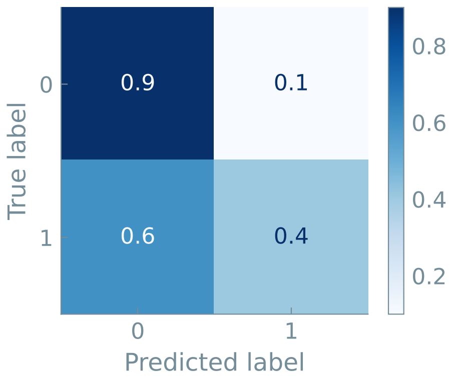


**Figure 130. Normalized confusion matrix, averaged over five independent runs, on the holdout test data for the prediction of specific heat transfer surface with the GPT-J model.** Models were trained using an unbalanced dataset with 20% of labels equal to '1', a training set of 25 data points, and 100 epochs (accuracy = 80%).

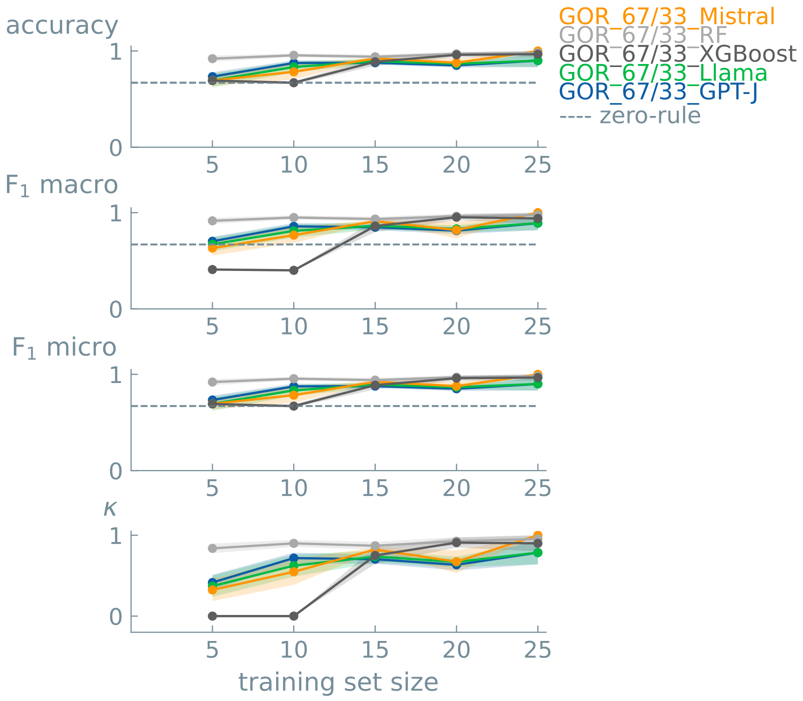


**Figure 131. Learning curves for binary classification models (unbalanced classes, 67/33%) for Gain Output Ratio (GOR).** Data points indicate the mean value of five different experiments. Error bands show the standard error of the mean. We used 0.67 as random guess accuracy (dashed line), representing the zero rule baseline, i.e., a model that always predicts the most common class. Accuracy: GPT-J=0.900±0.068, Llama=0.900±0.068, Mistral=1.0±0.0, random forest=0.980±0.020, XGBoost=0.967±0.033 (LLM epochs = 100, LLM learning rate = 0.0003, random forest and XGBoost=default parameters, training set size = 25 data points).

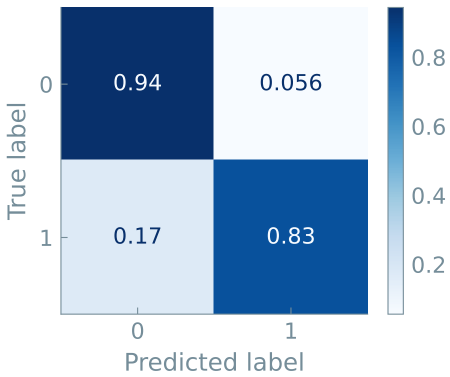


**Figure 132. Normalized confusion matrix, averaged over five independent runs, on the holdout test data for the prediction of Gain Output Ratio (GOR) with the GPT-J model.** Models were trained using an unbalanced dataset with 33% of labels equal to 1, a training set of 25 data points, and 100 epochs (accuracy = 90%).

## 5.5 Detection Response of Gas Sensors

The dataset was provided by: Mehrdad Asgari[23] and Fahimeh Hooriabad Saboor[24]

### 5.5.1 Scientific Background

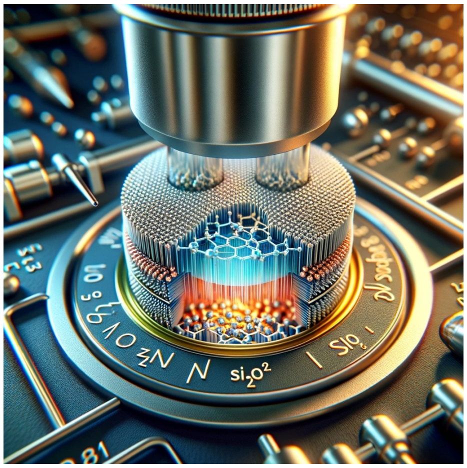

**Figure 133.** AI generated representation of the rendering of a gas sensor.

Sensors play a crucial role across various domains, such as environmental monitoring, industrial safety, and medical diagnostics. However, developing efficient gas sensors poses significant challenges due to the profound impact of material structure and composition on sensor performance. Investigating the intricate relationships between material structure, composition, and sensing response is essential for designing and improving gas sensors with enhanced sensitivity and selectivity. In this study, we focus on two main types of gas sensors: core-shell and composite. Building upon our previous research on core-shell nanostructures' effectiveness in sensing properties,[89] we synthesized a comprehensive set of core-shell and composite sensors with varying $ZnO/SiO_2$ compositions. Given the complexity of sensor nanostructures, traditional modeling approaches may fall short, necessitating the exploration of new models capable of understanding this relationship. We evaluated the selective detection of ethanol ($C_2H_5OH$) compared to interfering gases like carbon monoxide (CO), methane ($CH_4$), propane ($C_3H_8$), trichloroethylene ($C_2HCl_3$), and toluene ($C_6H_5CH_3$) in dry air.

Silica/ZnO core/shell gas sensors and composite nanostructured gas sensors were prepared using two-step (Figure 134a) and one-step microemulsion methods,(Figure 134b) respectively. The zinc oxide concentration in the samples ranged from 15% to 90% by weight. The morphology of core-shell and composite sensors is illustrated in the Figure 135 and Figure 136, respectively.

The powder samples were mixed in deionized water and ball-milled to create a uniform paste. This paste was then screen-printed onto an alumina substrate, placed between two gold electrodes spaced 1 mm apart, which were already present on the alumina surface (Figure 137). All sensors underwent drying at 80 °C and annealing at 480 °C for 2 hours. To evaluate sensor performance, an experimental setup utilizing a continuous flow system was

[23]Department of Chemical Engineering & Biotechnology, University of Cambridge, Philippa Fawcett Drive, Cambridge CB3 0AS, United Kingdom

[24]Chemical Engineering Department, University of Mohaghegh Ardabili, P.O. Box 179, Ardabil, Iran

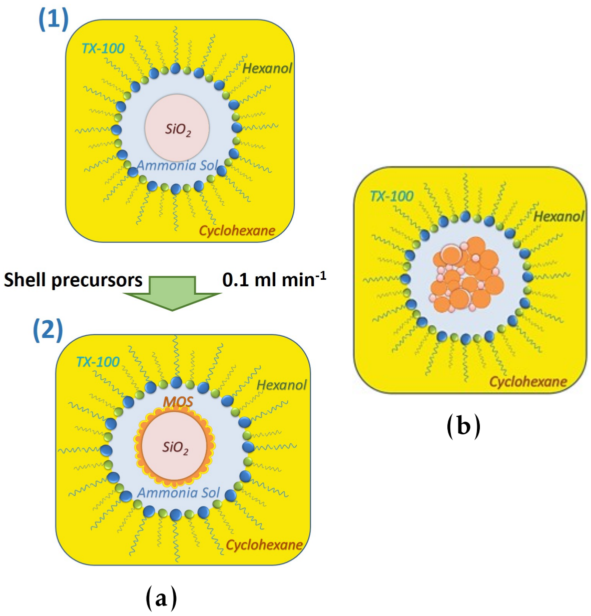


**Figure 134. Sensor synthesis.** (a) Illustration depicting the two-step microemulsion synthesis process used to create core-shell sensors. (b) Diagram illustrating the single-step microemulsion synthesis process for producing composite sensors.

employed (Figure 138), exposing the sensors to various gases at temperatures from 300 °C to 500 °C.

In this study, we develop a model to predict the sensing response as a function of sensor type (core-shell and composite), zinc oxide content, and operating temperature. This model can help better understand the underlying mechanisms responsible for the sensing behavior and identify relationships between material properties and performance. This can guide the development of intelligently designed sensors that operate within a specific temperature range of interest, leading to discoveries and innovations in the field of sensor technology.

### 5.5.2 Dataset

The dataset contains 56 data points, including information on the type of sensor, i.e., core-shell and composite, its zinc oxide content, and operating temperature.

We predict the sensor detection response. The dataset's sensing response values distribution is shown in Figure 139. To predict the sensor's sensing response, we used the sensor type (core-shell and composite), its zinc oxide content, and the operating temperature as inputs.

We used a simple prompt template shown in Table 57 for experiments to predict sensor response.

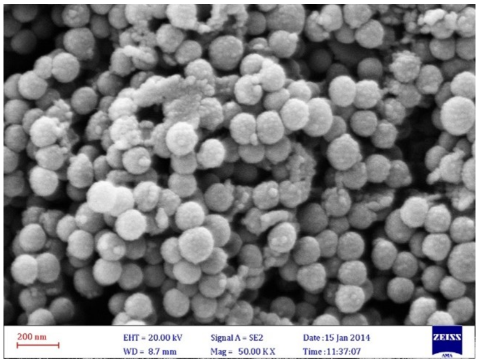


**Figure 135. SEM image displaying the morphology of a core-shell sample with 40 wt% Zn.**

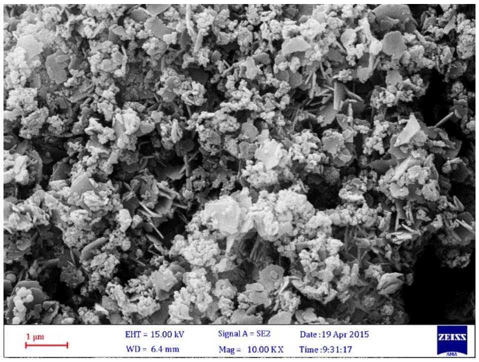


**Figure 136. SEM image displaying the morphology of a composite sample with 40 wt% Zn.**

### 5.5.3 LLM results

**Base case** To train the binary classification models, we split the dataset into two classes of equal size based on the sensor detection response separated by the median, i.e., sensing response threshold of 12. For this dataset, we have used 100 fine-tuning epochs. Otherwise, we would have obtained a too-high number of NaN or invalid predictions with the GPT-J model. We fine-tuned three LLMs, i.e., GPT-J, Llama, and Mistral, and we also trained two "traditional" ML models, i.e., XGBoost and random forest (RF), for comparison purposes. Table 58 and Figure 140 show that models trained with 100 epochs perform much better than random guess (shown by the dashed line) when using training sizes equal to or greater than 30 data points. Similar accuracy values (89-90%) are obtained with GPT-J, RF, and XGBoost, and slightly lower values with Llama and Mistral (86-88%) for a training set of 45 data points.

As an example,Figure 141 shows an averaged (over five independent runs) normalized

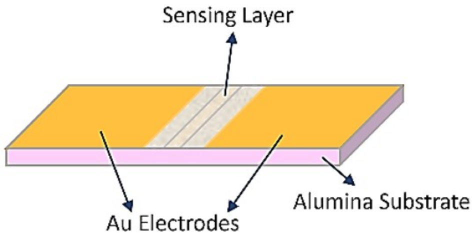


**Figure 137. A schematic depiction of a sensor substrate coated with a paste of the sensing material.**

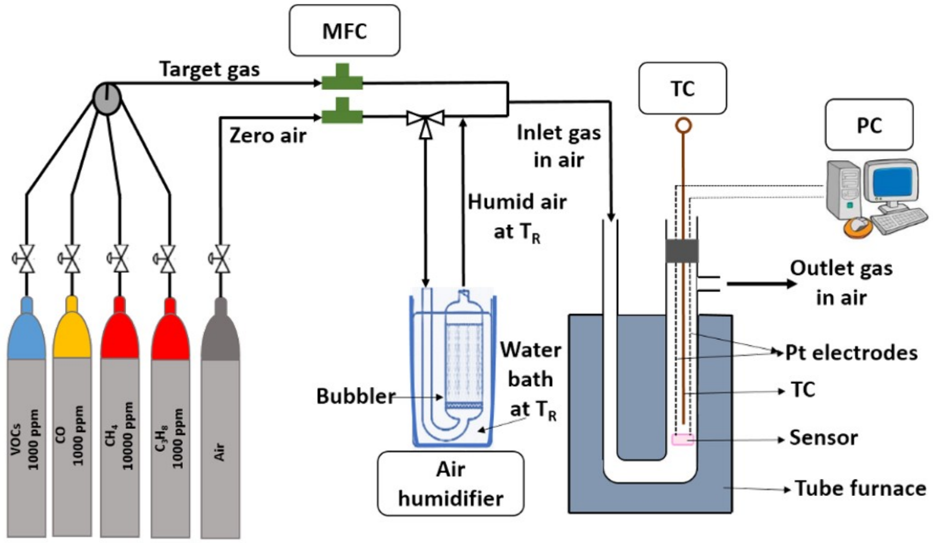


**Figure 138. A schematic illustration of the flow-through gas sensor setup utilized in this study.**

confusion matrix for the GPT-J model trained using a training set of 45 data points and 100 epochs. We can see the good predictive performance of the model for the two classes in the dataset.

**Real split** To simulate a more realistic case, we trained binary classification models using unbalanced datasets to predict whether a sensor gives a response within the top 30% highest response values in the dataset (detection response threshold = 20). Three LLMs (GPT-J, Llama, and Mistral) and two "traditional" ML models (XGBoost and RF) were also fine-tuned with an unbalanced dataset using 100 epochs. Figure 142 shows acceptable performance for these models, which still perform better than random guess (shown by the dashed line). Similar accuracy values are obtained for all the models when trained using an unbalanced

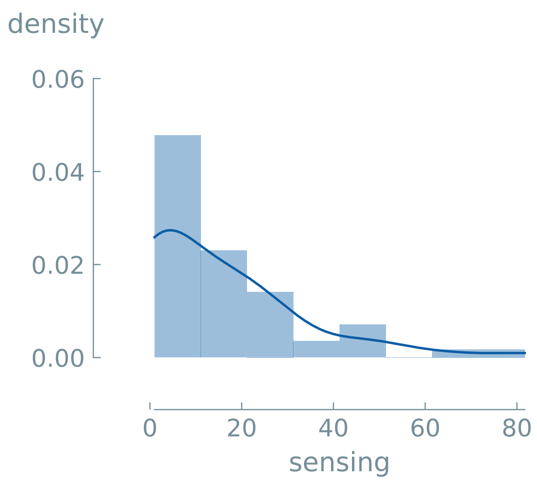


**Figure 139. Distribution of the dataset's detection response of gas sensors.** The median sensor detection response is 12.

dataset using a training set of 45 data points.

As an example, Figure 143 shows an averaged (over five independent runs) normalized confusion matrix for the GPT-J model trained with this unbalanced dataset using a training set of 45 data points and 100 epochs. We can see that the model predicts the two classes of the dataset quite well.

**Table 57. Example prompts and completions for predicting the sensor detection response.**

| prompt | completion | experimental |
|---|---|---|
| What is the sensor detection response of a <sensor type> gas sensor that contains <ZnO content>% of ZnO at an operating temperature of <temperature>°C? | 0 | Low |
| What is the sensor detection response of a <sensor type> gas sensor that contains <ZnO content>% of ZnO at an operating temperature of <temperature>°C? | 1 | High |

**Table 58. Overview of the accuracy results of LLMs and "traditional" ML models for binary classification (balanced classes) of the sensor response.** Five runs were performed to get the metrics average. LLMs were fine-tuned with 100 epochs and a learning rate of 0.0003. Maximum performances are highlighted in bold.

| Size | Model | Accuracy | F1 Macro | F1 Micro | Kappa |
|---|---|---|---|---|---|
| 45 | GPT-J (LLM) | 0.89 | 0.89 | 0.89 | 0.78 |
| | Llama (LLM) | 0.86 | 0.86 | 0.86 | 0.72 |
| | Mistral (LLM) | 0.88 | 0.87 | 0.87 | 0.76 |
| | RF | 0.89 | 0.89 | 0.89 | 0.78 |
| | **XGBoost** | **0.90** | **0.89** | **0.90** | **0.80** |
| | Zero-rule | 0.50 | 0.50 | 0.50 | 0.00 |

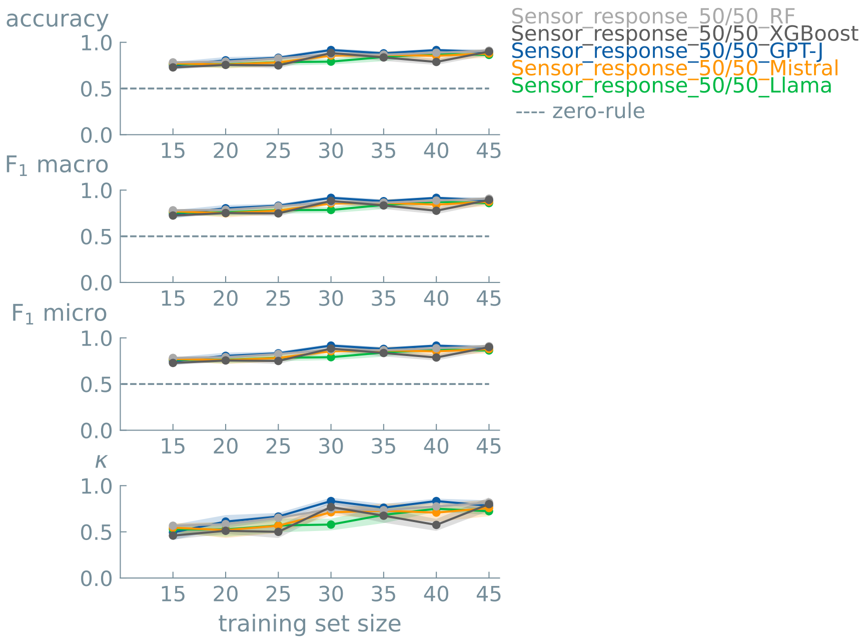


**Figure 140. Learning curves for binary classification models (balanced classes) for sensor response prediction.** Data points indicate the mean value of five different experiments. Error bands show the standard error of the mean. We used 0.50 as random guess accuracy (dashed line), representing the zero rule baseline, i.e., a model that always predicts the most common class. Accuracy: GPT-J=0.894±0.028, Llama=0.864±0.020, Mistral=0.879±0.045, random forest=0.891±0.018, XGBoost=0.900±0.037 (LLM epochs = 100, LLM learning rate = 0.0003, random forest and XGBoost=default parameters, training set size = 45 data points).

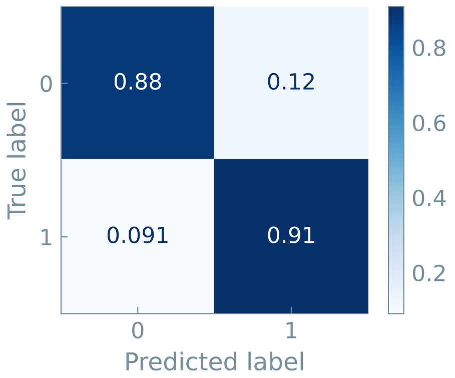


**Figure 141. Normalized confusion matrix, averaged over five independent runs, on the holdout test data for prediction of sensor detection response with the GPT-J model.** Models were trained using a training set of 45 data points and 100 epochs (accuracy = 89%).

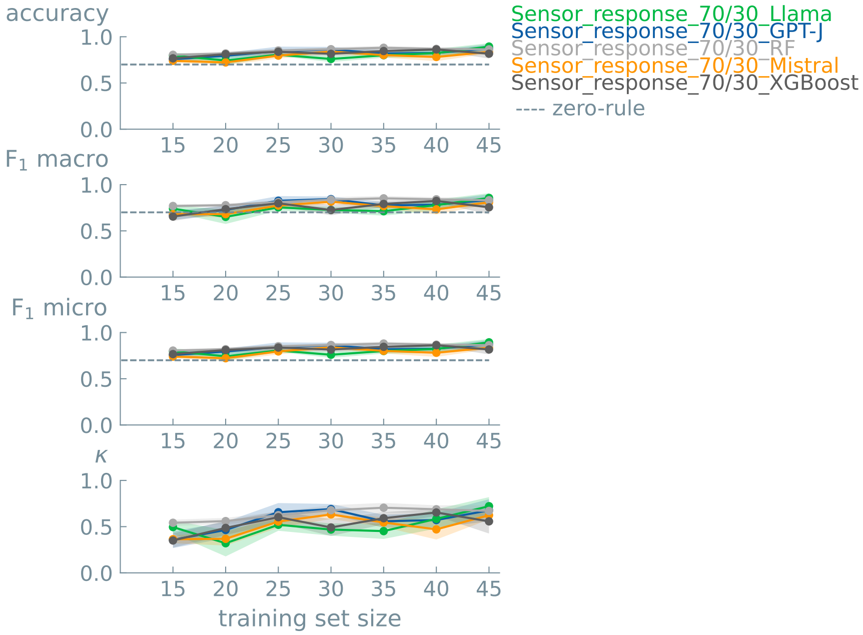


**Figure 142. Learning curves for binary classification models (unbalanced classes, 70/30%) for sensor detection response.** Data points indicate the mean value of five different experiments. Error bands show the standard error of the mean. We used 0.70 as a random guess accuracy (dashed line), representing the zero rule baseline, i.e., a model that always predicts the most common class. Accuracy: GPT-J=0.873±0.046, Llama=0.894±0.036, Mistral=0.836±0.018, random forest=0.864±0.024, XGBoost=0.817±0.052 (LLM epochs = 100, LLM learning rate = 0.0003, random forest and XGBoost=default parameters, training set size = 45 data points).

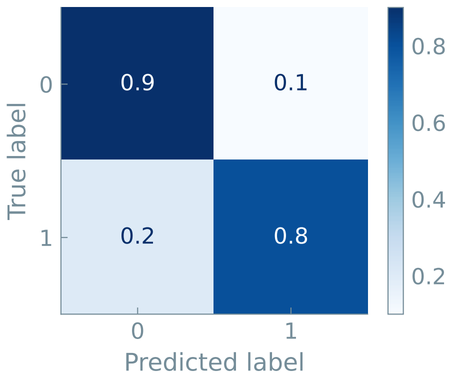


**Figure 143. Normalized confusion matrix, averaged over five independent runs, on the holdout test data for prediction of sensor detection response with the GPT-J model.** Models trained using an unbalanced dataset with 30% labels equal to 1, a training set of 45 data points, and 100 epochs (accuracy = 87%).

## 5.6 Stability of Gas Sensors

The dataset was provided by: Mehrdad Asgari[25] and Sahar Vahdatifar[26]

### 5.6.1 Scientific Background

Doping $SnO_2$-based gas sensors with various additives can enhance not only their sensitivity and selectivity but also, crucially, their long-term stability. For instance, research has indicated that certain additives have the potential to stabilize the performance of a $Pt/SnO_2$ sensor.[90] Moreover, elevating the annealing temperature during the synthesis process may mitigate the decline in sensitivity over time, thereby enhancing the sensors' stability. It is noteworthy that multiple factors, including compositions and annealing temperature, interact, complicating the development of a model to elucidate the sensor's behavior over time.

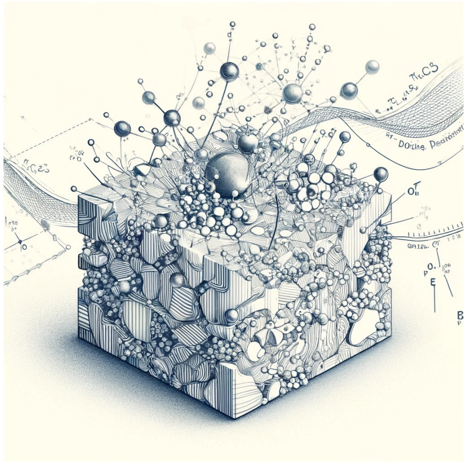

**Figure 144.** AI generated representation of the doping of $SnO_2$-based gas sensors.

Indeed, beyond the factors influencing stability, the very definition of stability is crucial for assessing whether a material is stable. This becomes increasingly significant, particularly as the decay in sensor sensitivity over time does not adhere to a linear or easily definable pattern. Various sensors exhibit diverse deactivation mechanisms and behaviors, complicating establishing a clear definition and prediction for sensor stability.

Herein, we aim to develop a model for predicting the stability of synthesized sensors with varying compositions, utilizing a robust dataset derived from systematic experiments conducted on our gas sensors of interest. In this case study, we focused on $Pt/SnO_2$ nanoparticles doped with various materials such as $MoO_3$, $CeO_2$, $Sm_2O_3$, and $SiO_2$ to enhance the long-term stability of gas sensors for carbon monoxide (CO) detection.[91] Pure $SnO_2$ and $SnO_2$ doped with 5.0 and 10.0 wt.% nano additives were synthesized by a sol-gel method, which is used to precisely control the composition of gas sensors, and then these materials were impregnated with 1.0 wt.% platinum. The effect of dopants and annealing temperature during synthesis on the detection ability was analyzed.[91]

To assess the stability of the materials, we conducted accelerated stability tests on the synthesized samples. These tests involved exposing the materials to air with 30% relative

[25] Department of Chemical Engineering & Biotechnology, University of Cambridge, Philippa Fawcett Drive, Cambridge CB3 0AS, United Kingdom
[26] Department of Chemical Engineering, College of Engineering, University of Tehran, Tehran, Iran

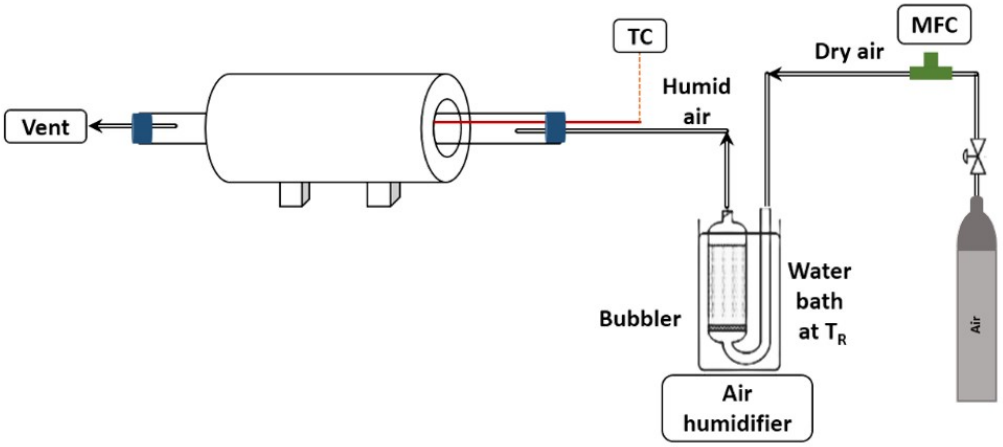


**Figure 145. Schematic figure of the custom-build setup for aging synthesized sensors.**

humidity at a custom-built setup designed for aging the synthesized sensors under controlled conditions. A schematic figure of this setup used for this study can be seen in (Figure 145). The temperature of the aging was set at 600 °C with the duration of aging varying up to 15 days. Subsequently, we evaluated the performance of the sensors through gas detection tests, specifically targeting 100 ppm of CO at 250 °C. These tests were conducted immediately after synthesis and repeated on days 5, 9, 12, and 15 to monitor any changes in performance over time. The sensors were fabricated following the method outlined in the previous section (Figure 137). Additionally, the sensitivity of the gas sensors to CO was measured using the setup described earlier (Figure 138).[91] The difference in detection response between days 5 and 15 served as a metric to gauge the stability of the gas sensors. Sensors exhibiting a response loss of less than 20% were deemed to be highly stable.

In this study, we develop a model to predict whether a $SnO_2$-based gas sensor is stable or not as a function of the type of dopant material, its dosage, and the calcination temperature during synthesis. This model could contribute to developing more stable and efficient gas sensors.

### 5.6.2 Dataset

The dataset contains 19 data points, including information on the type of dopant material, its dosage, and the calcination temperature during sensor synthesis.

We predict the stability of the sensor in the detection response. The dataset's sensing response loss distribution is shown in Figure 146. To predict sensor stability, we used the type of dopant material, its dosage, and the calcination temperature during synthesis as inputs.

We used a simple prompt template shown in Table 59 for experiments to predict sensor

stability.

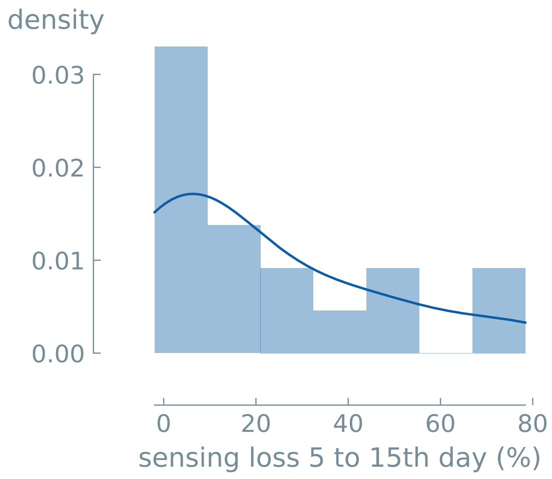


**Figure 146. Distribution of the detection response loss of $SnO_2$-based gas sensors in the dataset.** The median detection response loss between days 5 and 15 is 12%.

### 5.6.3 LLM results

**Base case** To train the binary classification models, we split the dataset into two classes of equal size based on sensor stability separated by the median, i.e., response loss between days 5 and 15 less than 12%. For this dataset, we used 120 fine-tuning epochs, as otherwise, we obtained a high number of NaN or invalid predictions with the GPT-J model. We fine-tuned three LLMs, i.e., GPT-J, Llama, and Mistral, and we also trained two "traditional" ML models, i.e., XGBoost and Random Forest (RF), for comparison purposes. Table 60 and Figure 147 show that the models trained with 120 epochs perform slightly better than random guess (shown by the dashed line). The higher accuracy was achieved with the GPT-J model (71%) for a training set of 15 data points, close to that obtained with RF (68%). The performance was slightly lower with Llama and Mistral (63%), while very low accuracy was reached with XGBoost (0.30%) for this dataset.

As an example, Figure 148 shows an averaged (over five independent runs) normalized confusion matrix for the GPT-J model trained using a training set of 15 data points and 120 epochs. The model sometimes fails to predict samples labeled '1' or '0'.

**Real split** To simulate a more realistic, less restrictive case, we trained binary classification models using unbalanced datasets to predict whether a sensor is stable. We define a sensor as stable if it has a response loss of less than 20%. Data points with this response loss represent 63% of our overall dataset. Three LLMs (GPT-J, Llama, and Mistral) and two "traditional"

**Table 59. Prompt template and completions.** This prompt was used to predict the sensor stability.

| prompt | completion | experimental |
|---|---|---|
| `What is the sensor stability of a gas sensor synthesized from Pt/SnO2 nanoparticles doped with <dopant type> at a dose of <dopant dosage> at an annealing temperature of <calcination temperature>°C?` | 0 | Low |
| `What is the sensor stability of a gas sensor synthesized from Pt/SnO2 nanoparticles doped with <dopant type> at a dose of <dopant dosage> at an annealing temperature of <calcination temperature>°C?` | 1 | High |

**Table 60. Overview of the accuracy results of LLMs and "traditional" ML models for binary classification (balanced classes) of the sensor stability.** Five runs were performed to get the metrics average. LLMs were fine-tuned with 120 epochs and a learning rate of 0.0003. Maximum performances are highlighted in bold.

| Size | Model | Accuracy | F1 Macro | F1 Micro | Kappa |
|---|---|---|---|---|---|
| 15 | **GPT-J (LLM)** | **0.71** | **0.70** | **0.71** | **0.42** |
| | Llama (LLM) | 0.63 | 0.56 | 0.63 | 0.25 |
| | Mistral (LLM) | 0.63 | 0.58 | 0.63 | 0.25 |
| | RF | 0.68 | 0.62 | 0.68 | 0.35 |
| | XGBoost | 0.30 | 0.24 | 0.30 | -0.40 |
| | Zero-rule | 0.50 | 0.50 | 0.50 | 0.00 |

ML models (XGBoost and RF) were also fine-tuned with an unbalanced dataset using 120 epochs. Figure 149 shows that the LLM models perform slightly better than random guess (shown by the dashed line) (79-83%) when using a training set of 15 data points. In this case, the LLM models show a higher performance than RF (75%) and XGBoost (63%).

As an example, Figure 150 shows an averaged (over five independent runs) normalized confusion matrix for the GPT-J model trained with this unbalanced dataset using a training set of 15 data points and 120 epochs. We can see that the model sometimes fails to predict stable sensors (i.e., label = 1).

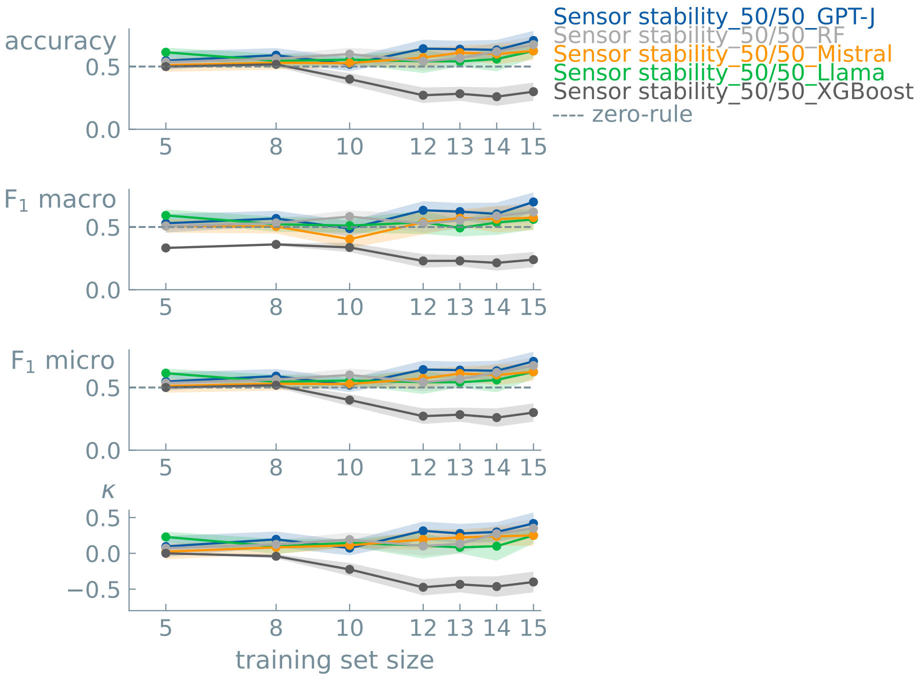


**Figure 147. Learning curves for binary classification models (balanced classes) for sensor stability.** Data points indicate the mean value of five different experiments. Error bands show the standard error of the mean. We used 0.50 as random guess accuracy (dashed line), representing the zero rule baseline, i.e., a model that always predicts the most common class. Accuracy: GPT-J=0.708±0.077, Llama=0.625±0.056, Mistral=0.625±0.072, random forest=0.675±0.065, XGBoost=0.300±0.073 (LLM epochs = 120, LLM learning rate = 0.001, random forest and XGBoost=default parameters, training set size = 15 data points).

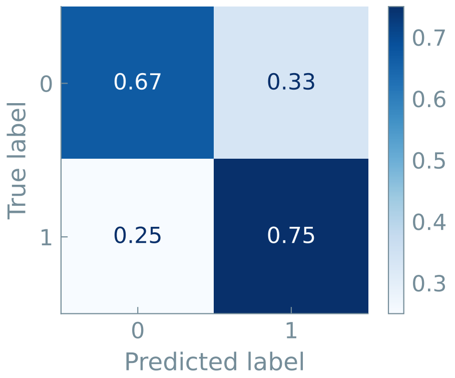


**Figure 148. Normalized confusion matrix, averaged over five independent runs, on the holdout test data for the prediction of sensor stability with the GPT-J model.** Models were trained using a training set of 15 data points and 120 epochs (accuracy = 71%).

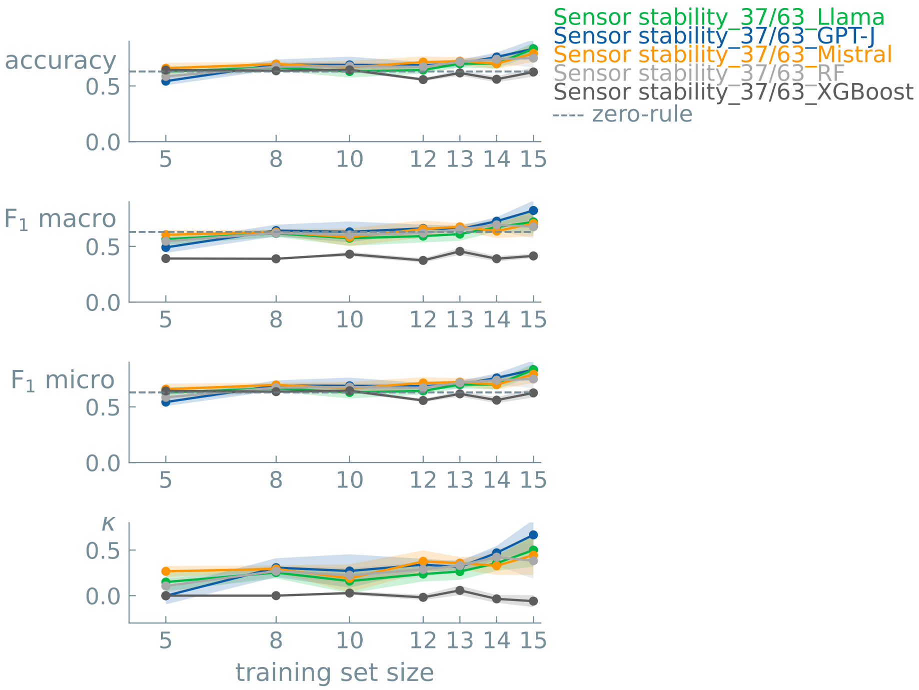


**Figure 149. Learning curves for binary classification models (unbalanced classes, 37/63%) for sensor stability.** Data points indicate the mean value of five different experiments. Error bands show the standard error of the mean. We used 0.63 as random guess accuracy (dashed line), which represents the zero rule baseline, i.e., a model that always predicts the most common class. Accuracy: GPT-J=0.833±0.083, Llama=0.833±0.053, Mistral=0.792±0.077, random forest=0.750±0.083, XGBoost=0.625±0.042 (LLM epochs = 120, LLM learning rate = 0.001, random forest and XGBoost=default parameters, training set size = 15 data points).

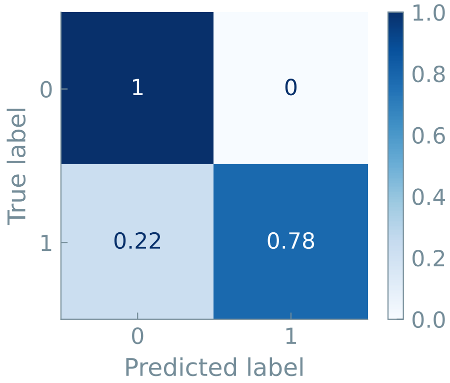


**Figure 150. Normalized confusion matrix, averaged over five independent runs, on the holdout test data for the prediction of sensor stability with the GPT-J model.** Models were trained using an unbalanced dataset with 63% of labels equal to 1, a training set of 15 data points, and 120 epochs (accuracy = 83%).

## 5.7 Gasification of Biomass

The dataset was provided by: María Victoria Gil and Covadonga Pevida[27]

### 5.7.1 Scientific Background

Bioenergy is one of the key pillars that will decarbonize our global energy systems in the coming years. Biomass is a versatile renewable resource that can replace fossil fuels and generate negative emissions. Gasification, which is the thermochemical conversion by partial oxidation at high temperatures of a solid carbonaceous feedstock into a gaseous product, is the most promising route for biomass valorization due to two main advantages of this process: high flexibility in terms of feedstock and versatility to produce different energy carriers. In fact, the gaseous stream produced, i.e., the syngas, can be used as fuel gas for heat and power generation and as feedstock for producing hydrogen, biofuels, and chemicals (see Figure 152).[92,93]

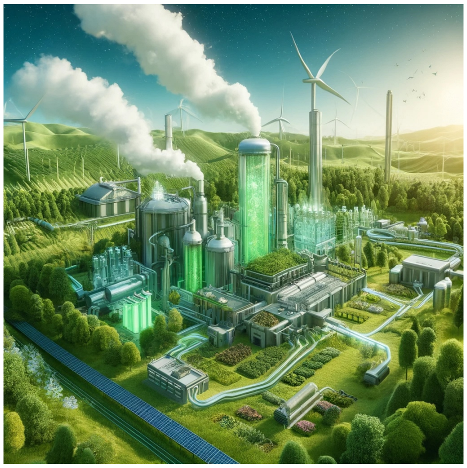

**Figure 151.** AI generated representation of bioenergy production.

Gil et al.[94] developed a model to predict the main outputs of the biomass gasification process from biomass properties and process conditions. From the predictions of the volume concentrations of $H_2$ and CO, the $H_2$/CO ratio in the synthesis gas can be calculated. These authors generated a dataset from this model, which included the gasification results of a series of biomasses whose characteristics were extracted from the literature. Using the molar $H_2$/CO ratio in the syngas, it is possible to classify whether a particular type of biomass is expected to be good for conversion to chemicals and fuels. This means to answer the question of whether biomass gasification produces syngas with a $H_2$/CO ratio greater than 1.8 since high ratios are required to use the syngas to synthesize fuel and chemicals.[95–98]

Given the broad range of possibilities related to the diversity of available biomasses and the versatility of the gasification gaseous product, predicting the syngas' characteristics will be crucial from a practical point of view to promote the development of the technology. Therefore, in this work, we use our LLMs approach[9] to build a machine learning model to predict the $H_2$/CO ratio in syngas obtained from biomass gasification using biomass properties as inputs.

[27] Instituto de Ciencia y Tecnología del Carbono (INCAR), CSIC, Francisco Pintado Fe 26, 33011 Oviedo, Spain

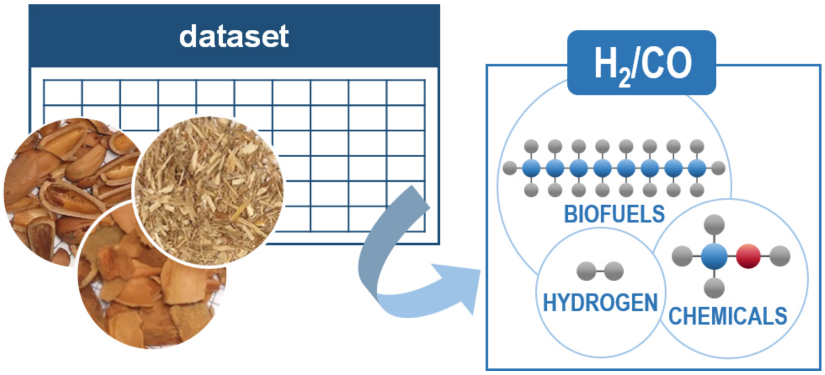


**Figure 152. Schematic representation of the biomass gasification case study.**

### 5.7.2 Dataset

We used the dataset generated by Gil et al.[94] on the $H_2$/CO ratio in syngas obtained from biomass gasification at 1173 K, steam-to-air ratio of 2.33 and stoichiometric ratio (SR) of 0.25. The dataset contains 50 data points, including the main characteristics of different biomass types, such as name, contents of C, H, O, ash, volatile matter (VM), and fixed carbon (FC) (wt%, db), as well as moisture content (MC) (wt%) and higher heating value (HHV) (MJ/kg).

We predict the $H_2$/CO ratio in syngas from biomass gasification. The distribution of this variable is shown in Figure 153. To predict the $H_2$/CO ratio in syngas from biomass gasification, we used the following variables as inputs: biomass name, contents of C, H, O, ash, volatile matter (VM), fixed carbon (FC) (wt%, db), moisture content (MC) (wt%), and biomass higher heating value (HHV) (MJ/kg).

We used a simple prompt template shown in Table 61 for experiments to predict $H_2$/CO ratio.

### 5.7.3 LLM results

**Base Case** To train the binary classification models, we split the dataset into two classes of equal size based on the syngas $H_2$/CO ratio separated by the median, i.e., 1.4. To avoid NaN or not valid predictions, we tested different numbers of epochs with the GPT-J model, as shown in Figure 154. We find that the model trained with 100 and 140 epochs performs better than random guesses (shown by the dashed line), with an accuracy of 78% for a training set of 45 data points and 100 epochs.

Therefore, three base LLMs, i.e., GPT-J, Llama, and Mistral, were fine-tuned /using 100 epochs. We also trained two “traditional” ML models, i.e., XGBoost and random Forest (RF), for comparison purposes. Table 62 and Figure 155 show that the highest accuracy (87%) was obtained with XGBoost. Lower performance was obtained with other models (68-78%).

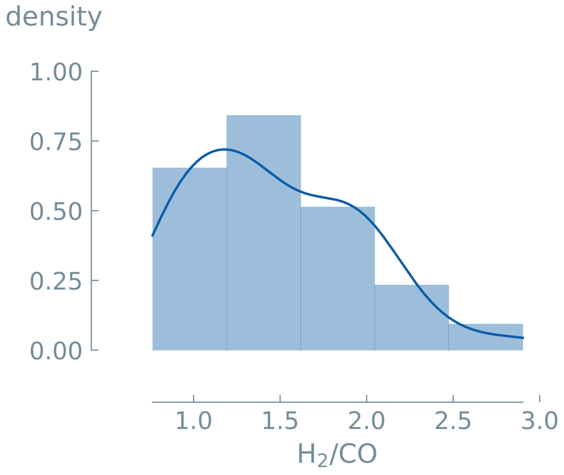


**Figure 153. Distribution of the syngas $H_2$/CO ratio in the dataset.** The median of the syngas $H_2$/CO ratio was is 1.4.

As an example, Figure 156 shows an averaged (over five independent runs) normalized confusion matrix for the GPT-J model trained using a training set of 45 data points and 100 epochs. We can see that the model often fails to predict samples labeled '1'.

**Real Split** To estimate if the gasification process of a given biomass produces syngas with a $H_2$/CO ratio higher than 1.8, a binary classification GPT-J model was trained with an unbalanced dataset since the data points with such values represent the 30% of the overall dataset. Figure 157 shows that this model does not perform better than random guess (shown by the dashed line), obtaining an accuracy of 70% when using a training set of 45 data points and 100 epochs.

Three LLMs (GPT-J, Llama, and Mistral) and two "traditional" ML models (XGBoost and RF) were also fine-tuned with an unbalanced dataset using 100 epochs. Figure 158 shows similar accuracy values for the LLMs and RF (70-72%), and a slightly higher accuracy for XGBoost (84%) when trained using an unbalanced dataset.

As an example, Figure 159a shows an averaged (over five independent runs) normalized confusion matrix for the GPT-J model trained with this unbalanced dataset using a training set of 45 data points and 140 epochs. We can see that the right predictions are close to the random guess.

However, we obtained better predictions of the syngas $H_2$/CO ratio values higher than 1.8 when we used a balanced dataset created by undersampling the majority class (label = 0) at the cost of reducing the size of the dataset. An accuracy of 70% was achieved with the GPT-J model using a training set of 25 data points and 140 fine-tuning epochs, which is slightly lower than that achieved with the initial balanced 50/50% larger dataset. The

**Table 61. Example prompts and completions for predicting the $H_2$/CO ratio in syngas from the inputs variables studied.**

| prompt | completion | experimental |
|---|---|---|
| What is the $H_2$/CO ratio in syngas obtained from steam gasification of <biomass name> with C content of <carbon content>%, H content of <hydrogen content>%, O content of <oxygen content>%, VM content of <volatile matter content>%, FC content of <fixed carbon content>%, ash content of <ash content>%, MC (%) content of <moisture content>, and HHV of <higher heating value> MJ/kg? | 0 | Low |
| What is the $H_2$/CO ratio in syngas obtained from steam gasification of <biomass name> with C content of <carbon content>%, H content of <hydrogen content>%, O content of <oxygen content>%, VM content of <volatile matter content>%, FC content of <fixed carbon content>%, ash content of <ash content>%, MC (%) content of <moisture content>, and HHV of <higher heating value> MJ/kg? | 1 | High |

**Table 62. Overview of the accuracy results of LLMs and "traditional" ML models for binary classification (balanced classes) of the $H_2$/CO ratio in syngas obtained from biomass gasification.** Ten runs were performed to get the metrics average. LLMs were fine-tuned with 100 epochs and a learning rate of 0.0003. Maximum performances are highlighted in bold.

| Size | Model | Accuracy | F1 Macro | F1 Micro | Kappa |
|---|---|---|---|---|---|
| 45 | GPT-J (LLM) | 0.78 | 0.74 | 0.78 | 0.54 |
| | Llama (LLM) | 0.76 | 0.74 | 0.76 | 0.53 |
| | Mistral (LLM) | 0.68 | 0.61 | 0.68 | 0.28 |
| | RF | 0.76 | 0.72 | 0.76 | 0.51 |
| | **XGBoost** | **0.87** | **0.81** | **0.87** | **0.68** |
| | Zero-rule | 0.50 | 0.50 | 0.50 | 0.00 |

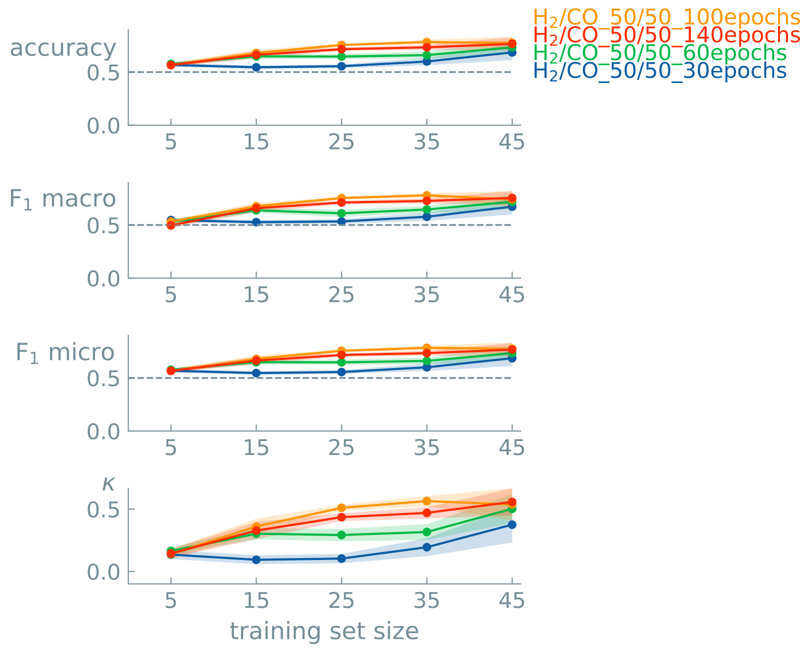


**Figure 154. Learning curves for binary classification GPT-J models (balanced classes) for the $H_2/CO$ ratio in syngas obtained from biomass gasification fine-tuned with different number of epochs.** Data points indicate the mean value of ten different experiments. Error bands show the standard error of the mean. We used 0.50 as random guess accuracy (dashed line), representing the zero rule baseline, i.e., a model that always predicts the most common class. Accuracy = 0.775±0.059 (epochs = 100, learning rate = 0.0003, training set size = 45 data points).

normalized confusion matrix in Figure 159b shows that the proportion of right predictions is above the random guess in the case of a smaller balanced dataset. However, if we compared these results with those in Figure 156, we can deduce that a slightly higher accuracy value is achieved when using more training data points.

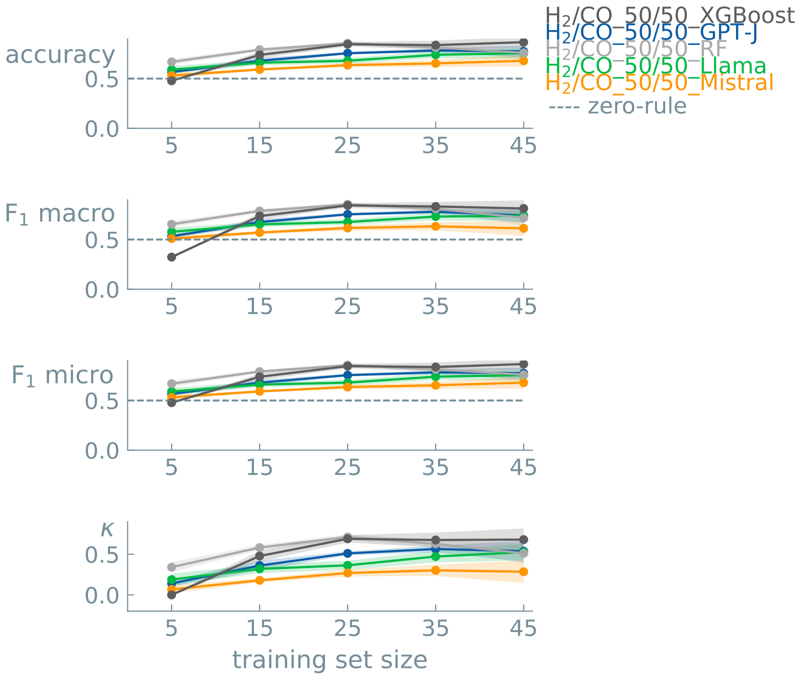


**Figure 155. Learning curves for binary classification models (balanced classes) for the $H_2$/CO ratio in syngas obtained from biomass gasification.** Data points indicate the mean value of ten different experiments. Error bands show the standard error of the mean. We used 0.50 as random guess accuracy (dashed line), representing the zero rule baseline, i.e., a model that always predicts the most common class. Accuracy: GPT-J=0.775±0.059, Llama=0.756±0.056, Mistral=0.680±0.061, random forest=0.760±0.065, XGBoost=0.867±0.054 (LLM epochs = 100, LLM learning rate = 0.0003, random forest and XGBoost=default parameters, training set size = 45 data points).

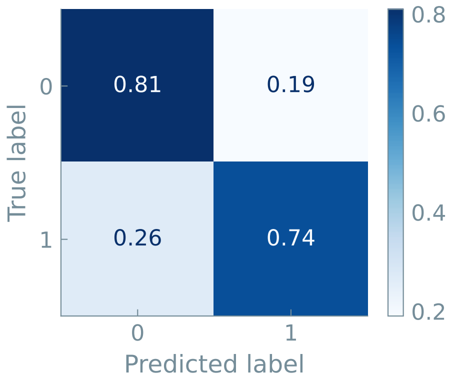


**Figure 156. Normalized confusion matrix, averaged over ten independent runs, on the holdout test data for syngas $H_2$/CO ratio prediction with the GPT-J model.** Models were trained in an balanced dataset using a training set of 45 data points and 100 epochs (accuracy = 78%).

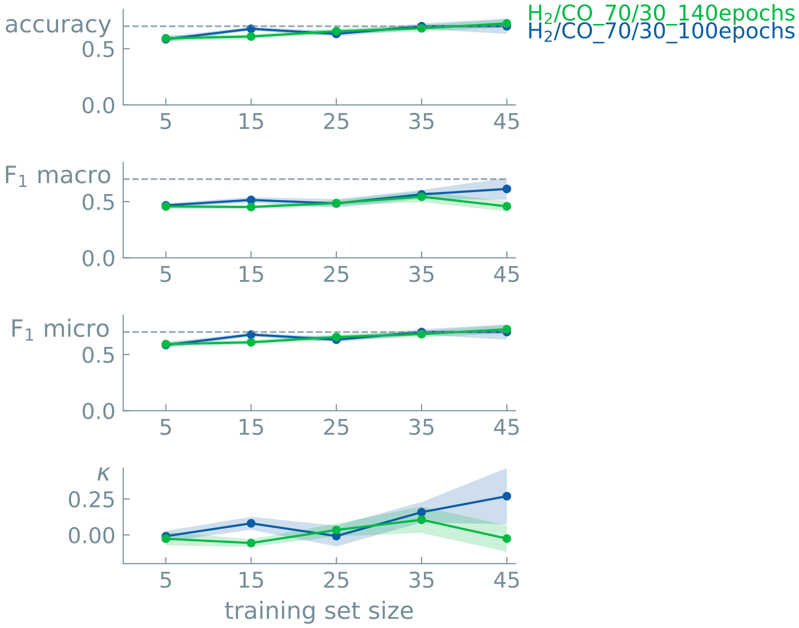


**Figure 157. Learning curves for binary classification GPT-J models (unbalanced classes, 70/30%) for the $H_2$/CO ratio in syngas obtained from biomass gasification fine-tuned with different number of epochs.** Data points indicate the mean value of ten different experiments. Error bands show the standard error of the mean. We used 0.70 as a random guess accuracy (dashed line), representing the zero rule baseline, i.e., a model that always predicts the most common class. Accuracy = 0.700±0.068 (epochs = 100, learning rate = 0.0003, training set size = 45 data points).

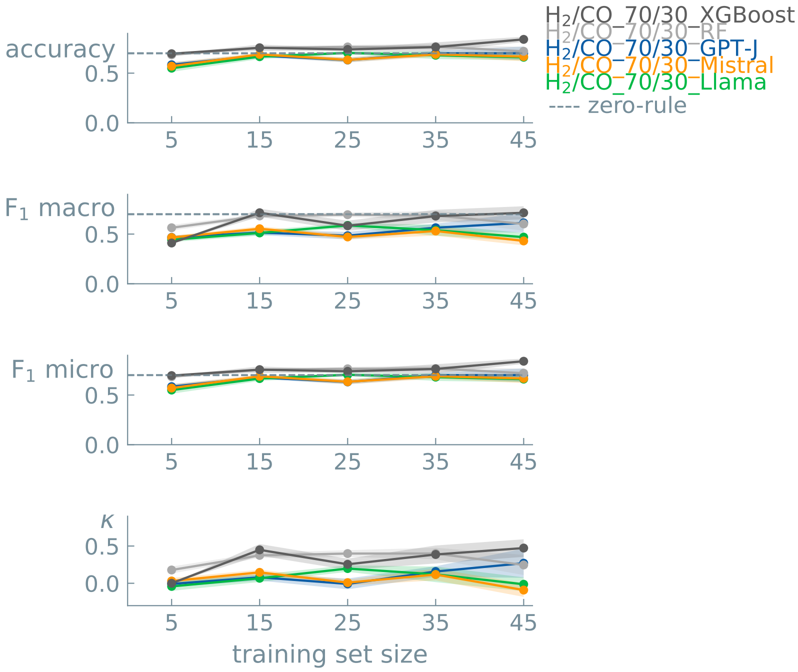


**Figure 158. Learning curves for binary classification models (unbalanced classes, 70/30%) for the $H_2/CO$ ratio in syngas obtained from biomass gasification.** Data points indicate the mean value of ten different experiments. Error bands show the standard error of the mean. We used 0.70 as a random guess accuracy (dashed line), representing the zero rule baseline, i.e., a model that always predicts the most common class. Accuracy: GPT-J=0.700±0.068, Llama=0.660±0.031, Mistral=0.667±0.047, random forest=0.720±0.044, XGBoost=0.840±0.026 (LLM epochs = 100, LLM learning rate = 0.0003, random forest and XGBoost=default parameters, training set size = 45 data points).

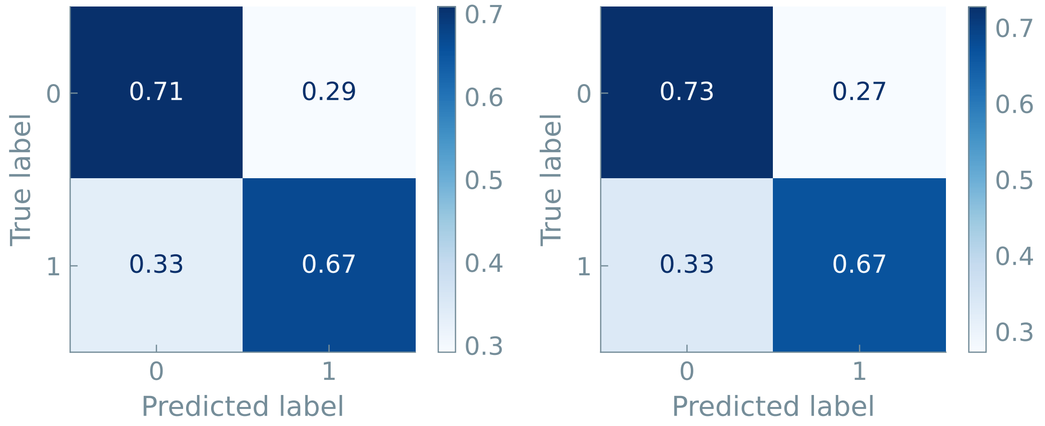


(a) (b)

**Figure 159. Normalized confusion matrix, averaged over ten independent runs, on the holdout test data for BET surface area prediction with the GPT-J model.** Models trained using an 'unbalanced' dataset with 70% of labels equal to 0, a training set of 45 data points and 100 epochs (accuracy = 70%) (a), and using a 'balanced' dataset with a training set of 25 data points and 140 epochs (accuracy = 70%) (b).

# Author contributions

| | Conceptualization | Data curation | Formal analysis | Funding acquisition | Investigation | Methodology | Project administration | Resources | Software | Supervision | Validation | Visualization | Writing – original draft | Writing – review & editing |
|---|---|---|---|---|---|---|---|---|---|---|---|---|---|---|
| Joren van Herk | ■ | ■ | ■ | | ■ | ■ | ■ | ■ | ■ | | ■ | ■ | ■ | ■ |
| María Victoria Gil | ■ | ■ | ■ | ■ | ■ | ■ | ■ | ■ | ■ | | ■ | ■ | ■ | ■ |
| Kevin Maik Jablonka | ■ | ■ | ■ | ■ | ■ | ■ | ■ | ■ | ■ | ■ | ■ | ■ | ■ | ■ |
| Alex Abrudan | | ■ | | | | | | | | | | | | ■ |
| Andy S. Anker | | ■ | | | | | | | | | | | | ■ |
| Mehrdad Asgari | | ■ | | | | | | | | ■ | | ■ | | ■ |
| Ben Blaiszik | | ■ | | ■ | | | | | | ■ | | | | ■ |
| Antonio Buffo | | ■ | | ■ | | | | | | ■ | | | | ■ |
| Leander Choudhury | | ■ | | | | | | | | | | | | ■ |
| Clemence Corminboeuf | | ■ | | ■ | | | | | | ■ | | | | ■ |
| Hilal Daglar | | ■ | | | | | | | | | | | | ■ |
| Amir Mohammad Elahi | | ■ | ■ | | | | | | ■ | | | | | ■ |
| Ian T. Foster | | ■ | | ■ | | | | | | ■ | | | | ■ |
| Susana Garcia | | ■ | | ■ | | | | | | ■ | | | | ■ |
| Matthew Garvin | | ■ | | | | | | | | | | ■ | | ■ |
| Guillaume Godin | | ■ | | | | | | | | | | | | ■ |
| Lydia L. Good | | ■ | | | | | | | | | | ■ | | ■ |
| Jianan Gu | | ■ | | | | | | | | | | | | ■ |
| Noemie Xiao Hu | | ■ | | | | | | | | | | | | ■ |
| Xin Jin | | ■ | | | | | | | | | | | | ■ |
| Tanja Junkers | | ■ | | ■ | | | | | | ■ | | | | ■ |
| Seda Keskin | | ■ | | ■ | | | | | | ■ | | | | ■ |
| Tuomas P.J. Knowles | | ■ | | ■ | | | | | | ■ | | | | ■ |
| Ruben Laplaza | | ■ | | | | | | | | | | ■ | | ■ |
| Michele Lessona | | ■ | | | | | | | | | | ■ | | ■ |
| Sauradeep Majumdar | | ■ | | | | | | | | | | | | ■ |
| Hossein Mashhadimoslem | | ■ | | | | | | | | | | | | ■ |
| Ruaraidh D. McIntosh | | ■ | | ■ | | | | | | ■ | | | | ■ |
| Seyed Mohamad Moosavi | | ■ | | ■ | | | | | | ■ | | | | ■ |
| Beatriz Mourino | | ■ | | | | | | | | | | | | ■ |
| Francesca Nerli | | ■ | | | | | | | | | | | | ■ |
| Covadonga Pevida | | ■ | | ■ | | | | | | | | | | ■ |
| Neda Poudineh | | ■ | | | | | | | | | | | | ■ |
| Mahyar Rajabi-Kochi | | ■ | | | | | | | | | | | | ■ |
| Kadi L. Saar | | ■ | | | | | | | | | | | | ■ |
| Fahimeh H. Saboor | | ■ | | | | | | | | ■ | | | | ■ |
| Morteza Sagharichiha | | ■ | | | | | | | | | | | | ■ |
| KJ Schmidt | | ■ | | ■ | | | | | | ■ | | | | ■ |
| Jiale Shi | | ■ | | | | | | | | | | | | ■ |
| Elena Simone | | ■ | | ■ | | | | | | | | | | ■ |
| Dennis Svatunek | | ■ | | ■ | | | | | | | | | | ■ |
| Marco Taddei | | ■ | | ■ | | | | | | ■ | | ■ | | ■ |
| Igor Tetko | | ■ | | ■ | | | | | | ■ | | | | ■ |
| Domonkos Tolnai | | ■ | | ■ | | | | | | ■ | | | | ■ |
| Sahar Vahdatifar | | ■ | | | | | | | | | | | | ■ |
| Jonathan Whitmer | | ■ | | | | | | | | ■ | | | | ■ |
| Florian Wieland | | ■ | | ■ | | | | | | ■ | | | | ■ |
| Regine Willumeit-Romer | | ■ | | ■ | | | | | | ■ | | | | ■ |
| Andreas Zuttel | | ■ | | ■ | | | | | | | | | | ■ |
| Berend Smit | ■ | ■ | ■ | ■ | ■ | ■ | ■ | ■ | ■ | ■ | ■ | ■ | ■ | ■ |

## References

[1] Wang, B.; Komatsuzaki, A. GPT-J-6B: A 6 Billion Parameter Autoregressive Language Model. https://github.com/kingoflolz/mesh-transformer-jax, 2021.

[2] Wang, B. Mesh-Transformer-JAX: Model-Parallel Implementation of Transformer Language Model with JAX. https://github.com/kingoflolz/mesh-transformer-jax, 2021.

[3] Gao, L.; Biderman, S.; Black, S.; Golding, L.; Hoppe, T.; Foster, C.; Phang, J.; He, H.; Thite, A.; Nabeshima, N.; Presser, S.; Leahy, C. The Pile: An 800GB Dataset of Diverse Text for Language Modeling. *arXiv preprint Arxiv-2101.00027* **2020**,

[4] Dubey, A. et al. The Llama 3 Herd of Models. 2024; https://arxiv.org/abs/2407.21783.

[5] Jiang, A. Q.; Sablayrolles, A.; Mensch, A.; Bamford, C.; Chaplot, D. S.; Casas, D. d. l.; Bressand, F.; Lengyel, G.; Lample, G.; Saulnier, L., et al. Mistral 7B. *arXiv preprint arXiv:2310.06825* **2023**,

[6] Dettmers, T.; Lewis, M.; Belkada, Y.; Zettlemoyer, L. GPT3.int8(): 8-bit Matrix Multiplication for Transformers at Scale. Advances in Neural Information Processing Systems. 2022.

[7] Dettmers, T.; Lewis, M.; Shleifer, S.; Zettlemoyer, L. 8-bit Optimizers via Block-wise Quantization. The Tenth International Conference on Learning Representations, ICLR. 2022.

[8] Hu, E. J.; Shen, Y.; Wallis, P.; Allen-Zhu, Z.; Li, Y.; Wang, S.; Chen, W. LoRA: Low-Rank Adaptation of Large Language Models. International Conference On Learning Representations. 2021.

[9] Jablonka, K. M.; Schwaller, P.; Ortega-Guerrero, A.; Smit, B. Leveraging large language models for predictive chemistry. *Nature Machine Intelligence* **2024**, DOI: 10.1038/s42256-023-00788-1.

[10] Shi, J.; Quevillon, M. J.; Valença, P. H. A.; Whitmer, J. K. Predicting Adhesive Free Energies of Polymer–Surface Interactions with Machine Learning. *ACS Appl. Mater. Interfaces* **2022**, *32*, 37161–37169.

[11] Schneider, L.; Schwarting, M.; Mysona, J.; Liang, H.; Han, M.; Rauscher, P. M.; Ting, J. M.; Venkatram, S.; Ross, R. B.; Schmidt, K. J.; Blaiszik, B.; Foster, I.; de Pablo, J. J. *In silico* active learning for small molecule properties. *Mol. Syst. Des. Eng.* **2022**, *7*, 1611–1621, DOI: 10.1039/d2me00137c.

[12] Tetko, I. V.; Sushko, Y.; Novotarskyi, S.; Patiny, L.; Kondratov, I.; Petrenko, A. E.; Charochkina, L.; Asiri, A. M. How Accurately Can We Predict the Melting Points of Drug-like Compounds? *Journal of Chemical Information and Modeling* **2014**, *54*, 3320–3329, DOI: 10.1021/ci5005288, PMID: 25489863.

[13] Tetko, I. V.; M. Lowe, D.; Williams, A. J. The development of models to predict melting and pyrolysis point data associated with several hundred thousand compounds mined from PATENTS. *Journal of Cheminformatics* **2016**, *8*, DOI: 10.1186/s13321-016-0113-y.

[14] Beckner, W.; Mao, C. M.; Pfaendtner, J. Statistical models are able to predict ionic liquid viscosity across a wide range of chemical functionalities and experimental conditions. *Molecular Systems Design & Engineering* **2018**, *3*, 253–263.

[15] Viswanath, D. S.; Ghosh, T. K.; Prasad, D. H.; Dutt, N. V.; Rani, K. Y. *Viscosity of liquids: theory, estimation, experiment, and data*; Springer Science & Business Media, 2007.

[16] Goussard, V.; Duprat, F.; Ploix, J.-L.; Dreyfus, G.; Nardello-Rataj, V.; Aubry, J.-M. A new machine-learning tool for fast estimation of liquid viscosity. application to cosmetic oils. *Journal of Chemical Information and Modeling* **2020**, *60*, 2012–2023.

[17] Dieringa, H.; Kainer, K. U. In *Springer Handbook of Materials Data*; Warlimont, H., Martienssen, W., Eds.; Springer International Publishing: Cham, 2018; pp 151–159.

[18] In vivo corrosion of four magnesium alloys and the associated bone response. *Biomaterials* **2005**, *26*, 3557–3563.

[19] Wolff, M.; Ebel, T.; Dahms, M. Sintering of Magnesium. *Advanced Engineering Materials* **2010**, *12*, 829–836, DOI: https://doi.org/10.1002/adem.201000038.

[20] Wolff, M.; Schaper, J. G.; Suckert, M. R.; Dahms, M.; Feyerabend, F.; Ebel, T.; Willumeit-Römer, R.; Klassen, T. Metal Injection Molding (MIM) of Magnesium and Its Alloys. *Metals* **2016**, *6*.

[21] Wolff, M.; Schaper, J. G.; Dahms, M.; Ebel, T.; Kainer, K. U.; Klassen, T. Magnesium powder injection moulding for biomedical application. *Powder Metallurgy* **2014**, *57*, 331–340, DOI: 10.1179/1743290114Y.0000000111.

[22] Pekguleryuz, M. O., Kainer, K. U., Arslan Kaya, A., Eds. *Fundamentals of Magnesium Alloy Metallurgy*; Woodhead Publishing Series in Metals and Surface Engineering; Woodhead Publishing, 2013; p iv, DOI: https://doi.org/10.1016/B978-0-85709-088-1.50012-4.

[23] Humphreys, F., Hatherly, M., Eds. *Recrystallization and Related Annealing Phenomena (Second Edition)*, second edition ed.; Elsevier: Oxford, 2004; pp 527–540, DOI: https://doi.org/10.1016/B978-008044164-1/50021-9.

[24] El-Sherik, A., Ed. *Trends in Oil and Gas Corrosion Research and Technologies*; Woodhead Publishing Series in Energy; Woodhead Publishing: Boston, 2017; pp 295–314, DOI: https://doi.org/10.1016/B978-0-08-101105-8.00012-7.

[25] Harmuth, J.; Wiese, B.; Bohlen, J.; Ebel, T.; Willumeit-Römer, R. Wide Range Mechanical Customization of Mg-Gd Alloys With Low Degradation Rates by Extrusion. *Frontiers in Materials* **2019**, *6*, DOI: 10.3389/fmats.2019.00201.

[26] Mingo, B.; Mohedano, M.; Blawert, C.; del Olmo, R.; Hort, N.; Arrabal, R. Role of Ca on the corrosion resistance of Mg–9Al and Mg–9Al–0.5Mn alloys. *Journal of Alloys and Compounds* **2019**, *811*, 151992, DOI: https://doi.org/10.1016/j.jallcom.2019.151992.

[27] Mingo, B.; Arrabal, R.; Mohedano, M.; Mendis, C.; del Olmo, R.; Matykina, E.; Hort, N.; Merino, M.; Pardo, A. Corrosion of Mg-9Al alloy with minor alloying elements (Mn, Nd, Ca, Y and Sn). *Materials & Design* **2017**, *130*, 48–58, DOI: https://doi.org/10.1016/j.matdes.2017.05.048.

[28] Gao, M.; Yang, K.; Tan, L.; Ma, Z. Role of bimodal-grained structure with random texture on mechanical and corrosion properties of a Mg-Zn-Nd alloy. *Journal of Magnesium and Alloys* **2022**, *10*, 2147–2157, DOI: https://doi.org/10.1016/j.jma.2021.03.024.

[29] Bahmani, A.; Arthanari, S.; Shin, K. S. Corrosion behavior of Mg–Mn–Ca alloy: Influences of Al, Sn and Zn. *Journal of Magnesium and Alloys* **2019**, *7*, 38–46, DOI: https://doi.org/10.1016/j.jma.2018.11.004.

[30] Effects of secondary phase and grain size on the corrosion of biodegradable Mg–Zn–Ca alloys. *Materials Science and Engineering: C* **2015**, *48*, 480–486, DOI: https://doi.org/10.1016/j.msec.2014.12.049.

[31] In- vitro corrosion behavior of the cast and extruded biodegradable Mg-Zn-Cu alloys in simulated body fluid (SBF). *Journal of Magnesium and Alloys* **2021**, *9*, 2078–2096, DOI: https://doi.org/10.1016/j.jma.2021.01.002.

[32] Influence of the amount of intermetallics on the degradation of Mg-Nd alloys under physiological conditions. *Acta Biomaterialia* **2021**, *121*, 695–712.

[33] Cai, C.; Song, R.; Wang, L.; Li, J. Effect of anodic T phase on surface micro-galvanic corrosion of biodegradable Mg-Zn-Zr-Nd alloys. *Applied Surface Science* **2018**, *462*, 243–254, DOI: https://doi.org/10.1016/j.apsusc.2018.08.107.

[34] Boeynaems, S.; Alberti, S.; Fawzi, N. L.; Mittag, T.; Polymenidou, M.; Rousseau, F.; Schymkowitz, J.; Shorter, J.; Wolozin, B.; Van Den Bosch, L.; Tompa, P.; Fuxreiter, M.

Protein Phase Separation: A New Phase in Cell Biology. *Trends in Cell Biology* **2018**, *28*, 420–435, DOI: https://doi.org/10.1016/j.tcb.2018.02.004.

[35] Saar, K. L.; Morgunov, A. S.; Qi, R.; Arter, W. E.; Krainer, G.; Lee, A. A.; Knowles, T. P. Learning the molecular grammar of protein condensates from sequence determinants and embeddings. *Proceedings of the National Academy of Sciences* **2021**, *118*, e2019053118.

[36] Mikolov, T.; Chen, K.; Corrado, G.; Dean, J. Efficient Estimation of Word Representations in Vector Space. *Proceedings of Workshop at ICLR* **2013**, *2013*.

[37] Li, Q.; Peng, X.; Li, Y.; Tang, W.; Zhu, J.; Huang, J.; Qi, Y.; Zhang, Z. LLPSDB: a database of proteins undergoing liquid–liquid phase separation in vitro. *Nucleic acids research* **2020**, *48*, D320–D327.

[38] Berman, H. M.; Westbrook, J.; Feng, Z.; Gilliland, G.; Bhat, T. N.; Weissig, H.; Shindyalov, I. N.; Bourne, P. E. The protein data bank. *Nucleic acids research* **2000**, *28*, 235–242.

[39] Tsai, W.-C.; Gayatri, S.; Reineke, L. C.; Sbardella, G.; Bedford, M. T.; Lloyd, R. E. Arginine Demethylation of G3BP1 Promotes Stress Granule Assembly*. *Journal of Biological Chemistry* **2016**, *291*, 22671–22685, DOI: https://doi.org/10.1074/jbc.M116.739573.

[40] Billinge, S. J. L.; Kanatzidis, M. G. Beyond crystallography: the study of disorder, nanocrystallinity and crystallographically challenged materials with pair distribution functions. *Chem. Commun. (Camb.)* **2004**, 749–760.

[41] Anker, A. S.; T. S. Kjær, E.; Dam, E. B.; J. L. Billinge, S.; Jensen, K. M. O.; Selvan, R. Characterising the Atomic Structure of Mono-Metallic Nanoparticles from X-Ray Scattering Data Using Conditional Generative Models. **2020**, DOI: 10.26434/chemrxiv.12662222.v1.

[42] Kjær, E. T. S.; Anker, A. S.; Weng, M. N.; Billinge, S. J. L.; Selvan, R.; Jensen, K. M. O. DeepStruc: towards structure solution from pair distribution function data using deep generative models. *Digital Discovery* **2023**, *2*, 69–80, DOI: 10.1039/d2dd00086e.

[43] Marangoni, A. G.; Wesdorp, L. H. *Structure and properties of fat crystal networks*; CRC Press, 2012; DOI: 10.1201/b12883.

[44] Pereira, E.; Junqueira, F. T.; Meirelles, A. J. D.; Maximo, G. J. Prediction of the melting behavior of edible fats using UNIFAC and UNIQUAC models. *Fluid Phase Equilibr* **2019**, *493*, 58–66, DOI: 10.1016/j.fluid.2019.04.004.

[45] Schaink, H. M. Calculation of the solid fat content of vegetable fats using the Hildebrand equation. *J Am Oil Chem Soc* **2023**, *100*, 929–944, DOI: 10.1002/aocs.12724.

[46] Ollivon, M.; Perron, R. Measurements of Enthalpies and Entropies of Unstable Crystalline Forms of Saturated Even Monoacid Triglycerides. *Thermochim Acta* **1982**, *53*, 183–194, DOI: 10.1016/0040-6031(82)85007-7.

[47] Seilert, J.; Flöter, E. A Configurational Approach to Model Triglyceride Pure Component Properties. *Eur J Lipid Sci Tech* **2021**, *123*, DOI: 10.1002/ejlt.202100010.

[48] Moorthy, A. S.; Liu, R.; Mazzanti, G.; Wesdorp, L. H.; Marangoni, A. G. Estimating Thermodynamic Properties of Pure Triglyceride Systems Using the Triglyceride Property Calculator. *J Am Oil Chem Soc* **2017**, *94*, 187–199, DOI: 10.1007/s11746-016-2935-1.

[49] Seilert, J.; Moorthy, A. S.; Kearsley, A. J.; Flöter, E. Revisiting a model to predict pure triglyceride thermodynamic properties: parameter optimization and performance. *J Am Oil Chem Soc* **2021**, *98*, 837–850, DOI: 10.1002/aocs.12515.

[50] Kolb, H. C.; Finn, M. G.; Sharpless, K. B. Click Chemistry: Diverse Chemical Function from a Few Good Reactions. *Angewandte Chemie International Edition* **2001**, *40*, 2004–2021, DOI: https://doi.org/10.1002/1521-3773(20010601)40:11<2004::AID-ANIE2004>3.0.CO;2-5.

[51] Houszka, N.; Mikula, H.; Svatunek, D. Substituent Effects in Bioorthogonal Diels–Alder Reactions of 1,2,4,5-Tetrazines. *Chemistry – A European Journal* **2023**, *29*, e202300345, DOI: https://doi.org/10.1002/chem.202300345.

[52] Svatunek, D. Computational Large-Scale Screening of Bioorthogonal 1,2,4,5-Tetrazine/trans-Cyclooctene Cycloadditions. *ChemRxiv* **2024**, DOI: https://doi.org/10.26434/chemrxiv-2024-24xcv.

[53] Busch, M.; Wodrich, M. D.; Corminboeuf, C. Linear scaling relationships and volcano plots in homogeneous catalysis – revisiting the Suzuki reaction. *Chem. Sci.* **2015**, *6*, 6754–6761, DOI: 10.1039/C5SC02910D.

[54] Laplaza, R.; Das, S.; Wodrich, M. D.; Corminboeuf, C. Constructing and interpreting volcano plots and activity maps to navigate homogeneous catalyst landscapes. *Nature Protocols* **2022**, *17*, 2550–2569.

[55] Cordova, M.; Wodrich, M. D.; Meyer, B.; Sawatlon, B.; Corminboeuf, C. Data-Driven Advancement of Homogeneous Nickel Catalyst Activity for Aryl Ether Cleavage. *ACS Catal.* **2020**, *10*, 7021–7031, DOI: 10.1021/acscatal.0c00774.

[56] Soengas, R. G.; Rodríguez-Solla, H. Modern synthetic methods for the stereoselective construction of 1,3-dienes. *Molecules* **2021**, *26*, 249, DOI: 10.3390/molecules26020249.

[57] Smyrnov, V.; Homassel, A.; Choudhury, L.; Waser, J. Isomerization of Cyclopropenes to 1,3-Dienes. **2024**, DOI: 10.26434/chemrxiv-2024-ckv1v, This content is a preprint and has not been peer-reviewed.

[58] Van Herck, J.; Abeysekera, I.; Buckinx, A.-L.; Cai, K.; Hooker, J.; Thakur, K.; Van de Reydt, E.; Voorter, P.-J.; Wyers, D.; Junkers, T. Operator-independent high-throughput polymerization screening based on automated inline NMR and online SEC. *Digital Discovery* **2022**, *1*, 519–526, DOI: 10.1039/D2DD00035K.

[59] Luo, T.; Gilmanova, L.; Kaskel, S. Advances of MOFs and COFs for photocatalytic CO2 reduction, H2 evolution and organic redox transformations. *Coordination Chemistry Reviews* **2023**, *490*, 215210, DOI: https://doi.org/10.1016/j.ccr.2023.215210.

[60] Fumanal, M.; Ortega-Guerrero, A.; Jablonka, K. M.; Smit, B.; Tavernelli, I. Charge Separation and Charge Carrier Mobility in Photocatalytic Metal-Organic Frameworks. *Advanced Functional Materials* **2020**, *30*, 2003792, DOI: https://doi.org/10.1002/adfm.202003792.

[61] Rosen, A. S.; Iyer, S. M.; Ray, D.; Yao, Z.; Aspuru-Guzik, A.; Gagliardi, L.; Notestein, J. M.; Snurr, R. Q. Machine learning the quantum-chemical properties of metal–organic frameworks for accelerated materials discovery. *Matter* **2021**, *4*, 1578–1597, DOI: https://doi.org/10.1016/j.matt.2021.02.015.

[62] Mouriño, B.; Jablonka, K. M.; Ortega-Guerrero, A.; Smit, B. In Search of Covalent Organic Framework Photocatalysts: A DFT-Based Screening Approach. *Advanced Functional Materials* **2023**, *33*, 2301594, DOI: https://doi.org/10.1002/adfm.202301594.

[63] Bucior, B.; Rosen, A.; Haranczyk, M.; Yao, Z.; Ziebel, M.; Farha, O.; Hupp, J.; Siepmann, J.; Aspuru-Guzik, A.; Snurr, R. Identification Schemes for Metal–Organic Frameworks To Enable Rapid Search and Cheminformatics Analysis. *Crystal Growth & Design* **2019**, *19*, DOI: 10.1021/acs.cgd.9b01050.

[64] Behera, A.; Kar, A. K.; Srivastava, R. Challenges and prospects in the selective photoreduction of CO2 to C1 and C2 products with nanostructured materials: a review. *Mater. Horiz.* **2022**, *9*, 607–639, DOI: 10.1039/D1MH01490K.

[65] Khan, M.; Akmal, Z.; Tayyab, M.; Mansoor, S.; Zeb, A.; Ye, Z.; Zhang, J.; Wu, S.; Wang, L. MOFs materials as photocatalysts for CO2 reduction: Progress, challenges and perspectives. *Carbon Capture Science & Technology* **2024**, *11*, 100191, DOI: https://doi.org/10.1016/j.ccst.2024.100191.

[66] M.S, R.; Shanmuga Priya, S.; Freudenberg, N. C.; Sudhakar, K.; Tahir, M. Metal-organic framework-based photocatalysts for carbon dioxide reduction to methanol: A review on progress and application. *Journal of CO2 Utilization* **2021**, *43*, 101374, DOI: https://doi.org/10.1016/j.jcou.2020.101374.

[67] Hong-Cai Zhou, J. R. L.; Yaghi, O. M. Big-Data Science in Porous Materials: Materials Genomics and Machine Learning. *Chemical Reviews* **2012**, *112*, 673–674.

[68] Jablonka, K. M.; Ongari, D.; Moosavi, S. M.; Smit, B. Big-Data Science in Porous Materials: Materials Genomics and Machine Learning. *Chem. Rev.* **2020**, *120*, 8066–8129, DOI: 10.1021/acs.chemrev.0c00004.

[69] Li, H.; Li, L.; Lin, R.-B.; Zhou, W.; Zhang, Z.; Xiang, S.; Chen, B. Porous metal-organic frameworks for gas storage and separation: Status and challenges. *EnergyChem* **2019**, *1*, 100006, DOI: https://doi.org/10.1016/j.enchem.2019.100006.

[70] Daglar, H.; Keskin, S. Combining Machine Learning and Molecular Simulations to Unlock Gas Separation Potentials of MOF Membranes and MOF/Polymer MMMs. *ACS Applied Materials & Interfaces* **2022**, *14*, 32134–32148.

[71] Chung, Y. G.; Haldoupis, E.; Bucior, B. J.; Haranczyk, M.; Lee, S.; Zhang, H.; Vogiatzis, K. D.; Milisavljevic, M.; Ling, S.; Camp, J. S.; Slater, B.; Siepmann, J. I.; Sholl, D. S.; Snurr, R. Q. Advances, Updates, and Analytics for the Computation-Ready, Experimental Metal–Organic Framework Database: CoRE MOF 2019. *J. Chem. Eng. Data* **2019**, *64*, 5985–5998, DOI: 10.1021/acs.jced.9b00835.

[72] Abe, J.; Popoola, A.; Ajenifuja, E.; Popoola, O. Hydrogen energy, economy and storage: Review and recommendation. *International Journal of Hydrogen Energy* **2019**, *44*, 15072–15086, DOI: https://doi.org/10.1016/j.ijhydene.2019.04.068.

[73] Usman, M. R. Hydrogen storage methods: Review and current status. *Renewable and Sustainable Energy Reviews* **2022**, *167*, DOI: https://doi.org/10.1016/j.rser.2022.112743.

[74] Klopčič, N.; Grimmer, I.; Winkler, F.; Sartory, M.; Trattner, A. A review on metal hydride materials for hydrogen storage. *Journal of Energy Storage* **2023**, *72*, 108456, DOI: https://doi.org/10.1016/j.est.2023.108456.

[75] Griessen, R.; Riesterer, T. In *Hydrogen in Intermetallic Compounds I: Electronic, Thermodynamic, and Crystallographic Properties, Preparation*; Schlapbach, L., Ed.; Springer Berlin Heidelberg: Berlin, Heidelberg, 1988; pp 219–284, DOI: 10.1007/3540183337_13.

[76] Witman, M.; Ling, S.; Grant, D. M.; Walker, G. S.; Agarwal, S.; Stavila, V.; Allendorf, M. D. Extracting an Empirical Intermetallic Hydride Design Principle from Limited Data via Interpretable Machine Learning. *The Journal of Physical Chemistry Letters* **2019**, *11*, 40–47, DOI: https://doi.org/10.1021/acs.jpclett.9b02971.

[77] Witman, M.; Allendorf, M.; Stavila, V. Database for machine learning of hydrogen storage materials properties. **2022**, DOI: 10.5281/zenodo.7324809.

[78] Andreasen, A. Predicting formation enthalpies of metal hydrides. **2004**,

[79] Poojan Modi, K.-F. A.-Z. Room Temperature Metal Hydrides for Stationary and Heat Storage Applications: A Review. *Frontiers in Energy Research* **2021**, *6*, DOI: https://doi.org/10.3389/fenrg.2021.616115.

[80] Wang, J.; Huang, L.; Yang, R.; Zhang, Z.; Wu, J.; Gao, Y.; Wang, Q.; O'Hare, D.; Zhong, Z. Recent advances in solid sorbents for CO2 capture and new development trends. *Energy Environ. Sci.* **2014**, *7*, 3478–3518, DOI: 10.1039/C4EE01647E.

[81] Zhao, Y.; Liu, X.; Han, Y. Microporous carbonaceous adsorbents for $CO_2$ separation via selective adsorption. *RSC Adv.* **2015**, *58*, 30310–30330, DOI: 10.1039/C5RA00569H.

[82] Patel, H. A.; Byun, J.; Yavuz, C. T. Carbon Dioxide Capture Adsorbents: Chemistry and Methods. *ChemSusChem* **2017**, *10*, 1303–1317, DOI: https://doi.org/10.1002/cssc.201601545.

[83] Samanta, A.; Zhao, A.; Shimizu, G. K. H.; Sarkar, P.; Gupta, R. Post-Combustion $CO_2$ Capture Using Solid Sorbents: A Review. *Industrial & Engineering Chemistry Research* **2012**, *51*, 1438–1463, DOI: 10.1021/ie200686q.

[84] Rodríguez-Reinoso, F.; Molina-Sabio, M. Activated carbons from lignocellulosic materials by chemical and/or physical activation: an overview. *Carbon* **1992**, *30*, 1111–1118, DOI: https://doi.org/10.1016/0008-6223(92)90143-K.

[85] Mashhadimoslem, H.; Vafaeinia, M.; Safarzadeh, M.; Ghaemi, A.; Fathalian, F.; Maleki, A. Development of Predictive Models for Activated Carbon Synthesis from Different Biomass for $CO_2$ Adsorption Using Artificial Neural Networks. *Industrial and Engineering Chemistry Research* **2021**, *60*, 13950–13966, DOI: 10.1021/acs.iecr.1c02754.

[86] Darre, N.; Toor, G. Desalination of Water: a Review. *Current Pollution Reports* **2018**, *4*, 1–8, DOI: 10.1007/s40726-018-0085-9.

[87] Sagharichiha, M.; Jafarian, A.; Asgari, M.; Kouhikamali, R. Simulation of a forward feed multiple effect desalination plant with vertical tube evaporators. *Chemical Engineering and Processing: Process Intensification* **2014**, *75*, 110–118, DOI: 10.1016/j.cep.2013.11.008.

[88] Zheng, H. *Solar Energy Desalination Technology*; Elsevier, 2017; p 173–258, DOI: 10.1016/b978-0-12-805411-6.00003-8.

[89] Saboor, F. H.; Khodadadi, A. A.; Mortazavi, Y.; Asgari, M. Microemulsion synthesized silica/ZnO stable core/shell sensors highly selective to ethanol with minimum sensitivity to humidity. *Sensors and Actuators B: Chemical* **2017**, *238*, 1070–1083, DOI: 10.1016/j.snb.2016.07.127.

[90] Xu, C.; Tamaki, J.; Miura, N.; Yamazoe, N. Grain size effects on gas sensitivity of porous SnO2-based elements. *Sensors and Actuators B: Chemical* **1991**, *3*, 147–155, DOI: 10.1016/0925-4005(91)80207-z.

[91] Vahdatifar, S.; Khodadadi, A. A.; Mortazavi, Y. Effects of nanoadditives on stability of Pt/SnO2 as a sensing material for detection of CO. *Sensors and Actuators B: Chemical* **2014**, *191*, 421–430, DOI: 10.1016/j.snb.2013.10.010.

[92] Tezer, Ö.; Karabağ, N.; Öngen, A.; Çolpan, C. Ö.; Ayol, A. Biomass gasification for sustainable energy production: A review. *International Journal of Hydrogen Energy* **2022**, *11*, 811, DOI: https://doi.org/10.1016/j.ijhydene.2022.02.158.

[93] Narnaware, S. L.; Panwar, N. Biomass gasification for climate change mitigation and policy framework in India: A review. *Bioresource Technology Reports* **2022**, *17*, 100892, DOI: 10.1016/j.biteb.2021.100892.

[94] Gil, M. V.; Jablonka, K. M.; Garcia, S.; Pevida, C.; Smit, B. Biomass to energy: a machine learning model for optimum gasification pathways. *Digital Discovery* **2023**, *2*, 929–940, DOI: 10.1039/d3dd00079f.

[95] Wender, I. Reactions of synthesis gas. *Fuel Processing Technology* **1996**, *48*, 189–297, DOI: https://doi.org/10.1016/S0378-3820(96)01048-X.

[96] Luyben, W. L. Control of parallel dry methane and steam methane reforming processes for Fischer-Tropsch syngas. *Journal of Process Control* **2016**, *39*, 77–87, DOI: 10.1016/j.jprocont.2015.11.007.

[97] Hussain, I.; Jalil, A. A.; Mamat, C. R.; Siang, T. J.; Rahman, A. F.; Azami, M. S.; Adnan, R. H. New insights on the effect of the $H_2$/CO ratio for enhancement of CO methanation over metal-free fibrous silica ZSM-5: Thermodynamic and mechanistic studies. *Energy Conversion and Management* **2019**, *199*, 112056, DOI: 10.1016/j.enconman.2019.112056.

[98] Khademi, M. H.; Alipour-Dehkordi, A.; Tabesh, M. Optimal design of methane tri-reforming reactor to produce proper syngas for Fischer-Tropsch and methanol synthesis processes: A comparative analysis between different side-feeding strategies. *International Journal of Hydrogen Energy* **2021**, *46*, 14441–14454, DOI: 10.1016/j.ijhydene.2021.01.215.
